# Supplementary material for: Design-Evolution Synergy Enables New-to-Nature Enzyme Chemistry
Source: bioRxiv. 2026 Oct 2:2026.10.01.754751. Preprint. [Version 1] doi: 10.64898/2026.10.01.754751 (PMC13648690; doi:10.64898/2026.10.01.754751)
Supplement: Supplementary file 1 [file media-1.pdf]

Design-Evolution Synergy Enables New-to-Nature Enzyme Chemistry

Zi-Qi Li,[1] Ruijie K. Zhang,[2,⊥] Pei-Pei Xie,[3,⊥] Jacob Kelly,[2,⊥] Yu Zhang,[3,⊥] Zi-Yang Qin,[1,⊥] Theophile Lambert,[1] Orna Mukhopadhyay,[1] Deirdre Hanley,[1] Amy E. Danson,[2] Josh Abramson,[2] Sukhdeep Singh,[2] Zachary Wu,[2] Pushmeet Kohli,[2] Peng Liu,[3,*] Jue Wang,[2,*] Frances H. Arnold[1,*]

[1]Division of Chemistry and Chemical Engineering, California Institute of Technology, Pasadena, California 91125, United States

[2]Google Deepmind, London, United Kingdom

[3]Department of Chemistry, University of Pittsburgh, 219 Parkman Avenue, Pittsburgh, Pennsylvania 15260, United States

*frances@cheme.caltech.edu
wajue@deepmind.com
pengliu@pitt.edu

Table of Contents

General Information 3

Analytical Instruments and Source of Commercial Reagents 3
Cloning and Mutagenesis 3
Transformation of BL21 *E. coli* cells 6
Cell Culture and Protein Expression 7
Protein Purification 7
Hemochrome Assay for the Determination of Hemoprotein Concentration 8
Analytical Reaction Setup and Product Quantification 8
Preparative-Scale Reaction Setup and Product Quantification 9

General Procedures for the Preparation of Starting Material 10

General procedure A for the synthesis of alkyl azide substrates 10
General procedure B for the synthesis of 1m 11
Selected Small-molecule Catalysts for Intramolecular C–H Amination 12

DNA Sequences and Protein Sequences 13

Part 1: *Tm*Pgb-PIP variants 13
Part 2: GDM-NT variants from recycled scaffold design 15
Part 3: GDM-NT variants from directed evolution and additional protein engineering thereof. 17
Part 4: GDM-NT variants from the third round of "novel scaffold" design 20
Part 5: GDM-NT variants used as negative control for structural analysis 22

Design Pipeline Details 24

Initial Design Details (First Round) 24
Partial Diffusion Details (Second Round) 24
Structural Diversity of Designs 27
Motif-Informed Design Details (Third Round) 29

Design with No-Protein Motif 32
Comparison between GDM-NT-401 and *Tm*Pgb-PIP-G7 35

Analytical-Scale Enzymatic Reaction Yield Analysis and Calibration Curves for Products 40

Part 1: *Tm*Pgb-PIP lineage analysis. 40
Part 2: *De novo* design with recycled scaffold data analysis. 42
Part 3: Evolution from GDM-NT-226 and GDM-NT-362. 43
Part 4: Additional engineering from top hit in error-prone library 44
Part 5: Reaction condition optimization using purified protein. 45
Part 6: Solvent stability test for GDM-NT-401 under high concentration of ethanol. 46
Part 7: T_{50} measurement for GDM-NT-401 and *Tm*Pgb-PIP-G7 47
Part 8: Third round of design using refined motifs. 48
Part 9: Substrate scope analysis. 49

Analytical-Scale Enzymatic Reaction Regioselectivity Analysis 61

Part 1: Regioselectivity analysis of *Tm*Pgb-PIP lineage. 61
Part 2: Regioselectivity analysis of GDM-NT variants 61
Part 3: Regioselectivity analysis of substrate scope study. 62

Computational Details and Additional Computational Results 73

Representative Analytical Data of Starting Materials 97

Supplementary References 98

Representative NMR spectrum 100

Representative Chiral HPLC-MS data 104

Part 1: Regio- and enantioselectivity analysis of 2a and 3a on reactions performed with *Tm*Pgb-PIP lineage. 104
Part 2: Regio- and enantioselectivity analysis of 2a and 3a on reactions performed with GDM-NT variants. 107
Part 3: Regio- and enantioselectivity analysis of substrate scope study on reactions performed with *Tm*Pgb-PIP-G7-5455 114
Part 4: Regio- and enantioselectivity analysis of substrate scope study on reactions performed with GDM-NT-401. 127

General Information

Analytical Instruments and Source of Commercial Reagents

Unless otherwise stated, all materials were used as received from commercial sources without further purification. Erlenmeyer flasks used for cell-culture and protein expression were purchased from VWR and autoclaved before use. Glassware used for analytical reaction set-up and HPLC analysis were purchased from Agilent and used as received. All solvents were purchased from MilliporeSigma, VWR, Thermofisher and used as received. Silica gel chromatography was carried out using Biotage® Sfär chromatography column. ^{1}H and ^{13}C NMR spectra were recorded on a Bruker Prodigy 400 MHz instrument (400 MHz for ^{1}H NMR, 101 MHz for ^{13}C NMR, and 376 MHz for ^{19}F NMR). ^{1}H NMR spectra were reported relative to Me_4Si (δ 0.0) in $CDCl_3$ or residual solvent signals (DMSO-d_5 at δ 2.54 or HDO at δ 4.79) unless otherwise stated. ^{13}C NMR spectra were calibrated to residual solvent signals ($CDCl_3$ at δ 77.16 or DMSO-d_6 at δ 39.52). Data for ^{1}H NMR are reported as follows: chemical shift (δ ppm), multiplicity (s = singlet, d = doublet, t = triplet, q = quartet, p = pentet, sext = sextet, m = multiplet, dd = doublet of doublets, dt = doublet of triplets, ddd = doublet of doublet of doublets), coupling constant (Hz), integration. Sonication was performed using a Qsonica Q500 sonicator. High-resolution mass spectra were obtained at the California Institute of Technology Mass Spectral Facility. Samples were analyzed by field ionization (FI) using a JEOL AccuTOF GC-Alpha (JMS-T2000GC) mass spectrometer interfaced with an Agilent 8890 GC system. Ions detected by FI are radical cations.

Chemical reactions were monitored using thin layer chromatography (Merck 60 silica gel plates) and a UV lamp for visualization. Analytical reverse-phase high-performance liquid chromatography-mass spectroscopy (HPLC-MS) was carried out using an Agilent 1260 series instrument and an Agilent C18 column (InfinityLab Poroshell 120 EC-C18, 4.6 × 50 mm, 2.7 µm; Part Number: 699975-902T) with water and acetonitrile, both containing 0.1% acetic acid, as the mobile phase. Analytical chiral polar protic HPLC-MS was conducted using an Agilent 1260 series instrument with methanol (containing 0.2 w% ammonium formate) and water (pH 4.0, with 20 mM ammonium formate). Enantiomers were separated using *dual* Agilent InfinityLab Poroshell 120 Chiral-V, 2.7 µm, 2.1 × 150 mm.

Escherichia coli cells were grown using Luria-Bertani or Terrific Broth medium (Research Products International) with 0.1 mg/mL ampicillin (LB_{amp} or TB_{amp}). Phusion polymerase, and *Taq* polymerase were purchased from New England Biolabs (NEB, Ipswich, MA). Plasmids were purified by miniprep (New England Biolabs). Sanger sequencing was performed by Laragen, Inc. (Culver City, CA) using the T7 promoter primer and T7 terminator primers. Full plasmid sequencing was performed by Plasmidsaurus Inc. (Monrovia, CA). M9–N minimal media (abbreviated as M9–N buffer, pH 7.2) were used as buffering systems for whole cells. M9–N buffer contains 47.7 mM Na_2HPO_4, 22.0 mM KH_2PO_4, 8.6 mM NaCl, 2.0 mM $MgSO_4$, and 0.1 mM $CaCl_2$.

Cloning and Mutagenesis

***Tm*Pgb-PIP** variants:

All genes were encoded with a C-terminal 6×His tag for purification and inserted between the *Nde*I and *Xho*I restriction sites in the pET22b(+) vector.

GDM-NT variants:

DNA for clonal genes was purchased from Twist Bioscience (CA, USA). Genes were inserted into a modified pTriEx-4 vector to contain an N-terminal 8×His tag and HRV-3C cleavage site. For all genes, codons were optimised for *E. coli* using Twist Bioscience.

Table S1. Primers used in site-saturation mutagenesis and error-prone PCR for ***Tm*Pgb-PIP** variants.

Primers	Sequence (5' → 3')
**Universal Forward (UniF)**	CCA ACT TAC TTC TGA CAA CGA TCG GAG GAC CGA AGG AGC TAA CCG CTT TTT TGC
**Universal Reverse (UniR)**	CGA TCG TTG TCA GAA GTA AGT TGG CCG CAG TGT TAT CAC TCA TGG TTA TGG CAG
**pET-005**	GAA ATA ATT TTG TTT AAC TTT AAG AAG GAG ATA TAC ATA TG
**pET-006**	GCC GGA TCT CAG TGG TGG TGG TGG TGG TGC TCG AG
**pET-007**	CAT ATG TAT ATC TCC TTC TTA AAG TTA AAC AAA ATT ATT TC
**pET-008**	CTC GAG CAC CAC CAC CAC CAC CAC TGA GAT CCG GC

Table S2. Primers used in error-prone PCR for **GDM-NT** variants.

Primers	Sequence (5' → 3')
**pTriEx-005**	GGTCTGGAAGTGCTGTTTCAAGGCCCGGGATCCAGTGGC
**pTriEx-006**	CAGCCTGCACCTGAGGTTAATCACTTAGTGTGATC
**pTriEx-007**	ATTAACCTCAGGTGCAGGCTGCCTATCAGAAGG
**pTriEx-008**	AAACAGCACTTCCAGACCGCTACTGCCATGATGGTG

Site-saturation mutagenesis

Site-saturation mutagenesis was performed using the 22-codon method (*1*). Primers including the three 22-codon trick codons (NDT, VHG, and TGG) and primers within the ampicillin resistance cassette (**UniF** and **UniR**) were used to amplify the plasmid of interest in two pieces, with an overlap for Gibson assembly[2] in the gene encoding the protein of interest (excluding where the mutations are introduced) and an overlap for Gibson assembly in the gene encoding β-lactamase (which confers ampicillin resistance). This two-piece assembly can prevent misassembled constructs from presenting antibiotic resistance. The PCR reactions were performed according to the protocol in **Table S3** and **Table S4**. The PCR products were digested with *Dpn*I (1 µL) at 37 ºC for at least 1 hour. The resulting PCR products with SYBR Gold nucleic acid stain were loaded on 1.5% agarose gels, purified via electrophoresis, and visualized on a blue transilluminator. The DNA bands of interest were excised, and the DNA was extracted with New England Biolabs DNA cleanup and gel extraction kit. The two linear PCR products for a given site-saturation library were assembled via Gibson assembly.

Table S3. PCR reaction components for SSM using Phusion polymerase.

PCR Reaction Components	Amount (µL)
PCR water	32
5× HF buffer	10
10 mM dNTPs mix	1
DMSO	0.5
10 µM Forward Primer	2.5
10 µM Reverse Primer	2.5
30 ng/µL Plasmid	0.5
Phusion® High-Fidelity polymerase	0.7

Table S4. Thermocycler conditions for Phusion PCR amplification

Step	Temperature (ºC)	Time (min:s)	Number of Cycles
Initial Denaturation	98	00:30	1
Denaturation	98	00:10	30
Annealing	55	00:20	
Extension	72	02:30	
Extension	72	10:00	1
Hold	10	-	

Random mutagenesis

Random mutations using error-prone PCR (*ep*PCR) were introduced using Taq polymerase with the addition of varying $MnCl_2$ concentrations (300–600 μM) following a reported protocol. Primers **005** and **006** were used to amplify the enzyme's cassette in the plasmid of interest. The PCR reactions were performed according to the protocol in **Table S5** and **Table S6**. The amplified fragments were purified by gel electrophoresis (1.5% agarose gel) with SYBR Gold nucleic acid stain. The DNA bands of interest were excised, and the DNA was extracted with New England Biolabs DNA cleanup and gel extraction kit. The PCR product was cloned into an empty pET22b (+) or pTriEx vector between primer sites **007** and **008** via Gibson assembly. The empty pET22b (+) or pTriEx vector can be generated from the parent plasmid using primers **007** and **008** following slightly modified protocol in **Table S3** and **Table S4** (4 min instead of 2.5 min extension time). Libraries generated at different $MnCl_2$ concentrations were tested to determine the optimal balance of high gene diversity and low rate of inactivation. The chosen library was further tested to find improved variants.

Table S5: PCR reaction components for *ep*PCR.

Reaction Components (μL)	$MnCl_2$ Concentration (μM)			
	300	400	500	600
10–30 ng/μL Plasmid	1	1	1	1
10 mM Forward Primer **005**	2	2	2	2
10 mM Reverse Primer **006**	2	2	2	2
10 mM dNTPs mix	4	4	4	4
1 mM $MnCl_2$	30	40	50	60
10× Standard Taq buffer	10	10	10	10
Taq DNA polymerase	1.6	1.6	1.6	1.6
PCR water	49.4	39.4	29.4	19.4

Table S6: Thermocycler conditions for Taq PCR amplification

Step	Temperature (ºC)	Time (min:s)	Number of Cycles
Initial Denaturation	95	05:00	1
Denaturation	95	00:30	30
Annealing	55	00:30	
Extension	72	01:00	
Extension	72	05:00	1
Hold	10	-	

Staggered extension process (StEP) PCR

Staggered extension process (StEP) PCR was performed using an equimolar mixture of plasmids of the variants to be recombined (*2*). This method recombines mutations that lead to improvements in function with high efficiency by performing PCR cycles with varying extension temperatures and shorter extension times. This protocol involves two PCR reactions. Primers **005** and **006** (1 µM) were used to amplify the region coding the enzyme of interest using the protocol in **Table S7** and **Table S8**. Multiple reactions are set up at varying annealing temperatures (50 → 70 ºC). The PCR product was digested with *Dpn*I (1 µL) at 37 ºC for at least 1 hour. The resulting product with SYBR Gold nucleic acid stain was loaded on 1.5% agarose gels and visualized on a blue transilluminator. The reaction that works at the lowest temperature is typically moved forward to the second PCR. The DNA band of interest was excised and extracted with New England Biolabs DNA cleanup and gel extraction kit. The resulting DNA product is further amplified using the protocol described for site-saturation mutagenesis (**Table S3** and **Table S4**) using primers **005** and **006** (10 µM). The resulting product with SYBR Gold nucleic acid stain was loaded on 1.5% agarose gels and visualized on a blue transilluminator. The DNA band of interest was excised, and the DNA was extracted with New England Biolabs DNA cleanup and gel extraction kit. The PCR product was cloned into a pET22b (+) vector between primer sites **007** and **008** via Gibson assembly (*3*).

Table S7: PCR reaction 1 components for StEP recombination.

**PCR reaction components**	**Amount (µL)**
PCR water	14.27 (adjustable)
10× Standard Taq buffer	2
10 mM dNTPs mix	0.4
Forward Primer (1 µM)	1
Reverse Primer (1 µM)	1
Mix Plasmids (250 ng total)	1 (adjustable)
Taq Polymerase	0.33

Table S8: Thermocycler conditions for PCR reaction 1 in StEP recombination.

**Step**	**Temperature (ºC)**	**Time (min:s)**	**Number of Cycles**
Initial Denaturation	95	5:00	1
Denaturation	95	00:10	
Annealing	55	00:01	120
Extension	Gradient (50 → 72)	00:01	
Hold	10	-	

Transformation of BL21 *E. coli* cells

Transformation of individual variants:

Plasmids constructed from Gibson assembly were used to transform *E. coli* T7 express BL21(DE3) electro-competent cells by electroporation. SOC medium was added to rescue the cells. Subsequently, the cells were incubated at 37 °C for 30 minutes before being plated on Luria Bertani medium (Research Products International) supplemented with carbenicillin (0.1 mg/mL) agarose plates. The agarose plates with the plated cells are incubated at 37 ºC for 12–16 h before the libraries are picked.

Transformation of multiple variants on 96-well plate:

100 µL of T7 Express chemically competent cells were aliquoted into a PCR strip on ice. Using a multichannel pipette, 5 µL of cells and 1 µL of DNA construct were added to each well of a sterile, cone-shaped 96-well plate. Following a 30-minute incubation on ice, the plate was heat-shocked at 42 °C for 10 seconds and immediately returned to ice for 5 minutes. Cells were rescued with 100 µL of SOC medium and incubated at 37 °C for 30 minutes for outgrowth. Following outgrowth, 10 µL of each culture was inoculated into a 96-deep well plate preloaded with 400 µL/well of LB medium supplemented with ampicillin (100 µL/mL) (TB_{amp}). These pre-cultures were incubated at 37 °C, 80% humidity, and 220 rpm shaking speed for 7–8 hours. Subsequently, 50 µL of each pre-culture was used to inoculate a secondary 96-deep well plate containing 950 µL/well of Terrific Broth supplemented with 100 µg/mL ampicillin (TB_{amp}) per well. This expression culture was shaken at 37 °C and 220 rpm until an OD_{600} of 0.8–1.0 was reached (typically 2–2.5 hours). The plates were then chilled on ice for 30–45 minutes and induced with 0.5 mM β-d-1-thiogalactopyranoside (IPTG) and 1.0 mM 5-aminolevulinic acid (ALA) (final concentrations). Protein expression was conducted at 22 °C and 220 rpm for 16–20 hours.

Cell Culture and Protein Expression

Analytical-scale protein expression on 96-Well Plate

Single colonies from LB_{carb} agar plates were picked using sterile toothpicks and cultured in deep-well 96-well plates containing LB_{amp} (500 µL/well) at 37 ºC, 80% humidity, and 220 rpm shaking speed for 7–8 hours. Subsequently, TB_{amp} (950 µL/well) in a deep-well plate was inoculated with an aliquot (50 µL/well) of these pre-cultures and allowed to shake for 2–2.5 hours at 37 ºC, 80% humidity, and 220 rpm until an optical density at 600 nm (OD_{600}) of 0.8–1.0. The plates were then rested on ice for 30–45 minutes and induced with 0.5 mM isopropyl *β*-D-1-thiogalactopyranoside (IPTG) and 1.0 mM 5-aminolevulinic acid (ALA) (final concentrations). Protein expression was conducted at 22 ºC, and 220 rpm for 16–20 hours.

Mid-scale protein expression in Erlenmeyer flasks

Single colonies from LB_{carb} agar plates were picked using sterile toothpicks and cultured in a culture tube containing LB_{amp} (5 mL/tube) at 37 ºC with 220 rpm shaking speed for 7–8 hours. Subsequently, TB_{amp} (50 mL) in a 125-mL Erlenmeyer flask was inoculated with an aliquot (1 mL) of these pre-cultures and allowed to shake for 2–2.5 hours at 37 ºC, and 220 rpm until an optical density at 600 nm (OD_{600}) of 0.8–1.0. The culture was then rested on ice for 30–45 min and induced with 0.5 mM IPTG and 1.0 mM ALA (final concentrations). Protein expression was conducted at 22 ºC and 180 rpm for 16–20 hours.

Protein Purification

The engineered protoglobin variants were expressed in *Escherichia coli* BL21(DE3) cells following slightly modified procedure in section C. A preculture of engineered protoglobin variants were cultured in an Erlenmeyer flask containing LB_{amp} (50 mL) at 37 ºC with 220 rpm shaking speed for 6 hours. Subsequently, TB_{amp} (1 L) in a 4-L Erlenmeyer flask was inoculated with 50 mL of the preculture and allowed to shake for 2–2.5 hours at 37 ºC, and 180 rpm until an optical density at 600 nm (OD_{600}) of 0.8–1.0. The culture was then rested on ice for 30–45 min and induced with 0.5 mM IPTG and 1.0 mM ALA (final concentrations). Protein expression was conducted at 22 ºC and 180 rpm for 16–20 hours and collected by centrifugation at 4,000 r.p.m. at 4 °C. After cell lysis by sonication, and centrifugation (20,000 g, 25 min, and 4 ºC), the proteins were purified by Ni-affinity chromatography. The clarified lysate was transferred to a nickel nitriloacetic acid (Ni-NTA) column equilibrated with equilibration buffer (50 mM KPi buffer, 200 mM NaCl, pH 8.0). The resin was washed with 200 ml Ni-NTA wash buffer (50 mM KPi buffer, 200 mM NaCl, 20 mM imidazole, pH 8.0). The proteins were eluted with Ni-NTA elution buffer (50 mM KPi buffer, 200 mM NaCl, 200 mM imidazole, pH 8.0). After elution, the proteins were dialysed in 50 mM KPi buffer (pH 8.0) using 3 KDa dialysis memberane and concentrated in 3kDa centrifugal filters. The haem-loaded protein concentration was determined using hemochrome assay.

Hemochrome Assay for the Determination of Hemoprotein Concentration

E. coli cells after expression were pelleted by centrifugation (4,000 g, 8 min, and 4 ºC). TB_{amp} medium was removed. Harvested cell pellets were resuspended to M9–N medium (pH 8.0) supplemented with D-glucose (25 mM final concentration) to OD_{600} = 50. The same cell suspension was used for reaction set-up and determination of protein concentration. Protein concentration in the cell was determined using the hemochrome assay on cell lysate (*4*). Lysate was obtained by sonication using a Qsonica Q500 sonicator (6 minutes, 1 second on, 2 seconds off, 35% amplitude, on wet ice). The cell debris was removed by centrifugation (14,000 g, 10 minutes, 4 °C). To a cuvette, 500 µL of the lysate and 500 µL of solution I [0.2 M NaOH, 40% (v/v) pyridine, 0.5 mM $K_3Fe(CN)_6$] were added. The UV-Vis spectrum (380–620 nm) of the oxidized state Fe(III) was recorded immediately. Sodium dithionite (10 µL of 0.5 M solution in water) was added, and the UV-Vis spectrum of the reduced state Fe(II) was recorded immediately. The protein concentration was calculated using the extinction coefficient and dilution factor (2× dilution in volume): $\varepsilon\ [557_{reduced} - 540_{oxidized}] = 23.98\ mM^{-1}cm^{-1}$. The hemochrome assay detects total heme level, which is a good approximation of over-expressed heme enzyme.

Analytical Reaction Setup and Product Quantification

Reaction set-up in 96-well plate

E. coli cells after expression in 96-well plates were pelleted by centrifugation (4,000 g, 8 min, and 4 ºC). TB_{amp} medium was removed. Harvested cell pellets were resuspended with 380 µL M9–N medium (pH 7.2). The resuspended cell in 96-well plates were introduced to an anaerobic chamber (coy chamber, oxygen level: <60 ppm). Alkyl azides **1** (20 µL, 0.1 M stock in ethanol) were added using a multi-channel pipette. The reactions were shaken at room temperature overnight at 700 rpm.

Reaction set-up in 2-mL vials with adjusted OD_{600} (whole-cell)

E. coli cells after expression in 125-mL Erlenmeyer flasks were pelleted by centrifugation (4,000 g, 8 min, and 4 ºC). TB_{amp} medium was removed. Harvested cell pellets were resuspended to M9–N medium (pH 7.2) to OD_{600} = 30. Resuspended cells (380 µL) were aliquoted to 2-mL screw cap vials and introduced to an anaerobic chamber (coy chamber, oxygen level: <60 ppm). Alkyl azides **1** (20 µL, 0.1 M stock in ethanol) were added using a multi-channel pipette. The reactions were shaken at room temperature overnight at 700 rpm.

Reaction set-up in 2-mL vials with adjusted haem loading (purified protein)

An aliquot of purified ***Tm*Pgb-PIP-G7** was diluted with M9–N buffer (50 mM, pH 7.2) to a final volume of 380 µL in a 2-mL vial, giving a haem concentration of 20 µM. Solution of purified protein was introduced to an anaerobic chamber (coy chamber, oxygen level: <60 ppm). Alkyl azide **1** (20 µL of a 0.1 M stock solution in ethanol) was added in a single portion under anaerobic conditions. The vial was sealed with a screw-top cap and shaken at 700 rpm overnight at room temperature or 37 °C.

An aliquot of purified **GDM-NT-401** was diluted with M9–N buffer (50 mM, pH 7.2) to a final volume of 370 µL in a 2-mL vial, giving a haem concentration of 10 µM. Solution of purified protein was introduced to an anaerobic chamber (coy chamber, oxygen level: <60 ppm). $Na_2S_2O_4$ (10 µL of a 0.1 M stock solution in M9–N buffer, pH 7.2), alkyl azide **1** (20 µL of a 0.1 M stock solution in ethanol) was added sequentially under anaerobic conditions. The vial was sealed with a screw-top cap and shaken at 700 rpm overnight at room temperature.

Reaction work-up on 96-well plate

After the reaction was completed, the vials were removed from the coy chamber. Acetonitrile (780 µL) was used to quench the reaction. 1,2,3-Trimethoxybenzene (5 mM final concentration) was used as internal standard (20 µL, 0.1 M stock in acetonitrile). The deep-well plates were shaken at room temperature for 20

min at 700 rpm and centrifuged (5,000 × g, 20 min, 4 °C). A sample of the supernatant (0.2 mL) was transferred to a 96-well plate and sealed with a heat plate sealer for reverse-phase HPLC-MS analysis. Products were identified and quantified by analyzing the absolute ion count on the single ion channel or comparing to a calibration curve generated with chemically synthesized standard products and 1,2,3-trimethoxybenzene as internal standard. Enantiomeric excess (*ee*) of the reactions were determined by analyzing an internal-standard-free sample using polar ionic HPLC-MS.

Reaction work-up for analytical reactions in 2-mL vials

After the reaction was completed, the vials were removed from the coy chamber. Acetonitrile (780 μL) was used to quench the reaction. 1,2,3-Trimethoxybenzene (5 mM final concentration) was used as internal standard (20 μL, 0.1 M stock in acetonitrile). The mixture was shaken at room temperature for 20 min at 700 rpm, transferred to an Eppendorf tube and centrifuged (5,000 × g, 20 min, 4 °C). A sample of the supernatant (0.2 mL) was transferred to a vial equipped with a glass insert for reverse-phase HPLC-MS analysis. Products were identified and quantified by comparing to a calibration curve generated with chemically synthesized standard products and 1,2,3-trimethoxybenzene as internal standard. Enantiomeric excess (*ee*) of the reactions were determined by analyzing an internal-standard-free sample using polar ionic HPLC-MS.

Preparative-Scale Reaction Setup and Product Quantification

Preparative reaction set-up

An aliquot of purified ***Tm*Pgb-PIP-G7** was diluted with M9–N buffer (50 mM, pH 7.2) to a final haem concentration of 20 μM. Alternatively, *e. coli* expressing **GDM-NT-401** were resuspended to M9–N medium (pH 7.2) to OD_{600} = 30. Solution of purified protein or resuspended cells (190 mL) were introduced to an anaerobic chamber (coy chamber, oxygen level: <60 ppm). Alkyl azide **1** (10 mL of a 0.1 M stock solution in ethanol) was added in a single portion under aerobic conditions. The flask was sealed with a screw-top lid and shaken at 220 rpm for 2 days at 37 °C.

Preparative reaction work-up

Upon completion of the reaction, acetonitrile (400 mL) was added to quench the reaction and precipitate the protein. The precipitant was removed by filtration through a pad of Celite. Excess acetonitrile was removed under reduced pressure. The remaining aqueous solution was acidified to pH 3–4 with 1 M HCl and washed with ethyl acetate (150 mL × 3). The aqueous phase was then basified to pH 10 with 2 M NaOH and extracted with ethyl acetate (150 mL × 3). The combined organic layers were dried over Na_2SO_4 and concentrated under reduced pressure. The resulting residue was characterized by ^{1}H NMR spectroscopy and was found to be of sufficient purity without further purification.

General Procedures for the Preparation of Starting Material

General procedure A for the synthesis of alkyl azide substrates

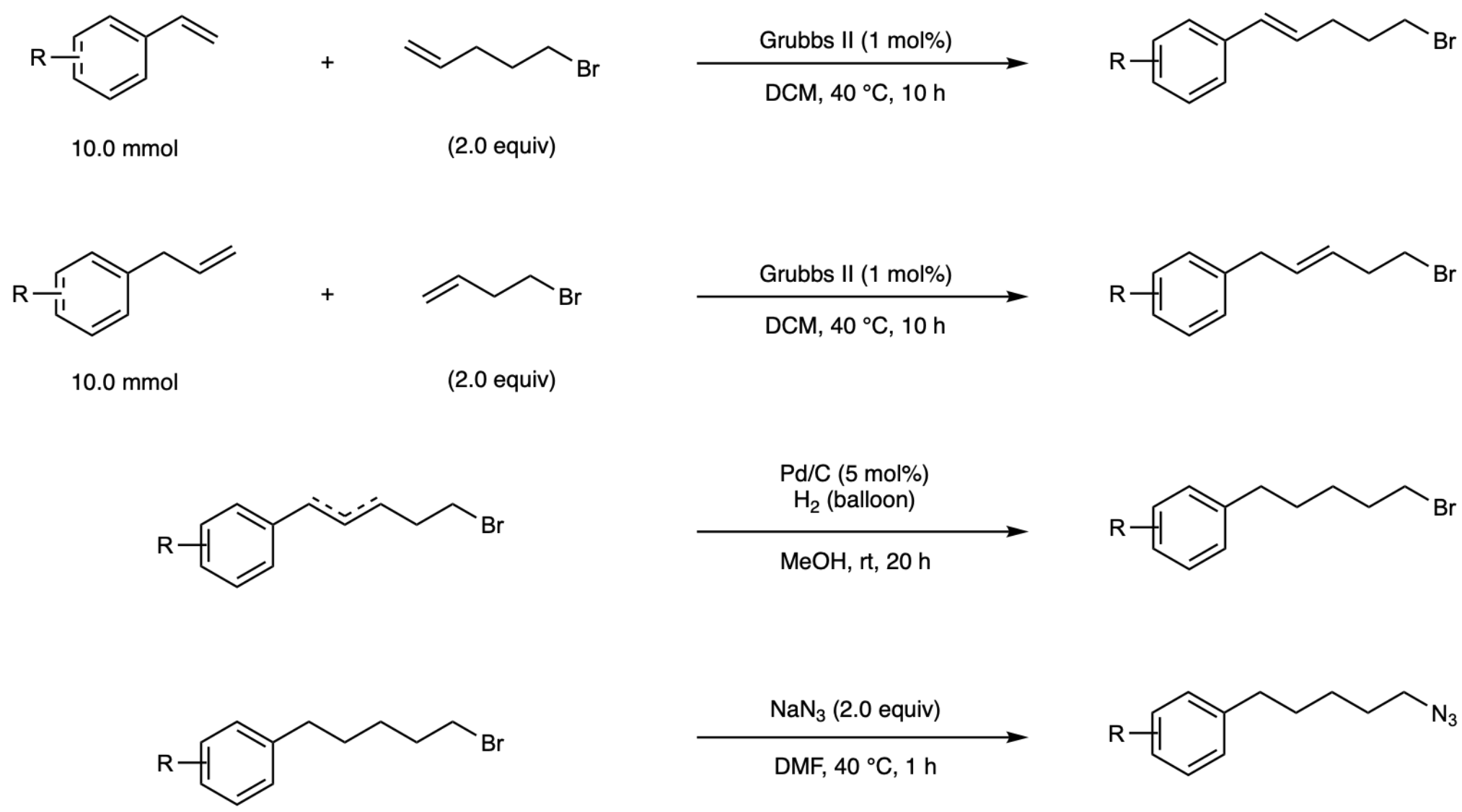

Scheme S1. General procedure A for the synthesis of alkyl azides through metathesis, hydrogenation, and azidation.

Olefin metathesis

Following a procedure slightly modified from literature (*5*), to a solution of styrene (10.0 mmol) and 5-bromo-1-pentene (20.0 mmol) in DCM (0.3 M) was added Grubbs II (1 mol%). The mixture was stirred at 40 °C for 10 h or until full conversion of the styrene monitored by thin-layer chromatography (TLC). Upon completion, the organic solvent was removed under reduced pressure, and the residue was subjected to next step without further purification. Alternatively, the same procedure could be applied for the combination of allylbenzene (10.0 mmol) and 4-bromo-1-butene (20 mmol).

Hydrogenation

To a solution of alkenyl bromide in MeOH (0.5 M) was added palladium on carbon (5 mol%). The reaction mixture was equipped with a hydrogen balloon and stirred for 20 h at room temperature or until full conversion of the styrene monitored by thin-layer chromatography (TLC). Upon completion, the reaction mixture was filtered, and the organic solvent was removed under reduced pressure. The residue was subjected to next step without further purification.

Azidation

To a solution of alkyl bromide in DMF (0.5 M) was added NaN_3 (2.0 equiv). The reaction mixture was stirred at 40 °C for 10 h or until full conversion of the starting material monitored by thin-layer chromatography (TLC). Upon completion, the reaction mixture was quenched with brine. filtered and the organic solvent was removed under reduced pressure. After this time, the reaction was quenched with water. The aqueous solution was extracted with ethyl acetate (50 mL ×3). The combined organic layers were dried over Na_2SO_4. The organic solvent was removed under reduced pressure, and the residue was subjected to flash column chromatography on silica gel with hexane as eluent to afford the 1-(5-azidopentyl)-benzene product.

General procedure B for the synthesis of 1m

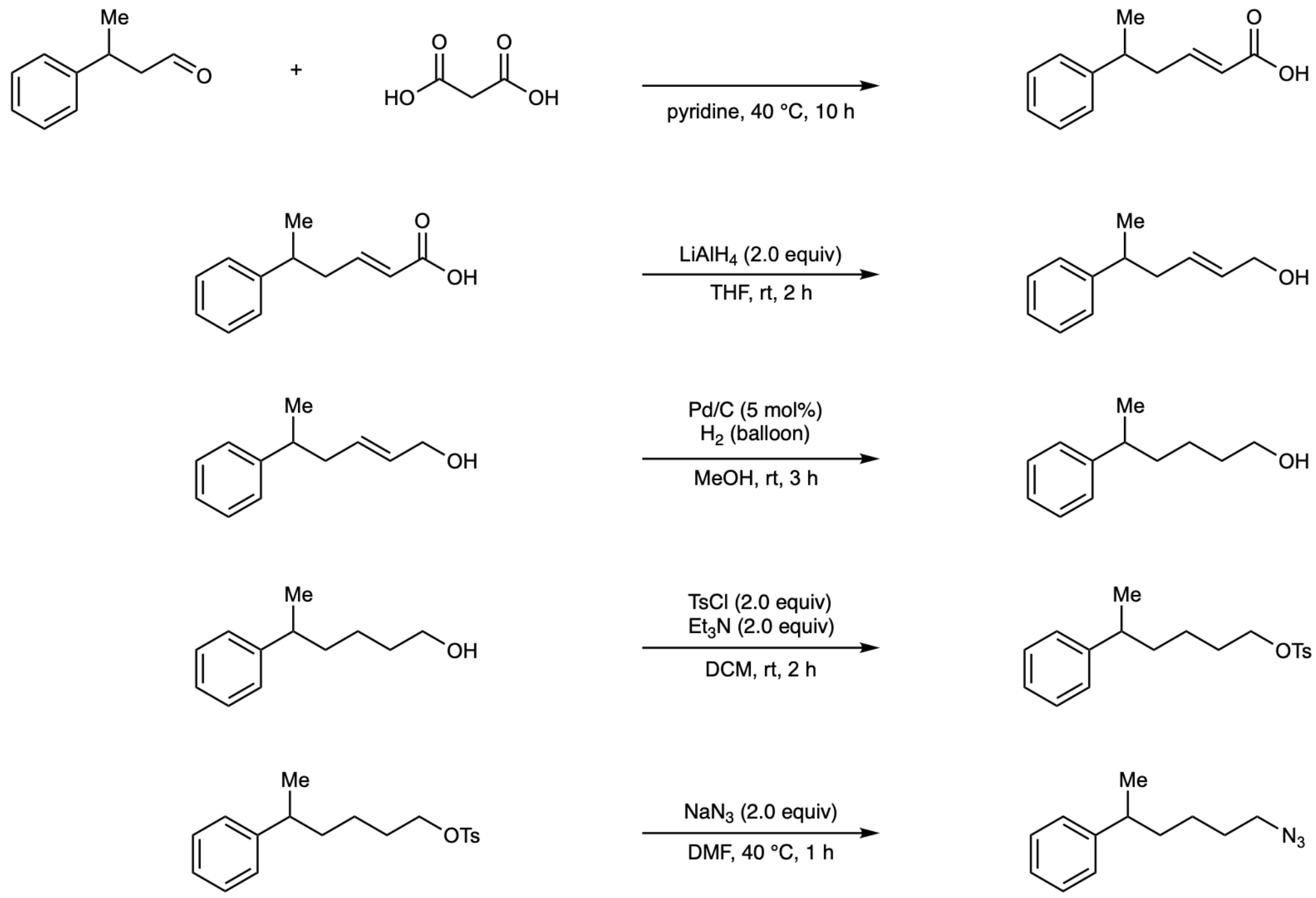

Scheme S2. General procedure B for the synthesis of substrate **1m** through Knoevenagel condensation, reduction, hydrogenation, tosylation, and azidation.

Knoevenagel condensation Doebner modification

The reaction was performed following a modified procedure from literature. A round-bottom flask containing a mixture of 3-phenylbutanal (12.0 mmol) and malonic acid (10.0 mmol) in pyridine (0.5 M) was equipped with a balloon. The mixture was stirred at 40 °C for 10 h or until full conversion of the substrate monitored by thin-layer chromatography (TLC). Upon completion, the organic solvent was removed under reduced pressure, and the residue was subjected to next step without further purification.

Reduction of carboxylic acid

To a solution of the α,β-unsaturated acid in THF, $LiAlH_4$ (2.0 equiv) was added at 0 °C. The reaction was gradually warmed to room temperature and stirred for 2 h or until full conversion of the substrate monitored by thin-layer chromatography (TLC). Upon completion, the reaction was quenched with cold saturated NH_4Cl solution. The aqueous solution was extracted with ethyl acetate (200 mL ×3). The combined organic layers were dried over Na_2SO_4. The organic solvent was removed under reduced pressure, and the residue was subjected to next step without further purification.

Hydrogenation

To a solution of alkenyl alcohol in MeOH (0.5 M) was added palladium on carbon (5 mol%). The reaction mixture was equipped with a hydrogen balloon and stirred for 20 h at room temperature or until full conversion of the styrene monitored by thin-layer chromatography (TLC). Upon completion, the reaction mixture was filtered, and the organic solvent was removed under reduced pressure. The residue was subjected to next step without further purification.

Tosylation

To a solution of alkyl alcohol in DCM (0.5 M) was added TsCl (2.0 equiv) and triethylamine (2.0 equiv). The reaction mixture was stirred at room temperature for 2 h or until full conversion of the styrene monitored by thin-layer chromatography (TLC). Upon completion, the reaction mixture was quenched with brine. The aqueous solution was extracted with DCM (200 mL ×3). The combined organic layers were dried over Na_2SO_4. The organic solvent was removed under reduced pressure, and the residue was subjected to next step without further purification.

Azidation

To a solution of tosyl protected alkyl alcohol in DMF (0.5 M) was added NaN_3 (2.0 equiv). The reaction mixture was stirred at 40 °C for 10 h or until full conversion of the starting material monitored by thin-layer chromatography (TLC). Upon completion, the reaction mixture was quenched with brine. filtered and the organic solvent was removed under reduced pressure. After this time, the reaction was quenched with water. The aqueous solution was extracted with ethyl acetate (50 mL ×3). The combined organic layers were dried over Na_2SO_4. The organic solvent was removed under reduced pressure, and the residue was subjected to flash column chromatography on silica gel with hexane as eluent to afford the 1-(5-azidopentyl)-benzene product.

Selected Small-molecule Catalysts for Intramolecular C–H Amination

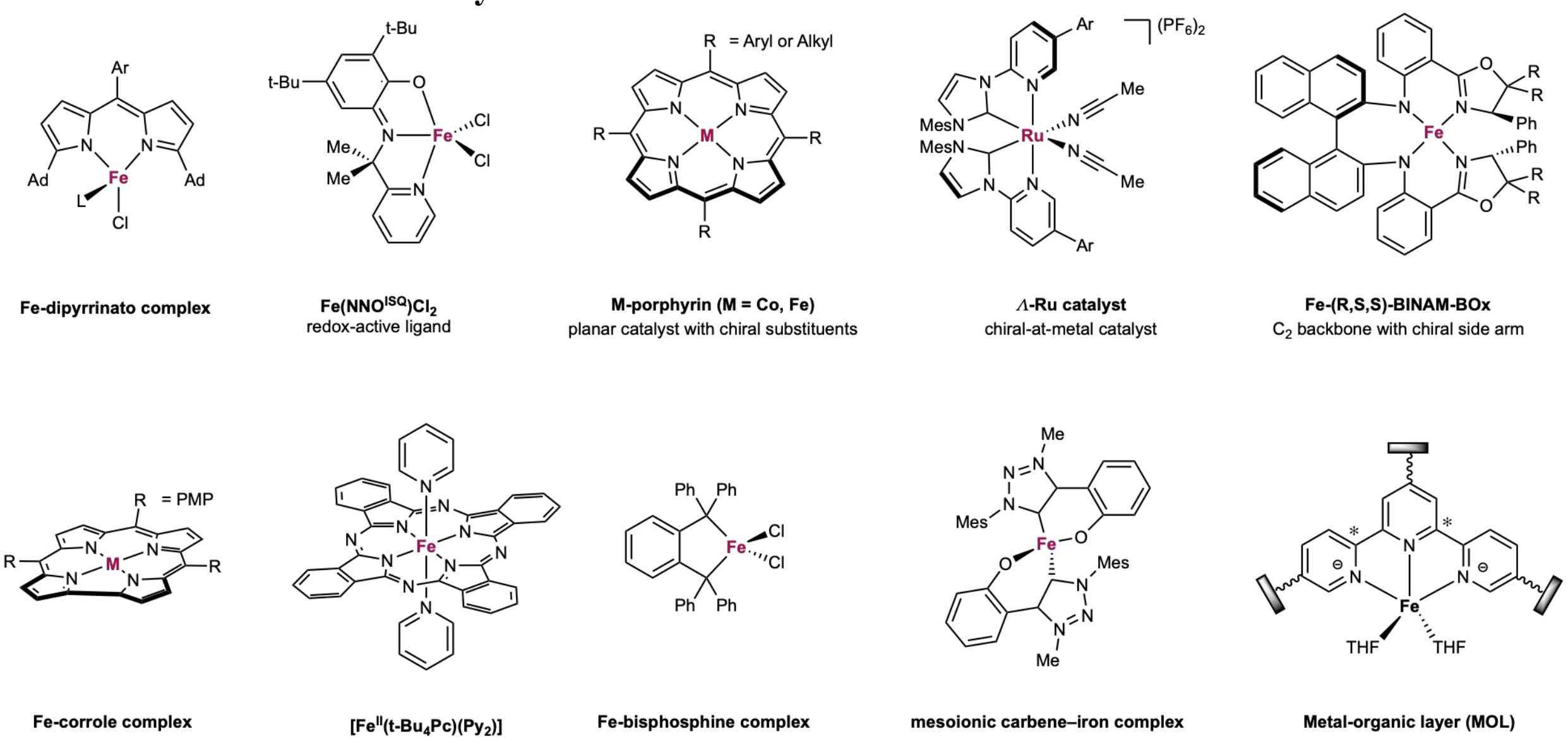

Scheme S3. Selected examples of small-molecule catalysts for Intramolecular C–H Amination of Alkyl azides.

DNA Sequences and Protein Sequences

Part 1: *Tm*Pgb-PIP variants

DNA sequence of ***Tm*Pgb-wt**:

ATGCCGCAAATTCCCGGTTACACTTATGGAGATCCCGCCTTACCTCCCAGTCCCGTTTCTTTA
GAGGAATTGGAGCGTCTGAAGGCTTGCTTGCTGTGGACGGAGGAGGATGATAAAGCATTAG
AGCAGGCCGGTAAAGTATTAGAGGACCAAGTAGAAGAAGTGTTAGATTTATGGTACGGCTT
TGTCGGGAGCCATCCTCATTTACTTCACTATTTCACTGATCCTCAGGGGAACCCGATCCCCGA
CTATCTTGAGCGTGTTCGCCGCCGTTTCGGACAGTGGATTCTGGATACCTGCTTTCGTCCCAA
AGACGAAACCTGGCTTCGTTATCAGCATGAGATTGGCTTACGTCATCATCGCACAAAAAAAA
ACCAAACTGACGGCGTGACCTCCGTCCCACATATTCCGTTGCGCTATTTGATCAGTTTTATTT
ATCCCATTACAGCCACCATCAAACCCTTCCTGACTAAGAAGGGCCACAATCCCGAGGAGGTG
GAGCGTATGTATCAGGCATGGTTCAAGGCAGTTGTATTGCAAGTAGCACTTTGGTCCTATCC
TTACACTCAGCCTGGCGACTTTCTCGAG

Amino acid sequence of ***Tm*Pgb-wt**:

MPQIPGYTYGDPALPPSPVSLEELERLKACLLWTEEDDKALEQAGKVLEDQVEEVLDLWYGFVG
SHPHLLHYFTDPQGNPIPDYLERVRRRFGQWILDTCFRPKDETWLRYQHEIGLRHHRTKKNQTD
GVTSVPHIPLRYLISFIYPITATIKPFLTKKGHNPEEVERMYQAWFKAVVLQVALWSYPYTQPGDF
LE

DNA sequence of ***Tm*Pgb-PIP-G1-5442**:

ATGCCGCAAATTCCCGGTTACACTTATGGAGATCCCGCCTTACCTCCCAGTCCCGTTTCTTTA
GAGGAATTGGAGCGTCTGAAGGCTTGCTTGCTGTGGACGGAGGAGGATGATAAAGCATTAG
AGCAGGCCGGTAAAGTATTAGAGGACCAAGTAGAAGAAGTGTTAGATTTATGGTATGGCTT
TGTCGGGAGCCATCCTCATTTACTTCACTATTTCACTGATCCTCAGGGGAACCCGATCCCCGA
CTATCTTGAGCGTGTTCGCCGCCGTTTCGGACAGTGGATTCTGGATACCTGCTTTCGTCCCAA
AGACGAAACCTGGCTTCGTTATCAGCATGAGATTGGCTTACGTCATCATCGCACAAAAAAAA
ACCAAACTGACGGCGTGACCTCCGTCCCACATATTCCGTTGCGCTATTTGATCAGTTTTATTT
ATCCCACTACAGCCACCATCAAACCCTTCCTGACTAAGAAGGGCCACAATCCCGAGGAGGT
GGAGCGTATGTATCAGGCATGGTTCAAGGCAGTTGTATTGCAAGTAGCACTTTGGTCCTATC
CTTACACTCAGCCTGGCGACTTTCTCGAG

Amino acid sequence of ***Tm*Pgb-PIP-G1-5442**:

MPQIPGYTYGDPALPPSPVSLEELERLKACLLWTEEDDKALEQAGKVLEDQVEEVLDLWYGFVG
SHPHLLHYFTDPQGNPIPDYLERVRRRFGQWILDTCFRPKDETWLRYQHEIGLRHHRTKKNQTD
GVTSVPHIPLRYLISFIYPTTATIKPFLTKKGHNPEEVERMYQAWFKAVVLQVALWSYPYTQPGD
FLE

DNA sequence of ***Tm*Pgb-PIP-G2-5443**:

ATGCCGCAAATTCCCGGTTACACTTATGGAGATCCCGCCTTACCTCCCGGTCCCGTTTCTTTA
GAGGAATTGGAGCGTCTGAAGGCTTGCTTGCTGTGGACGGAGGAGGATGATAAAGCATTAG
AGCAGGCCGGTAAAGTATTAGAGGACCAAGTAGAAGAAGTGTTAGATTTATGGTATGGCTT
TGTCGGGAGCCATCCTCATCTACTTCACTATTTCACTGATCCTCAGGGGAACCCGATCCCCGA
CTATCTTGAGCGTGTTCGCCGCCGTTTCGGACAGTGGATTCTGGATACCTGCTTTCGTCCCAA
AGACGAAACCTGGCTTCGTTATCAGCATGAGATTGGCTTACGTCATCATCGCACAAAAAAAA
ACCAAACTGACGGCGTGACCTCCGTCCCACATATTCCGTTGCGCTATTCGATCAGTTTTATTT
ATCCCACTACAGCCACCATCAAACCCTTCCTGACTAAGAAGGGCCACAATCCCGAGGAGGT
GGAGCGTATGCATCAGGCATGGTTCAAGGCAGTTGTATTGCAAGTAGCACTTTGGTCCTATC
CTTACACTCAGCCTGGCGACTTTCTCGAG

Amino acid sequence of ***Tm*****Pgb-PIP-G2-5443**:

MPQIPGYTYGDPALPPGPVSLEELERLKACLLWTEEDDKALEQAGKVLEDQVEEVLDLWYGFV
GSHPHLLHYFTDPQGNPIPDYLERVRRRFGQWILDTCFRPKDETWLRYQHEIGLRHHRTKKNQT
DGVTSVPHIPLRYSISFIYPTTATIKPFLTKKGHNPEEVERMHQAWFKAVVLQVALWSYPYTQPG
DFLE

DNA sequence of ***Tm*****Pgb-PIP-G3-5444**:

ATGCCGCAAATTCCCGGTTACACTTATGGAGATCCCGCCTTACCTCCCGGTCCCGTTTCTTTA
GAGGAATTGGAGCGTCTGAAGGCTAGCTTGCTGTGGACGGAGGAGGATGATAAAGCATTAG
AGCAGGCCGGTAAAGTCTTAGAGGACCAAGTAGAAGAAGTGTTAGATTTATGGTATGGCTTT
GTCGGGAGCCATCCTCATCTACTTCACTATTTCACTGATCCTCAGGGGAACCCGATCCCCGA
CTATCTTGAGCGTGTTCGCCGCCGTTTCGGACAGTGGATTCTGGATACCTGCTTTCGTCCCAA
AGACGAAACCTGGCTTCGTTATCAGCATGAGATTGGCTTACGTCATCATCGCACAAAAAAAA
ACCAAACTGACGGTGTGACCTCCGTCCCACATATTCCGTTGCGCTATTCGATCAGTTTTATTT
ATCCCACTACAGCCACCATCAAACCCTTCCTGACTAAGAAGGGCCACAATCCCGAGGAGGT
GGAGCGTATGCATCAGGCATGGTTCAAGGCAGTTGTATTGCAAGTAGCACTTTGGTCCTATC
CTTACACTCAGCCTGGCGACTTTCTCGAG

Amino acid sequence of ***Tm*****Pgb-PIP-G3-5444**:

MPQIPGYTYGDPALPPGPVSLEELERLKASLLWTEEDDKALEQAGKVLEDQVEEVLDLWYGFV
GSHPHLLHYFTDPQGNPIPDYLERVRRRFGQWILDTCFRPKDETWLRYQHEIGLRHHRTKKNQT
DGVTSVPHIPLRYSISFIYPTTATIKPFLTKKGHNPEEVERMHQAWFKAVVLQVALWSYPYTQPG
DFLE

DNA sequence of ***Tm*****Pgb-PIP-G4-5445**:

ATGCCGCAAATTCCCGGTTACACTTATGGAGATCCCGCCTTACCTCCCGGTCCCGTTTCTTTA
GAGGAATTGGAGCGTCTGAAGGCTAGCTTGCTGTGGACGGAGGAGGATGATAAAGCATTAG
AGCAGGCCGGTAAAGTCTTAGAGGACCAAGTAGAAGAAGTGTTAGATTTATGGTATGGCTG
GGTCGGGAGCCATCCTCATCTACTTCACTATTTCACTGATCCTCAGGGGAACCCGATCCCCG
ACTATCTTGAGCGTGTTCGCCGCCGTTTCGGACAGTGGATTCTGGATACCTGCTTTCGTCCCA
AAGACGAAACCTGGCTTCGTTATCAGCATGAGATTGGCTTACGTCATCATCGCACAAAAAAA
AACCAAACTGACGGTGTGACCTCCGTCCCACATATTCCGTTGCGCTATTCGATCAGTTTTATT
TATCCCACTACAGCCACCATCAAACCCTTCCTGACTAAGAAGGGCCACAATCCCGAGGAGGT
GGAGCGTATGCATCAGGCATGGTTCAAGGCAGTTGTATTGCAAGTAGCACTTTGGTCCTATC
CTTACACTCAGCCTGGCGACTTTCTCGAG

Amino acid sequence of ***Tm*****Pgb-PIP-G4-5445**:

MPQIPGYTYGDPALPPGPVSLEELERLKASLLWTEEDDKALEQAGKVLEDQVEEVLDLWYGWV
GSHPHLLHYFTDPQGNPIPDYLERVRRRFGQWILDTCFRPKDETWLRYQHEIGLRHHRTKKNQT
DGVTSVPHIPLRYSISFIYPTTATIKPFLTKKGHNPEEVERMHQAWFKAVVLQVALWSYPYTQPG
DFLE

DNA sequence of ***Tm*****Pgb-PIP-G5-5446**:

ATGCCGCAAATTCCCGGTTACACTTATGGAGATCCCGCCTTACCTCCCGGTCCCGTTTCTTTA
GAGGAATTGGAGCGTCTGAAGGCTAGCTTGCTGTGGACGGAGGAGGATGATAAAGCATTAG
AGCAGGCCGGTAAAGTCTTAGAGGACCAAGTAGAAGAAGTGTTAGATTTATGGTATGGCTG
GGTCGGGAGCCATCCTCATCTACTTCACTATTTCACTGATCCTCAGGGGAACCCGATCCCCG
ACTATCTTGAGCGTGTTCGCCGCCGTTTCGGACAGTGGATTCTGGATACCTGCTTTCGTCCCA
AAGACGAAACCTGGCTTCGTTATCAGCATGAGATTGGCTTACGTCATCATCGCACAAAAAAA
AACCAAACTGACGGTGTGACCTCCGTCCCACATATTCCGTTGCGCTATAATATCAGTTTTATT
TATCCCACTACAGCCACCATCAAACCCTTCCTGACTAAGAAGGGCCACAATCCCGAGGAGGT

GGAGCGTATGCATCAGGCATGGTTCAAGGCAGTTGTATTGCAAGTAGCACTTTGGTCCTATC
CTTACACTCAGCCTGGCGACTTTCTCGAG

Amino acid sequence of ***Tm*****Pgb-PIP-G5-5446**:

MPQIPGYTYGDPALPPGPVSLEELERLKASLLWTEEDDKALEQAGKVLEDQVEEVLDLWYGWV
GSHPHLLHYFTDPQGNPIPDYLERVRRRFGQWILDTCFRPKDETWLRYQHEIGLRHHRTKKNQT
DGVTSVPHIPLRYNISFIYPTTATIKPFLTKKGHNPEEVERMHQAWFKAVVLQVALWSYPYTQPG
DFLE

DNA sequence of ***Tm*****Pgb-PIP-G6-5447**:

ATGCCGCAAATTCCCGGTTACACTTATGGAGATCCCGCCTTACCTCCCGGTCCCGTTTCTTTA
GAGGAATTGGAGCGTCTGAAGGCTAGCTTGCTGTGGACGGAGGAGGATGATAAAGCATTAG
AGCAGGCCGGTAAAGTCTTAGAGGACCAAGTAGAAGAAGTGTTAGATTTATGGTATGGCTG
GGTCGGGAGCGAACCTCATCTACTTCACTATTTCACTGATCCTCAGGGGAACCCGATCCCCG
ACTATCTTGAGCGTGTTCGCCGCCGTTTCGGACAGTGGATTCTGGATACCTGCTTTCGTCCCA
AAGACGAAACCTGGCTTCGTTATCAGCATGAGATTGGCTTACGTCATCATCGCACAAAAAAA
AACCAAACTGACGGTGTGACCTCCGTCCCACATATTCCGTTGCGCTATAATATCAGTTTTATT
TATCCCACTACAGCCACCATCAAACCCTTCCTGACTAAGAAGGGCCACAATCCCGAGGAGGT
GGAGCGTATGCATCAGGCATGGTTCAAGGCAGTTGTATTGCAAGTAGCACTTTGGTCCTATC
CTTACACTCAGCCTGGCGACTTTCTCGAG

Amino acid sequence of ***Tm*****Pgb-PIP-G6-5447**:

MPQIPGYTYGDPALPPGPVSLEELERLKASLLWTEEDDKALEQAGKVLEDQVEEVLDLWYGWV
GSEPHLLHYFTDPQGNPIPDYLERVRRRFGQWILDTCFRPKDETWLRYQHEIGLRHHRTKKNQT
DGVTSVPHIPLRYNISFIYPTTATIKPFLTKKGHNPEEVERMHQAWFKAVVLQVALWSYPYTQPG
DFLE

DNA sequence of ***Tm*****Pgb-PIP-G7-5448**:

ATGCCGCAAATTCCCGGTTACACTTATGGAGATCCCGCCTTACCTCCCGGTCCCGTTTCTTTA
GAGGAATTGGAGCGTCTGAAGGCCAGCTTGCTGTGGACGGAGGAGGATGATAAAGCATTAG
AGCAGGCCGGTAAAGTCTTAGAGGACCAAGTAGAAGAAGTGTTAGATTTATGGTATGGCTG
GGTCGGGGGTGAACCTCATCTACTTCACTATTTCACTGATCCTCAGGCGAACCCGATCCCCG
ACTATCTTGAGCGTGTTCGCCGCCGTTTCGGACAGTGGATTCTGGATACCTGCTTTCGTCCCA
AAGACGAAACCTGGCTTCGTTATCAGCATGAGATTGGCTTACGTCATCATCGCACAAAAAAA
AACCAAACTGACGGTGTGACCTCCGTCCCACATATTCCGTTGCGCTATAATATCAGTTTTATT
TATCCCACTACAGCCACCATCAAACCCTTCCTGACTAAGAAGGGCCACAATCCCGAGGAGGT
GGAGCGTATGCATCAGGCATGGTTCAAGGCAGTTGTATTGAGTGTAGCACTTTGGTCCTATC
CTTACACTCAGCCTAGCGACTTTCTCGAG

Amino acid sequence of ***Tm*****Pgb-PIP-G7-5448**:

MPQIPGYTYGDPALPPGPVSLEELERLKASLLWTEEDDKALEQAGKVLEDQVEEVLDLWYGWV
GGEPHLLHYFTDPQANPIPDYLERVRRRFGQWILDTCFRPKDETWLRYQHEIGLRHHRTKKNQT
DGVTSVPHIPLRYNISFIYPTTATIKPFLTKKGHNPEEVERMHQAWFKAVVLSVALWSYPYTQPS
DFLE

Part 2: GDM-NT variants from recycled scaffold design

DNA acid sequence of **GDM-NT-121-5449**

CCTCCGCTTGTGGAGCCAATTGAAGATCCCGCAGAACGTGCAGCAATCTTGGCGGAATTGAA
AGCTTTCTTGCGGTTCCTGGCTGCTCTGAGTCGTGAGGAACTGGAACAGGTCACGAAGCGGC

GCGCTGGGGGTACATTGGATGAACTTCACGCACTTCTCCGTGAATTGGCAGCCGCCGACTTG
GAACTCCTTCTTGCTTTACGGGTGAGAGCAACCCGTGCCGATATAAACGCGTTTGCTAATGC
TTCGGACGAAGAGTTAGCGAAACTTGAAGCAATCCTGGCGGAAGAAATTGACAGATTAGTC
GCTCGTATGGAAGAAGCCCTTCCAGGCCTGGATTATGACGCAATTAGAGCCGAAGTCCTGGC
GGCTGTGAAGGAAGCGCTGGCTGCGCTGAAAGAAGGTGGTCTGGATGCATTACTGGATTAC
GTTTATCGAAGACTGGGAGAATTAGGGGCCAGAGTTTTGAGCTTGGATGTGAAAAAACTGA
GAGAACTGCATCCGGATGCGCCGCTGGGTCCATTAGTGGCCGCTGCCAAGGGCATTACGATA
GTCGAGATGGAAATTCCCGAGGCACTTCTGCCACTTCTCGTACGTGAGTTTCGAGAAGGGTT
GCCGGCACTTGACCGTCGCTATGCAGATGAATTAACTCGCTCCCAGCTGGCTACTTTAGTGG
CACTCTATGCCTTTAGCCTGATTCTTATGGCCTTACTTGCGGCGAAACAAAAACCCGATGCCC
CCAAAGTGCGCGTGTTCATCGTGCACCTTACCTATGATGACGGGACACTCACTCTGTTGCTC
ACTCCGGCA

Amino acid sequence of **GDM-NT-121-5449:**

PPLVEPIEDPAERAAILAELKAFLRFLAALSREELEQVTKRRAGGTLDELHALLRELAAADLELLL
ALRVRATRADINAFANASDEELAKLEAILAEEIDRLVARMEEALPGLDYDAIRAEVLAAVKEAL
AALKEGGLDALLDYVYRRLGELGARVLSLDVKKLRELHPDAPLGPLVAAAKGITIVEMEIPEAL
LPLLVREFREGLPALDRRYADELTRSQLATLVALYAFSLILMALLAAKQKPDAPKVRVFIVHLTY
DDGTLTLLLTPA

DNA sequence of **GDM-NT-226-5450:**

CCACCTGTCCTGCGCCCAGTTACTGACCCGGCCCGTCGCGCCGAGTTGCACGCCGCGCTGCT
TGAATTACTGCGTCAGTTGGCCGAACTCAGTGATGAACAACTTGCGGCGTGCTTAAAACGCC
GTTACGGGCTGACACAAGATGAGGTTAAAGAATTGTTAAAAGAACTGGCAGCAGCGGATCT
TCGCCTGCTGAACGCACTTGAAGTACATGCTACGAGAGCGGATATCGATGCTGCCCGCGATT
GGTCGGATGAAGAGTGGGCACGCCTGCGGGCGGTTTTTCGCGCGTTCCTGCGCGAACTGGTC
GCCCGGCTTGCTGCCACAGTTCCGGGTTTAGACGGAGAAGCGCTCTTGGCACGTCTGGAAGC
GGCGCTGGATGAGGCATTAGCCGCTCTGCGGGCAGGAGGTTTTGATGCTCTGCTGGATACAG
TAGCGCGTCAGCTCGAAGAGGCAGGTATTCCCTTGGTGGTATTTGATCTCCGCCGACTTGCC
GCTTTGCCAGGAGTCCCACCCCTTCCGGCTGTTCTGGCCGGCGCTCGAGGCCTGGCGATCGC
TGTTCTGGATGTCCCCCCTGCGGTGGCAGATTTATTCGCAGAGGTTTTATCCCGCGACATTCC
AGCATTCCAAGCACGTGTAGCCCCTGAACTTACCGCTTCTCAGATGGCTGTCTTGGCGGCAC
TTGCAACTGCCGCGTTGCTGCTTGCTGCTGCGTTCGTACGTGAAGCGCGACCGGATGCGCCC
ACAGTTCGCTTGGTCGTCGCGCACTTGACCTTTCCGGATGGCAACCTGGTTTTAGAGTTTCGT
GTAGAG

Pairwise identity (compared to **GDM-NT-121-5450**): 45.41%

Amino acid sequence of **GDM-NT-226-5450:**

PPVLRPVTDPARRAELHAALLELLRQLAELSDEQLAACLKRRYGLTQDEVKELLKELAAADLRL
LNALEVHATRADIDAARDWSDEEWARLRAVFRAFLRELVARLAATVPGLDGEALLARLEAALD
EALAALRAGGFDALLDTVARQLEEAGIPLVVFDLRRLAALPGVPPLPAVLAGARGLAIAVLDVPP
AVADLFAEVLSRDIPAFQARVAPELTASQMAVLAALATAALLLAAAFVREARPDAPTVRLVVA
HLTFPDGNLVLEFRVE

DNA acid sequence of **GDM-NT-151-5451**

AAGACTATAACACTGGAATCGGAATCCAGCCTGCCTTTTTCAGTACTCGAGTTGCTGTTGTC
ACCTGAGAATCTGGCCCGTCTTCTGGCGACATGGAAAAGTGAACACATAAGAATAGAAAGT
ATCGAAATACTGGAGTTTGTACCTGGTGAGCGCGTCACGGTCAAAGTTAAAGTGACTTTTAA
AGTGGCAGGTGAAGAAGTTCATCTTGAAAGCGTGGTAACGTTCGAACGCGTCGAGGGCGAT
CGTGCTCGTATCACCGCCGTCATTACCTACGATGAAGACAAGTTTCGTGAGGCACTCGAAAA

ACTTAGCGATGAAGAAATCAAAGACTGGATGTTAGCCGTTGCGAAGGAAGTTGCGGCAGCT
GTCAGAGAGTTGCTTGAGGAATTACGCGAACGAGGAGAAATTGATCTTAGTGACGAAGAAA
TCGATCGCCTCGTCGTTGCGGTCGGTCTGAAACATATGGGTGTGTTAGCTAAGTTAATCGAA
GCGGTTACCGGGAAAGACTACTCTGAACTGCTGGAGGAAGTTAAAAAACGTTTCCTGGAGG
AATGGTTG

Amino acid sequence of **GDM-NT-151-5451:**

KTITLESESSLPFSVLELLLSPENLARLLATWKSEHIRIESIEILEFVPGERVTVKVKVTFKVAGEEV
HLESVVTFERVEGDRARITAVITYDEDKFREALEKLSDEEIKDWMLAVAKEVAAAVRELLEELR
ERGEIDLSDEEIDRLVVAVGLKHMGVLAKLIEAVTGKDYSELLEEVKKRFLEEWL

DNA sequence of **GDM-NT-362-5452:**

GAAACGCTGGAGTTGGAAAGTGAAGCATCACTGCCCCTCGACGTGCTGGAGTTACTGTTATC
CCCTGAGAATCTTCCTCGTGTGTTGGCAGTGGGCCGTGGAGAACATTTCCGTCTGCTCAGTCT
TGAGGTTCTTGCGAGAGAACCGGGCCGTCGTGTAACGGTCGCTTCTCGCGCTGAAATAAGAC
TGGGTGATCGCTGGATCCCGGTAGATGTCGTGCTGACATTTGAGCGACAGCCGGATGGCAGC
GTGCGCATTAGAGCCGTACTGACATTCGACGCCGACGCCGTGCGAGAGGCATTGGCGGACA
TGACGGATGAAGAAATTGAGGATCTTTTGTTAGGTTTTGCCTTAGAGGTTGCTGCAGCGGTG
AGAGCACTTCTGGAAGAATTGCGCGAAGCTGGACGTATAGATCTGTCTGACGAAGAAATTG
CCGCGCTGGCACTGCGAGTGGGTTTAAAACATATGCGTGTCCTGGCACGGATTTTAGAGAAA
GCGACTGGGCGTGACTTATCTGCGGTATTAGAACGCGCGGAGGAACGCCTCAAAAAAGAAC
TCGCG

Pairwise identity (compared to **GDM-NT-151-5451**): 50.6%

Amino acid sequence of **GDM-NT-362-5452:**

ETLELESEASLPLDVLELLLSPENLPRVLAVGRGEHFRLLSLEVLAREPGRRVTVASRAEIRLGDR
WIPVDVVLTFERQPDGSVRIRAVLTFDADAVREALADMTDEEIEDLLLGFALEVAAAVRALLEE
LREAGRIDLSDEEIAALALRVGLKHMRVLARILEKATGRDLSAVLERAEERLKKELA

Part 3: GDM-NT variants from directed evolution and additional protein engineering thereof.

DNA acid sequence of **GDM-NT-399-5453**

GAAACGCTGGAGTTGGAAAGTGAAGCATCACTGCCCCTCGACGTGCTGGAGTTACTGTTATC
CCCTGAGAATCTTCCTCGTGTGTTGGCAGTGGGCCGTGGAGAACATTTCCGTCTGCTCAGTCT
TGAGGTTCTTGCGAGAGAACCGGGCCGTCGTCTAACGGTCGCTTCTCGCGCTGAAATAAGAC
TGGGTGACCGCTGGGTCCCGGTAGATGTCGTGCTGACATTTGAGCGACAGCCGGATGGCAGC
GTGCGCATTAGAGCCGTACTGACATTCGACGCCGACGCCGTGCGAGGGGCATTGGCGGACA
TGACGGATGAAGAAATTGAGGATCTTTTGTTAGGTTTTGCCTTAGAGGTTGCTGCAGCGGTG
AGAGCACTTCTGGAAGAATTGCGCGAAGCTGGACGTATAGATCTGTCTGACGAAGAAATTG
CCGCGCTGGCACTGCGAGTGGGTTTAAAACTTATGCGTGTCCTGGCACGGATTTTGGAGAAA
GCGACTGGGCGTGACTTATCTGCGGTATTAGAACGCGCGGAGGAACGCCTCAAAAAAGAAC
TCGCG

Amino acid sequence of **GDM-NT-399-5453:**

ETLELESEASLPLDVLELLLSPENLPRVLAVGRGEHFRLLSLEVLAREPGRRLTVASRAEIRLGDR
WVPVDVVLTFERQPDGSVRIRAVLTFDADAVRGALADMTDEEIEDLLLGFALEVAAAVRALLEE
LREAGRIDLSDEEIAALALRVGLKLMRVLARILEKATGRDLSAVLERAEERLKKELA

DNA acid sequence of **GDM-NT-400-5454:**

GAAACGCTGGAGTTGGAAAGTGAAGCATCACTGCCCCTCGACGTGCTGGAGTTACTGTTATC
CCCTGAGAATCTTCCTCGTGTGTTGGCAGTGGGCCGTGGAGAACATTTCCGTCTGCTCAGTCT
TGAGGTTCTTGCGAGAGAACCGGGCCGTCGTGTAACGGTCGCTTCTCGCGCTGAAATAAGAC
TGGGTGATCGCTGGATCCCGGTAGATGTCGTGCTGACATTTGAGCGACAGCCGGATGGCAGC
GTGCGCATTAGAGCCGTACTGACATTCGACGCCGACGCCGTGCGAGAGGCATTGGCGGACA
TGACGGATGAAGAAATTGAGGATCTTTTGTTAGGTTTTGCCTTAGAGGTTGCTGCAGCGGTG
AGAGCACTTCTGGAAGAATTGCGCGAAGCTGGACGTATAGATCTGTCTGACGAAGAAATTG
CCGCGCTGGCACTGCGAGTGGGTTTAAAAATGTTGCGTGTCCTGGCACGGATTTTAGAGAAA
GCGACTGGGCGTGACTTATCTGCGGTATTAGAACGCGCGGAGGAACGCCTCAAAAAAGAAC
TCGCG

Amino acid sequence of **GDM-NT-400-5455:**

ETLELESEASLPLDVLELLLSPENLPRVLAVGRGEHFRLLSLEVLAREPGRRVTVASRAEIRLGDR
WIPVDVVLTFERQPDGSVRIRAVLTFDADAVREALADMTDEEIEDLLLGFALEVAAAVRALLEE
LREAGRIDLSDEEIAALALRVGLKMLRVLARILEKATGRDLSAVLERAEERLKKELA

DNA sequence of **GDM-NT-401-5455:**

GAAACGCTGGAGTTGGAAAGTGAAGCATCACTGCCCCTCGACGTGCTGGAGTTACTGTTATC
CCCTGAGAATCTTCCTCGTGTGTTGGCAGTGGGCCGTGGAGAACATTTCCGTCTGCTCAGTCT
TGAGGTTCTTGCGAGAGAACCGGGCCGTCGTCTTACGGTCGCTTCTCGCGCTGAAATAAGAC
TGGGTGATCGCTGGGTTCCGGTAGATGTCGTGCTGACATTTGAGCGACAGCCGGATGGCAGC
GTGCGCATTAGAGCCGTACTGACATTCGACGCCGACGCCGTGCGAGGTGCATTGGCGGACAT
GACGGATGAAGAAATTGAGGATCTTTTGTTAGGTTTTGCCTTAGAGGTTGCTGCAGCGGTGA
GAGCACTTCTGGAAGAATTGCGCGAAGCTGGACGTATAGATCTGTCTGACGAAGAAATTGC
CGCGCTGGCACTGCGAGTGGGTTTAAAAATGTTGCGTGTCCTGGCACGGATTTTAGAGAAAG
CGACTGGGCGTGACTTATCTGCGGTATTAGAACGCGCGGAGGAACGCCTCAAAAAAGAACT
CGCG

Amino acid sequence of **GDM-NT-401-5455:**

ETLELESEASLPLDVLELLLSPENLPRVLAVGRGEHFRLLSLEVLAREPGRRLTVASRAEIRLGDR
WVPVDVVLTFERQPDGSVRIRAVLTFDADAVRGALADMTDEEIEDLLLGFALEVAAAVRALLEE
LREAGRIDLSDEEIAALALRVGLKMLRVLARILEKATGRDLSAVLERAEERLKKELA

DNA acid sequence of **GDM-NT-402-5456:**

GAAACGCTGGAGTTGGAAAGTGAAGCATCACTGCCCCTCGACGTGCTGGAGTTACTGTTATC
CCCTGAGAATCTTCCTCGTGTGTTGGCAGTGGGCCGTGGAGAACATTTCCGTCTGCTCAGTCT
TGAGGTTCTTGCGAGAGAACCGGGCCGTCGTCTTACGGTCGCTTCTCGCGCTGAAATAAGAC
TGGGTGATCGCTGGGTTCCGGTAGATGTCGTGCTGACATTTGAGCGACAGCCGGATGGCAGC
GTGCGCATTAGAGCCGTACTGACATTCGACGCCGACGCCGTGCGAGGTGCATTGGCGGACAT
GACGGATGAAGAAATTGAGGATCTTTTGTTAGGTTTTGCCTTAGAGGTTGCTGCAGCGGTGA
GAGCACTTCTGGAAGAATTGCGCGAAGCTGGACGTATAGATCTGTCTGACGAAGAAATTGC
CGCGCTGGCACTGCGAGTGGGTTTAAAAATGGCACGTGTCCTGGCACGGATTTTAGAGAAA
GCGACTGGGCGTGACTTATCTGCGGTATTAGAACGCGCGGAGGAACGCCTCAAAAAAGAAC
TCGCG

Amino acid sequence of **GDM-NT-402-5456:**

ETLELESEASLPLDVLELLLSPENLPRVLAVGRGEHFRLLSLEVLAREPGRRLTVASRAEIRLGDR
WVPVDVVLTFERQPDGSVRIRAVLTFDADAVRGALADMTDEEIEDLLLGFALEVAAAVRALLEE
LREAGRIDLSDEEIAALALRVGLKMARVLARILEKATGRDLSAVLERAEERLKKELA

DNA acid sequence of **GDM-NT-403-5457:**

GAAACGCTGGAGTTGGAAAGTGAAGCATCACTGCCCCTCGACGTGCTGGAGTTACTGTTATC
CCCTGAGAATCTTCCTCGTGTGTTGGCAGTGGGCCGTGGAGAACATTTCCGTCTGCTCAGTCT
TGAGGTTCTTGCGAGAGAACCGGGCCGTCGTCTTACGGTCGCTTCTCGCGCTGAAATAAGAC
TGGGTGATCGCTGGGTTCCGGTAGATGTCGTGCTGACATTTGAGCGACAGCCGGATGGCAGC
GTGCGCATTAGAGCCGTACTGACATTCGACGCCGACGCCGTGCGAGGTGCATTGGCGGACAT
GACGGATGAAGAAATTGAGGATCTTTTGTTAGGTTTTGCCTTAGAGGTTGCTGCAGCGGTGA
GAGCACTTCTGGAAGAATTGCGCGAAGCTGGACGTATAGATCTGTCTGACGAAGAAATTGC
CGCGCTGGCACTGCGAGTGGGTTTAAAATTGGCACGTGTCCTGGCACGGATTTTAGAGAAAG
CGACTGGGCGTGACTTATCTGCGGTATTAGAACGCGCGGAGGAACGCCTCAAAAAAGAACT
CGCG

Amino acid sequence of **GDM-NT-403-5457:**

ETLELESEASLPLDVLELLLSPENLPRVLAVGRGEHFRLLSLEVLAREPGRRLTVASRAEIRLGDR
WVPVDVVLTFERQPDGSVRIRAVLTFDADAVRGALADMTDEEIEDLLLGFALEVAAAVRALLEE
LREAGRIDLSDEEIAALALRVGLKLARVLARILEKATGRDLSAVLERAEERLKKELA

DNA acid sequence of **GDM-NT-404-5458:**

GAAACGCTGGAGTTGGAAAGTGAAGCATCACTGCCCCTCGACGTGCTGGAGTTACTGTTATC
CCCTGAGAATCTTCCTCGTGTGTTGGCAGTGGGCCGTGGAGAACATTTCCGTCTGCTCAGTCT
TGAGGTTCTTGCGAGAGAACCGGGCCGTCGTCTTACGGTCGCTTCTCGCGCTGAAATAAGAC
TGGGTGATCGCTGGGTTCCGGTAGATGTCGTGCTGACATTTGAGCGACAGCCGGATGGCAGC
GTGCGCATTAGAGCCGTACTGACATTCGACGCCGACGCCGTGCGAGGTGCATTGGCGGACAT
GACGGATGAAGAAATTGAGGATCTTTTGTTAGGTTTTGCCTTAGAGGTTGCTGCAGCGGTGA
GAGCACTTCTGGAAGAATTGCGCGAAGCTGGACGTATAGATCTGTCTGACGAAGAAATTGC
CGCGCTGGCACTGCGAGTGGGTTTAAAACATATGCGTGTCCTGGCACGGATTTTAGAGAAAG
CGACTGGGCGTGACTTATCTGCGGTATTAGAACGCGCGGAGGAACGCCTCAAAAAAGAACT
CGCG

Amino acid sequence of **GDM-NT-404-5458:**

ETLELESEASLPLDVLELLLSPENLPRVLAVGRGEHFRLLSLEVLAREPGRRLTVASRAEIRLGDR
WVPVDVVLTFERQPDGSVRIRAVLTFDADAVRGALADMTDEEIEDLLLGFALEVAAAVRALLEE
LREAGRIDLSDEEIAALALRVGLKHMRVLARILEKATGRDLSAVLERAEERLKKELA

DNA acid sequence of **GDM-NT-405-5459:**

GAAACGCTGGAGTTGGAAAGTGAAGCATCACTGCCCCTCGACGTGCTGGAGTTACTGTTATC
CCCTGAGAATCTTCCTCGTGTGTTGGCAGTGGGCCGTGGAGAACATTTCCGTCTGCTCAGTCT
TGAGGTTCTTGCGAGAGAACCGGGCCGTCGTCTTACGGTCGCTTCTCGCGCTGAAATAAGAC
TGGGTGATCGCTGGGTTCCGGTAGATGTCGTGCTGACATTTGAGCGACAGCCGGATGGCAGC
GTGCGCATTAGAGCCGTACTGACATTCGACGCCGACGCCGTGCGAGGTGCATTGGCGGACAT
GACGGATGAAGAAATTGAGGATCTTTTGTTAGGTTTTGCCTTAGAGGTTGCTGCAGCGGTGA
GAGCACTTCTGGAAGAATTGCGCGAAGCTGGACGTATAGATCTGTCTGACGAAGAAATTGC
CGCGCTGGCACTGCGAGTGGGTTTAAAACATGCACGTGTCCTGGCACGGATTTTAGAGAAAG
CGACTGGGCGTGACTTATCTGCGGTATTAGAACGCGCGGAGGAACGCCTCAAAAAAGAACT
CGCG

Amino acid sequence of **GDM-NT-405-5459:**

ETLELESEASLPLDVLELLLSPENLPRVLAVGRGEHFRLLSLEVLAREPGRRLTVASRAEIRLGDR
WVPVDVVLTFERQPDGSVRIRAVLTFDADAVRGALADMTDEEIEDLLLGFALEVAAAVRALLEE
LREAGRIDLSDEEIAALALRVGLKHARVLARILEKATGRDLSAVLERAEERLKKELA

DNA acid sequence of **GDM-NT-406-5460:**

GAAACGCTGGAGTTGGAAAGTGAAGCATCACTGCCCCTCGACGTGCTGGAGTTACTGTTATC
CCCTGAGAATCTTCCTCGTGTGTTGGCAGTGGGCCGTGGAGAACATTTCCGTCTGCTCAGTCT
TGAGGTTCTTGCGAGAGAACCGGGCCGTCGTCTTACGGTCGCTTCTCGCGCTGAAATAAGAC
TGGGTGATCGCTGGGTTCCGGTAGATGTCGTGCTGACATTTGAGCGACAGCCGGATGGCAGC
GTGCGCATTAGAGCCGTACTGACATTCGACGCCGACGCCGTGCGAGGTGCATTGGCGGACAT
GACGGATGAAGAAATTGAGGATCTTTTGTTAGGTTTTGCCTTAGAGGTTGCTGCAGCGGTGA
GAGCACTTCTGGAAGAATTGCGCGAAGCTGGACGTATAGATCTGTCTGACGAAGAAATTGC
CGCGCTGGCACTGCGAGTGGGTTTAAAATTGTTGCGTGTCCTGGCACGGATTTTAGAGAAAG
CGACTGGGCGTGACTTATCTGCGGTATTAGAACGCGCGGAGGAACGCCTCAAAAAAGAACT
CGCG

Amino acid sequence of **GDM-NT-406-5460:**

ETLELESEASLPLDVLELLLSPENLPRVLAVGRGEHFRLLSLEVLAREPGRRLTVASRAEIRLGDR
WVPVDVVLTFERQPDGSVRIRAVLTFDADAVRGALADMTDEEIEDLLLGFALEVAAAVRALLEE
LREAGRIDLSDEEIAALALRVGLKLLRVLARILEKATGRDLSAVLERAEERLKKELA

DNA acid sequence of **GDM-NT-407-5461:**

GAAACGCTGGAGTTGGAAAGTGAAGCATCACTGCCCCTCGACGTGCTGGAGTTACTGTTATC
CCCTGAGAATCTTCCTCGTGTGTTGGCAGTGGGCCGTGGAGAACATTTCCGTCTGCTCAGTCT
TGAGGTTCTTGCGAGAGAACCGGGCCGTCGTCTTACGGTCGCTTCTCGCGCTGAAATAAGAC
TGGGTGATCGCTGGGTTCCGGTAGATGTCGTGCTGACATTTGAGCGACAGCCGGATGGCAGC
GTGCGCATTAGAGCCGTACTGACATTCGACGCCGACGCCGTGCGAGGTGCATTGGCGGACAT
GACGGATGAAGAAATTGAGGATCTTTTGTTAGGTTTTGCCTTAGAGGTTGCTGCAGCGGTGA
GAGCACTTCTGGAAGAATTGCGCGAAGCTGGACGTATAGATCTGTCTGACGAAGAAATTGC
CGCGCTGGCACTGCGAGTGGGTTTAAAAATGATGCGTGTCCTGGCACGGATTTTAGAGAAAG
CGACTGGGCGTGACTTATCTGCGGTATTAGAACGCGCGGAGGAACGCCTCAAAAAAGAACT
CGCG

Amino acid sequence of **GDM-NT-407-5461:**

ETLELESEASLPLDVLELLLSPENLPRVLAVGRGEHFRLLSLEVLAREPGRRLTVASRAEIRLGDR
WVPVDVVLTFERQPDGSVRIRAVLTFDADAVRGALADMTDEEIEDLLLGFALEVAAAVRALLEE
LREAGRIDLSDEEIAALALRVGLKMMRVLARILEKATGRDLSAVLERAEERLKKELA

Part 4: GDM-NT variants from the third round of “novel scaffold” design

DNA acid sequence of **GDM-NT-507-5509:**

AAAACCACATTTGAATTGCGGTATGAAGGTGGCGAGGTGGTCCTGTCCAGTAAAACCGAAG
TGGATGGGAAGGTGTTAGAAACACGTCGCCGTGGTCGGCCGGAAAACATTGCGTTTATTGCG
AGACGTCTGCTTGCTGCGCCTGATGATGAGCTGCTTGATGTGGCTCTGAGAGCGGCGATTGC
TATATTAATTGCTGATGCTATAGCAGATTACCTCAAACTCACGGAAGGCGAAGATGCAGTGC
CATCTTTGGAATTGCGTCTTAAACTGATCGAAATCCTGGCCGAGAGAATTAAATATGAGGTC
GAAAAAATTGAAGGCGGCTTGCGTGTTACTTTCGAAGTCGATCTGGAAGCACTGGCTAAAG
ATCCGTCCGTACCAGAAGAAGTTCGGGAGATCCTGAAAAAATTCAAAAAAGCTAGTGGCAA
AATCACAATTCACGTCACAGAAGCCGACCTGCGCGCATTATTGGCCGCCATTCGCGCGGCCC
TGGCGGCCCTGCCAGCGGACGCAACACCCGAGGAAATTCAAGCTGTCGTGCGCGCAGTGGC
CGGTGCATTTATTGCGGAACGGGGTCTCACTCGTCTTGCCGAGGGGGAAATCGAATTGGAGG
TCAAA

Amino acid sequence of **GDM-NT-507-5509:**

KTTFELRYEGGEVVLSSKTEVDGKVLETRRRGRPENIAFIARRLLAAPDDELLDVALRAAIAILIA
DAIADYLKLTEGEDAVPSLELRLKLIEILAERIKYEVEKIEGGLRVTFEVDLEALAKDPSVPEEVRE
ILKKFKKASGKITIHVTEADLRALLAAIRAALAALPADATPEEIQAVVRAVAGAFIAERGLTRLAE
GEIELEVK

DNA acid sequence of **GDM-NT-543-5510:**

TTAACTCCGGAGGAACTGTTAGCCCGCGCTCGAGAATTAGAAGCCAGAGCCCCACCTTTTAC
TGTACCTGAGGGGGTTTTAGCCGCCGCGACGGAAGTGCTGCGTGAAGCAGTCGCGAAGCTC
GCCGAAAAAGTGGGAGAAGAAAAAGCAAAAGAATTGGTAGAGCGTGTGTTATCGGATGAA
TATTATGCTCGTCTGACCAAGAAACGTCTGGAGATCCTGGCAGCCGACCCTTGGATCCGGGC
TGTTATGCAGGAAATTCTGGATCTGCTGGAAGAAGCCGTCGCCAAAGGGAAAAACCCTATG
GAAATCCTGTTAAAAGCAATTCTGGAAACGGTGCGCTACCTCATCGAAAAAGTGGGAGAAG
ATACTGTAATCGCCCTGACCCGAGAACGCGCAAAAGGTAAAGTTGTTGTTCAACGGATCGTT
CGCATTGACGGTAAAACTTATGTCATAGTCGCAAGCTTACCTTTGTCTGAAGAAACACTGGA
CAAGAGATTGCTGGGGCCAATTGTATTTGTCTTTGAAGTCCCTAACGAACTTGAAGAAGAGC
TGCGTGAGCTGCTTAAAGAAGATCCGTTTCGTTGTTTAGTTAAAGCGGTGCTGAACGCGAAA
GAGTTGTATCTGTTAGAGCCAGTCCGTGAAGATGGTGAATTAACCGTCCGCCTGAAACCACT
GCATCGCTTACTGCTCGCGATCGCCGTGATGTTAAAGGACATTCGTGACCGCCTCGGGGAGG
ACATAGCTGAGGAAGCCGCCAAAGTCCTGTTAGACACCTTAAAGCGTGAGGTTGCCGCA

Amino acid sequence of **GDM-NT-543-5510:**

LTPEELLARARELEARAPPFTVPEGVLAAATEVLREAVAKLAEKVGEEKAKELVERVLSDEYYA
RLTKKRLEILAADPWIRAVMQEILDLLEEAVAKGKNPMEILLKAILETVRYLIEKVGEDTVIALTR
ERAKGKVVVQRIVRIDGKTYVIVASLPLSEETLDKRLLGPIVFVFEVPNELEEELRELLKEDPFRCL
VKAVLNAKELYLLEPVREDGELTVRLKPLHRLLLAIAVMLKDIRDRLGEDIAEEAAKVLLDTLK
REVAA

DNA acid sequence of **GDM-NT-589-5511:**

TCACGAGAAGAGATTCGTGAGGCACTGTTAGCAGCGATTGAGGAAGTTCGCGCAGACGAAG
AAGTTCGTGCAGCGCAAGCGGAGGCGGCAGCCATTGCTGACGAAGCAGCGGAAAAAGTGGC
TAGCCCAGCAGTGGCTGCAACCCTGCGCCAGCTGGCAGAGCTCATCCGTCAGCATCCCTTAG
CCCTCGCTACCCTGGAAGTTGCGCTGGGGAAGACGGAACGCGGCCGACGTCTGCGCGCACT
GTTGGTTGCAGCGGCAGAGGCCGCGGCTGGCTTGACAGAAGAAGAACGTGCGGTCTTACGG
CGCGCGTTGGAGCTTGCGGAGCGCATGCGCGATCTGGTACTGAAAAAAGAAATAAACAGTC
CCGAAGAACTGAAAGCGACTGTCGCCGAAATTCGCCGTCTTCTTGCCGAAGCCGAGCCGCTG
ATCGCCGAAGCCGAGGAAATAGCGCGCCGAAAAGGCATTAAAGTTCCGGAACCAGATGCCG
CAGTGGTTGCTGCTGCAACCCCAGAAGATGCCTTTGTAATAGGAAAAGCCGTAGCCAAGATT
GCAAAAAAATTTGGTTTGAAAACGATGTTCATGAAAATTGCAGAATTCGTTCTGGAAGGCAA
ACTGTCTCGTGAAGAGGCACTGGAAGCCTTGGGCGTAGCAGTTAGAGCGCTGCGGGCCGTG
CTTCGCGTGCGTGCGATAGTTCTGGCCATGGCCGCGCAGCTGGTTCCCGAATTACAGGTGAC
CGTGGAATTTCGTCGCCTGCTGTTGACCCTCGCAGAGATCGTAGCGCGCTTCCCGGAGGCAT
TTCTGGAAGGAATAATCGAAGCTTTAAAGGAAGGT

Amino acid sequence of **GDM-NT-589-5511:**

SREEIREALLAAIEEVRADEEVRAAQAEAAAIADEAAEKVASPAVAATLRQLAELIRQHPLALAT
LEVALGKTERGRRLRALLVAAAEAAAGLTEEERAVLRRALELAERMRDLVLKKEINSPEELKAT
VAEIRRLLAEAEPLIAEAEEIARRKGIKVPEPDAAVVAAATPEDAFVIGKAVAKIAKKFGLKTMF
MKIAEFVLEGKLSREEALEALGVAVRALRAVLRVRAIVLAMAAQLVPELQVTVEFRRLLLTLAEI
VARFPEAFLEGIIEALKEG

Part 5: GDM-NT variants used as negative control for structural analysis

DNA sequence of **GDM-NT-105:**

ACTCCAGAGGAACTTGTTGCTTTTATGTTGGATCCAGATGCTGACGTTGATGAAGCACTGGC
TAGACTCGAAGAATTTCTTGCTACTGCCACGCCTGAAGAGTTGCTTGAGCTTGCACGTGTAG
TGTTTGCTGCGGTTGTCGAACGATTCCCTCGGATAATGGCATTACACGCTCATCTGCGAGAA
CATGGAGGGCGCCCTCCGCCGCCGGCGGGCTTTTATTTTGCCATGGGTGTGATGACGCTGCT
GGGAGATAACGCGCGTCGGCAGTTACAGCGTAAATATGGTCTGTCAGAAGAAGAAGGGGCG
CTGTTGTTTCTTGACGCCGTTGACCGCGTTATACCTGAGCTTGTGAGAGCCCTGTTAGGACGT
GAACCGGATCCTCGCCGTCGCCGGTTAACCATGCTTTTCGTTTTGTATCTTGAATATCCAGAA
CTGTTTAAAAAATTATTCACAGAAGAAGAGCAGGCCTTCGTAAGACGTTTATTTCGCCGCGT
TCTGGCTGAAGGTGTTGAGGCCGCATTGGCCGCCTTGACACCGGAAGAACGCGCAAGATTG
GATGCCATGTACGCTAAATTTCTGGCGGCAGCTGAGGAGCTCAAGAAGAAATATCCGGAAG
CCGCTGCAGAGATGGAAGCCCTCGCAGCCCGGCTGCGAGAGCTGGCGCTTCGGATCAATGC
CCGGGTGGCTTCCCTGTCTGAAGCAGACCAGGCGGCACTGCAGGCAGCGGTTGATGCCGCAT
TCGCCGAAGCGGCTGCTCGTACGCCGCTTGCTGATCTTTTGGCG

Amino acid sequence of **GDM-NT-105:**

TPEELVAFMLDPDADVDEALARLEEFLATATPEELLELARVVFAAVVERFPRIMALHAHLREHG
GRPPPPAGFYFAMGVMTLLGDNARRQLQRKYGLSEEEGALLFLDAVDRVIPELVRALLGREPDP
RRRRLTMLFVLYLEYPELFKKLFTEEEQAFVRRLFRRVLAEGVEAALAALTPEERARLDAMYAK
FLAAAEELKKKYPEAAAEMEALAARLRELALRINARVASLSEADQAALQAAVDAAFAEAAART
PLADLLA

DNA sequence of **GDM-NT-141:**

GGGGACGAGGTTAAAGCTGCCCTGATCCGTGCTGCCGAAGCAGTATATGCGGGTGATCGTG
AGGGTGCGCGCGCTGCCCTGGCGGAGGCAGTTGACGCCCTGGAGAAGAGATCCCGGGAACA
AGTGAGACTGGCAATCAAAGGTCTGCTCGAAATAGCAAAATTTGTAGCAGAACTGCTCAAG
GAGTTAACCCCAGAACAAAAAGAAGCGCTGCTGGCGCAGGGCGTCCTTGAGGCAATTAAAC
GTTTTTTCCTTGCCCTTGCTGCCATAGCAGGTCTGGTGGGCGGTGACGAAATGGTTGATGAA
GTGCTTGAAATGGGAAAAGAGGTCGCGGAACTTTTAAAGGAGGTTGTTGGGGAGGAAAAAG
CGGACGAATTTAAGGAAGAAGTCGAAAAAGGAGTTGCTGAACTTCGGACACTTACTCCAGA
AGAACGCAAAGAAAGAGCCGTTGATCTCCTGCTGGAAGTGTTAGAAGCGATGGTCGCCCGG
GGTCTTAGCCTTGACCAGATTAAAGAGTTTGCGCGTTTGTTGGCAGAACTGCTGACAGCTGA
AACCGAAGAAGAAGTAAAAGCAGTTGTCGAAAAAGTGAAAGCAATCCACAAAGCGAGTGT
TGCACGTCTCAAGGCA

Amino acid sequence of **GDM-NT-141:**

GDEVKAALIRAAEAVYAGDREGARAALAEAVDALEKRSREQVRLAIKGLLEIAKFVAELLKELT
PEQKEALLAQGVLEAIKRFFLALAAIAGLVGGDEMVDEVLEMGKEVAELLKEVVGEEKADEFK
EEVEKGVAELRTLTPEERKERAVDLLLEVLEAMVARGLSLDQIKEFARLLAELLTAETEEEVKAV
VEKVKAIHKASVARLKA

Table S9. Summary of mutations introduced in each round of evolution for ***Tm*Pgb-PIP** variants:

All mutations are compared to *Tm*Pgb-wt. New mutations are highlighted in red.

Entry	Variant	Mutations from ***Pa*Pgb-wt**
1	***Tm*Pgb-PIP-G1-5442**	I148T
2	***Tm*Pgb-PIP-G2-5443**	S17G, L141S, I148T, Y170H
3	***Tm*Pgb-PIP-G3-5444**	S17G, C30S, L141S, I148T, Y170H

4	***Tm*Pgb-PIP-G4-5445**	S17G, C30S, F62W, L141S, I148T, Y170H
5	***Tm*Pgb-PIP-G5-5446**	S17G, C30S, F62W, S141N, I148T, Y170H
6	***Tm*Pgb-PIP-G6-5447**	S17G, C30S, F62W, H66E, S141N, I148T, Y170H
7	***Tm*Pgb-PIP-G7-5448**	S17G, C30S, F62W, S65G, H66E, G78A, S141N, I148T, Y170H, Q180S, G192S

Table S10. Summary of mutations introduced in the final round of engineering for **GDM-NT** variants:

Entry	Variant	Mutations from **GDM-NT-362-5452**
1	**GDM-NT-399-5453**	V53L, I68V, E99G, H155L
2	**GDM-NT-400-5454**	H155M-M156L
3	**GDM-NT-401-5455**	V53L-I68V-E99G-H155M-M156L
4	**GDM-NT-402-5456**	V53L-I68V-E99G-H155M-M156A
5	**GDM-NT-403-5457**	V53L-I68V-E99G-H155L-M156A
6	**GDM-NT-404-5458**	V53L-I68V-E99G
7	**GDM-NT-405-5459**	V53L-I68V-E99G-M156A
8	**GDM-NT-406-5460**	V53L-I68V-E99G-H155L-M156L
9	**GDM-NT-407-5461**	V53L-I68V-E99G-H155M

Design Pipeline Details

Initial Design Details (First Round)

The initial designs were made based on AlphaFold 3 (AF3) predictions of ***Tm*Pgb** and ***Ap*Pgb**. For details of this round of design, see descriptions in our report on AlphaProtein Novo (*6*).

Partial Diffusion Details (Second Round)

We performed partial diffusion from "parent" designs **GDM-NT-121** and **GDM-NT-151**, which are described in companion work (*6*). For each "parent" design, we generated two groups of designs by applying enough renoising steps to ensure the post-denoising median TM-score distributions centered around 0.95 or 0.99. **GDM-NT-226** and **GDM-NT-362** were created by this process, derived from **GDM-NT-121** and **GDM-NT-151** respectively.

Metric Definitions

We used AF3 (*7*) for structure prediction. Metric definitions are as follows:

Subsets

- `sampled/` refers to metrics on the sampled structure
- `sequence/` refers to metrics on the sequence (from LigandMPNN)
- `monomer/` refers to metrics on a monomer (apo) fold of the design
- `complex/` refers to metrics on a complex (protein with ligands from the motif) fold of the design
- `complex/expanded_motif/` refers to motif metrics where the entire residue of a motif (in this case the heme-ligating histidine) in included, versus motif metrics which only involve the atoms fixed in the motif

Sample Metrics

- percent_ligand_bb_clashes: Percent of ligand atoms within 2.0 Å of a protein backbone atom
- max_consecutive_identical_aatypes: Max consecutive identical residues
- max_single_aatype_fraction: Max fraction of any single amino acid type
- num_blocklisted_motifs: Number of subsequences matching anything from the following sequences.
  - WSHPQFEK (Strep-tag)
  - EQKLISEEDL (c-Myc-tag)
  - ENLYFQ[SGAMCH] (TEV protease)
  - LEVLFQGP (3C protease cleavage site)
  - YPYDVPDYA (HA-tag)
  - HHHHH (His-tag)
  - DYKDDDDK (FLAG-tag)
  - GKPIPNPLLGLDST (V5-tag)
  - LVPRGS (Thrombin cleavage)
- unknown_count: Number of unknown ('X') amino acid type
- sample_his_rmsd: the RMSD of the ligating His sidechain atoms from the ideal pentagonal plane

Consistency Metrics

- tm_score: Template Modelling score (*8*) measuring length-normalised global structural similarity.
- interface_lddt: LDDT (*9*) computed between residues or atoms not on the same chain.
- mean_pocket_bb_aligned_ligand_rmsd: Mean RMSD of ligand heavy atoms after superimposing the predicted structure onto the reference on backbone atoms within 10 Å of the ligand.
- mean_pocket_bb_rmsd: Mean RMSD of pocket backbone atoms as defined above.

- motif_allatom_rmsd: All-atom RMSD between AF3 prediction and input motif structure
- motif_with_ligand_allatom_rmsd: same as above, with inclusion of the ligand motif atoms

Confidence Metrics

- predicted_distance_error: AF3 predicted distance error over all atoms
- chain_pair_pde_mean_sym: Symmetrised mean predicted distance error between chains.
- ligand_plddt_mean: Mean ligand pLDDT.

Structure Prediction Metrics

- pocket_C9_min_atom_dist: the minimum distance between any atom of the designed protein to the tail carbon atom (‘C9‘) of the substrate’s phenyl group, based on structure prediction of the sample with the substrate and heme cofactor
- pocket_C9_allatom_neighbors_mean: the number of design atoms within 4, 6, or 8 Å, averaged across the different distance thresholds, of the substrate’s ‘C9‘ atom, based on structure prediction with the substrate
- propionate_hbond_num_atoms: the number of potential H-bond partner atoms to any of the heme propionate’s oxygens (O1A, O2A, O1D, O2D), based on the structure prediction of the protein in complex with the heme and substrate
- propionate_hbond_num_res: the number of unique residues corresponding to the above

We also included metrics that quantify the variance of two relevant ligand atoms across structure prediction with five different representations of the small molecules and states involved: heme and substrate, heme and piperidine, heme and pyrrolidine, and the heme covalently bound to the substrate with two different SMILES representations. The SMILES representations are:

- CC1=C(CCC(O)=O)C2=NC1=Cc1c(C=C)c(C)c3\C=C4/N=C(/C=C5\N([Fe](N(CCCCCC6=CC=CC=C6)N=N)n13)\C(=C/2)C(CCC(O)=O)=C5C)C(C)=C4C=C
- [Fe]N(CCCCCC1=CC=CC=C1)N=N.CC1=C(CCC([O-])=O)/C2=C/C3=N/C(=C\C4=C(C)C(C=C)=C([N-]4)/C=C4\N=C(\C=C\1/[N-]\2)C(C=C)=C4C)/C(C)=C3CCC([O-])=O.

And the resulting metrics are:

- fe_self_rmsd: the RMSD of the iron atom in the porphyrin across the five different structure predictions, after global alignment to the sampled structure
- n_self_rmsd: same as above, but for the ligating nitrogen of the substrate

Finally, we specified a metric for regioselectivity based on the Hydrogen Atom Transfer step of the reaction, where the near-attack conformation to the hydrogen of the fourth vs fifth carbon in the alkyl chain suggests regioselectivity for pyrrolidine and piperidine, respectively. Because AF3 does not generate hydrogens, we instead simply use the distance to the corresponding carbon as a proxy metric.

- piperidine_propensity: the difference in distance between nitrogen and the fifth carbon away from the substrate from structure prediction, and the distance between the nitrogen and the fourth carbon. We take the minimum value over 7 structure prediction seeds of the design in complex with “heme and substrate” (as described above) for filtering. Note that AF3 Leaving Atom (AF3-LA) (*6*) was used for computing this metric. All other metrics were computed with AF3.

Filtering Process

After denoising and resequencing with LigandMPNN (*10*), the samples were first filtered by:
(*also applied in multi-resequence filtering)*

- sampled/percent_ligand_bb_clashes <= 5.0
- sequence/max_consecutive_identical_aatypes = 5
- sequence/max_single_aatype_fraction <= 0.25

- sequence/num_blocklisted_motifs <= 0
- sequence/unknown_count <= 0
- monomer/predicted_distance_error <= 2.0
- monomer/tm_score >= 80
- complex/chain_pair_pde_mean_sym <= 2.0
- complex/interface_lddt >= 30
- complex/mean_pocket_bb_aligned_ligand_rmsd <= 2.5
- complex/mean_pocket_bb_rmsd <= 2.0
- complex/expanded_motif/motif_allatom_rmsd <= 1.5
- complex/expanded_motif/motif_with_ligand_allatom_rmsd <= 2.5
- complex/ligand_plddt_mean >= 75

(*not applied in multi-resequence filtering)*

- pocket_C9_min_atom_dist between 2.8 and 3.8
- pocket_C9_allatom_neighbors_mean between 38 and 62, inclusive
- propionate_hbond_num_atoms between 3 and 6, inclusive
- propionate_hbond_num_res between 3 and 5, inclusive
- sampled_his_rmsd <= 0.08
- ligating_n_self_rmsd <= 0.8
- his_heme_distance <= 3.0

Additional criteria for a subset of samples:

- The piperidine_propensity filter as predicted by AF3-LA was applied at <= 0.35.
- Samples were subjected to a "multi-resequence" filter. For each candidate (comprising a backbone and its "original" LigandMPNN sequence passing the full set of filters described above), we generated 7 "additional" LigandMPNN sequences. A candidate passed the multi-resequence filter if all of these additional 7 re-sequences met the criteria labeled "Also applied in multi-resequence filtering." These 7 additional re-sequences were only used for filtering: we use the original LigandMPNN sequence for experimental characterisation.

Selection Process

In total, 96 total partial diffusion designs were tested (48 derived from **GDM-NT-121** and 48 derived from **GDM-NT-151**). Within each 48-design set, half (24) had a post-denoising median TM-score of 0.95, and the other half had a median TM-score of 0.99. These subgroups were further divided evenly based on previously described selection criteria: 12 designs passed only the base filters, while the remaining 12 passed both the base and additional filters.

Designs in the 0.99 TM-score group were chosen at random. Conversely, designs in the 0.95 TM-score group were selected *via* K-medoids clustering by TM-score to identify representative centroids for each design facet. To minimize the contribution of the termini during clustering, we trimmed the sequences before determining the TM-score by removing the first 4 and last 2 residues from **GDM-NT-121** designs, and the first 3 and last 1 residues from **GDM-NT-151** designs.

Structural Diversity of Designs

Design Similarity to PDB Chains

Heme-containing structures were retrieved from a March 2026 PDB (*11*) snapshot. Heme cofactors were restricted to the following CCD codes representing iron-porphyrin variants: HEM, HEC, HEA, HEB, HEO, HDD, SRM, HCO, 1FH, 2FH. We pre-filtered to PDB entries containing at least one non-polymer entity, then identified heme-containing entries by parsing ligand residue names, yielding 7,543 heme-containing structures. We further restricted this analysis to PDB chains that are ligated to a heme, using proximity to the Fe atom of each heme to disambiguate multiple chains. Sequences were then clustered with MMseqs2 (*12*) (easy-cluster, 70% identity, 80% bidirectional coverage, greedy set-cover mode), yielding 1,016 non-redundant chains.

We computed all all-vs-all structural alignments using TM-align on the representative single-chain structures plus the two partial diffusion designs (**GDM-NT-226** and **GDM-NT-362**) to obtain TM-scores. The pairwise distance between structures was defined as 1 minus the mean TM-score normalised to both chain lengths.

Separately, each design was aligned against all heme-proximal PDB chains (before clustering) using TM-align to identify the closest structural match without sequence-identity reduction. The resulting TM-align superposition for this top-scoring hit is shown in Figure 3b.

Visualizations

Each heme-binding chain was assigned to a functional family by keyword matching against the concatenated PDB entry description and title fields, applied in the following priority order: P450 ("p450" or CYP followed by a digit or word boundary); Globin ("globin", "hemoglobin", or "myoglobin"); Peroxidase ("catalase-peroxidase", "katg", or "peroxidase"); Peroxygenase ("peroxygenase" or "UPO"); Catalase ("catalase", excluding any catalase-peroxidases as they are classified under Peroxidase); Dioxygenase ("dioxygenase", "TDO", or "IDO"/"IDO1"/"IDO2"); Heme oxygenase ("heme oxygenase" or "haem oxygenase"); Nitric oxide synthase ("nitric oxide synthase"); Cytochrome c ("cytochrome c" without co-occurring "oxidase", "p450", or "peroxidase"; note this results in a heterogeneous group which includes single heme cytochrome c's as well as multi heme proteins); Siroheme (SRM CCD code or "siroheme"). Remaining entries were labelled "Other heme proteins".

Metric MDS was applied to the precomputed distance matrix using scikit-learn (*13*) (n_components=2, dissimilarity='precomputed', metric=True, init='classical_mds', n_init=1, max_iter=10000, random_state=42). Kruskal's stress-1 was 0.35, indicating moderate distortion in the two-dimensional projection. The most structurally similar PDB chains for each design are highlighted in Fig. 3b.

UMAP (*14*) was applied to the same distance matrix (n_components=2, metric=precomputed, n_neighbors=30, min_dist=0.3, random_state=42). Parameters were selected from a sweep spanning local (n_neighbors=5, min_dist=0.05) to global (n_neighbors=50, min_dist=0.5) regimes; the chosen values best preserved global cluster topology while maintaining interpretable inter-family separations.

In both plots, PDB representatives are coloured by the protein family, and de novo designs are overlaid as gold stars.

UMAP projection based on all-pairs TM-align scores across non-redundant heme-binding chains

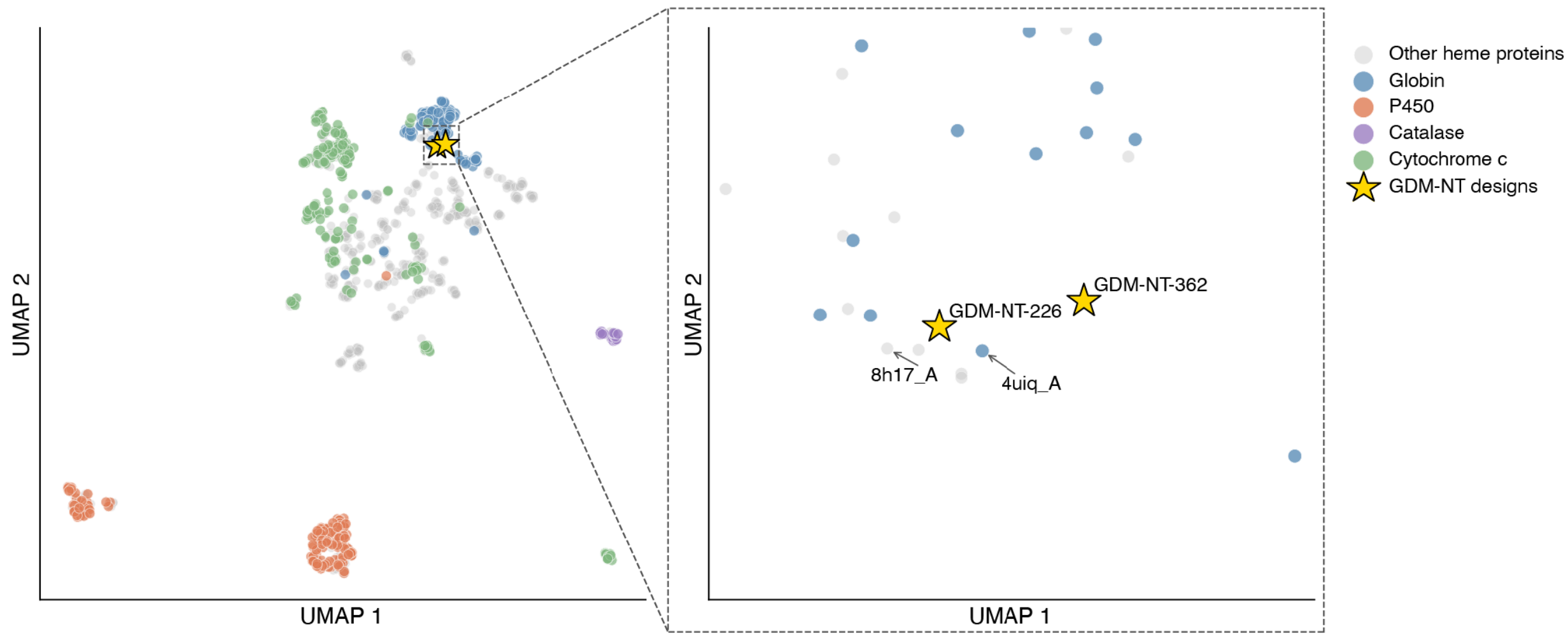

Figure. S1 UMAP projection based on all-pairs TM-align scores across non-redundant heme-binding chains from the PDB (March 2026), clustered at 70% sequence identity (n=1016). Each point represents a cluster representative, colored by family, with GDM-NT designs projected into the same space. Note: the cytochrome c label is a heterogeneous grouping defined by PDB annotation. The inset (right) zooms into the region surrounding the two designs, with the most structurally similar PDB chain for each design labelled (8h17_A for **GDM-NT-226**; 4uiq_A for **GDM-NT-362**). Structural superpositions of these closest matches are shown in Figure 3b. Designs occupy a sparsely populated region of the landscape

Motif-Informed Design Details (Third Round)

With the successful designs obtained from partial diffusion and post-design evolution, we sought to test whether these successful designs could in return inform the design pipeline. Specifically, we anticipated extracting motifs from successful designs after the second round could lead to higher success rate and better scaffold diversity. Guided by this principle, the third design round explored alternative noncanonical residue options at the proximal axial position while otherwise keeping the design approach consistent with the second design round, with exceptions in motif generation and metrics, as described below.

Motif Generation
We extracted haem-bound piperidine poses and their corresponding proximal axial residues from AF3-LA predictions of **GDM-NT-362**, **GDM-NT-401**, **GDM-NT-406**. In contrast to earlier rounds, we used AF3-LA (instead of AF3), and we did not enforce a covalent bond between the product and the iron on the HEM ligand. Previously, this extra covalent bond was used to ensure coordination between HEM and the product; we found it unnecessary for this round.

Metrics
Because this design round incorporates axial ligands other than histidine, we generalized several metrics to accommodate the geometries introduced by these alternative axial ligands.

1) Axial Heme Distance:
This metric measures the distance between the "tip atom" of the residue at the proximal axial position and the iron on the HEM ligand. We defined the following tip atoms for each axial ligand:

- **NE2** for Histidine (**GDM-NT-362**)
- **SD** for Methionine (**GDM-NT-401**)
- **CD1** for Leucine (**GDM-NT-406**)

For **GDM-NT-362**, AF3-LA predicts an axial heme distance of 2.1 Å. The corresponding threshold used in the second round of nitrene transfer design was 3.0 Å. For motifs derived from **GDM-NT-401** and **GDM-NT-406**, we established thresholds that maintain the same ratio between the threshold and the mean axial heme distance across 10 folding seeds using AF3-LA.

The maximum thresholds used per motif were:

- **GDM-NT-362**: ≤ 3.0 Å
- **GDM-NT-401**: ≤ 4.0 Å
- **GDM-NT-406**: ≤ 4.8 Å

2) Sampled Axial Residue RMSD:
In previous rounds, the axial residue RMSD metric took the residue corresponding to the axial ligand from the generator sample, aligned it to an idealized geometry of that same side-chain, and computed the RMSD.

The goal of this metric is to evaluate the validity of the terminal side-chain frame of the sampled residue. For histidine, the entire side-chain is rigid and can be represented by a single frame, but this implementation had to be adapted for the more flexible leucine and methionine side-chains.

To generalize this metric, we recalibrated the RMSD for histidine into a threshold based on the maximum absolute bond length error (compared to CCD idealised coordinates). An RMSD threshold of 0.08 was previously used for the axial residue RMSD. By regressing the maximum absolute bond length error onto the RMSD for histidine ($R^2 = 0.97$), we derived a maximum absolute bond length error cutoff of 0.14 Å.

Additionally, we introduced a maximum absolute bond angle error cutoff of 20°. We observed that the generator would sometimes produce samples, particularly for leucine, with acceptable bond lengths but severely distorted bond angles. This loose 20° cutoff was applied across all motifs to filter out such anomalies.

3) Multi-resequencing:
In the third round of design, we exclusively selected designs passing 7/7 multi-resequences. This is in contrast to the novel-scaffold facet of the second round of design described in AP Novo (*6*), which selected designs passing at least 4/7 multi-resequences.

Scaffold Novelty of the Third Round of Design

The three top-performing designs **GDM-NT-507**, **GDM-NT-543**, **GDM-NT-589** all feature low TM-scores compared to known natural haemproteins. Due to limited space, we show the TM-align superposition of **GDM-NT-543** (blue) with its closest structural match (gray) in the following figure.

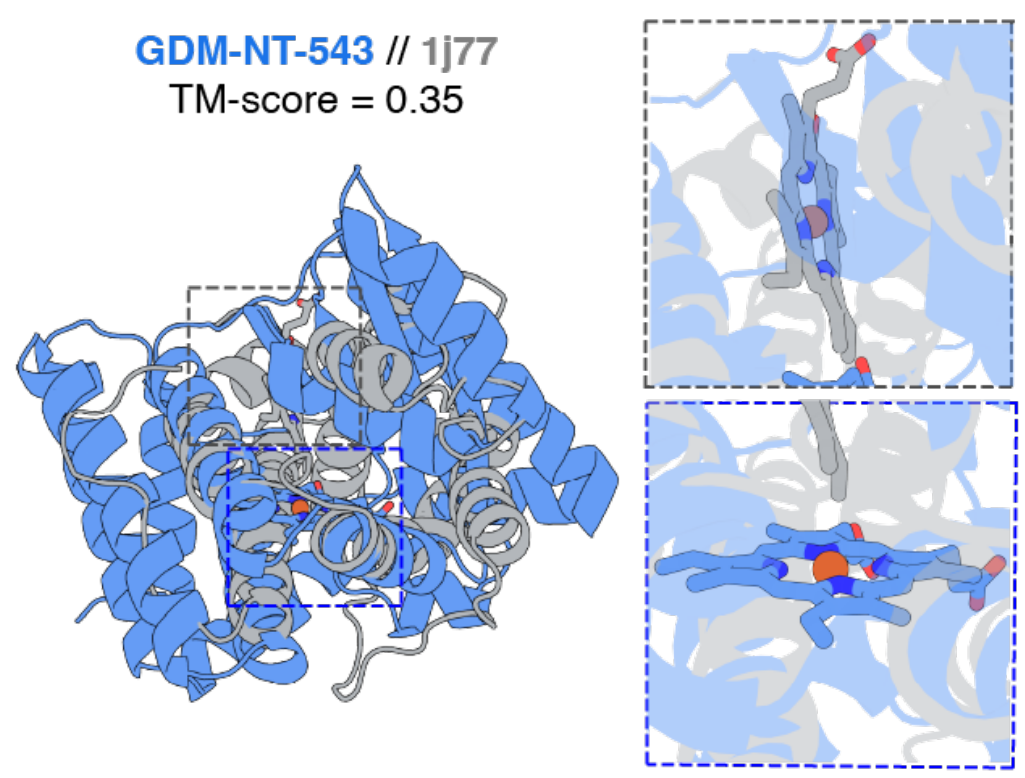

Figure S2. Structural diversity of additional active designs. TM-align superposition of **GDM-NT-543** (blue) with its closest structural match (gray) from the PDB (March 2026), showing folds distinct from known haem proteins. Insets show the haem binding pocket of designs and natural proteins

Beta-Sheet Content Analysis Across Natural and Designed Haem Proteins

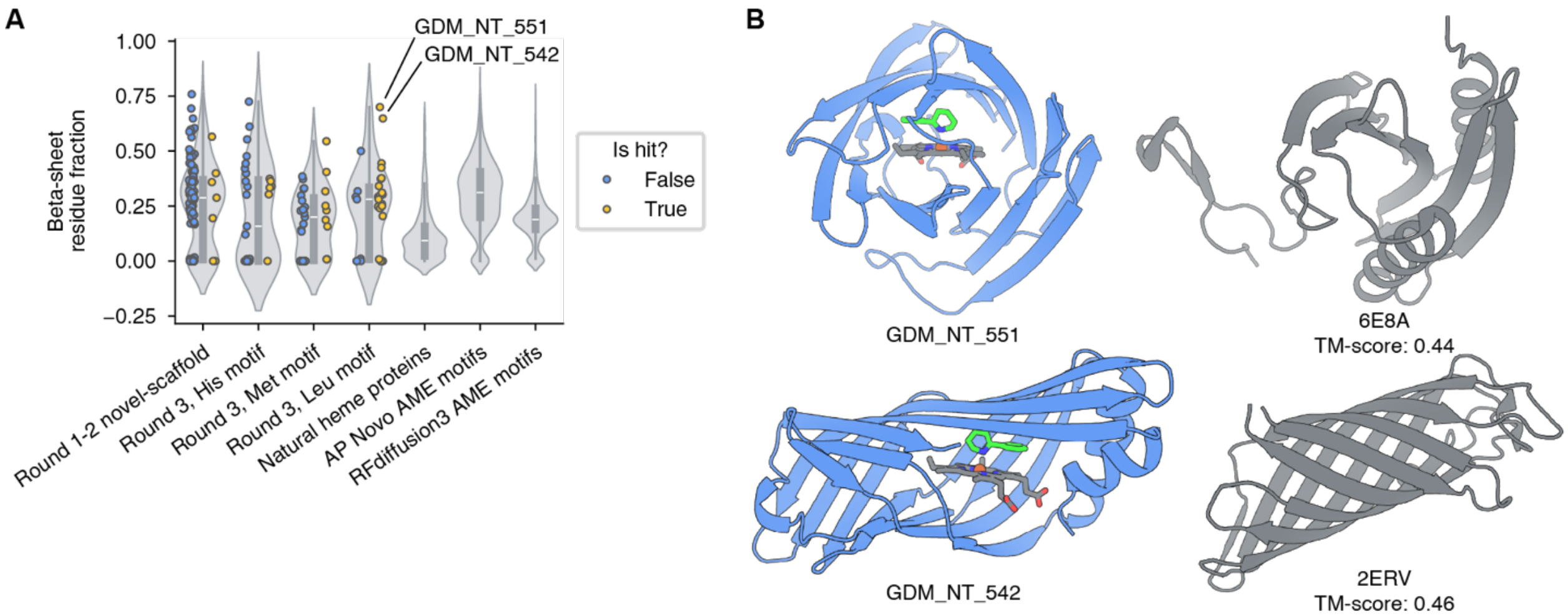

Figure. S3. Beta-sheet content of designs. (A) Fraction of residues annotated as beta sheet by DSSP, for different design rounds, natural haem-containing proteins in the PDB, AP Novo designs generated using the input motifs from the AME benchmark, and RFdiffusion3 designs generated for the same AME motifs. PDB proteins were filtered to 20,402 entries containing HEM and then clustered by mmseqs2 at 70% identity to 1,019 entries. Round 1-2 designs and generation settings for the AME benchmark are as described by the AP Novo paper (*6*). **(B)** Design structures (blue) and nearest Foldseek hit in the PDB (grey) for the two active designs with highest beta-sheet content. **GDM-NT-551** has a ***Tm*Pgb-wt** normalized activity of 3.9 and **GDM-NT-542** has an activity of 0.74. Both designs have 99:1 rr for piperidine.

Design with No-Protein Motif
We evaluated the distribution of axial ligands when sampling without an explicitly defined axial ligand in the motif.

Distribution of Residues at the Presumed Axial Position Across Heme-binding Protein Sequences in the PDB and "no-protein motif" designs

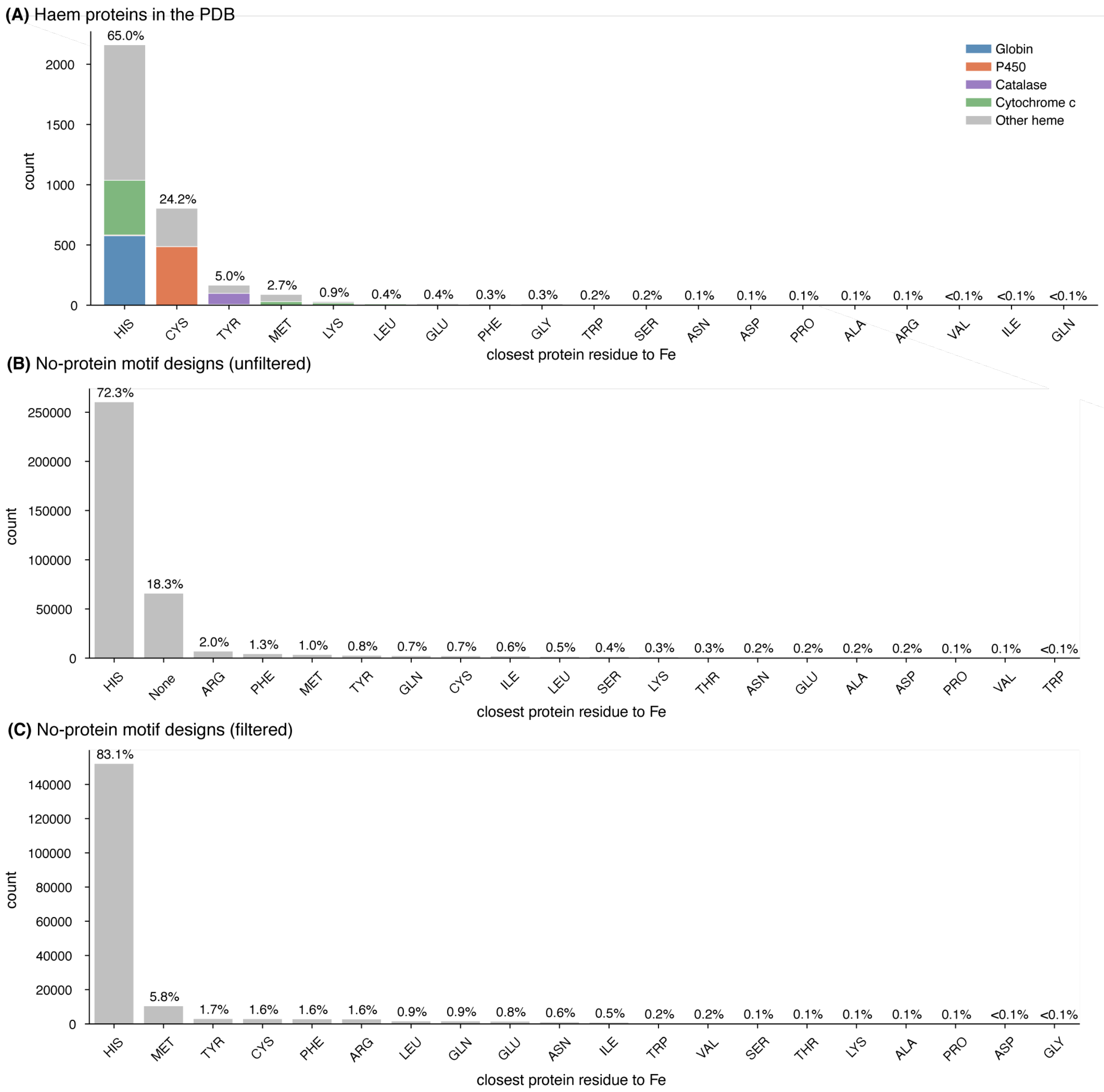

Figure S4 Distribution of the closest protein residue to Fe in natural haemoproteins and no-protein motif designs. (A) Haem-binding protein sequences in the PDB. For each haem iron, the nearest protein residue was identified from unique haem-binding sequences in the March 2026 PDB. Entries were deduplicated by collapsing those sharing the same protein sequence and nearest-residue identity; non-standard amino acid codes (e.g., methylhistidine, selenocysteine) were excluded (n = 3,326 unique entries). Bars are coloured by protein family. Histidine is the most frequently observed nearest residue, notably serving as the axial ligand in globins. Cysteine is the second most common characteristic of P450 thiolate

ligation. **(B)** Unfiltered no-protein motif designs. Designs were generated using input motifs consisting only of a haem cofactor and N-heterocyclic product, without specifying a presumed axial ligand (n = 360,344 designs). The closest protein residue to Fe was assigned as the amino acid with the nearest side-chain atom to the haem iron, provided the distance was ≤3.5 Å; designs with no residue within this cutoff are labelled "None" and are not included in the next panel. **(C)** No-protein motif designs after basic quality filtering. From the initial no-protein motifdesigns in (B), filters on sequence composition and ligand clashes were applied (retaining n = 183,122, 50.8%). The closest residue to Fe was assigned by majority vote across 7 AF3 seeds, designating the residue with the nearest side-chain atom to the haem iron (≤3.5 Å).

Generator

We identified the side-chain atom closest to the HEM iron in the sampled design. If this distance was 3.5 Å or closer, that atom's residue was designated as the axial ligand for that seed.

Table S11. Distribution of residues at proximal axial position using no-protein motif designs (unfiltered)

Residue	Count	Percent
HIS	260580	72.3%
None	65968	18.3%
ARG	7089	2.0%
PHE	4515	1.3%
MET	3706	1.0%
TYR	2865	0.8%
GLN	2462	0.7%
CYS	2387	0.7%
ILE	2192	0.6%
LEU	1671	0.5%
SER	1480	0.4%
LYS	1174	0.3%
THR	928	0.3%
ASN	853	0.2%
GLU	688	0.2%
ALA	603	0.2%
ASP	549	0.2%
PRO	338	0.1%
VAL	224	0.1%
TRP	72	<0.1%

Generator + Light Filtering (AF3 Agreement)

We evaluated designs with the heme and substrate across 7 structure prediction seeds. For each seed, we identified the side-chain atom closest to the HEM iron. If this distance was 3.5 Å or closer, that atom's residue was designated as the axial ligand for that seed.

We then took a majority vote across the 7 seeds to determine the final axial ligand, filtering out any designs where no single residue held a majority.

We removed any samples where the maximum axial heme distance exceeded 5.5 Å and applied the following basic filters on the generated sequence and structure:

sample/percent_ligand_bb_clashes: ≤ 5.0
sequence/max_consecutive_identical_aatypes: ≤ 5
sequence/max_single_aatype_frac: ≤ 0.25
sequence/num_blocklisted_motifs: ≤ 0
sequence/unknown_count: ≤ 0
sequence/seq_len: ≥ 90

These basic filters removed roughly half of the initial samples:

- **Passed:** 183,122 / 360,344 (50.8%)
- **Removed:** 177,222 (49.2%)

Of the remaining 183,122 samples, the distribution of generated axial ligands was as follows:

Table S12. Distribution of residues at proximal axial position using no-protein motif designs (filtered)

Residue	Count	Percent
HIS	152,200	83.1%
MET	10,550	5.8%
TYR	3,043	1.7%
CYS	2,999	1.6%
PHE	2,881	1.6%
ARG	2,849	1.6%
LEU	1,715	0.9%
GLN	1,691	0.9%
GLU	1,552	0.8%
ASN	1,164	0.6%
ILE	895	0.5%
TRP	328	0.2%
VAL	280	0.2%
SER	251	0.1%
THR	213	0.1%
LYS	179	0.1%

Residue	Count	Percent
ALA	143	0.1%
PRO	111	0.1%
ASP	75	<0.1%
GLY	3	<0.1%

As these data demonstrate, without explicitly enforcing the axial ligand in the motif, the generator predominantly defaults to histidine and does not generate samples with alternative axial ligands at meaningful frequencies.

It is also worth noting that this distribution reflects only light filtering. Once the full critic is applied–where pass rates are approximately 10 times lower for methionine compared to histidine–this distribution tilts even further toward histidine.

Comparison between GDM-NT-401 and *Tm*Pgb-PIP-G7

Predicted Structures from AF3-LA of Different Protein Scaffolds in Complex with Alkyl Nitrenoid Intermediates

Predicted structures from AF3-LA of the ***Tm*Pgb-PIP-G7** and **GDM-NT-401** scaffolds in complex with various substrates from the substrate scope analysis. For each complex, 8 structural predictions were generated using AF3-LA with distinct random seeds. The final displayed structure was chosen by taking the top two predictions based on substrate-atom pLDDT, and subsequently selecting the conformation with the minimum N1–C5 distance (measured between the substrate nitrogen and the fifth carbon of the alkyl chain), or the N1-C6 distance for substrate **2n**. Substrates **2a** and **2b** are shown together as AF3-LA cannot model different isotopes. Using this filtering pipeline, AF3-LA consistently predicts a catalytically favourable near-attack conformation for substrates within the **GDM-NT-401** scaffold - a geometry notably absent in the ***Tm*Pgb-PIP-G7** scaffold. The ***Tm*Pgb-PIP-G7** sequence was folded incorporating multiple sequence alignment (MSA) data (using databases MGnify 2022-05, UniRef90 2022-05, SmallBFD).

Predicted structure of **GDM-NT-401** *in complex with alkyl nitrenoid intermediates derived from substrate scope study in Fig. 4*

All predicted structures feature a near-attack conformation with the alkyl chain bending toward the heme co-factor.

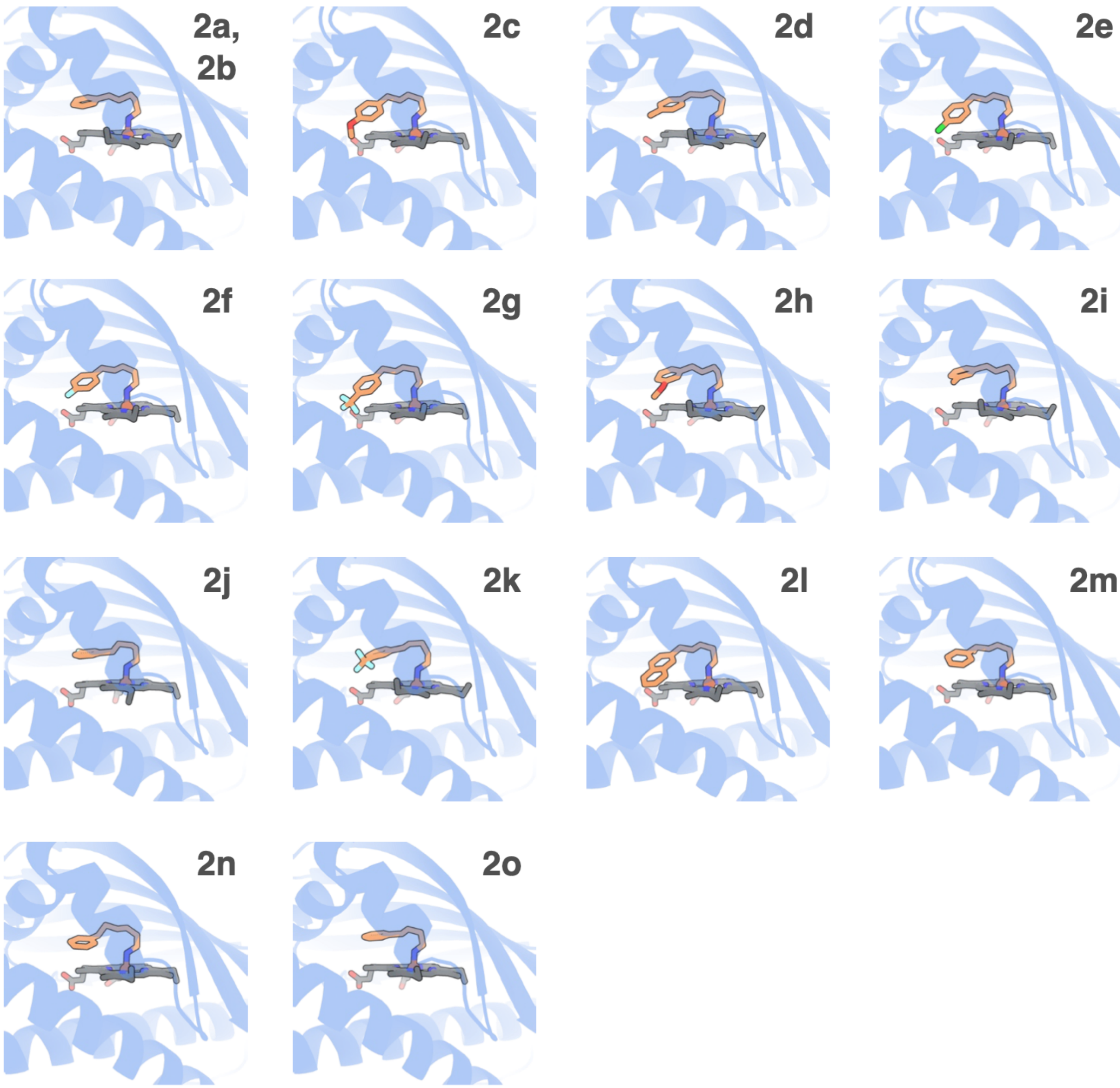

Figure S5. Predicted structures from AF3-LA of **GDM-NT-401** in complex with various alkyl nitrenoid intermediates from the substrate scope study.

*Predicted structure of **Tm**Pgb-PIP-G7 in complex with alkyl nitrenoid intermediates derived from substrate scope study in Fig. 4*

All predicted structures feature a linear conformation

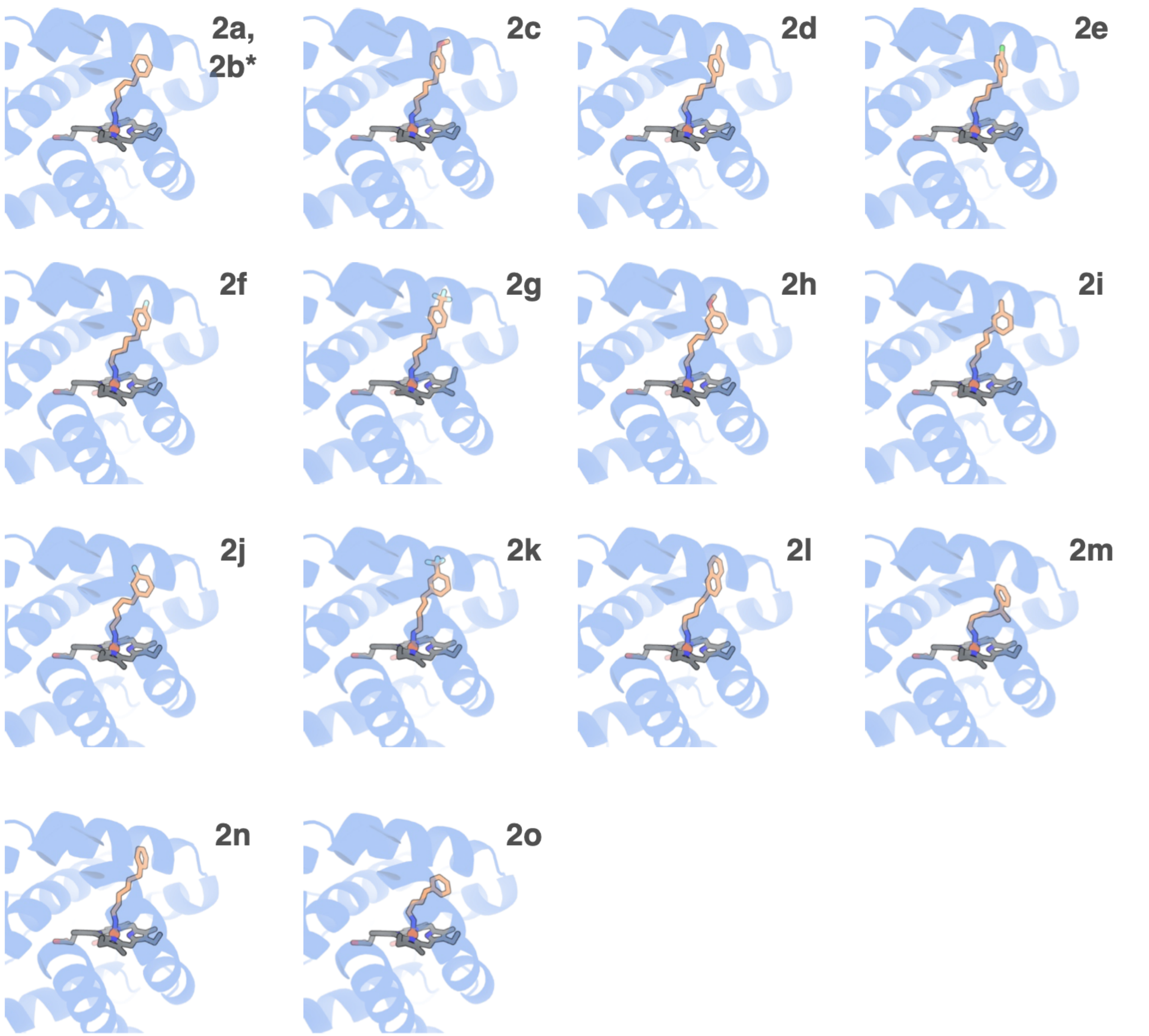

Figure S6 Predicted structures from AF3-LA of ***Tm*Pgb-PIP-G7** in complex with various alkyl nitrenoid intermediates from the substrate scope study.

Summary of N1–C5 distances in predicted structures from AF3-LA of **GDM-NT-401** and ***TmPgb-PIP-G7*** *in complex with various substrates from the substrate scope analysis.*

In general, the predicted structures of **GDM-NT-401** in complex with different alkyl nitrenoid intermediates have a shorter N1–C5 distances compared to ***Tm*Pgb-PIP-G7**. This result corresponds well with the overall higher yields obtained with **GDM-NT-401**.

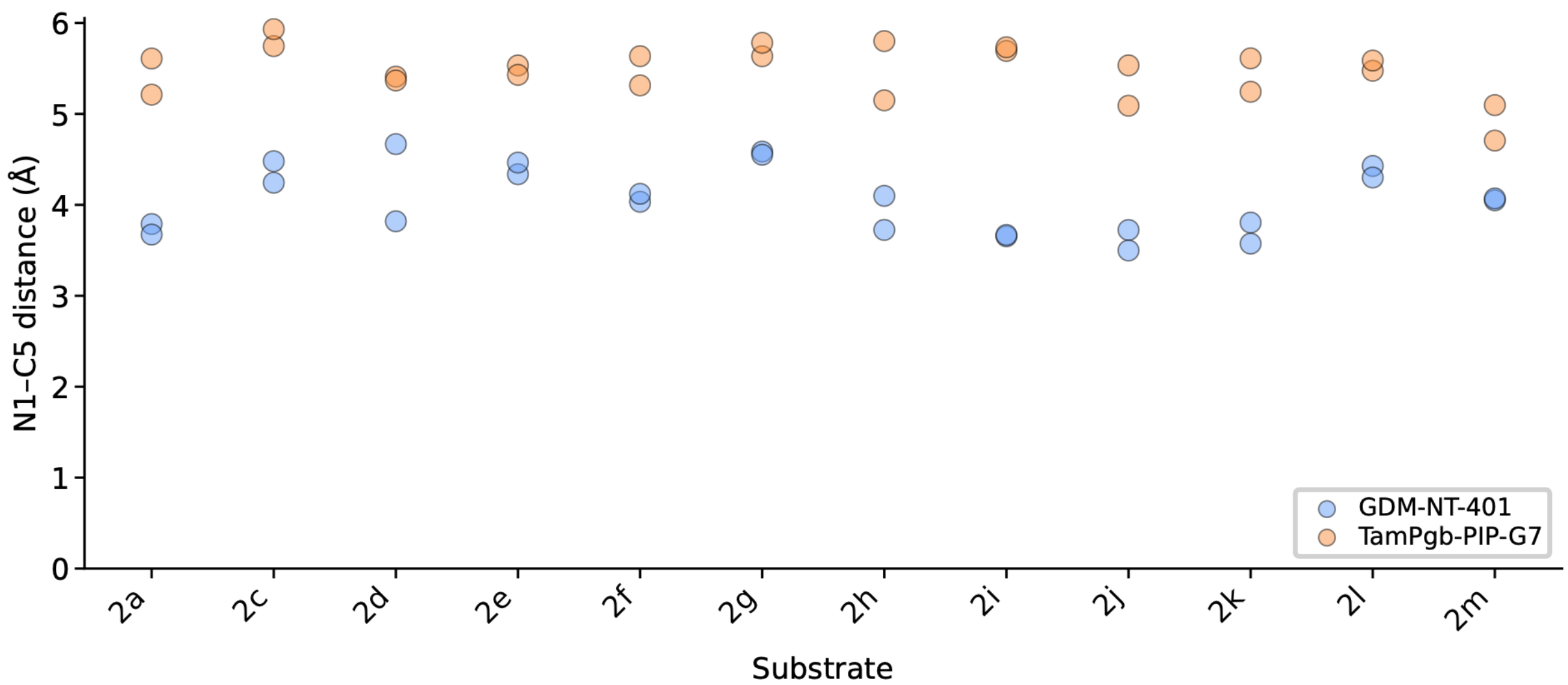

Figure S7. N1–C5 distances in predicted structures from AF3-LA of ***Tm*Pgb-PIP-G7** in complex with various alkyl nitrenoid intermediates from the substrate scope study.

Comparison between ***Tm*Pgb-PIP-G7** and **GDM-NT-401** *for the formation of* **2n**

A comparison between ***Tm*Pgb-PIP-G7** and **GDM-NT-401** on their predicted structure from AF3-LA in complex with corresponding alkyl nitrenoid intermediate for the formation of **2n** is shown below in Fig. S6. 1) **GDM-NT-401** demonstrated a close-to-attack conformation, while ***Tm*Pgb-PIP-G7** demonstrated a relatively linear conformation. The corresponding N1–C6 distances also demonstrated that **GDM-NT-401** has a shorter average distance than ***Tm*Pgb-PIP-G7**. This result corresponds well to the experimental data where 13% yield of 2n was observed using **GDM-NT-401** and no detectable activity was observed using ***Tm*Pgb-PIP-G7**. 2) Despite the average N1–C6 and N1–C5 distances (Fig. S5) for **GDM-NT-401** are similar (both around 4 Å), the N1–H–C6 versus N1–H–C5 distance and angle clearly favours the formation of a seven-membered ring.

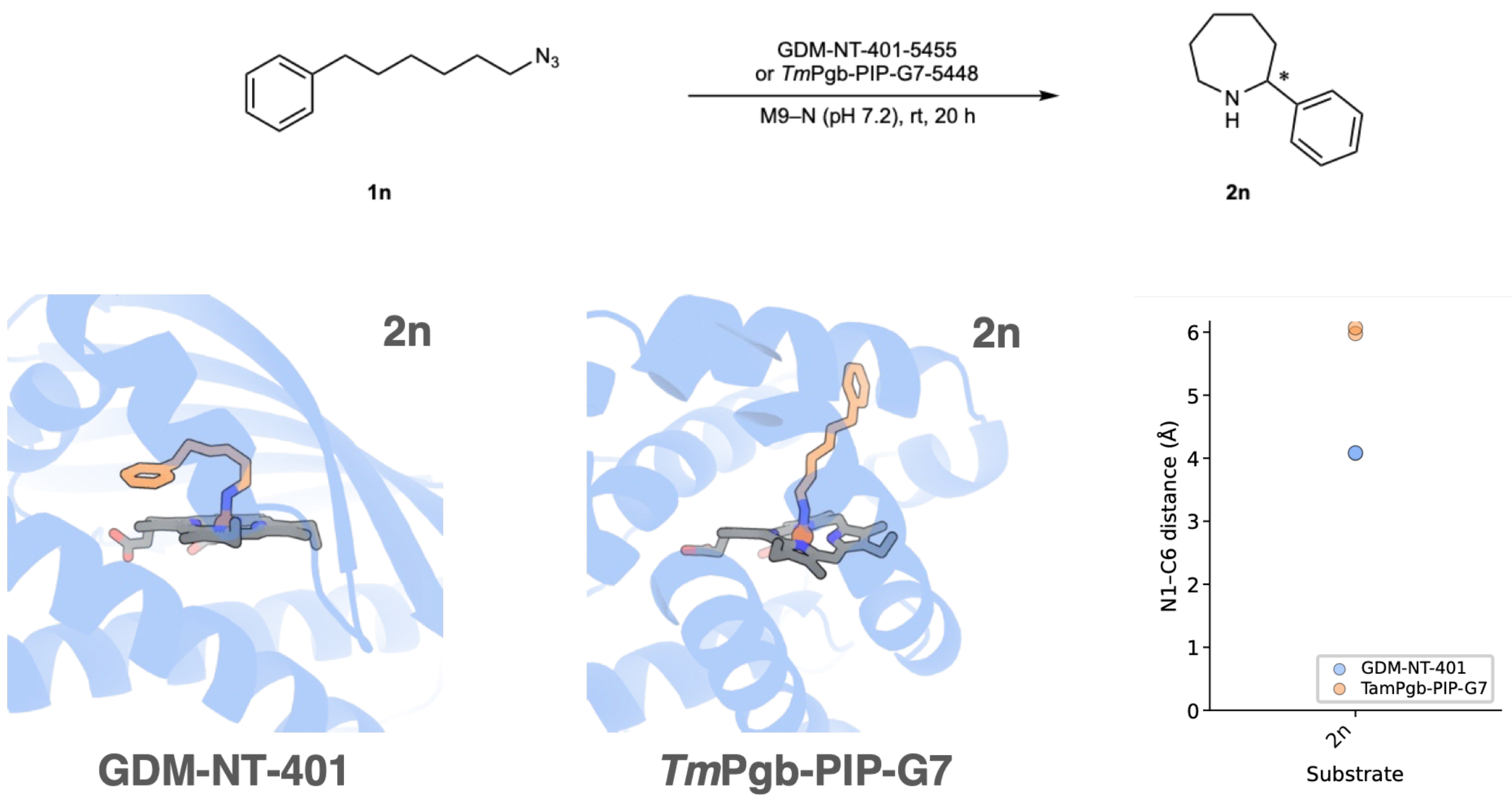

Figure S8. Predicted structures from AF3-LA of **GDM-NT-401** in complex with alkyl nitrenoid intermediate to form **2n**.

UV-Vis Analysis of GDM-NT-401

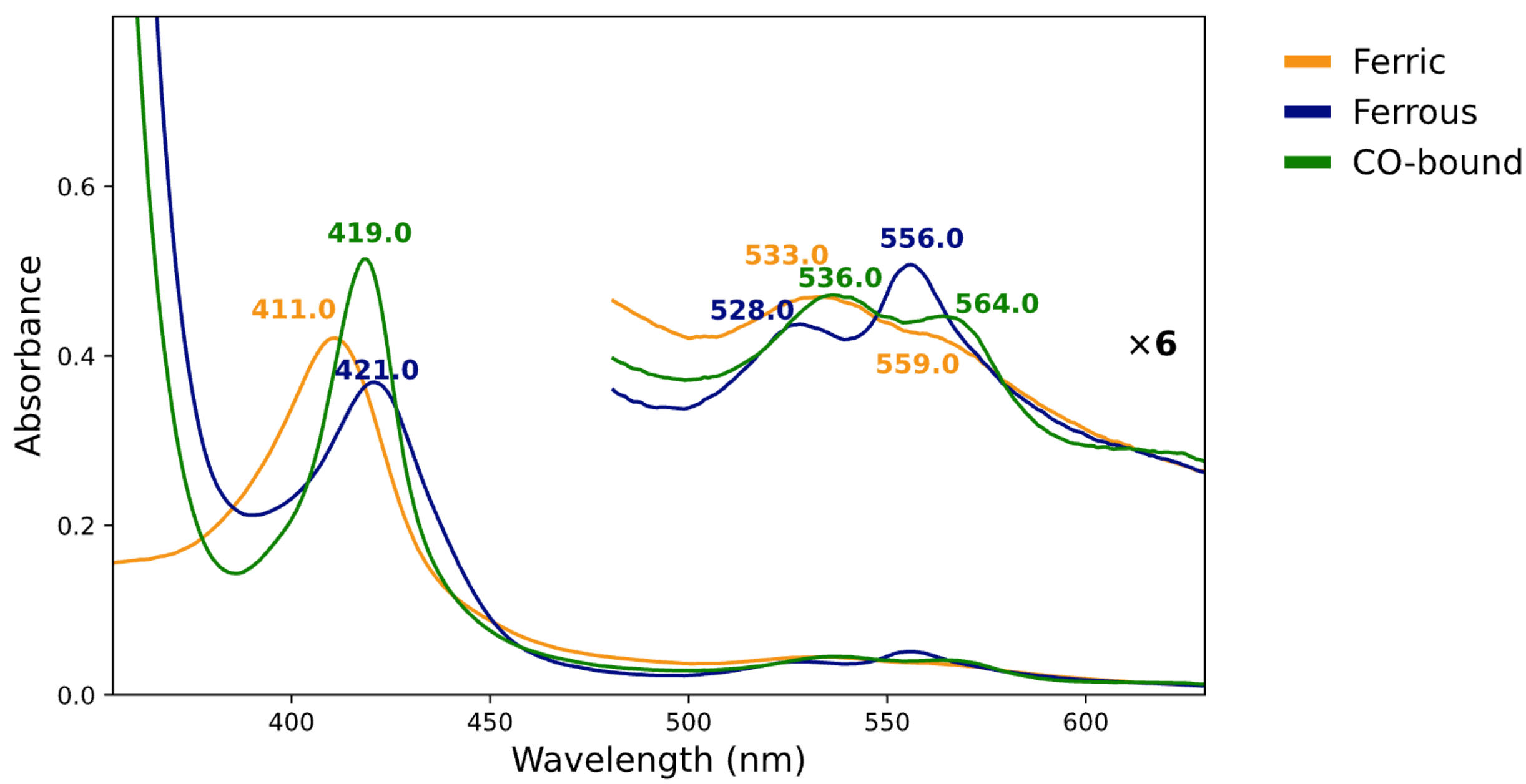

Figure S9. UV-vis spectrum of **GDM-NT-401**. Yellow: ferric state. Blue: ferrous state. Green: CO-bound ferrous state.

Analytical-Scale Enzymatic Reaction Yield Analysis and Calibration Curves for Products

All enzymatic reactions on analytical scale were conducted following the general procedure described in **General Information**. Reactions for each substrate were set up in triplicate. Product formation was quantified by LC-MS based on the calibration curve of the corresponding racemic standard products.

Calibration curves of reference compounds were conducted for the determination of yield. For each substrate, at least five different concentrations of product (0.125, 0.25, 0.5, 1.0, 1.5, 2, 2.5, 3, 4, and 5 mM) were prepared in 400 µL M9–N (pH = 7.2) buffer. This solution was quenched using the reaction work-up procedure described in **General Information** using 1,2,3-trimethylbenzene as internal standard. The samples were analyzed by HPLC based on UV absorbance at 254, 235, or 210 nm. The calibration curves depict the ratio of concentration in mM (y-axis) against product area to internal standard area (x-axis). *Notes*: Pdt = integration of product on UV spectroscopy, IS = integration of internal standard on UV spectroscopy. In cases where a set of reactions are all having <10% yields, a calibration curve using single ion channel ion count was used without referring to an internal standard.

Chiral protic HPLC-MS was used to determine the enantioselectivity of the reactions. Single ion channel MS spectroscopy was included to support the clean separation of the two enantiomers. For the analysis of regioselectivity, all measurements were made without considering the different response of regio isomers on HPLC-MS. Considering the ratio of the minor regioisomers is generally below 20%, this adjustment won't make large differences in the final regioselectivity.

Part 1: *Tm*Pgb-PIP lineage analysis.

All yields were calculated using a single ion channel calibration curve measured on the same day, using the same HPLC-MS, and with the same bottle of eluent. All yields within the ***Tm*Pgb-PIP** lineage using whole *E. coli* cell as catalysts are within the linear range of the calibration curve. All yields represent *only* the yields of **2a**.

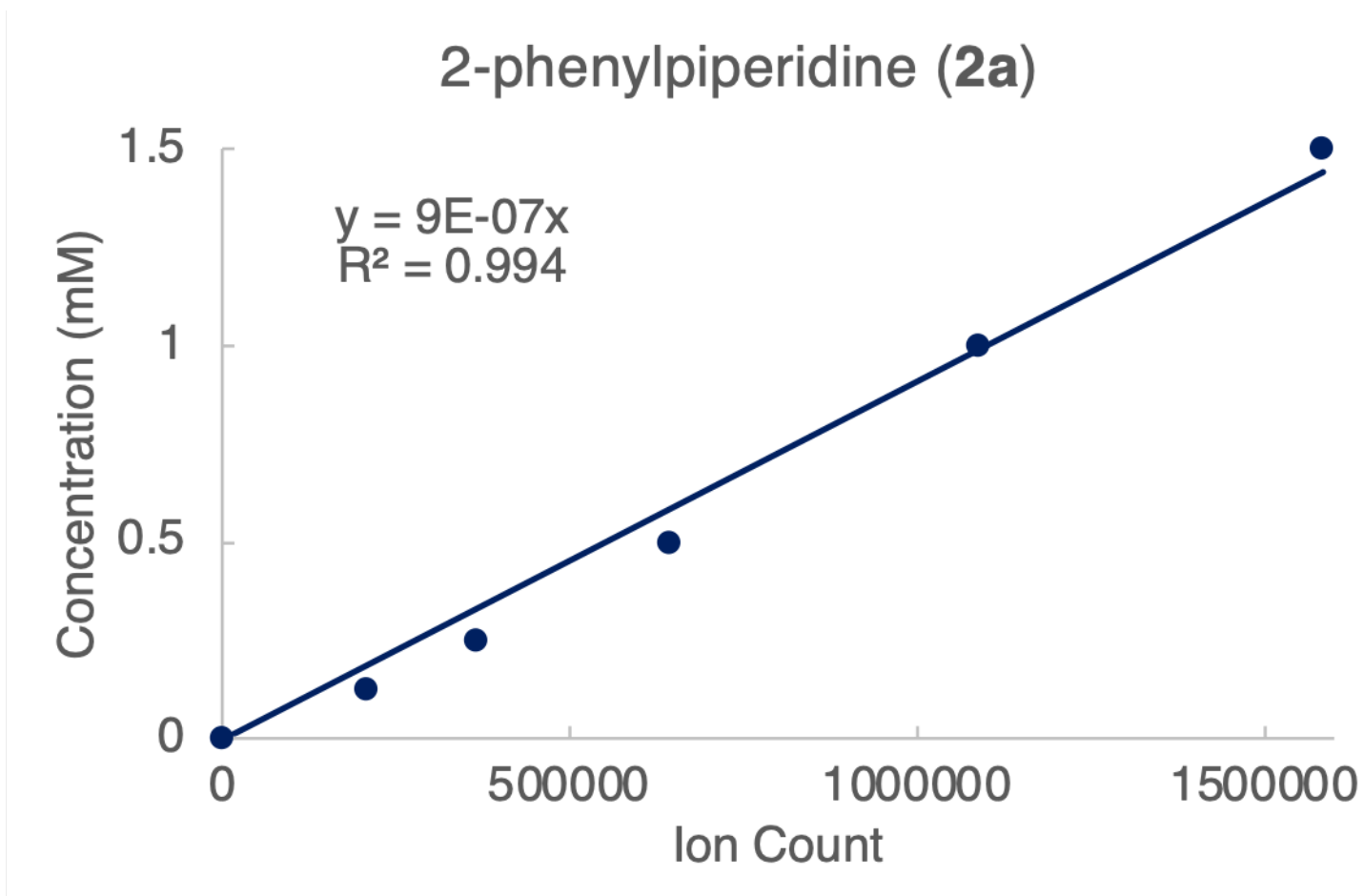

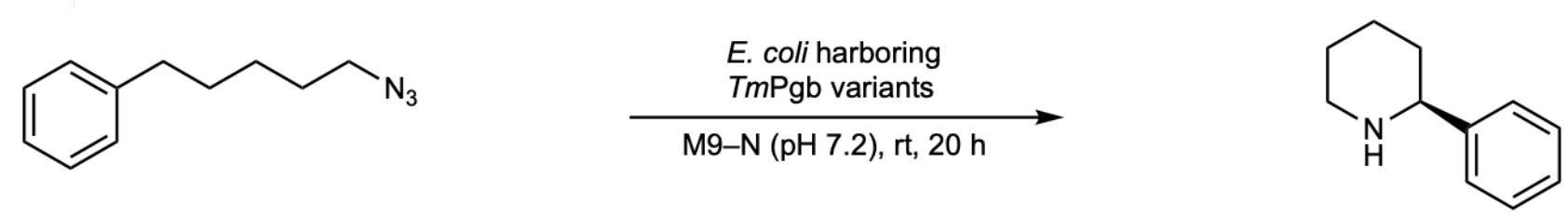

1a 2a

***Tm*Pgb-wt** (whole *E.coli* cell with 11.5 µM heme loading)

Entry	Product	Yield (%)	Average (%)	STDEV	Average TTN
1	4156.1	0.08			
2	3717.6	0.07	0.07	0.01	0.3
3	4340.9	0.08			

***Tm*Pgb-PIP-G1-5442** (whole *E.coli* cell with 11.9 μM heme loading)

Entry	Product	Yield (%)	Average (%)	STDEV	Average TTN
1	28315.9	0.52			
2	28773.1	0.52	0.54	0.03	2
3	31091.8	0.57			

***Tm*Pgb-PIP-G2-5443** (whole *E.coli* cell with 11.4 μM heme loading)

Entry	Product	Yield (%)	Average (%)	STDEV	Average TTN
1	43809.0	0.80			
2	44714.0	0.81	0.80	0.02	3
3	42667.4	0.78			

***Tm*Pgb-PIP-G3-5444** (whole *E.coli* cell with 9.3 μM heme loading)

Entry	Product	Yield (%)	Average (%)	STDEV	Average TTN
1	47379.5	0.86			
2	50912.8	0.93	0.91	0.04	5
3	51474.2	0.94			

***Tm*Pgb-PIP-G4-5445** (whole *E.coli* cell with 11.7 μM heme loading)

Entry	Product	Yield (%)	Average (%)	STDEV	Average TTN
1	97787.6	1.78			
2	106253.1	1.94	1.84	0.08	8
3	99661.0	1.82			

***Tm*Pgb-PIP-G5-5446** (whole *E.coli* cell with 10.9 μM heme loading)

Entry	Product	Yield (%)	Average (%)	STDEV	Average TTN
1	183923.9	3.35			
2	182699.1	3.33	3.38	0.06	15
3	189104.1	3.45			

***Tm*Pgb-PIP-G6-5447** (whole *E.coli* cell with 10.7 μM heme loading)

Entry	Product	Yield (%)	Average (%)	STDEV	Average TTN
1	142822.3	2.60			
2	131523.9	2.40	2.48	0.11	12
3	134397.7	2.45			

***Tm*Pgb-PIP-G7-5448** (whole *E.coli* cell with 10.2 μM heme loading)

Entry	Product	Yield (%)	Average (%)	STDEV	Average TTN
1	302443.6	5.51			
2	333834.6	6.08	5.97	0.41	29
3	346630.9	6.32			

Part 2: ***De novo*** **design with recycled scaffold data analysis.**

All yields were calculated using a single ion channel calibration curve measured on the same day, using the same HPLC-MS, and with the same bottle of eluent. All yields listed in this section were obtained using whole *E. coli* cell as catalysts and are within the linear range of the calibration curve. All yields represent *only* the yields of **2a**.

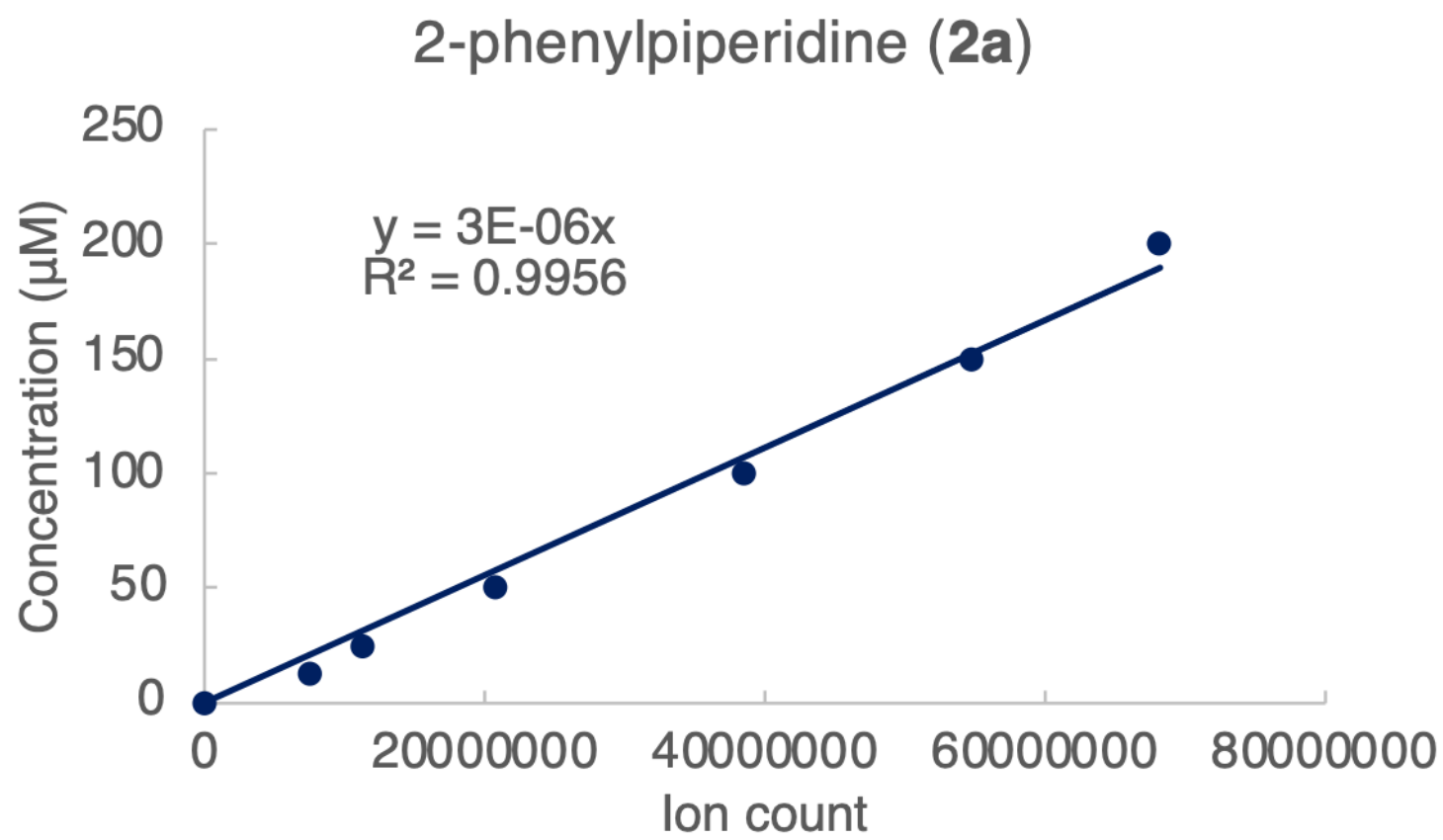

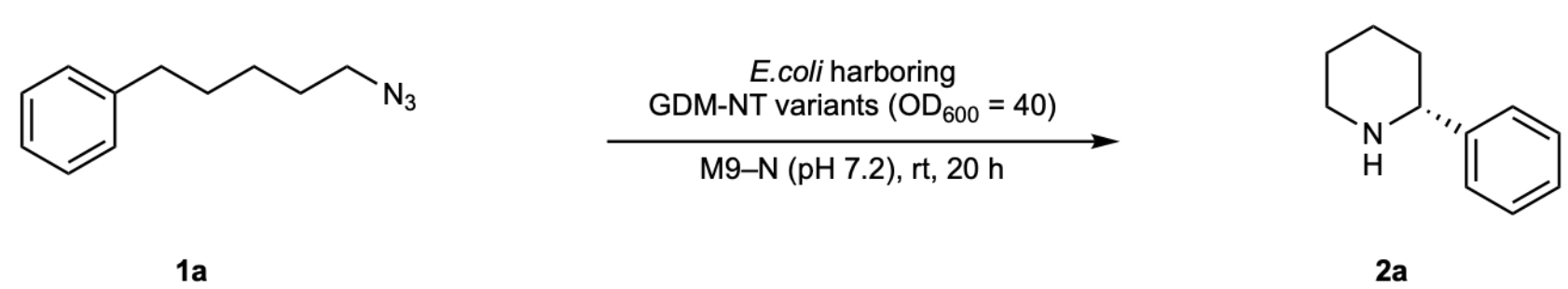

Table S13. Results using selected variants in recycled-scaffold design based on **GDM-NT-121**

	Rxn 1	Rxn 2	Rxn 3	Hemeloading (µM)	Average Yield (%)	Average TTN
***Tm*Pgb-wt**	321052	203194	238715	6.5	0.01	0.1
**GDM-NT-121**	1616377	1595036	1623099	2.6	0.09	1.7
**GDM-NT-215**	3137075	3193042	3175384	1.7	0.18	5.2
**GDM-NT-217**	2094515	2021621	1987190	1.8	0.11	3.2
**GDM-NT-226**	28209976	27907585	28518283	2.8	1.58	28.2
**GDM-NT-227**	1647956	1518064	1659073	3.1	0.09	1.5
**GDM-NT-228**	480373	476117	483799	1.6	0.03	0.9

Table S14. Results using selected variants in recycled-scaffold design based on **GDM-NT-151**

	Rxn 1	Rxn 2	Rxn 3	Hemeloading (µM)	Average Yield (%)	Average TTN
***Tm*Pgb-wt**	321052	203194	238715	6.5	0.01	0.1
**GDM-NT-151**	578534	578840	542094	1.7	0.03	0.9
**GDM-NT-350**	1795490	1722655	1735434	2.3	0.10	2.1
**GDM-NT-356**	5384664	5580567	6000699	2.7	0.32	5.8
**GDM-NT-359**	3940367	4047304	4345490	3.6	0.23	3.2
**GDM-NT-361**	1321241	1475285	1588278	1.7	0.08	2.5
**GDM-NT-362**	8486036	8421601	8587747	13.8	0.47	1.7

Part 3: Evolution from **GDM-NT-226** and **GDM-NT-362.**

After identifying GDM-NT-226 **and GDM-NT-362** as the starting point for further evolution, we performed one round of random mutagenesis using error-prone PCR and identified multiple variants with beneficial mutations.

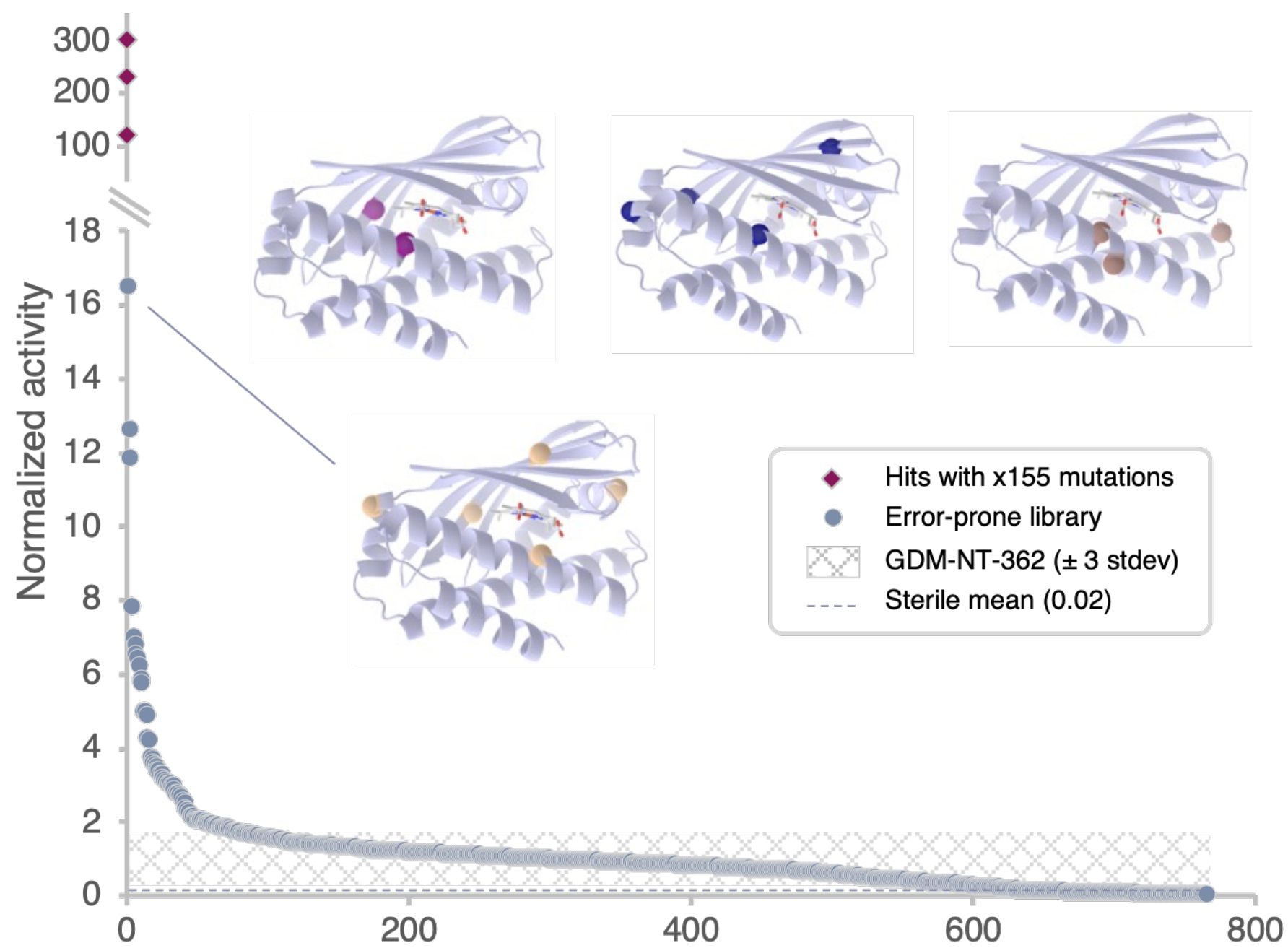

Figure S10. Retention of function curve for directed evolution using **GDM-NT-362** as parent. Three variants demonstrated higher than 100-fold improvement compared to **GDM-NT-362**. The illustration of these three variants with mutations highlighted in purple, blue, and brown were generated by AlphaFold 3. Note that all three variants have a mutation at a shared site 155. Three additional variants demonstrated 10- to 20-fold improvement compared to **GDM-NT-362**. The top hit with mutations highlighted in yellow was generated by AlphaFold 3. The area highlighted in cross pattern represents results of **GDM-NT-362** within ±3×standard deviation. The sterile mean of the reaction with respect to **GDM-NT-362** is high in dash line at the bottom of the figure.

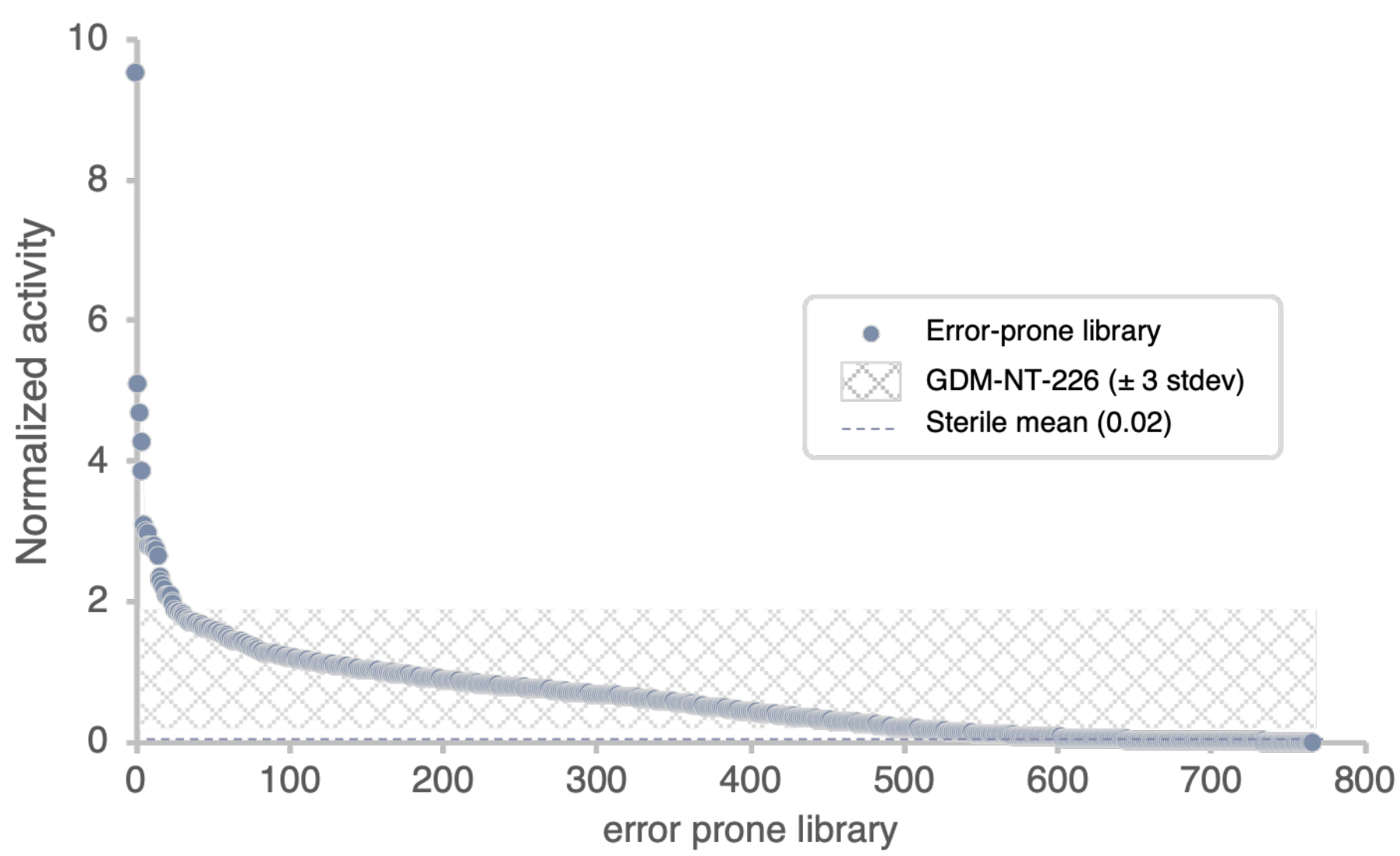

Figure S11. Retention of function curve for directed evolution using **GDM-NT-226** as parent. Four variants demonstrated >4-fold improvement compared to **GDM-NT-226**. The area highlighted in cross pattern represents results of **GDM-NT-226** within ±3×standard deviation. The sterile mean of the reaction with respect to **GDM-NT-226** is high in dash line at the bottom of the figure.

Part 4: Additional engineering from top hit in error-prone library

One variant with four mutations V53L, 68V, E99G, H155L, later designated as **GDM-NT-399**, was used as the parent for further protein engineering. We identified site 155 as the designed site for axial ligand of the heme co-factor. As a result, we focused on mutations at site 155 and adjacent site 156, while keeping the remaining three mutations at site 53, 68, and 99. We later determined that these three mutations (V53L, 68V, E99G) introduced through error-prone PCR were beneficial to the reaction yield (comparing **GDM-NT-401** with **GDM-NT-400**). All yields represent only the yields of **2a**.

After this final round of protein engineering, the top-performing variant **GDM-NT-401** was selected as final variant for substrate scope study.

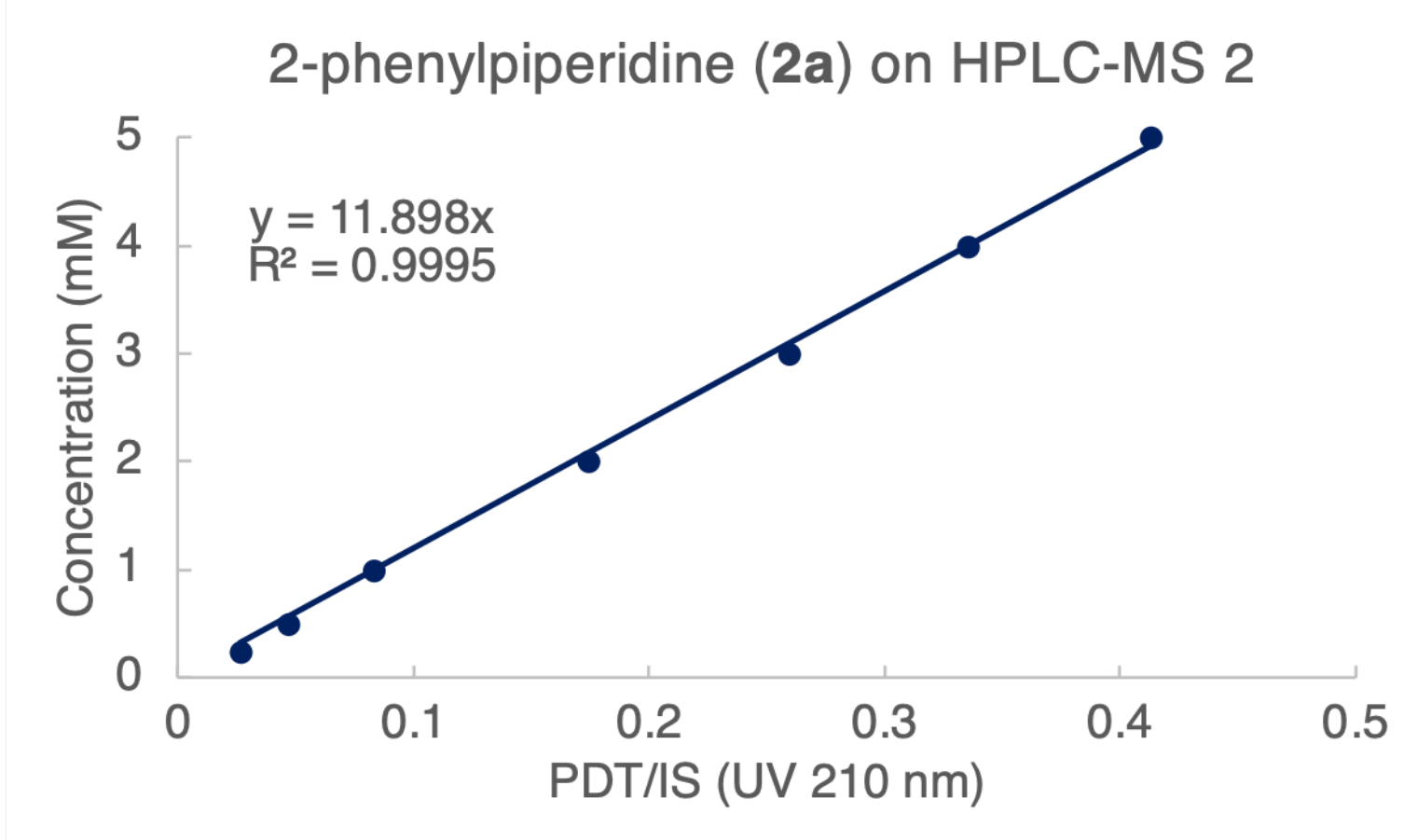

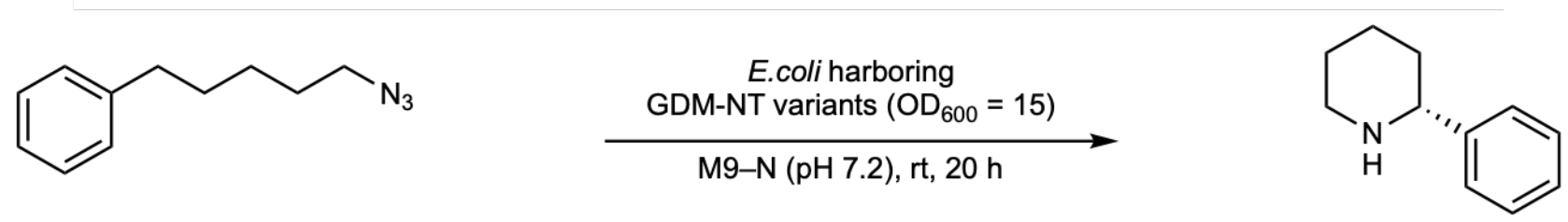

1a **2a**

Table S15. Results of final round of protein engineering. Analytical-scale reactions were performed on a 96-well plate, with an averaged OD_{600} of 15.

	x155	Mutations from GDM-NT-362	PDT	IS	Yield (%)
**GDM-NT-362**	H	-	NA	NA	<1
**GDM-NT-404**	H	V53L-I68V-E99G	NA	NA	<1
**GDM-NT-405**	H	V53L-I68V-E99G-M156A	NA	NA	<1
**GDM-NT-399**	L	V53L-I68V-E99G-H155L	444.1	5706.2	18.5
**GDM-NT-406**	L	V53L-I68V-E99G-H155L-M156L	744.6	5673.7	31.2
**GDM-NT-403**	L	V53L-I68V-E99G-H155L-M156A	781.8	5781.5	32.2
**GDM-NT-407**	M	V53L-I68V-E99G-H155M	127.7	5632.3	5.4
**GDM-NT-401**	M	V53L-I68V-E99G-H155M-M156L	872.1	5744.3	36.1
**GDM-NT-402**	M	V53L-I68V-E99G-H155M-M156A	565.6	5810.3	23.2
**GDM-NT-400**	M	H155M-M156L	630.5	5761.5	26.0

Part 5: Reaction condition optimization using purified protein.

Despite reactions performed using *e.coli* harboring ***Tm*Pgb-PIP** variants and **GDM-NT** variants do not require the addition of external reductant; reactions performed with purified protein exhibited diverging behavior between final variants ***Tm*Pgb-PIP-G7** and **GDM-NT-401**. While no external reductant is required for reactions performed with purified ***Tm*Pgb-PIP-G7**, a catalytic amount of $Na_2S_2O_4$ was required for reactions performed with **GDM-NT-401**. Additionally, this trend was consistent with a previously reported variant ***Tm*Pgb-PYR-G2-5441**, where no external reductant was required.

An aliquot of ***Tm*Pgb-PYR-G2-5441**, ***Tm*Pgb-PIP-G7**, or **GDM-NT-401** were added to a 2-mL reaction vial and adjusted to 10 µM *final* heme concentration with M9–N (pH 7.2) buffer. The reaction vials were transferred to a coy chamber. Subsequently, different amount of a freshly prepared stock solution of $Na_2S_2O_4$ (100 mM in M9–N buffer) was added to the vial and gently mixed by shaking the vial holder for 10 seconds. Final concentrations of $Na_2S_2O_4$ added to the reactions vary from 0 mM, 1 mM, 2.5 mM, to 5 mM. Then, 20 µL of a stock solution of **1a** (100 mM in ethanol) was added to the reaction mixture. The reaction vial was capped and shaken for 20 h at 700 rpm and room temperature.

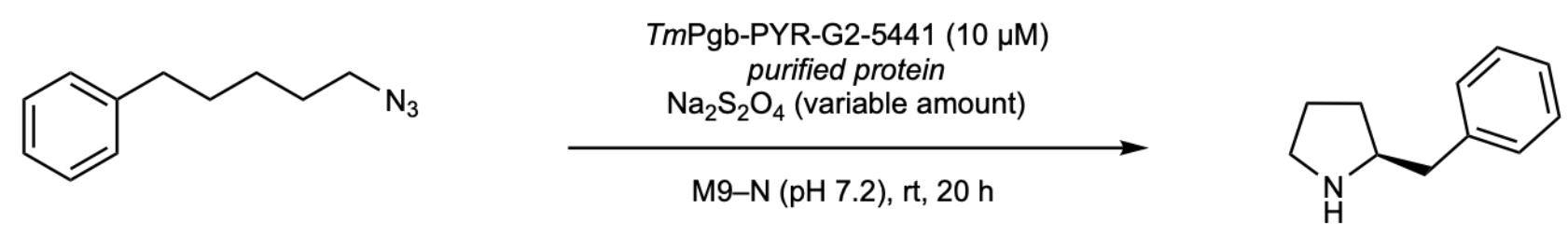

1a (5.0 mM) **3a**

Table S16. Effect of external reductant for ***Tm*Pgb-PYR-G2-5441** (purified enzyme) on UV channel.

$Na_2S_2O_4$ (mM)	PDT	IS	Yield (%)	Average Yield (%)	STDEV
0	742.2	5481.3	26.7		
0	721.2	5322.4	26.7	27	1.09
0	782.7	5397.4	28.6		
1	251.8	5279.5	9.4		
1	263.6	5286.9	9.8	10	0.79
1	301.3	5427.2	10.9		
2.5	32.0	5230.0	1.2		
2.5	40.3	5259.3	1.5	1.2	0.37
2.5	20.8	5340.2	0.8		
5	17.4	5310.3	0.6		
5	18.5	5345.3	0.7	0.6	0.19
5	8.7	5133.0	0.3		

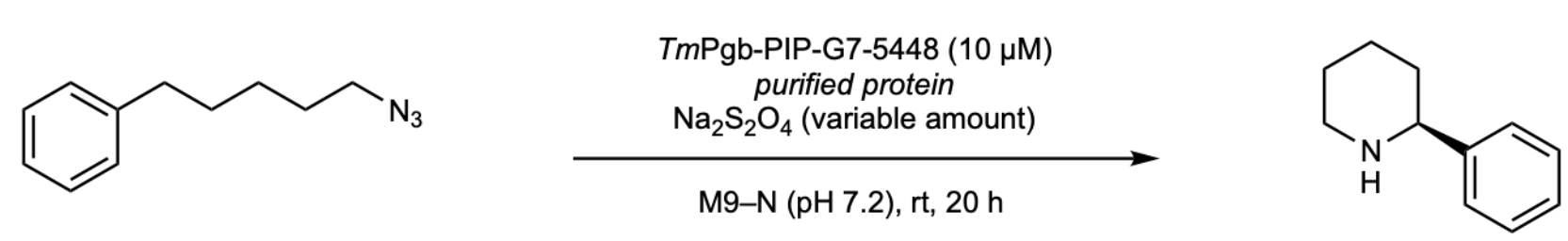

1a (5.0 mM) **2a**

Table S17. Effect of external reductant for ***Tm*Pgb-PIP-G7-5448** (purified enzyme) on single ion mass channel.

$Na_2S_2O_4$ (mM)	PDT ion count Rxn 1	PDT ion count Rxn 2	PDT ion count Rxn 3	PDT ion count average	Normalized Yield	STDEV
0	240283.2	276580.7	252683.0	256515.6	1.00	18449.8

1	163707.1	167370.2	205207.6	178761.6	0.70	22976.0
2.5	32425.2	26376.1	36414.4	31738.6	0.12	5054.3
5	20368.7	21099.1	20030.4	20499.4	0.08	546.2

Table S18. Effect of external reductant for ***Tm*Pgb-PIP-G7-5448** (purified enzyme) on UV channel using 0 mM and 1 mM $Na_2S_2O_4$ as external reductant. This result agrees with **Table S15**. Due to low UV intensity, results using 2.5 mM and 5 mM $Na_2S_2O_4$ as external reductant are not available.

$Na_2S_2O_4$ (mM)	PDT	IS	Yield (%)	Average Yield (%)	STDEV
0	128.8	5367.0	4.7		
0	150.3	5385.1	5.5	5.1	0.39
0	144.5	5523.5	5.2		
1	83.6	5379.3	3.1		
1	82.8	5368.0	3.0	3.2	0.26
1	96.4	5422.0	3.5		

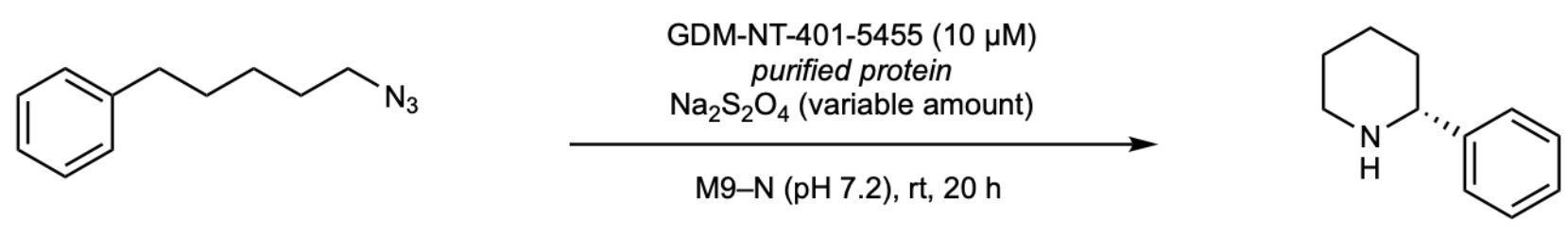

Table S19. Effect of external reductant for **GDM-NT-401-5455** (purified enzyme) on UV channel.

$Na_2S_2O_4$ (mM)	PDT	IS	Yield (%)	Average Yield (%)	STDEV
0	139.4	4539.0	6.0		
0	137.1	4701.8	5.7	6	0.21
0	136.0	4749.3	5.6		
1	1537.7	4594.7	65.9		
1	1384.1	4621.3	59.0	60	6.03
1	1247.8	4558.5	53.9		
2.5	1840.4	4517.9	80.2		
2.5	1843.8	4619.3	78.6	78	2.32
2.5	1791.3	4663.5	75.7		
5	1277.3	4548.9	55.3		
5	1318.3	4483.2	57.9	59	4.02
5	1492.5	4652.5	63.2		

Part 6: Solvent stability test for GDM-NT-401 under high concentration of ethanol.

Solvent stability test for **GDM-NT-401** was performed by conducting the reaction under different volume percentage of ethanol as co-solvent. An aliquot of **GDM-NT-401** in KPi buffer (pH 8.0) were diluted with corresponding amounts (400µL – 400 µL × ethanol% –17µL enzyme solution – 10 µL $Na_2S_2O_4$ solution) of M9–N buffer (pH 7.2). The solution was brought to a coy chamber. Subsequently, different amounts of ethanol were added (400 µL × ethanol% – 20 µL substrate **1a** solution). We noticed that at 80% or higher ethanol loading, the solution became turbid, suggesting protein denaturation. Then, 10 µL of a freshly

prepared stock solution of $Na_2S_2O_4$ (100 mM in M9–N buffer) was added to the vial and gently mixed by shaking the vial holder for 10 seconds. Finally, 20 μL of a stock solution of **1a** (100 mM in ethanol) was added to the reaction mixture. The reaction vial was capped and shaken for 20 h at 700 rpm and room temperature.

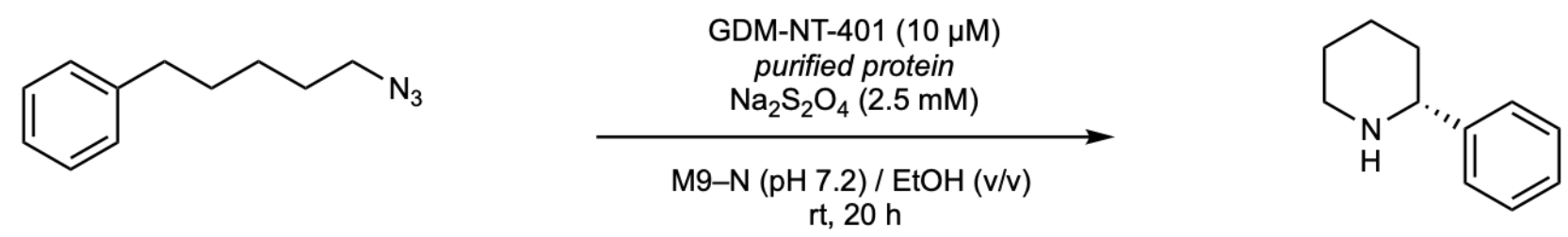

1a (5.0 mM) **2a**

Table S20. Results of solvent stability test for **GDM-NT-401** under high concentration of ethanol. Reactions were run in duplicate.

	PDT Rxn 1	IS Rxn 1	PDT Rxn 2	IS Rxn 2	Average Yield (%)	STDEV
**5% EtOH**	2124.8	5612.0	2081.0	5822.8	72	3.0
**20% EtOH**	1920.5	5466.5	1856.6	5421.6	68	1.2
**40% EtOH**	1107.6	5538.9	1010.3	5676.8	37	3.1
**60% EtOH**	1402.8	5537.4	1136.0	5422.0	46	6.1
**80% EtOH**	657.9	5449.4	666.3	5572.2	24	0.2
**90% EtOH**	109.2	5496.9	108.4	5600.0	4	0.1

Part 7: T_{50} measurement for GDM-NT-401 and *Tm*Pgb-PIP-G7

Aliquots of the stock solution of **GDM-NT-401** or ***Tm*Pgb-PIP-G7** were added to PCR tubes and diluted with M9–N (pH 7.2) buffer to 150 μL. The PCR tubes were incubated at different temperatures using a thermocycler for 1 h and cooled down to room temperature for 1 h. The solution was transferred to reaction vials and topped up to 370 μL with M9–N (pH 7.2) buffer. The solution was brought to a coy chamber. Subsequently, 10 μL of a freshly prepared stock solution of $Na_2S_2O_4$ (100 mM in M9–N buffer) was added to the vial and gently mixed by shaking the vial holder for 10 seconds. Then, 20 μL of a stock solution of **1a** (100 mM in ethanol) was added to the reaction mixture. The reaction vial was capped and shaken for 20 h at 700 rpm and room temperature.

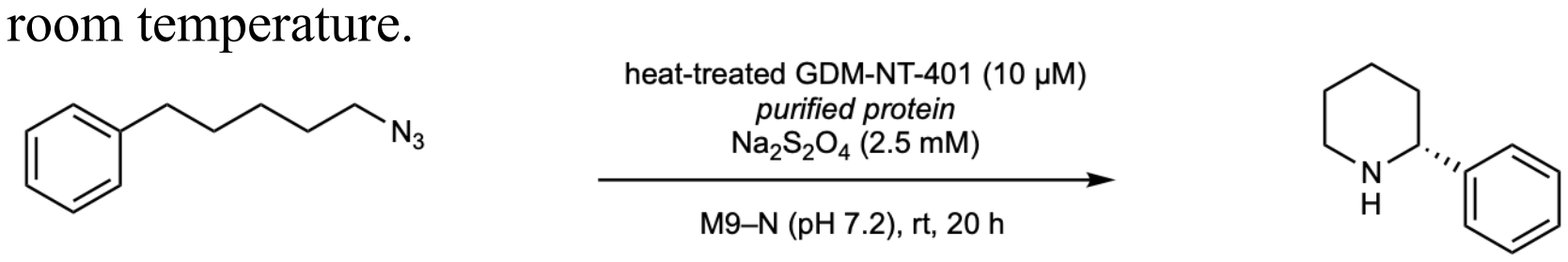

1a (5.0 mM) **2a**

Table S21. T_{50} measurement for **GDM-NT-401**

Temp. (°C)	PDT Rxn 1	IS Rxn 1	PDT Rxn 2	IS Rxn 2	Average Yield (%)	Normalized Yield	STDEV
4	1497.2	4937.1	1563.6	4804.8	62	1.00	0.050
29.8	1628.7	4910.7	1596.3	4824.4	65	1.05	0.002
35.1	1488.5	4778.9	1550.4	4851.3	62	1.00	0.018
39.2	1578.4	4892.5	1594.7	4852.9	64	1.04	0.013
46.9	1601.6	4866.7	1600.6	4854.6	65	1.05	0.001
54.7	1496.0	4828.1	1541.7	4767.6	62	1.01	0.030
59.6	1714.3	4804.4	1664.3	4637.5	70	1.14	0.005
60.4	1620.6	4872.4	1636.0	4884.1	66	1.06	0.005
65	1704.3	4865.6	1730.8	4869.0	70	1.12	0.012
76.2	1693.8	4872.7	1742.8	4936.9	69	1.11	0.012

81.4	1675.5	4850.5	1727.1	4876.4	69	1.11	0.020
85	1665.7	4840.6	1690.2	4781.4	69	1.11	0.021

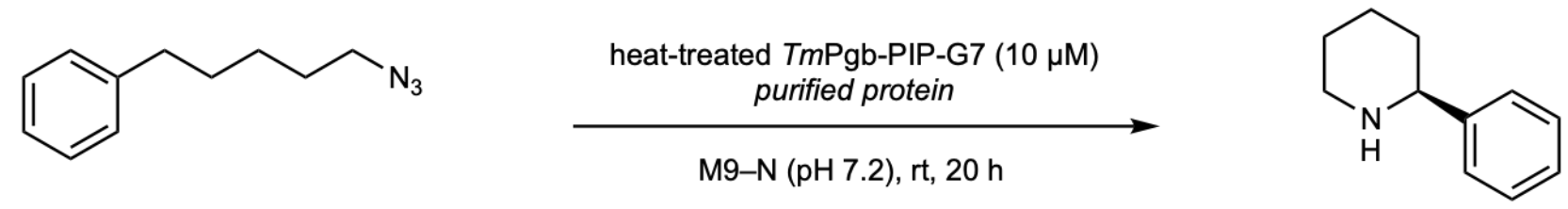

1a (5.0 mM) **2a**

Table S22. T_{50} measurement for ***Tm*Pgb-PIP-G7**

Temp. (°C)	PDT ion count Rxn 1	PDT ion count Rxn 2	PDT ion count average	Normalized Yield	STDEV
4	424219.2	481448.2	452833.7	1.00	0.089
29.8	380428.5	427653.5	404041.0	0.89	0.074
32.2	374280.8	445751.5	410016.2	0.91	0.112
39.2	393659.6	406688.8	400174.2	0.88	0.020
46.9	389157.2	388710.7	388933.9	0.86	0.001
54.7	378291.2	390006.4	384148.8	0.85	0.018
57.7	388774.5	436880.8	412827.6	0.91	0.075
59.6	383348.1	391706.5	387527.3	0.86	0.013
60.4	338108.4	390896.8	364502.6	0.80	0.082
65	168664.4	184524.0	176594.2	0.39	0.025
66.7	141247.3	153473.6	147360.5	0.33	0.019
68.6	51971.4	50511.6	51241.5	0.11	0.002
71.2	6431.4	7807.4	7119.4	0.02	0.002
76.2	trace	trace	trace	trace	NA
81.4	trace	trace	trace	trace	NA
85	trace	trace	trace	trace	NA

Part 8: Third round of design using refined motifs.

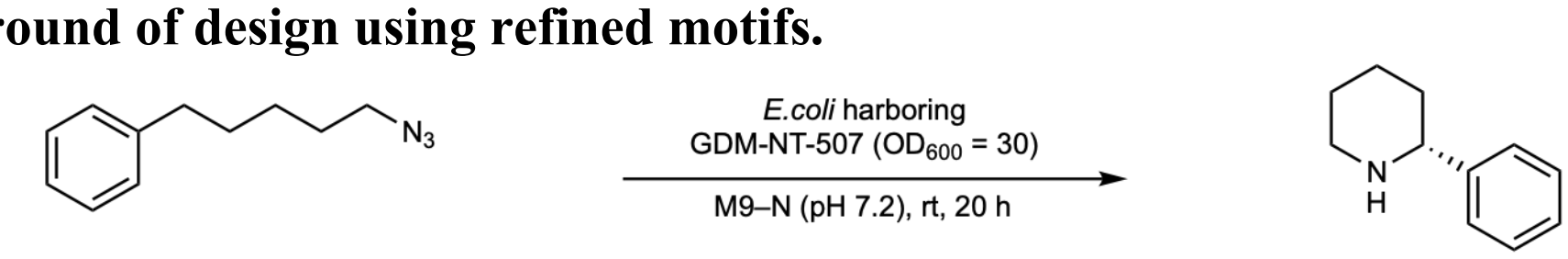

1a **2a**

Reaction performed **with GDM-NT-507** (whole *E.coli* cell with 1.8 μM heme loading)

Entry	Product	IS	P/IS	Yield (%)	Average (%)	STDEV	Average TTN
1	483.3	5527.7	0.087	17.2			
2	522.0	5829.8	0.090	17.6	17.0	0.8	470
3	471.9	5808.5	0.081	16.0			

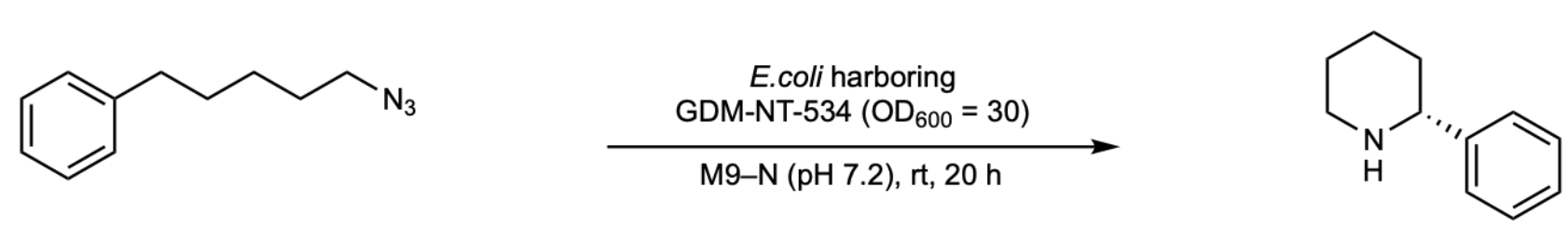

1a **2a**

Reaction performed **with GDM-NT-534** (whole *E.coli* cell with 1.8 μM heme loading)

Entry	Product	IS	P/IS	Yield (%)	Average (%)	STDEV	Average TTN
1	392.2	5679.2	0.069	13.6			
2	523.6	5803.6	0.090	17.8	15.5	2.1	420
3	448.9	5871.5	0.076	15.1			

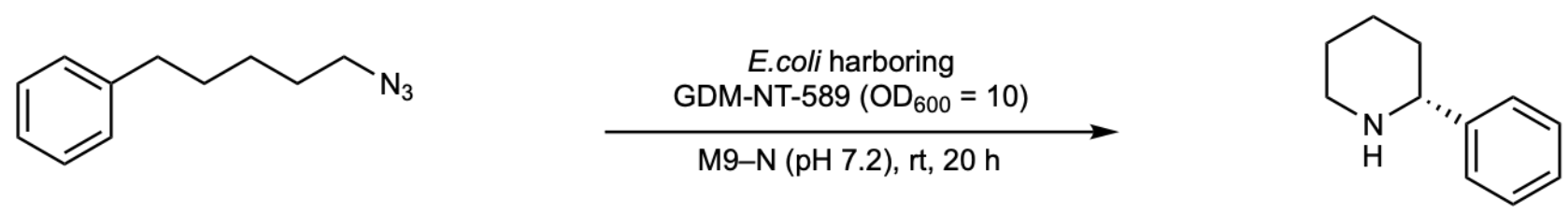

1a 2a

Reaction performed **with GDM-NT-589** (whole *E.coli* cell with OD_{600} = 10)
This variant was only able to express at low cell density likely due to relatively larger protein size.

Entry	Product	IS	P/IS	Yield (%)	Average (%)	STDEV	Average TTN
1	499.2	5201.1	0.096	18.9			
2	428.9	5267.5	0.081	16.0	17.2	1.4	N.A.
3	463.4	5141.6	0.090	17.8			
4	442.6	5388.1	0.082	16.2			

Part 9: Substrate scope analysis.

Analytical reaction yield obtained with *E.coli* harboring **GDM-NT-401** (OD_{600} = 30, 2.8 µM heme loading) or purified ***Tm*Pgb-PIP-G7-5448** (20 µM heme loading). All yields were calculated using a calibration curve measured on UV spectrum (210 nm or 254 nm) with 1,2,3-trimethoxybenzene as internal standard. Reactions with <1% yields were omitted.

Calibration curve and data analysis for 2a and 2b

2a and **2b** were measured using the same calibration curve.

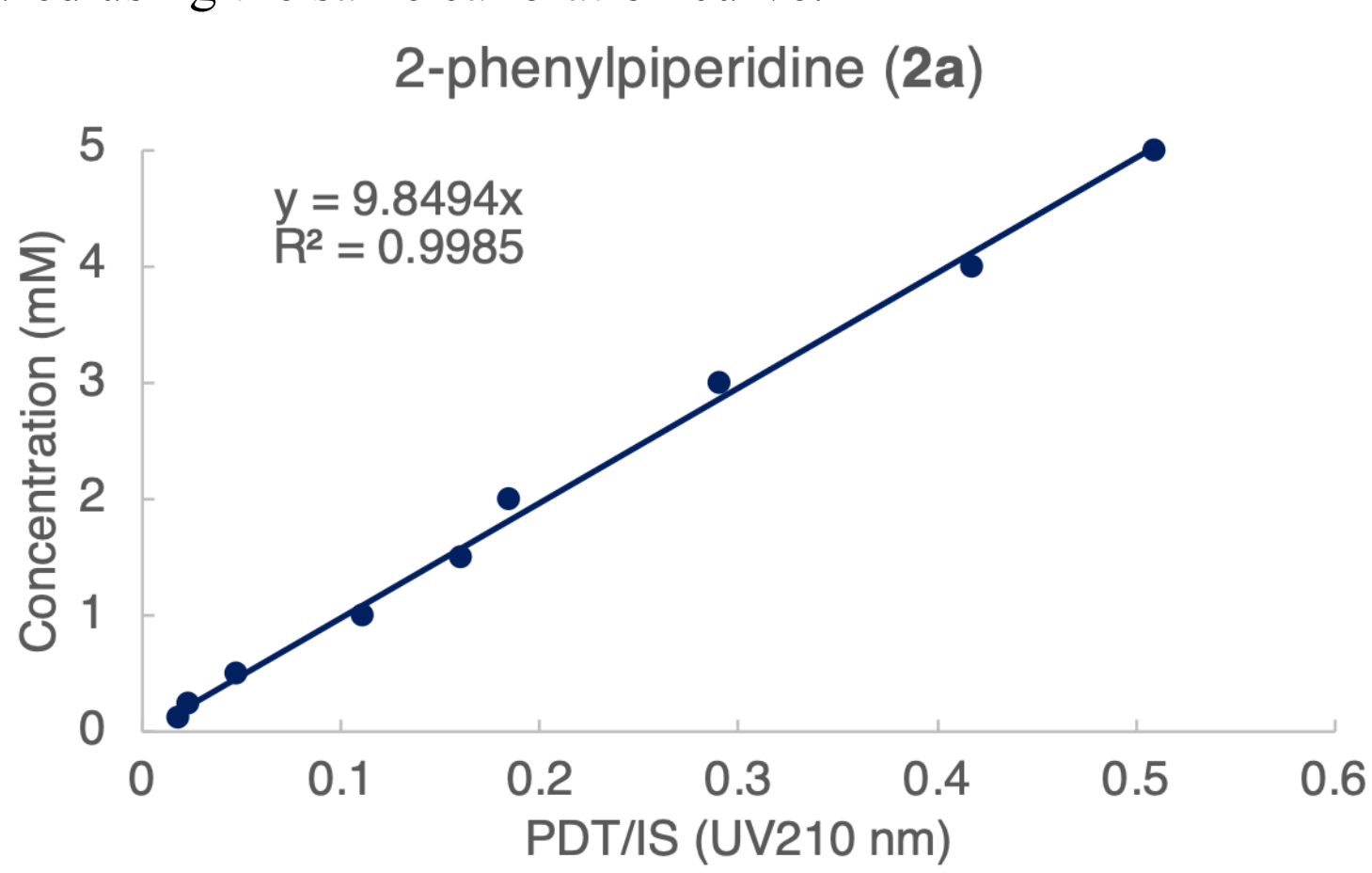

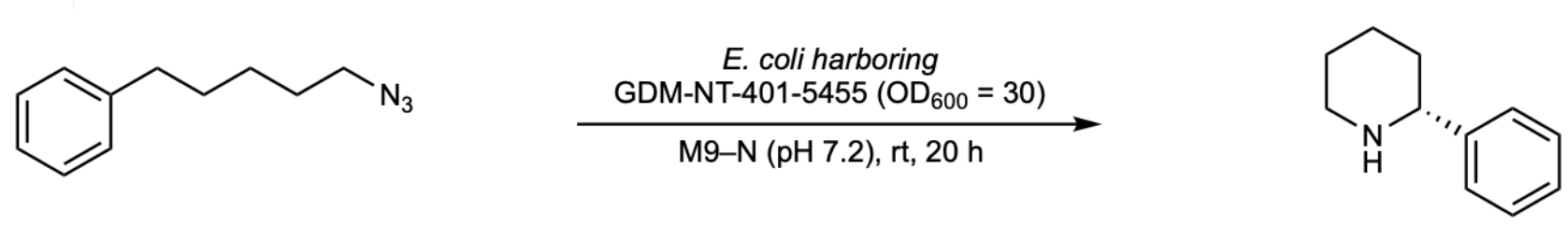

1a 2a

Reaction performed **with GDM-NT-401-5455** (whole *E.coli* cell with 2.8 μM heme loading)

Entry	Product	IS	P/IS	Yield (%)	Average (%)	STDEV	Average TTN
1	1724.8	4824.8	0.36	70.4			
2	1564.7	4682.5	0.33	65.8	66	3.7	1200
3	1533.3	4781.9	0.32	63.1			

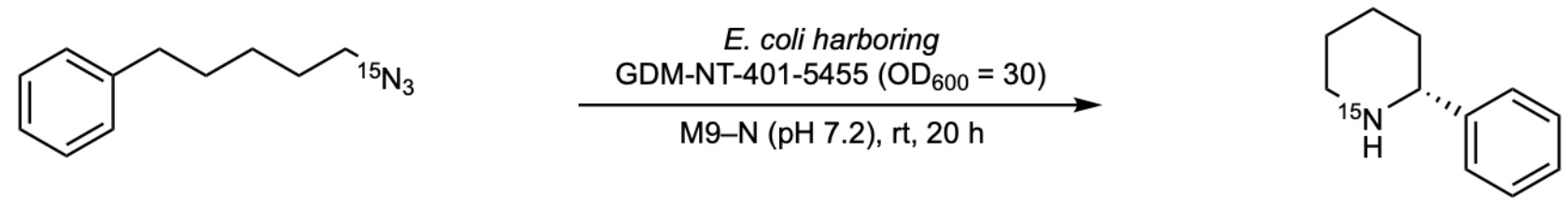

1a **2b**

Reaction performed **with GDM-NT-401-5455** (whole *E.coli* cell with 2.8 μM heme loading)

Entry	Product	IS	P/IS	Yield (%)	Average (%)	STDEV	Average TTN
1	1620.5	4695.6	0.35	68.0			
2	1348.2	4716.3	0.29	56.3	59	7.9	1100
3	1255.1	4651.3	0.27	53.2			

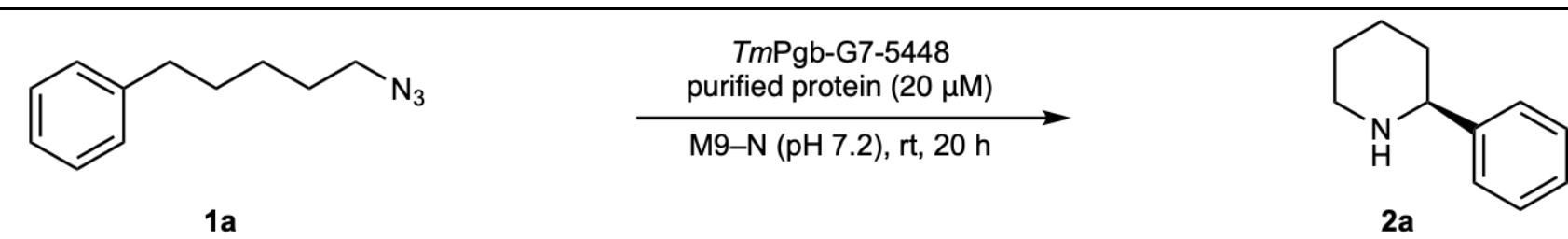

1a **2a**

Reaction performed **with *Tm*Pgb-G7-5448** (purified protein with 20 μM heme loading)

Entry	Product	IS	P/IS	Yield (%)	Average (%)	STDEV	Average TTN
1	303.6	4736.1	0.06	12.6			
2	276.4	4622.1	0.06	11.8	12	0.4	31
3	288.4	4568.7	0.06	12.4			

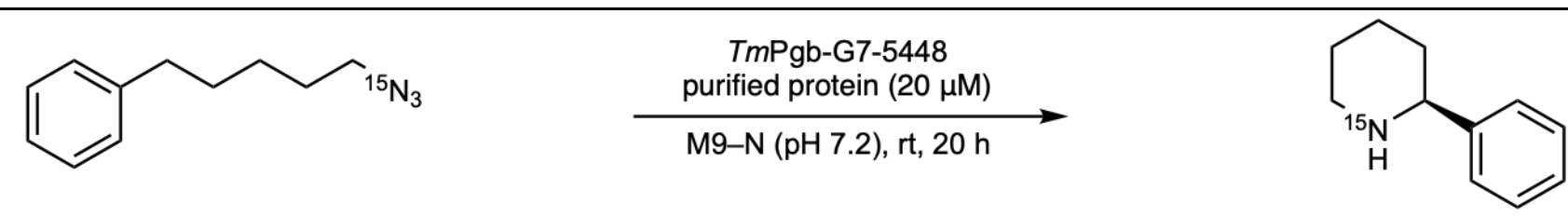

1a **2b**

Reaction performed **with *Tm*Pgb-G7-5448** (purified protein with 20 μM heme loading)

Entry	Product	IS	P/IS	Yield (%)	Average (%)	STDEV	Average TTN
1	295.8	6145.3	0.05	9.5			
2	337.6	6143.7	0.05	10.8	11	1.1	27
3	368.8	6202.4	0.06	11.7			

Calibration curve and data analysis for 2c

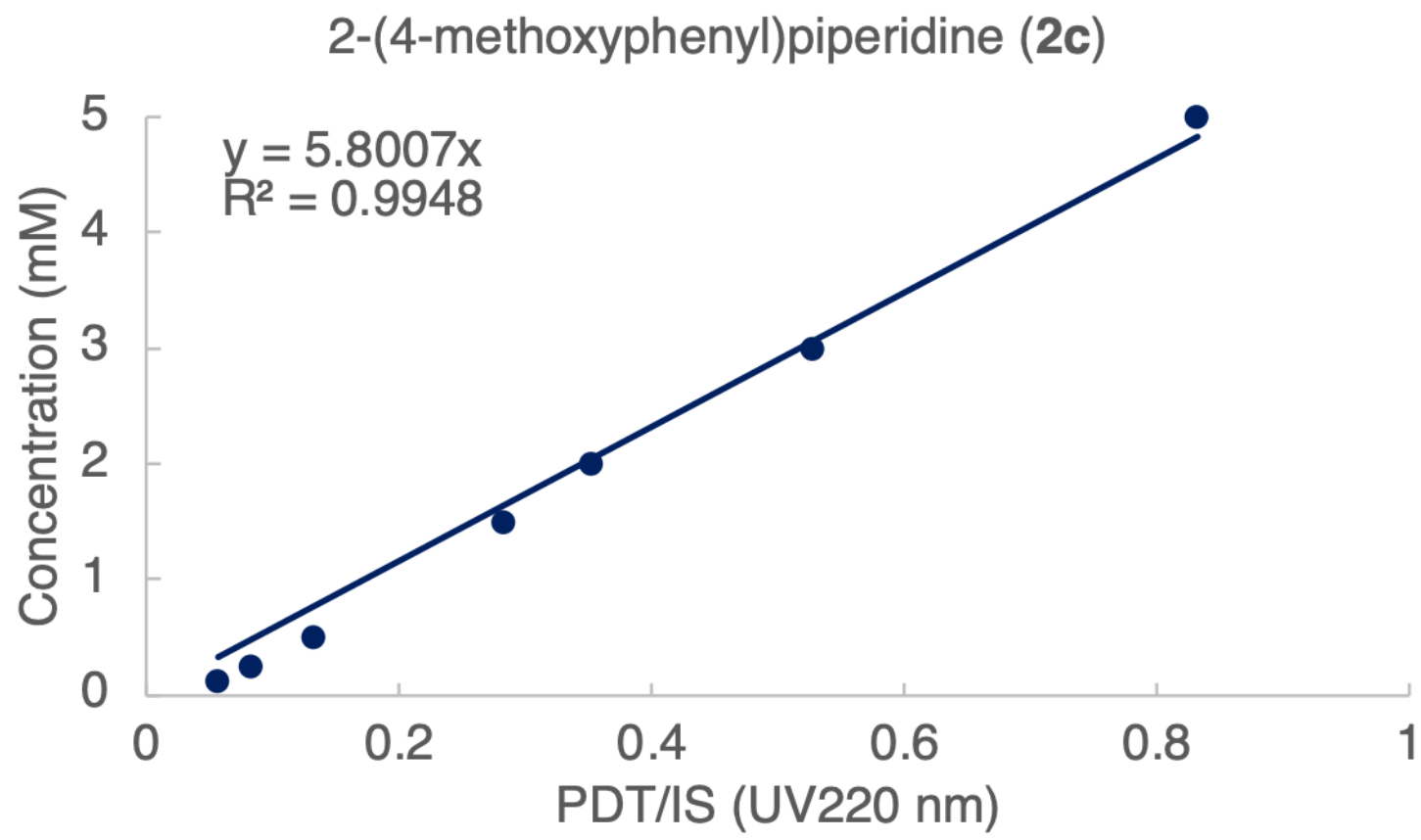

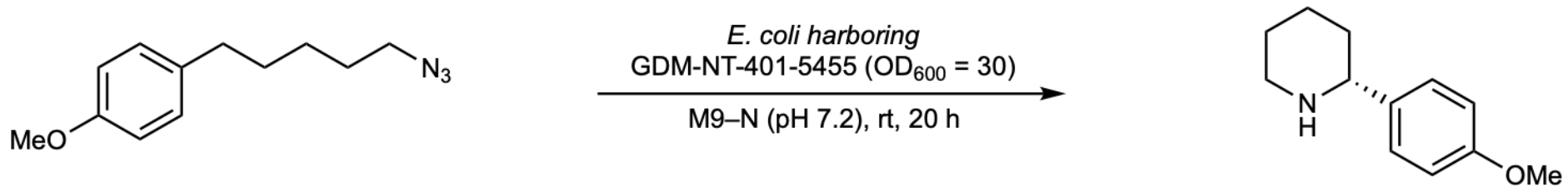

Reaction performed **with GDM-NT-401-5455** (whole *E.coli* cell with 2.8 μM heme loading)

Entry	Product	IS	P/IS	Yield (%)	Average (%)	STDEV	Average TTN
1	201.7	2602.4	0.08	9.0			
2	214.0	2732.6	0.08	9.1	9	0.6	168
3	235.1	2687.4	0.09	10.2			

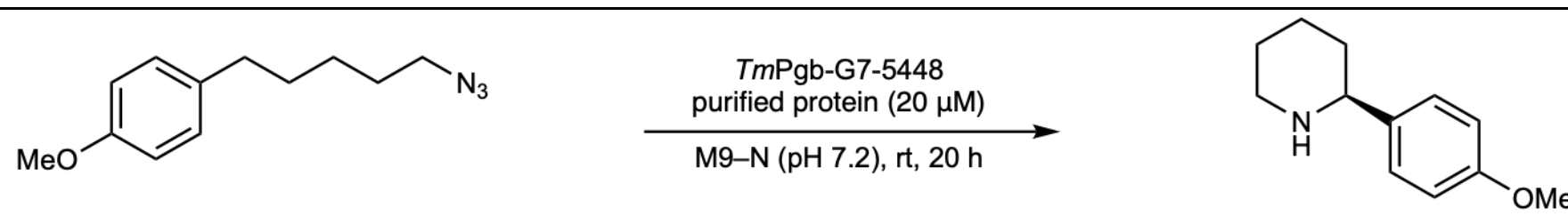

1c **2c**, <1% yield

Reaction performed **with *Tm*Pgb-G7-5448** (purified protein with 20 μM heme loading) furnished **2c** in <1% yield.

Calibration curve and data analysis for 2d

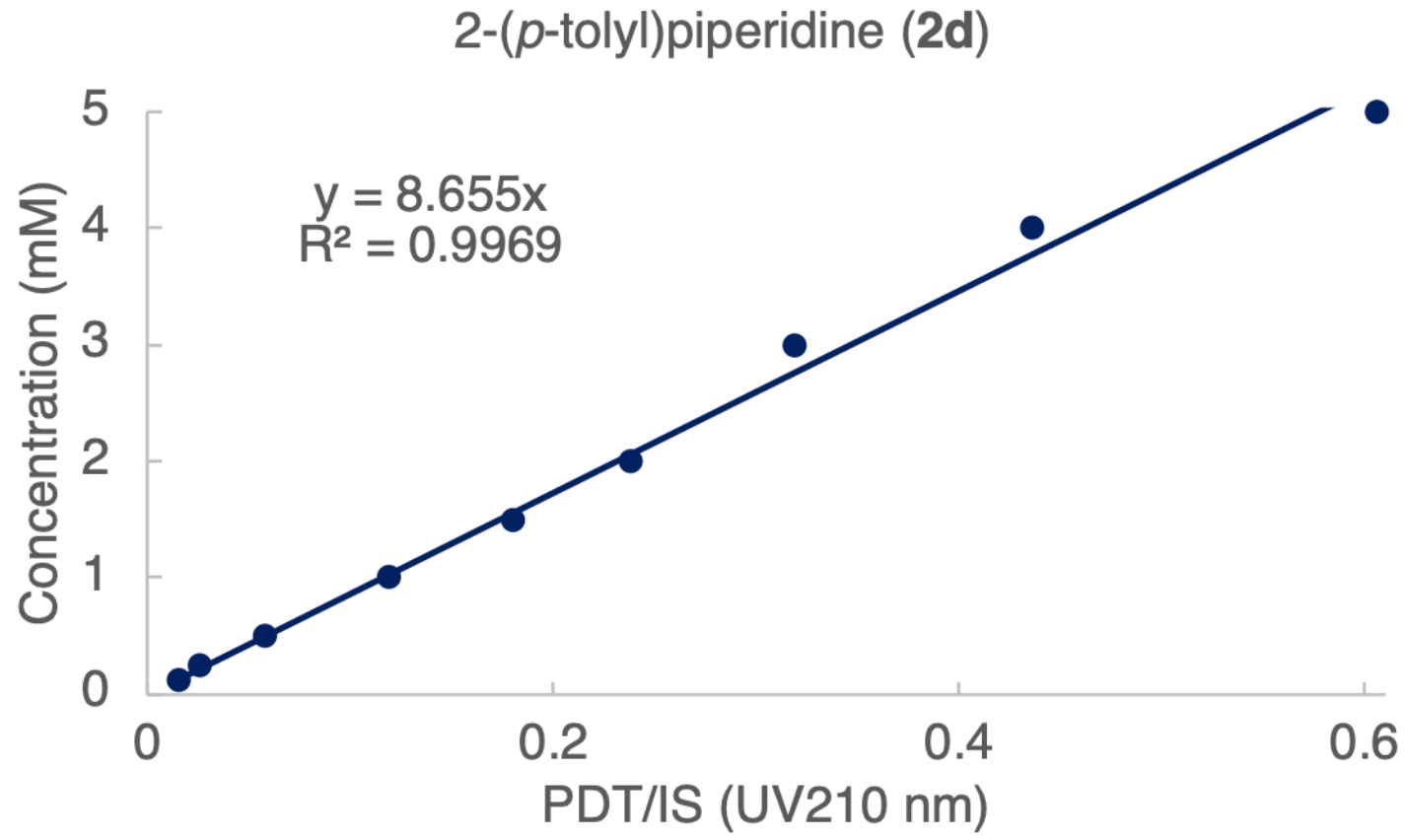

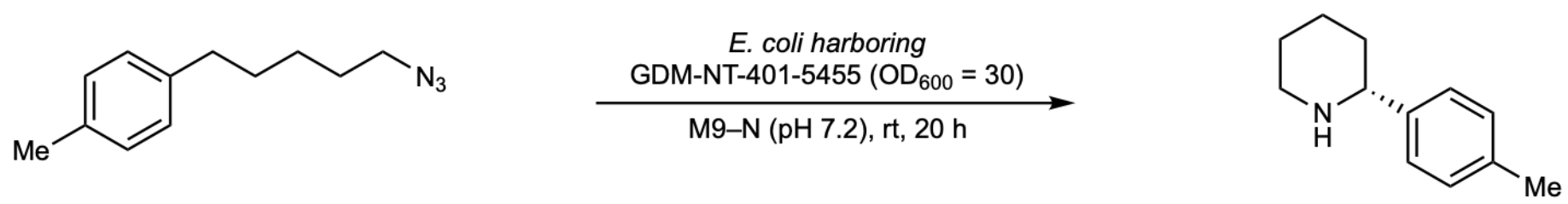

Reaction performed **with GDM-NT-401-5455** (whole *E.coli* cell with 2.8 μM heme loading)

Entry	Product	IS	P/IS	Yield (%)	Average (%)	STDEV	Average TTN
1	1430.3	4422.4	0.32	56.0			
2	1291.1	4407.5	0.29	50.7	52	3.8	926
3	1253.6	4446.2	0.28	48.8			

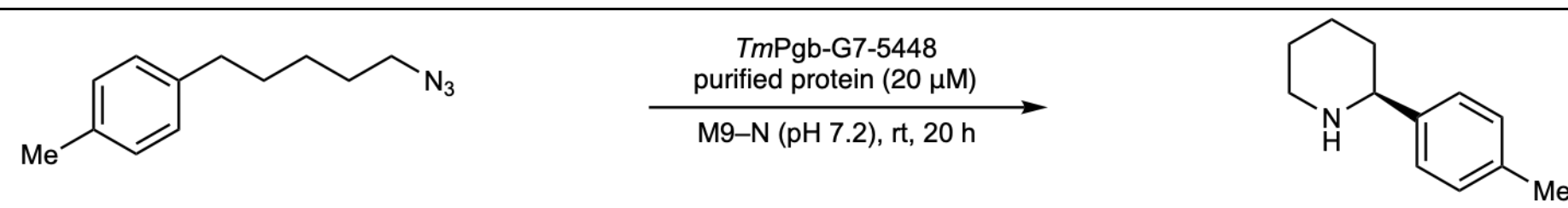

Reaction performed **with *Tm*Pgb-G7-5448** (purified protein)

Entry	Product	IS	P/IS	Yield (%)	Average (%)	STDEV	Average TTN
1	214.6	4291.7	0.05	8.7			
2	214.2	4459.5	0.05	8.3	9	0.2	21
3	215.9	4307.3	0.05	8.7			

Calibration curve and data analysis for 3e

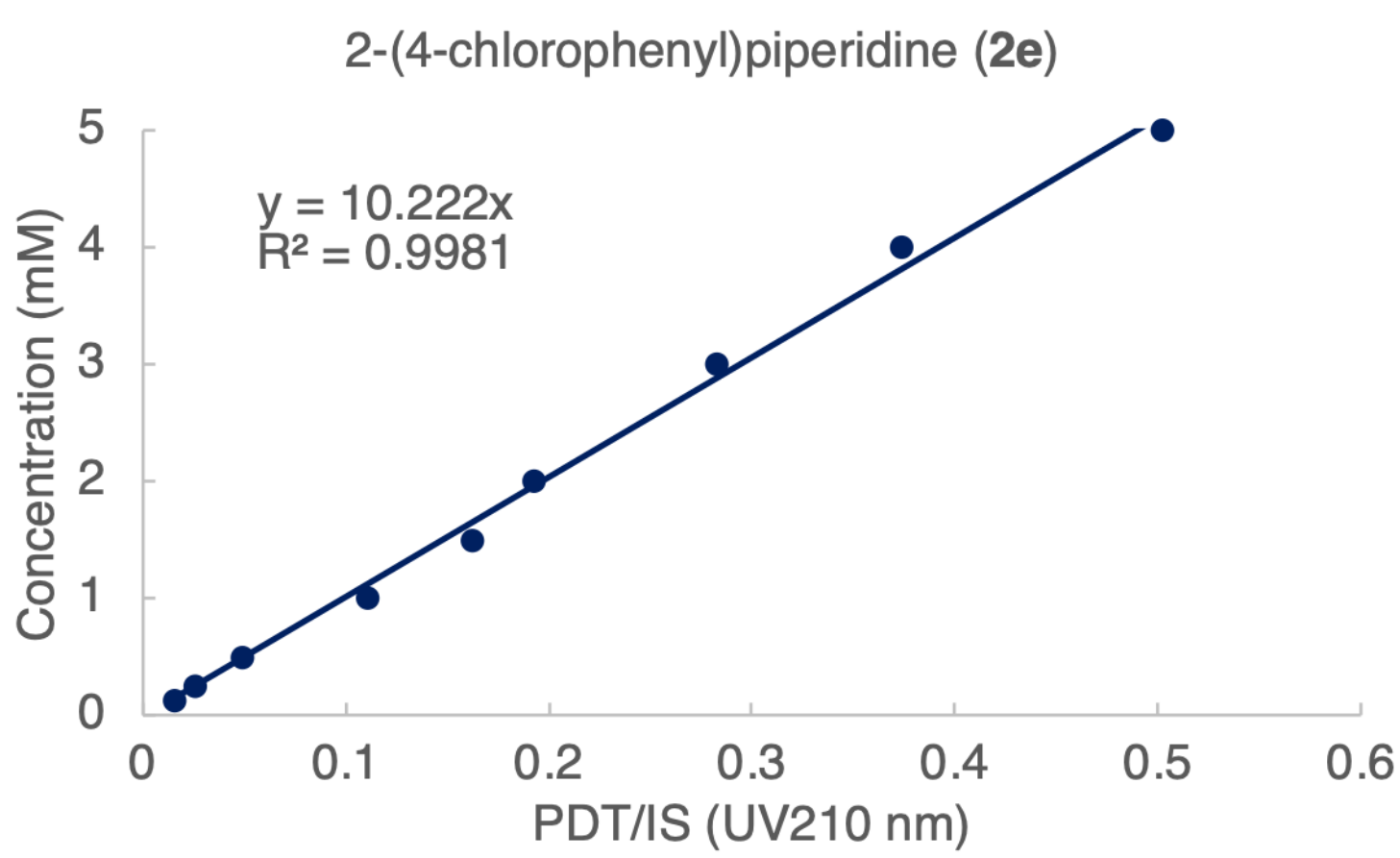

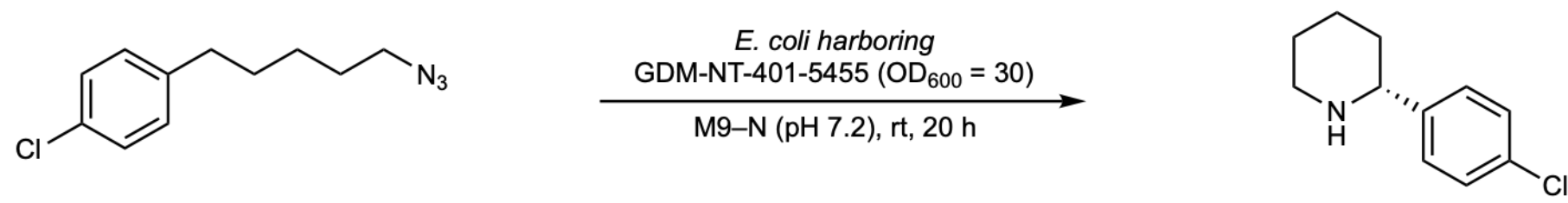

Reaction performed **with GDM-NT-401-5455** (whole *E.coli* cell with 2.8 μM heme loading)

Entry	Product	IS	P/IS	Yield (%)	Average (%)	STDEV	Average TTN
1	128.9	4697.0	0.03	5.6			
2	129.2	4743.7	0.03	5.6	5	0.6	94
3	105.5	4756.9	0.02	4.5			

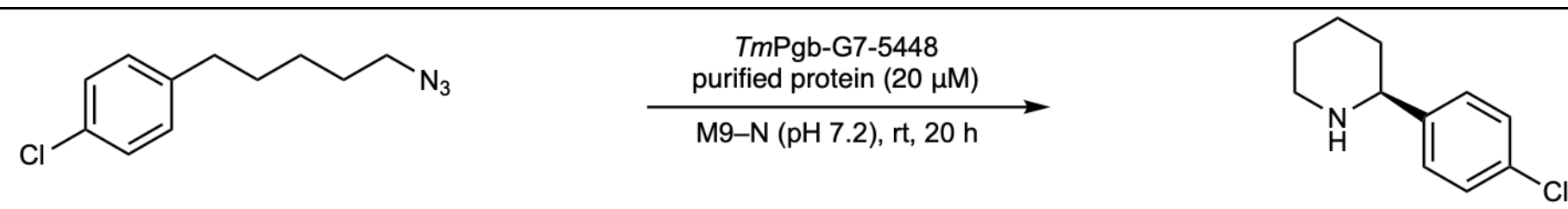

Reaction performed **with *Tm*Pgb-G7-5448** (purified protein)

Entry	Product	IS	P/IS	Yield (%)	Average (%)	STDEV	Average TTN
1	62.8	5797.3	0.011	2.2			
2	40.1	5726.6	0.007	1.4	2	0.5	4
3	37.1	5924.2	0.006	1.3			

Calibration curve and data analysis for 3f

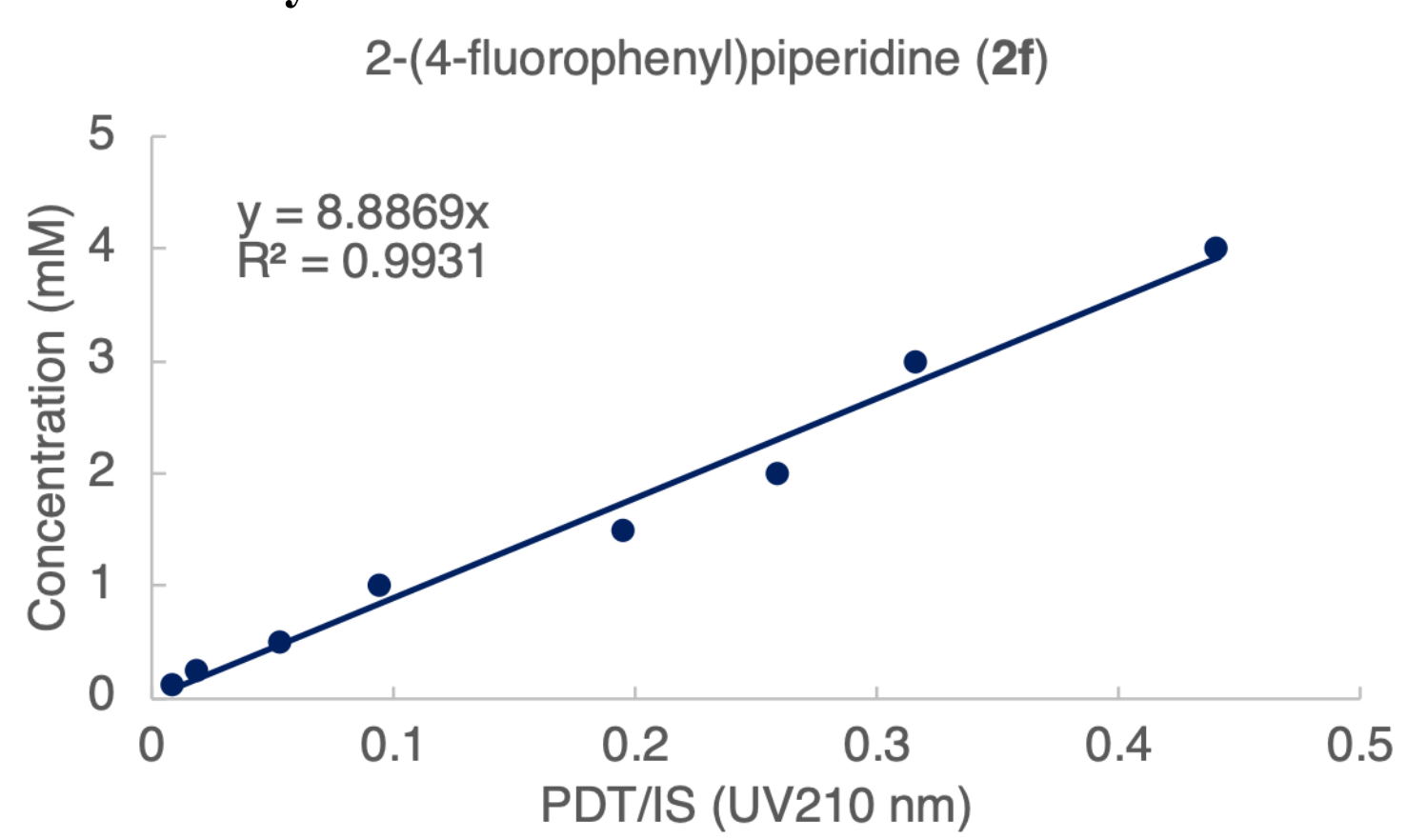

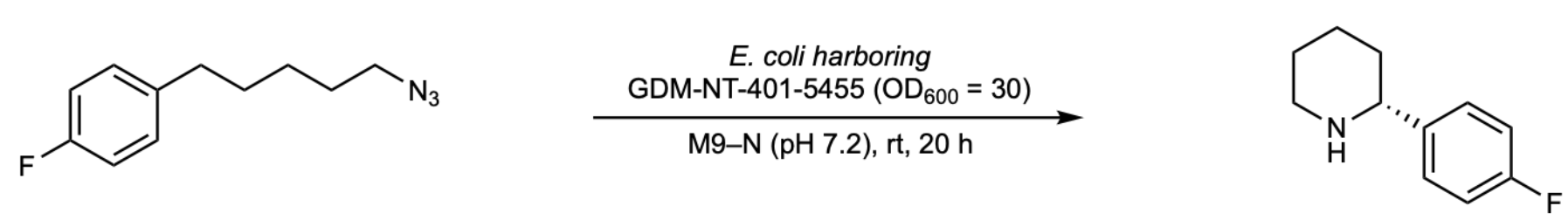

Reaction performed **with GDM-NT-401-5455** (whole *E.coli* cell with 2.8 μM heme loading)

Entry	Product	IS	P/IS	Yield (%)	Average (%)	STDEV	Average TTN
1	956.8	4743.6	0.20	35.9			
2	944.7	4760.1	0.20	35.3	34	2.1	600
3	844.2	4680.3	0.18	32.1			

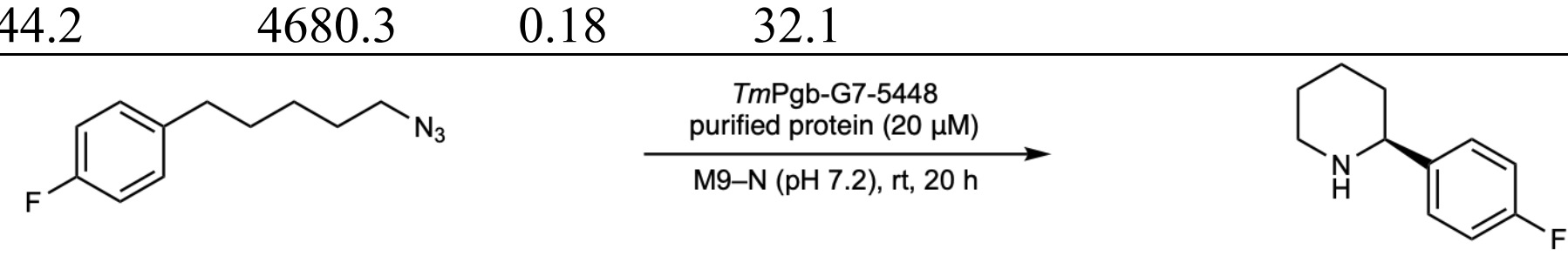

Reaction performed **with *Tm*Pgb-G7-5448** (purified protein)

Entry	Product	IS	P/IS	Yield (%)	Average (%)	STDEV	Average TTN
1	66.5	4774.2	0.014	2.5			
2	59.8	4471.9	0.013	2.4	3	0.4	7
3	80.5	4583.2	0.018	3.1			

Calibration curve and data analysis for 2g

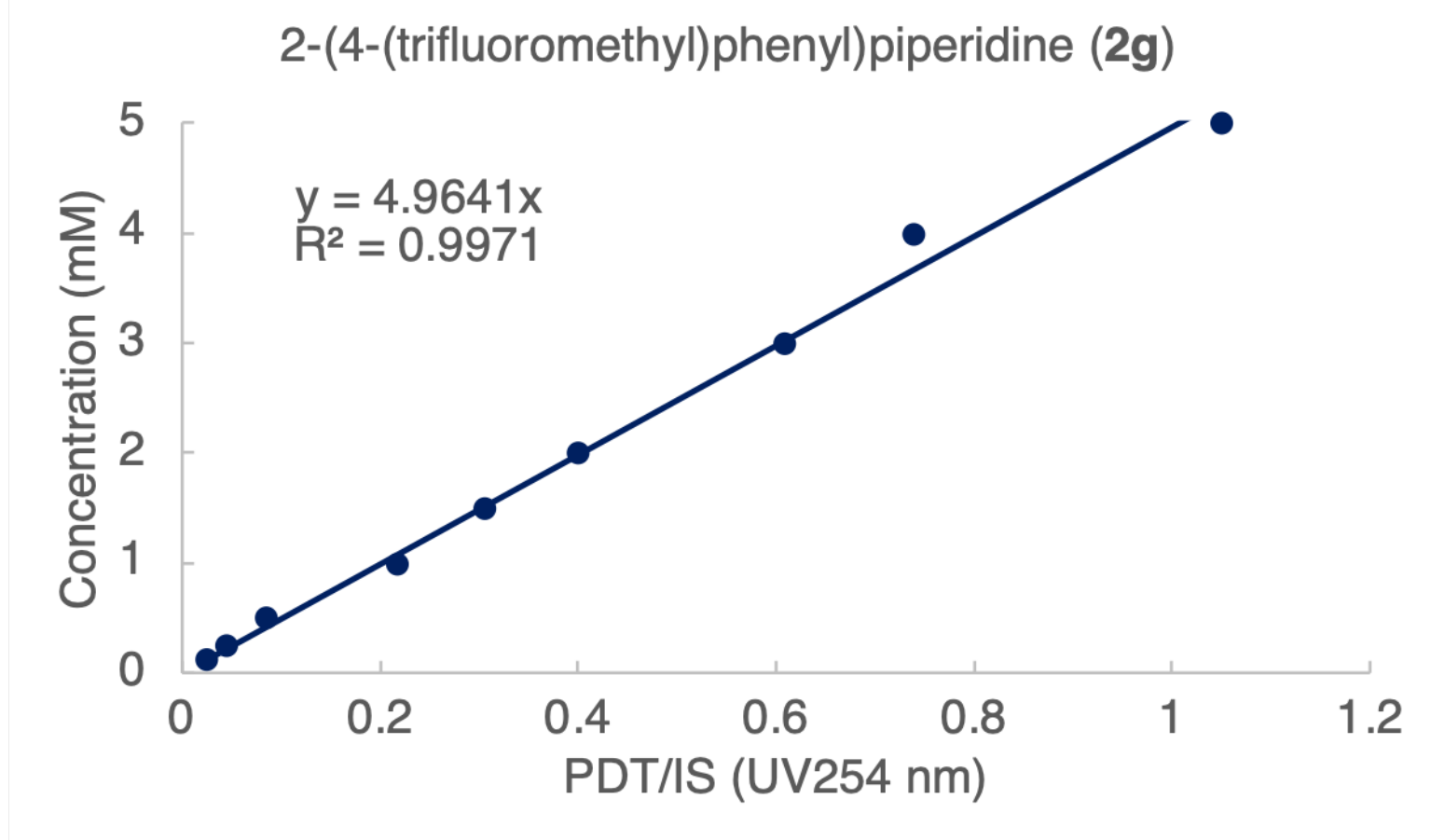

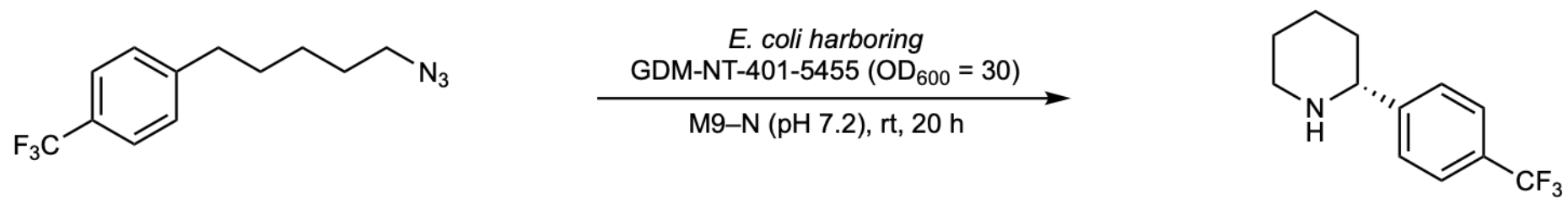

Reaction performed **with GDM-NT-401-5455** (whole *E.coli* cell with 2.8 μM heme loading)

Entry	Product	IS	P/IS	Yield (%)	Average (%)	STDEV	Average TTN
1	36.1	111.9	0.32	32.1			
2	34.0	118.1	0.29	28.6	30	1.7	539
3	35.4	117.8	0.30	29.8			

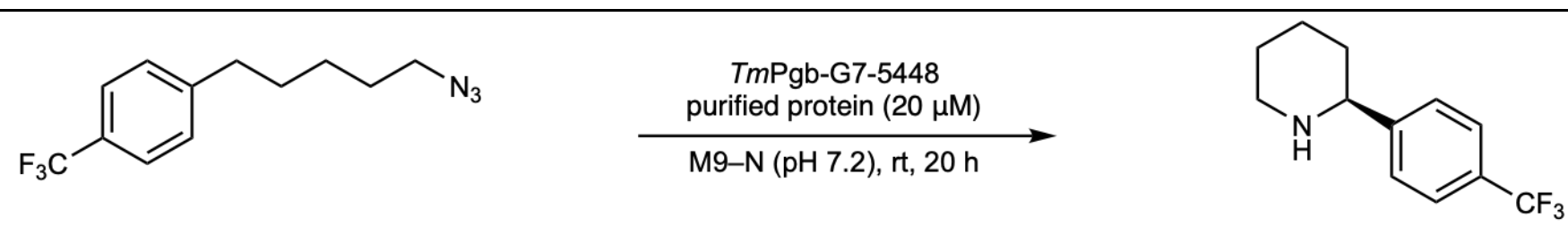

1g **2g**, <1% yield

Reaction performed **with *Tm*Pgb-G7-5448** (purified protein with 20 μM heme loading) furnished **2g** in <1% yield.

Calibration curve and data analysis for 2h

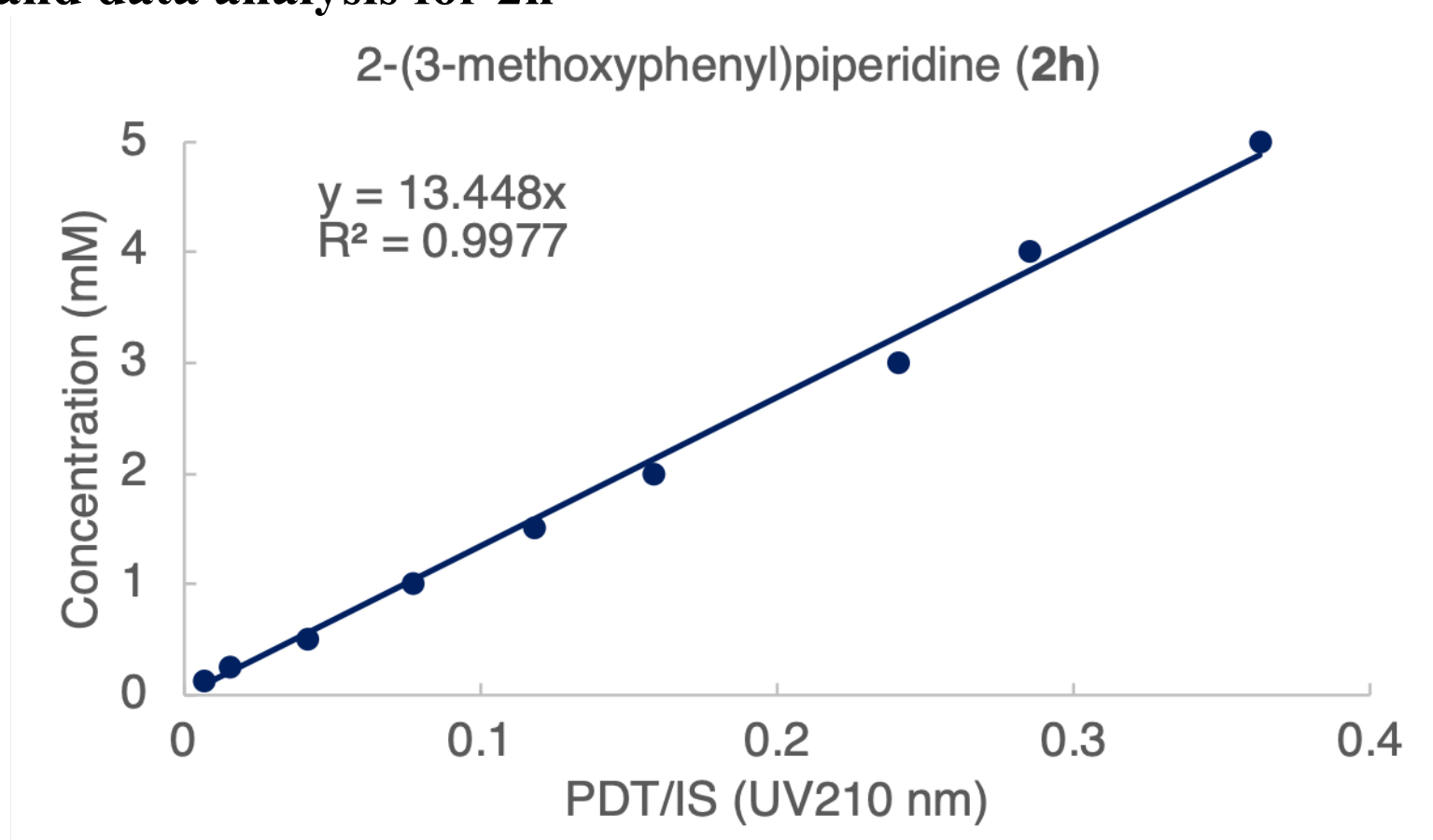

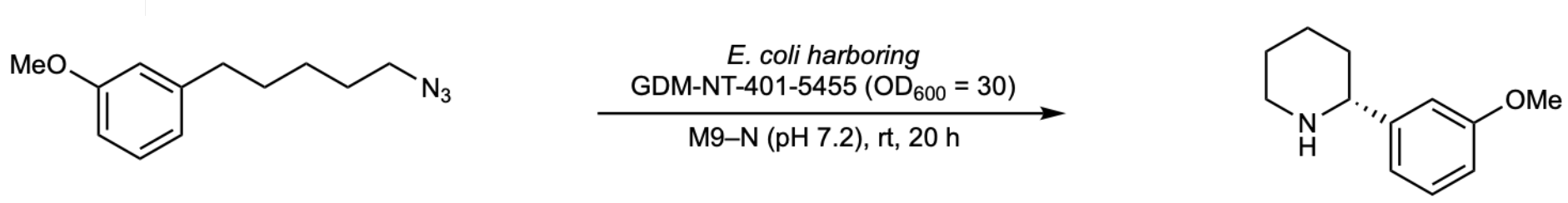

Reaction performed **with GDM-NT-401-5455** (whole *E.coli* cell with 2.8 μM heme loading)

Entry	Product	IS	P/IS	Yield (%)	Average (%)	STDEV	Average TTN
1	786.6	4754.6	0.17	44.5			
2	760.0	4680.6	0.16	43.7	46	3.3	800
3	883.0	4783.8	0.18	49.6			

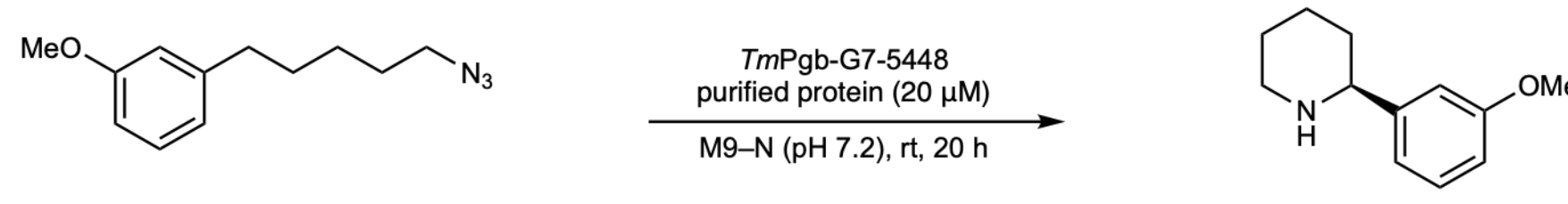

1h **2h**, <1% yield

Reaction performed **with *Tm*Pgb-G7-5448** (purified protein with 20 μM heme loading) furnished **2h** in <1% yield.

Calibration curve and data analysis for 2i

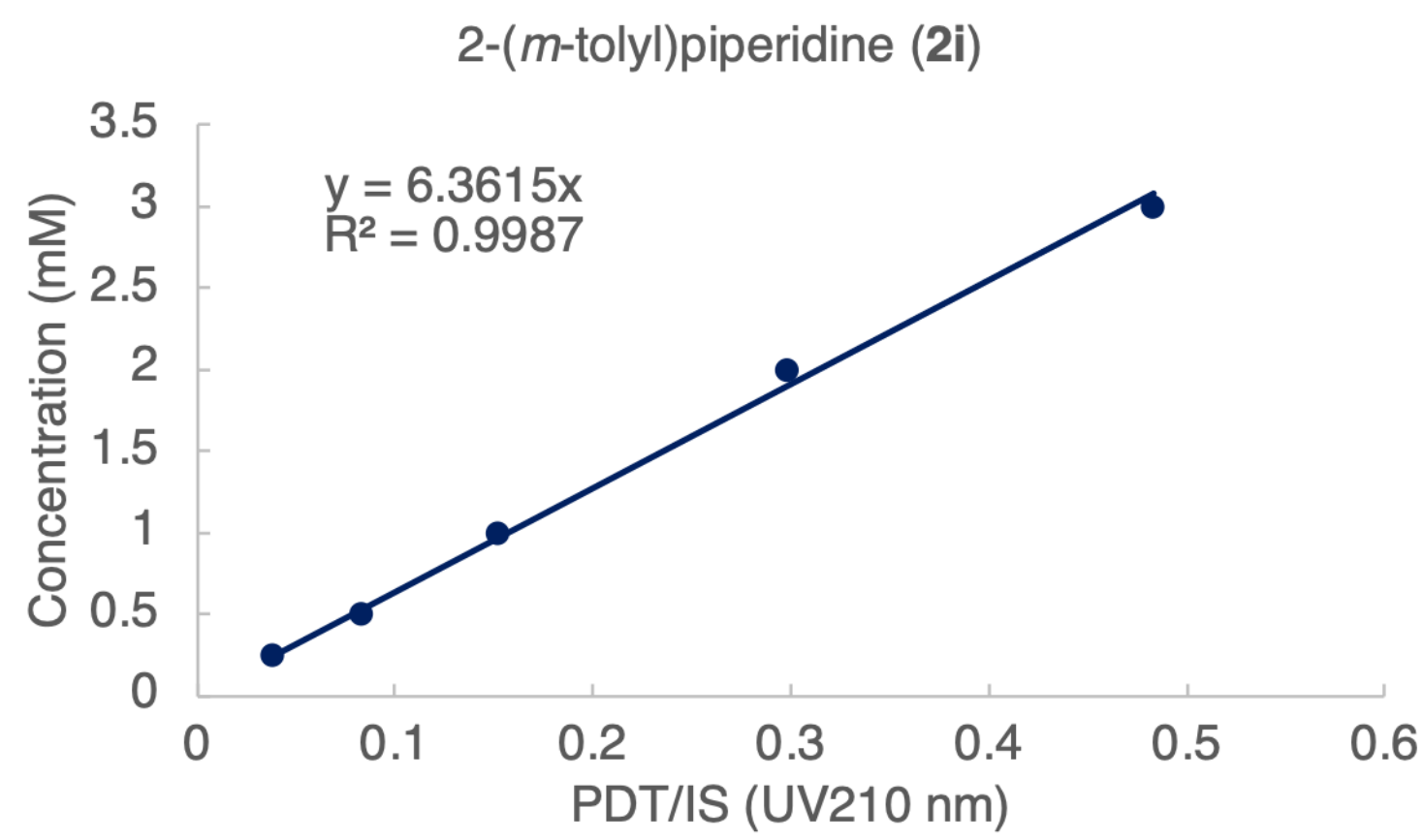

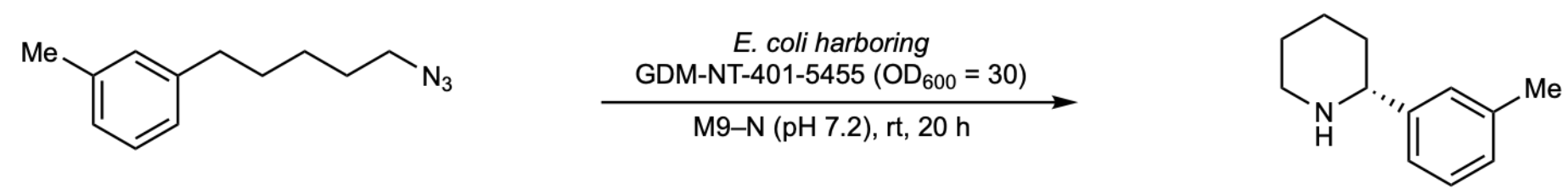

Reaction performed **with GDM-NT-401-5455** (whole *E.coli* cell with 2.8 μM heme loading)

Entry	Product	IS	P/IS	Yield (%)	Average (%)	STDEV	Average TTN
1	1162.5	4558.7	0.26	32.4			
2	1185.2	4345.1	0.27	34.7	33	1.3	594
3	1143.0	4451.1	0.26	32.7			

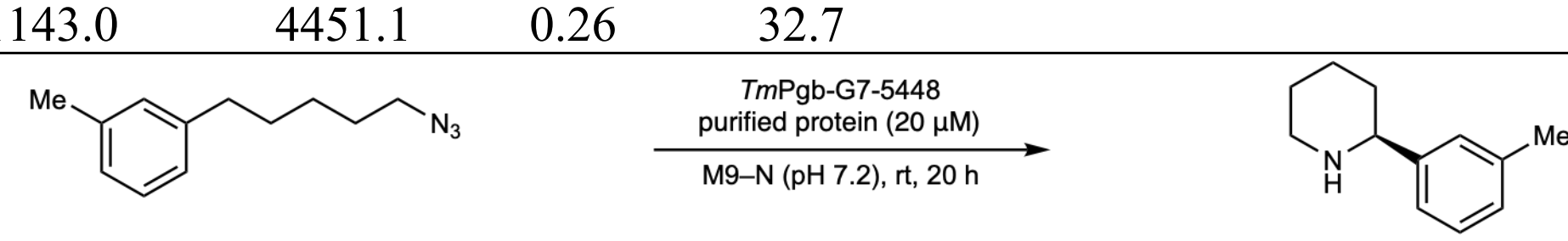

Reaction performed **with *Tm*Pgb-G7-5448** (purified protein)

Entry	Product	IS	P/IS	Yield (%)	Average (%)	STDEV	Average TTN
1	56.9	4748.3	0.012	1.5			
2	41.1	4396.5	0.009	1.2	1.3	0.2	3
3	44.0	4438.5	0.010	1.2			

Calibration curve and data analysis for 2j

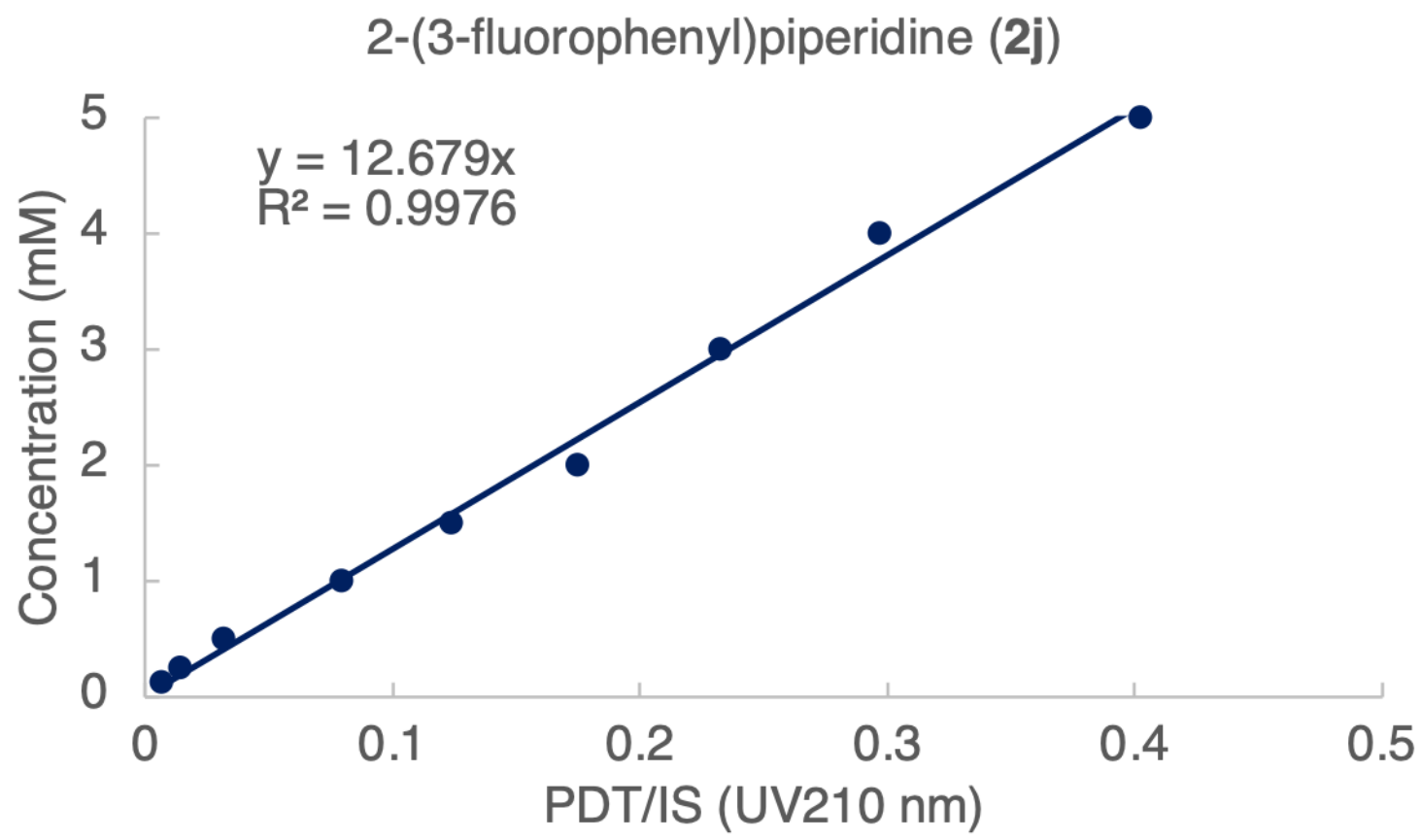

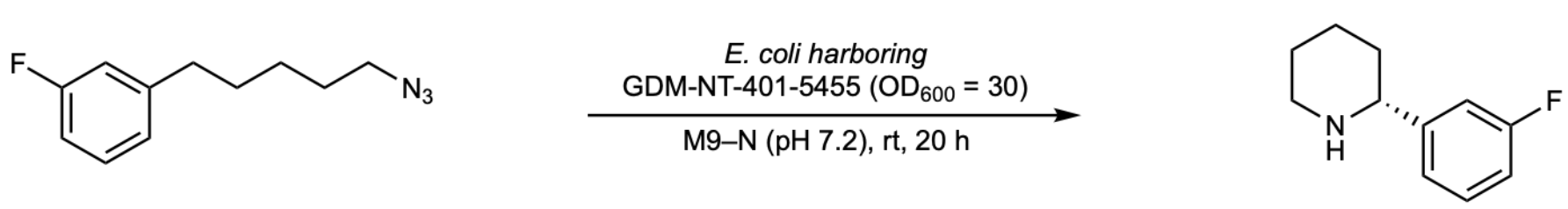

Reaction performed **with GDM-NT-401-5455** (whole *E.coli* cell with 2.8 μM heme loading)

Entry	Product	IS	P/IS	Yield (%)	Average (%)	STDEV	Average TTN
1	476.1	4533.5	0.11	26.6			
2	315.2	4402.9	0.07	18.2	21	4.8	376
3	305.3	4202.3	0.07	18.4			

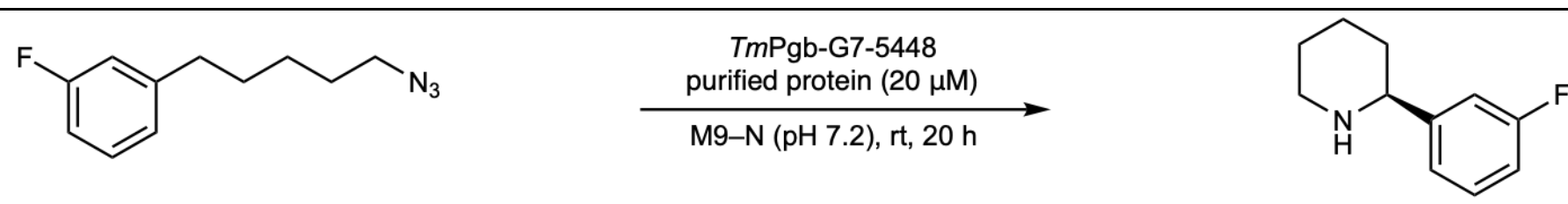

Reaction performed **with *Tm*Pgb-G7-5448** (purified protein)

Entry	Product	IS	P/IS	Yield (%)	Average (%)	STDEV	Average TTN
1	44.0	6296.9	0.007	1.8			
2	51.1	6303.4	0.008	2.1	2	0.1	5
3	49.0	6279.7	0.008	2.0			

Calibration curve and data analysis for 2k

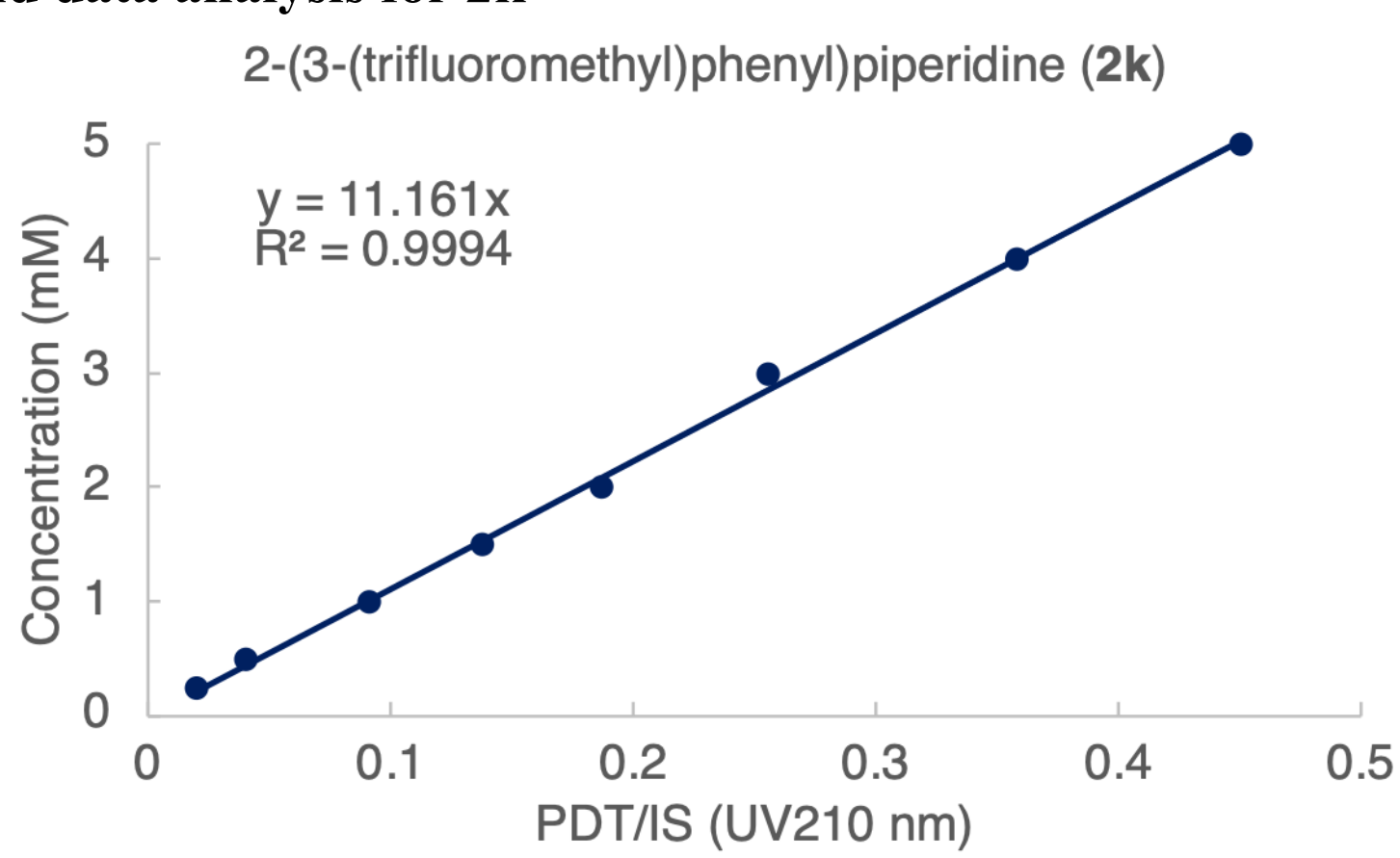

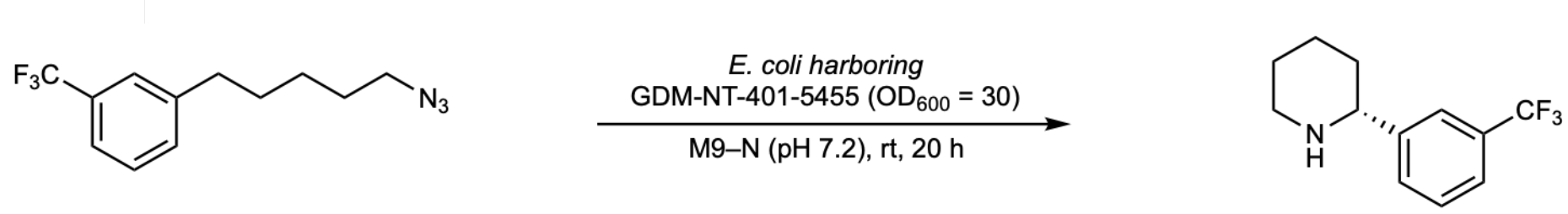

Reaction performed **with GDM-NT-401-5455** (whole *E.coli* cell with 2.8 µM heme loading)

Entry	Product	IS	P/IS	Yield (%)	Average (%)	STDEV	Average TTN
1	396.4	4568.3	0.09	19.4			
2	371.1	4482.0	0.08	18.5	19	0.6	334
3	380.5	4625.9	0.08	18.4			

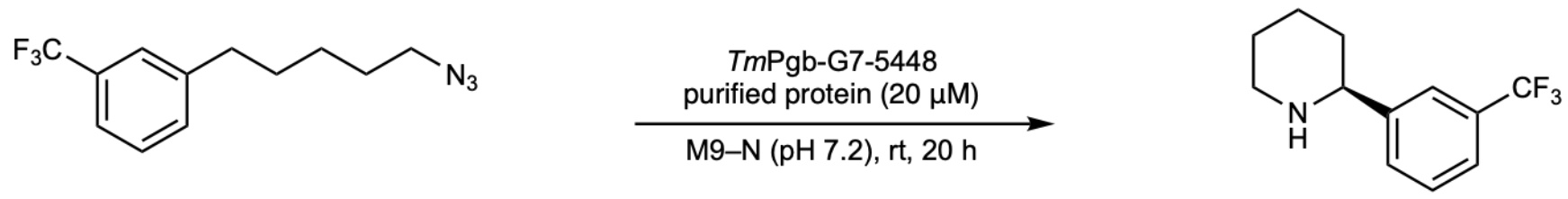

1k **2k**, <1% yield

Reaction performed **with *Tm*Pgb-G7-5448** (purified protein with 20 µM heme loading) furnished **2k** in <1% yield.

Calibration curve and data analysis for 2l

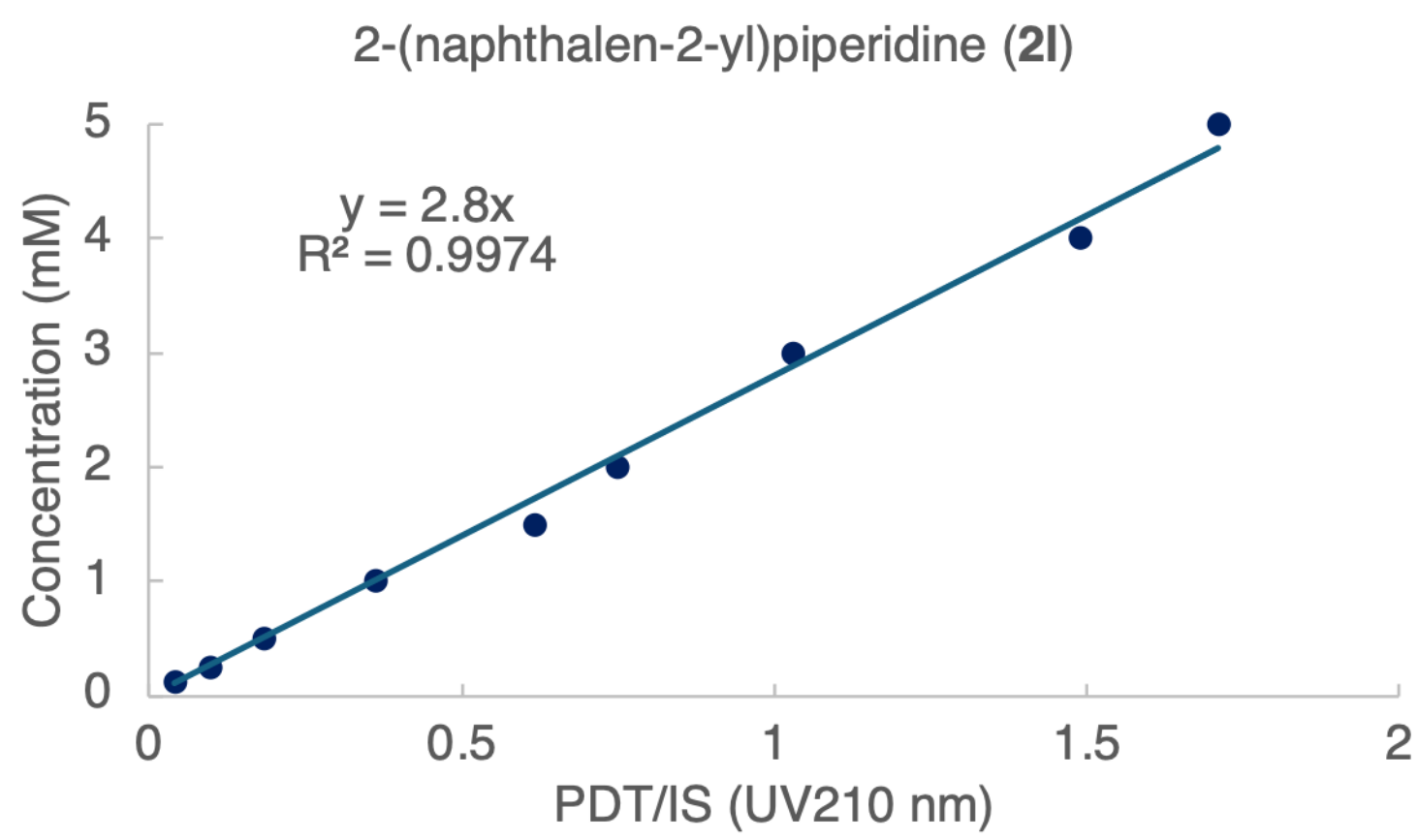

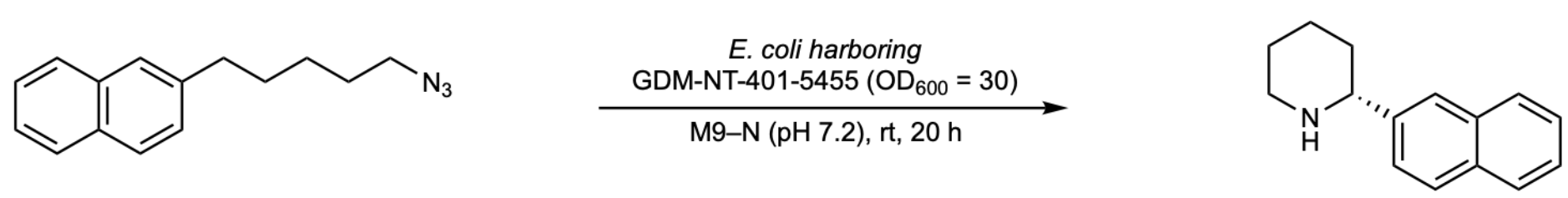

Reaction performed **with GDM-NT-401-5455** (whole *E.coli* cell with 2.8 µM heme loading)

Entry	Product	IS	P/IS	Yield (%)	Average (%)	STDEV	Average TTN
1	306.4	4721.5	0.06	3.6			
2	268.8	4632.7	0.06	3.2	3	0.2	60
3	259.9	4556.9	0.06	3.2			

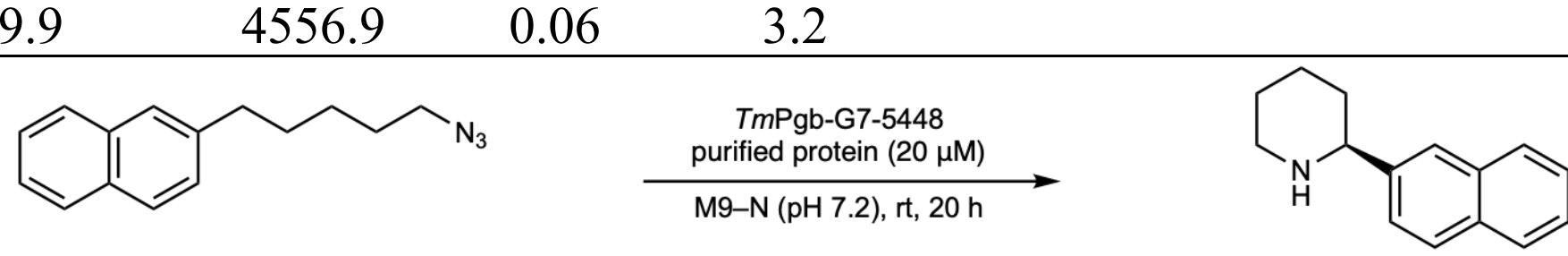

1l **2l**, <1% yield

Reaction performed **with *Tm*Pgb-G7-5448** (purified protein with 20 µM heme loading) furnished **2l** in <1% yield.

Calibration curve and data analysis for 2m

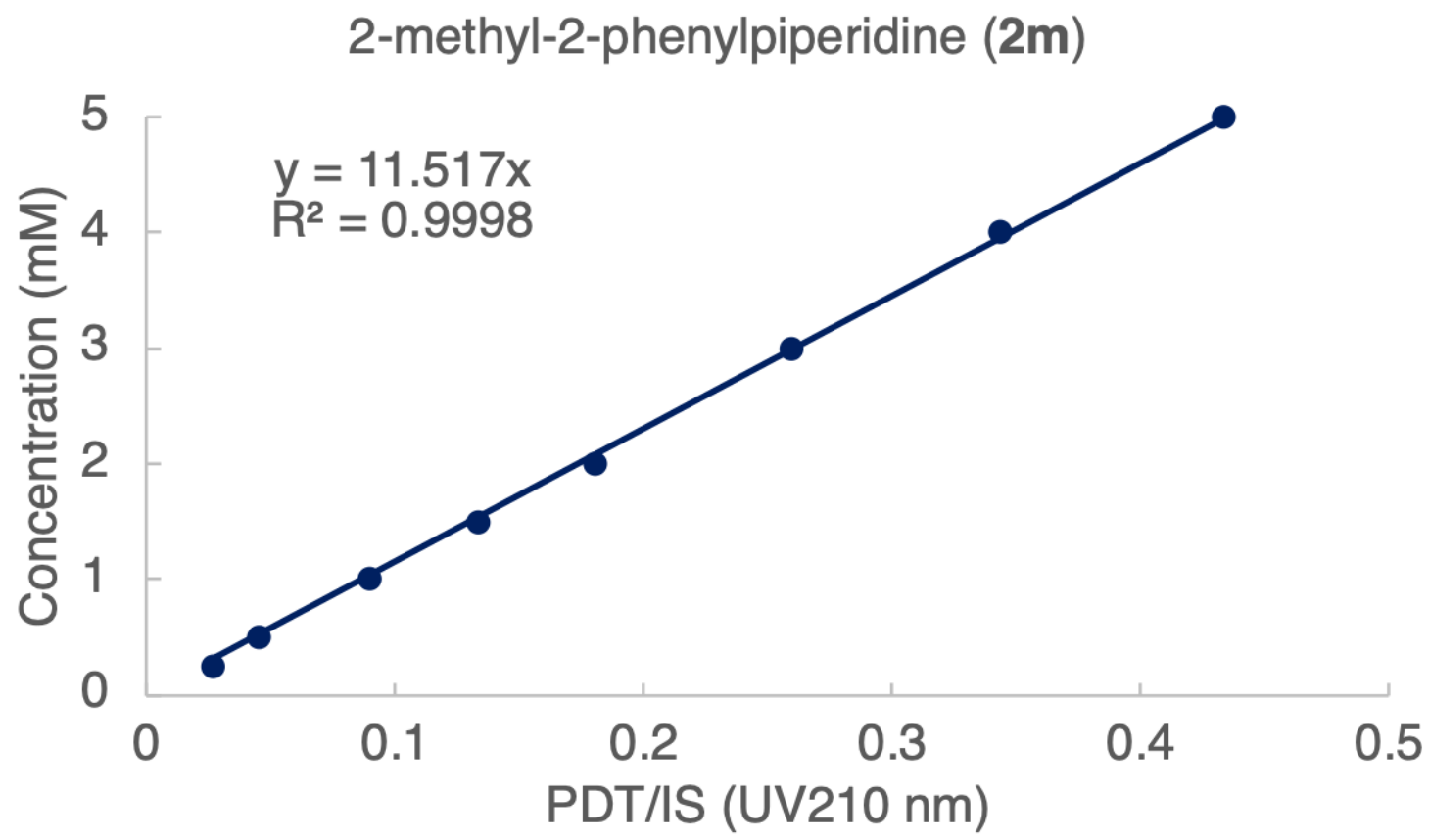

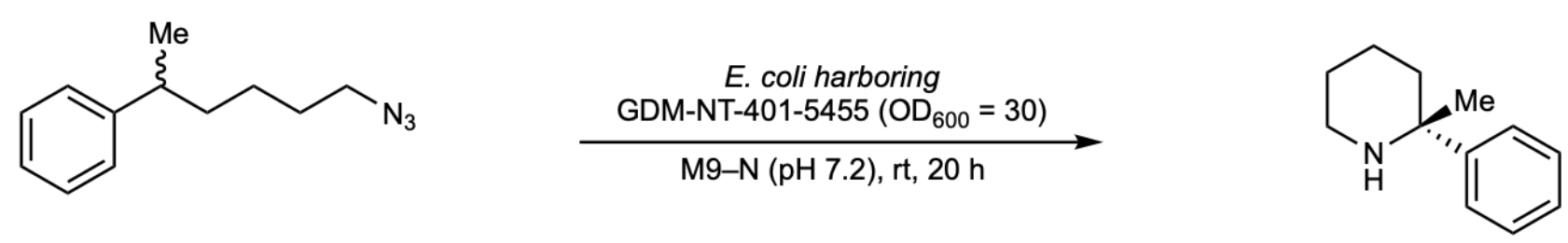

Reaction performed **with GDM-NT-401-5455** (whole *E.coli* cell with 2.8 μM heme loading)

Entry	Product	IS	P/IS	Yield (%)	Average (%)	STDEV	Average TTN
1	425.0	4341.0	0.10	22.6			
2	443.0	4359.8	0.10	23.4	23	0.5	407
3	426.9	4373.5	0.10	22.5			

Reaction performed **with GDM-NT-401-5455** (purified protein with 20 μM heme loading)

Entry	Product	IS	P/IS	Yield (%)	Average (%)	STDEV	Average TTN
1	853.2	4295.7	0.20	45.8			
2	851.8	4325.7	0.20	45.4	45	0.4	113
3	847.9	4336.6	0.20	45.0			

Calibration curve and data analysis for 2n

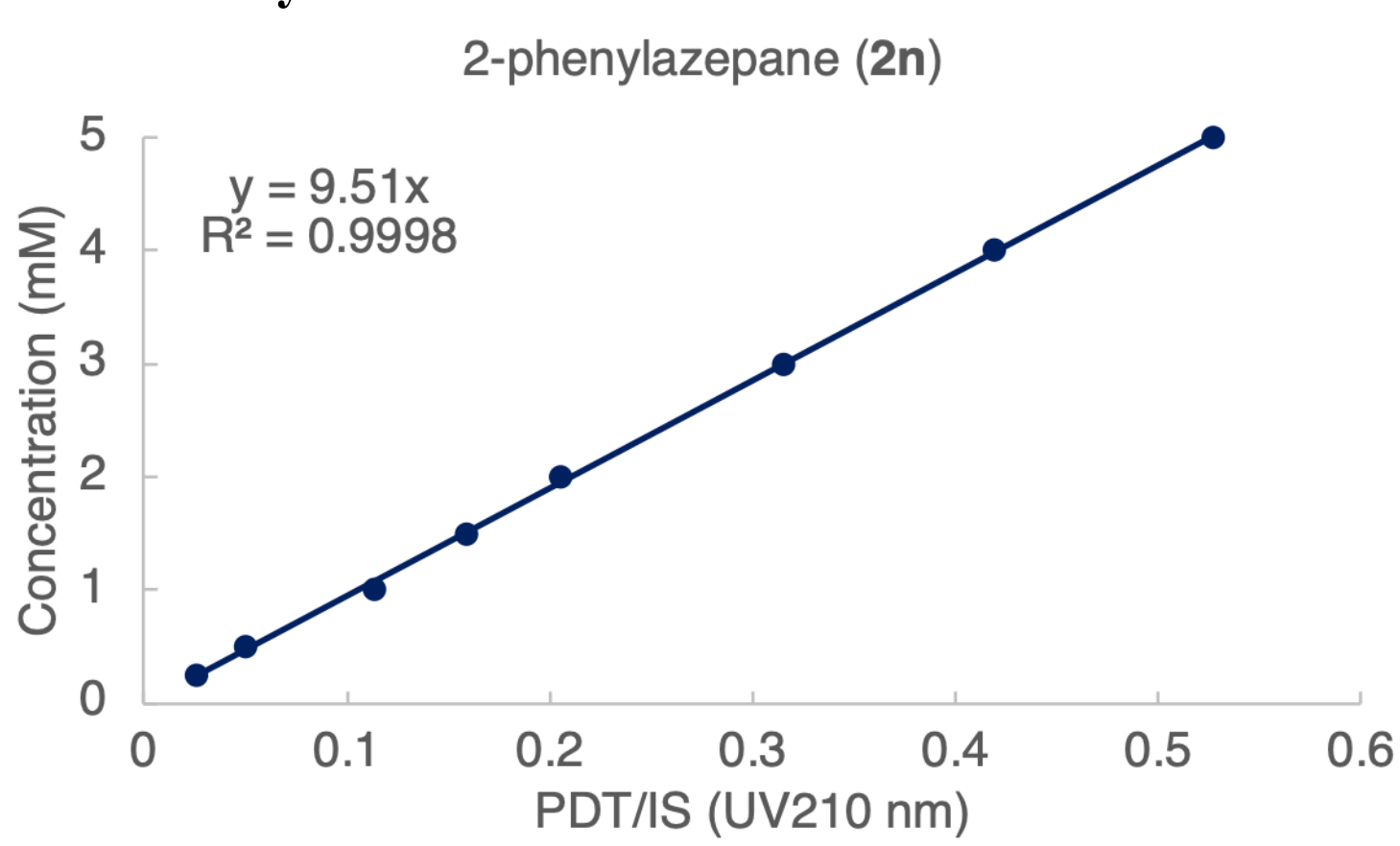

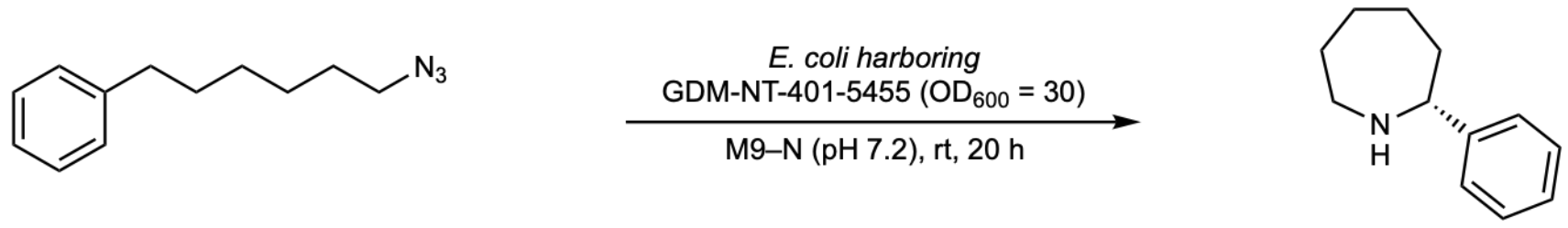

Reaction performed **with GDM-NT-401-5455** (whole *E.coli* cell)

Entry	Product	IS	P/IS	Yield (%)	Average (%)	STDEV	Average TTN
1	282.3	4323.3	0.07	12.4			
2	292.8	4457.5	0.07	12.5	13	0.9	232
3	326.9	4438.7	0.07	14.0			

Calibration curve and data analysis for 2o

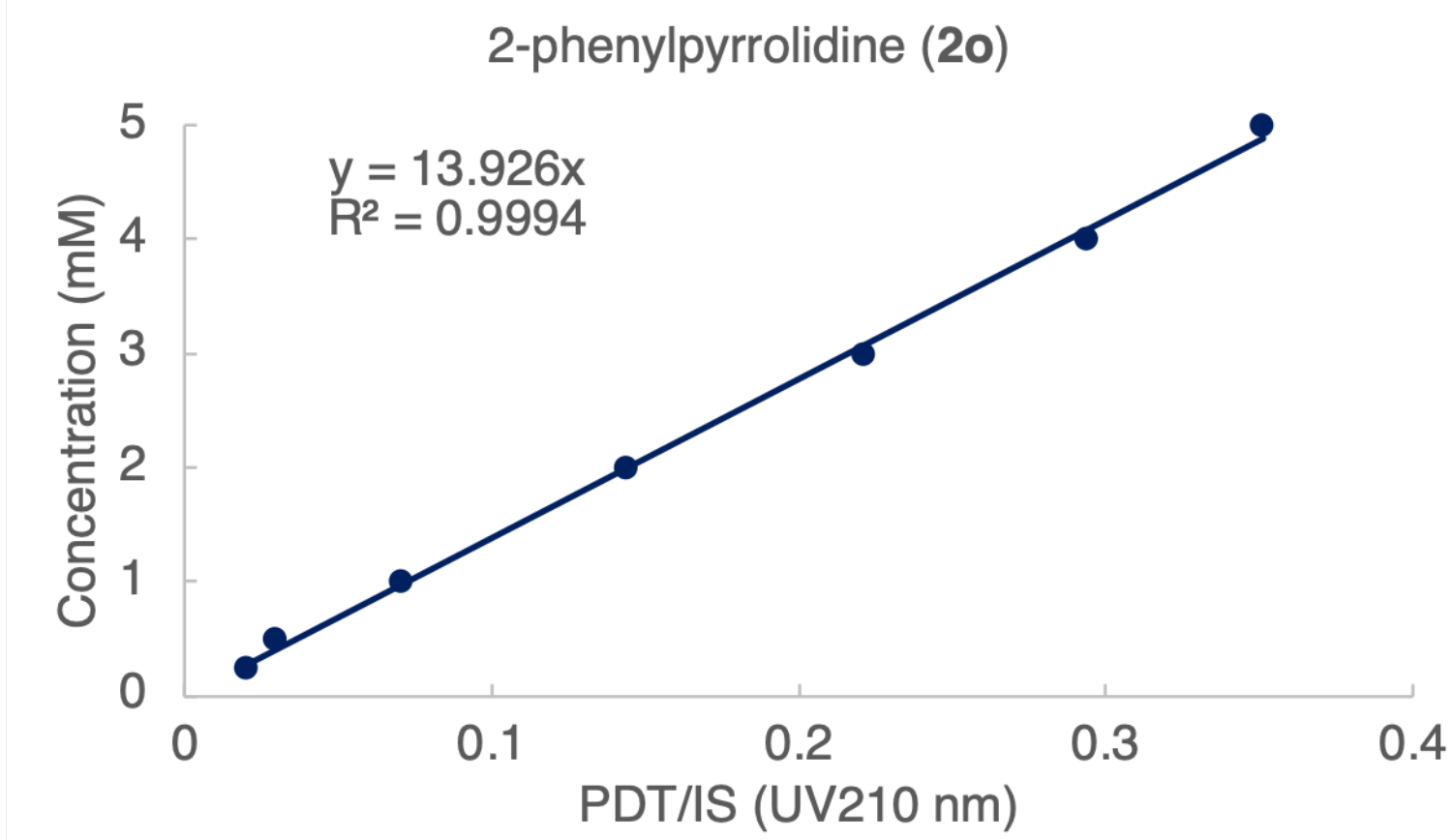

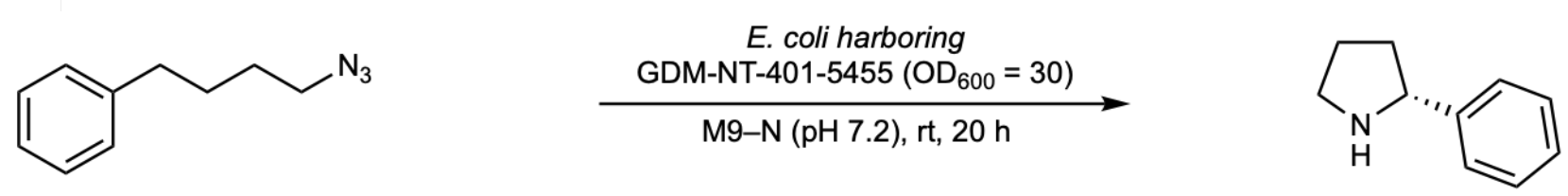

Reaction performed **with GDM-NT-401-5455** (whole *E.coli* cell)

Entry	Product	IS	P/IS	Yield (%)	Average (%)	STDEV	Average TTN
1	754.2	4485.0	0.17	46.8			
2	784.2	4434.7	0.18	49.3	48	1.4	851
3	753.1	4477.3	0.17	46.9			

Calibration curve and data analysis for 2p

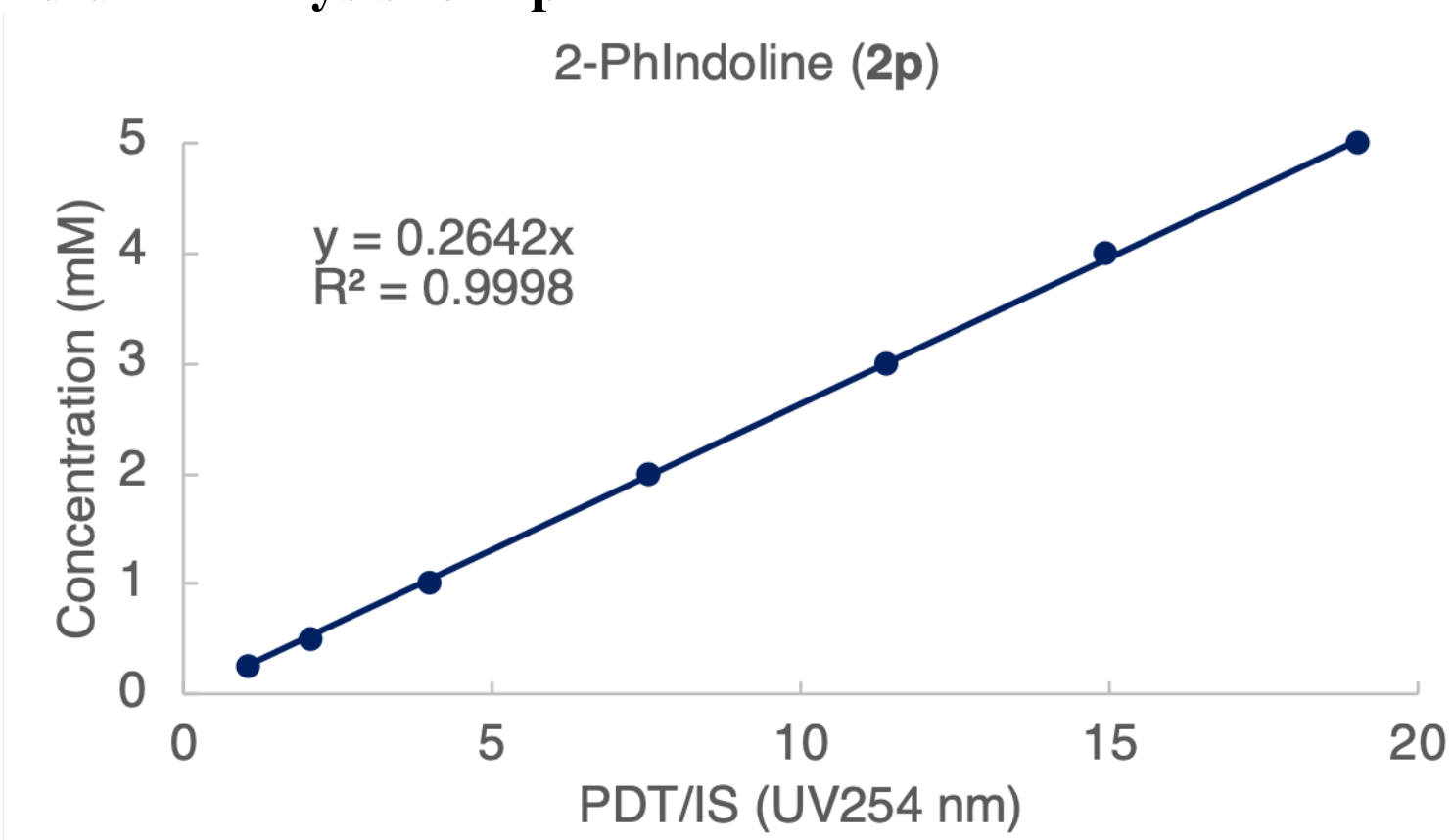

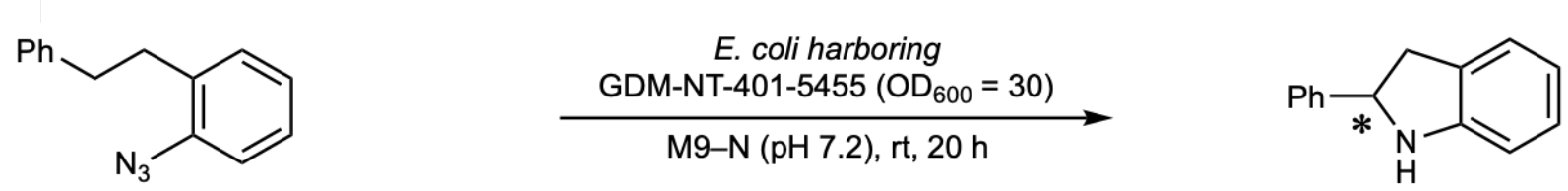

Reaction performed **with GDM-NT-401-5455** (whole *E.coli* cell)

Entry	Product	IS	P/IS	Yield (%)	Average (%)	STDEV	Average TTN
1	1144.5	94.7	12.09	63.9	64	1.1	1144
2	1138.4	92.2	12.35	65.2			

3	1114.6	93.4	11.93	63.1

Calibration curve and data analysis for 2q

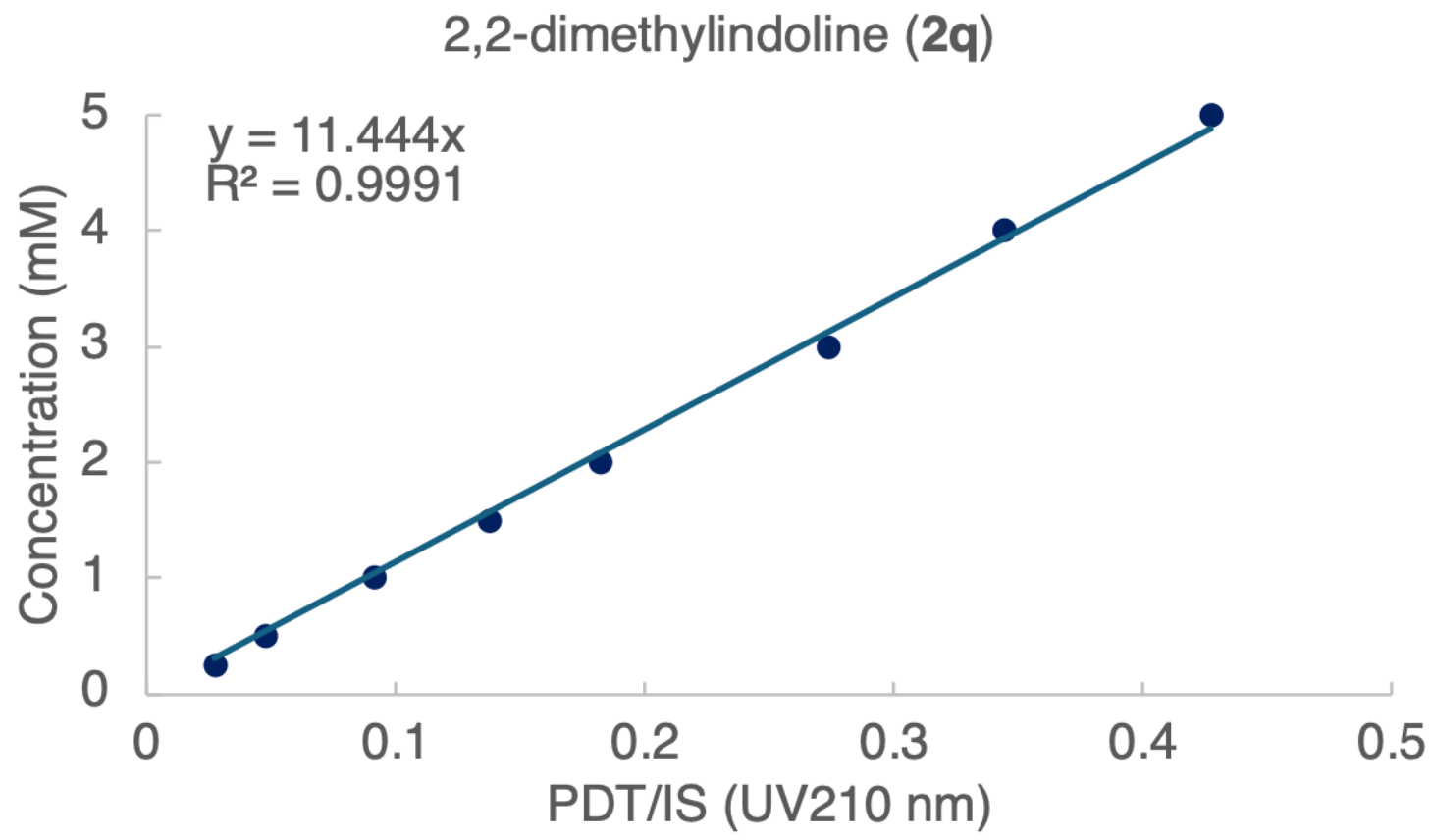

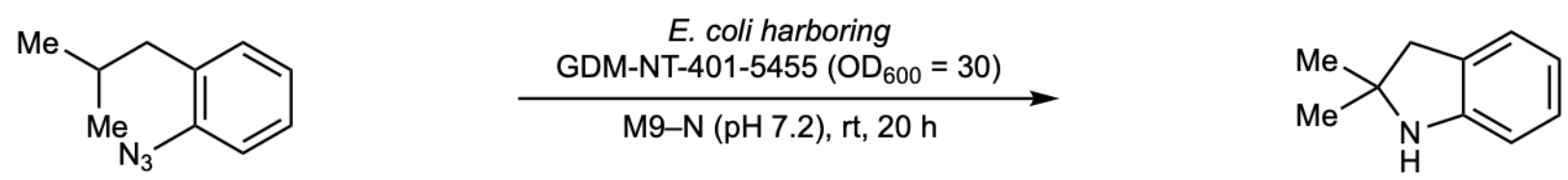

Reaction performed **with GDM-NT-401-5455** (whole *E.coli* cell)

Entry	Product	IS	P/IS	Yield (%)	Average (%)	STDEV	Average TTN
1	2268.5	5164.6	0.44	100.5			
2	2152.5	5250.2	0.41	93.8	99	4.7	1769
3	2272.8	5057.5	0.45	102.9			

Analytical-Scale Enzymatic Reaction Regioselectivity Analysis

As all reactions performed with **GDM-NT-401-5455** have >99:1 rr, we omitted this section of original data for simplicity (refer to enantioselectivity analysis section for detailed evidence). In this section, we only demonstrate regioselectivity of reactions performed with ***Tm*Pgb-G7-5448**. All regioselectivities were determined by analyzing the integration ratio on single ion channel mass spectrometry. There are two sources of original data: 1) reverse-phase HPLC-MS analysis using C18 column. 2) chiral polar ionic HPLC-MS analysis using chiral V column. The best source of data for analyzing regioselectivity was chosen based on resolution factor (R_s). When HPLC-MS analysis using C18 column was used, we show the screenshots of the single ion channel spectrums below. When HPLC-MS analysis using polar ionic chiral V column was used, please refer to the enantioselectivity analysis section for screenshots of the spectrums.

Part 1: Regioselectivity analysis of *Tm*Pgb-PIP lineage.

Table S23. Regioselectivity analysis of ***Tm*Pgb-PIP** lineage. Results analyzed on polar ionic HPLC-MS.

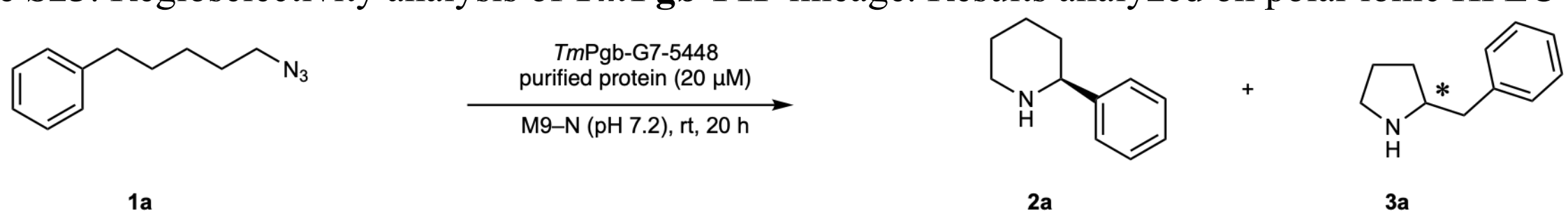

	**2a**-f1	**2a**-f2	**3a**-f1	**3a**-f2	**2a**	**3a**	rr (**2a**:**3a**)
***Tm*Pgb-wt**	4578	230690	455684	8001453	235267	8457137	3:97
**PIP-G1**	0	1434458	1486397	270189	1434458	1756586	45:55
**PIP-G2**	0	1996270	1270715	290422	1996270	1561137	56:44
**PIP-G3**	0	2377421	708547	267999	2377421	976546	71:29
**PIP-G4**	0	5318512	471101	219056	5318512	690157	89:11
**PIP-G5**	0	10183343	1133631	514638	10183343	1648270	86:14
**PIP-G6**	0	8326293	1115724	356353	8326293	1472076	85:15
**PIP-G7**	0	16744088	2771103	467014	16744088	3238117	84:16

Part 2: Regioselectivity analysis of GDM-NT variants

Table S24. Regioselectivity analysis of Selected results using selected variants in recycled-scaffold design based on **GDM-NT-121**. Results analyzed on polar ionic HPLC-MS.

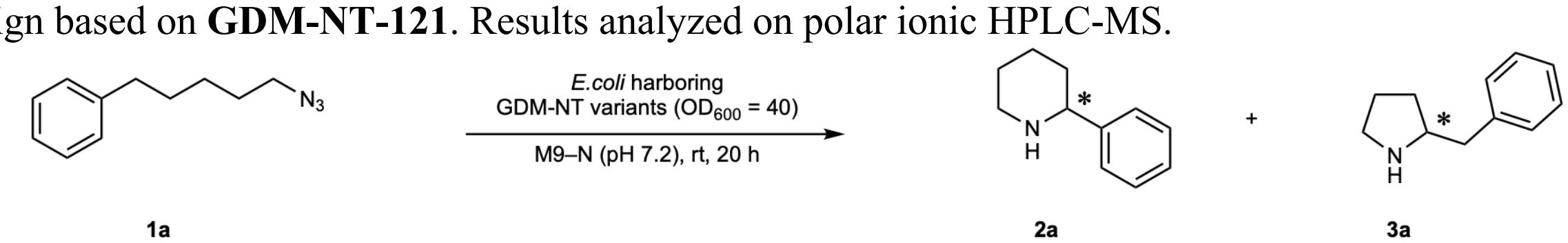

	**2a**-f1	**2a**-f2	**3a**-f1	**3a**-f2	**2a**	**3a**	rr (**2a**:**3a**)
***Tm*Pgb-wt**	1010	64158	114644	1715495	65168	1830139	3:97
**GDM-NT-121**	105591	244460	5714	7718	350051	13432	96:4
**GDM-NT-215**	266139	430943	8949	8810	697081	17758	98:2
**GDM-NT-217**	285303	151843	4884	12284	437147	17168	96:4
**GDM-NT-226**	4647607	2520857	1871	3029	7168464	4901	>99:1
**GDM-NT-227**	189184	175706	11246	12413	364890	23659	94:6
**GDM-NT-228**	85353	28646	7995	5448	113999	13444	89:11

Table S25. Regioselectivity analysis of Selected results using selected variants in recycled-scaffold design based on **GDM-NT-151**. Results analyzed on polar ionic HPLC-MS.

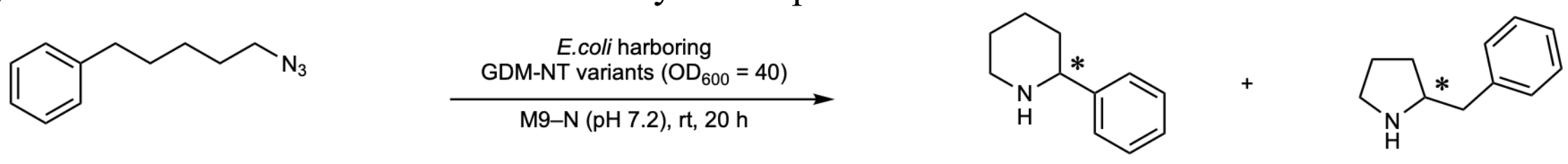

1a 2a 3a

	**2a**-f1	**2a**-f2	**3a**-f1	**3a**-f2	**2a**	**3a**	rr (**2a:3a**)
***Tm*Pgb-wt**	1010	64158	114644	1715495	65168	1830139	3:97
**GDM-NT-151**	18301	98460	2733	3969	116761	6702	95:5
**GDM-NT-350**	113820	297942	0	25090	411761	25090	94:6
**GDM-NT-356**	171827	1100276	6839	6149	1272102	12988	99:1
**GDM-NT-359**	168656	767392	3480	12155	936048	15635	98:2
**GDM-NT-361**	124639	197673	2243	16327	322312	18570	95:5
**GDM-NT-362**	1770315	102717	2701	1195	1873032	3896	>99:1

Part 3: Regioselectivity analysis of substrate scope study.
Reactions performed with ***Tm*Pgb-PIP-G7-5448** on polar ionic chiral HPLC-MS or reverse-phase HPLC-MS.

Regioselectivity analysis of **2a** using ***Tm*Pgb-PIP-G7-5448** (polar ionic HPLC-MS):

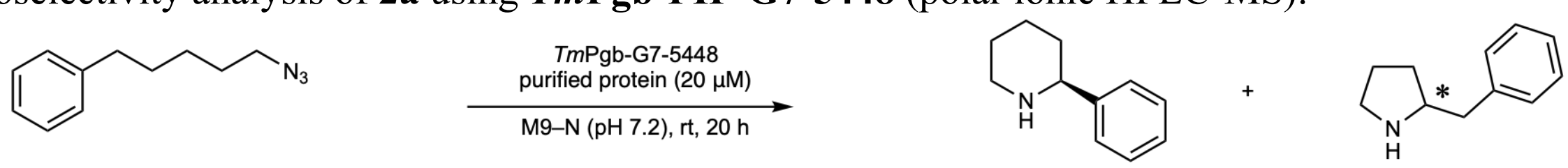

1a 2a 3a

	**2a**-f1	**2a**-f2	**3a**-f1	**3a**-f2	**2a**	**3a**	rr (**2a:3a**)
**Area (ion counts)**	0	9426293	1667976	495131	9426293	2163110	81:19

Regioselectivity analysis of **2b** using ***Tm*Pgb-PIP-G7-5448** (polar ionic HPLC-MS):

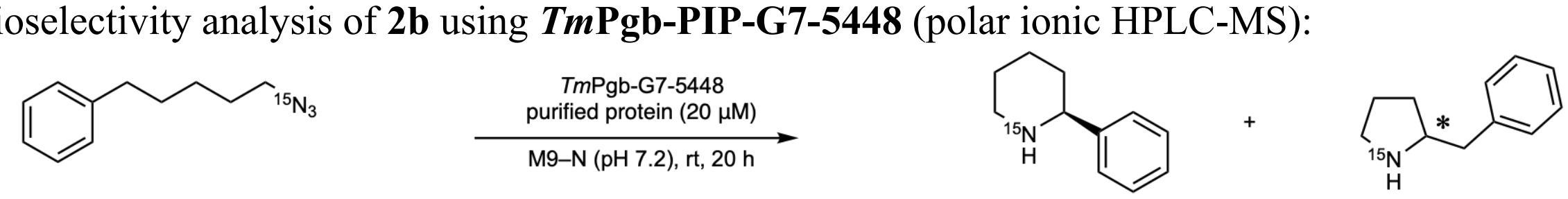

1b 2b 3b

	**2a**-f1	**2a**-f2	**3a**-f1	**3a**-f2	**2a**	**3a**	rr (**2a:3a**)
**Area (ion counts)**	0	2834641	634790	184361	2834641	819151	78:22

Regioselectivity analysis of **2c** using ***Tm*Pgb-PIP-G7-5448** (polar ionic HPLC-MS):

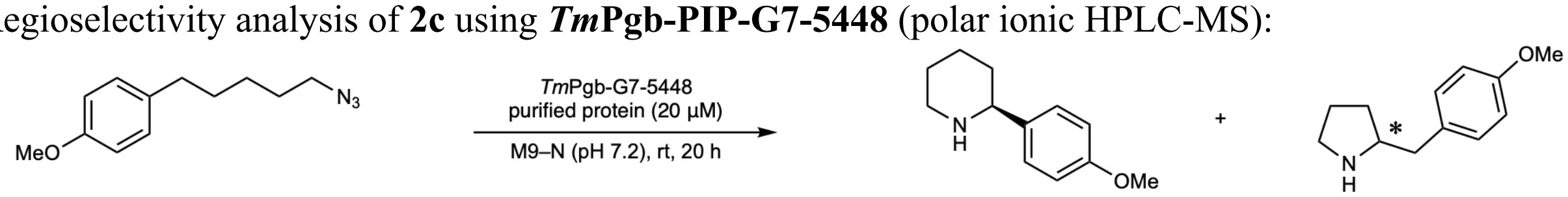

1c 2c 3c

	**2c**-f1	**2c**-f2	**3c**-f1	**3c**-f2	**2c**	**3c**	rr (**2c:3c**)
**Area (ion counts)**	0	203817.2	1315.3	37518.9	203817.2	38834.2	84:16

Regioselectivity analysis of **2f** using ***Tm*Pgb-PIP-G7-5448** (reverse phase HPLC-MS on C18 column):

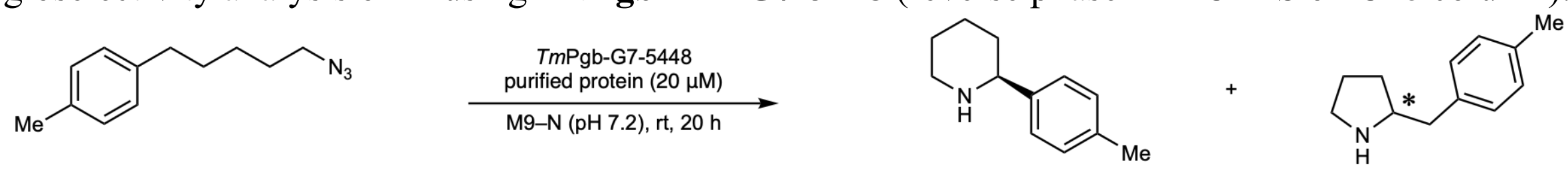

1d **2d** **3d**

	**2e**	**3e**	rr (**2e**:**3e**)	Average rr (**2e**:**3e**)
**1**	377340.2	30129.2	92.6:7.4	
**2**	391193.4	27640.4	93.4:6.6	93:7
**3**	387767.1	30128.6	92.8:7.2	

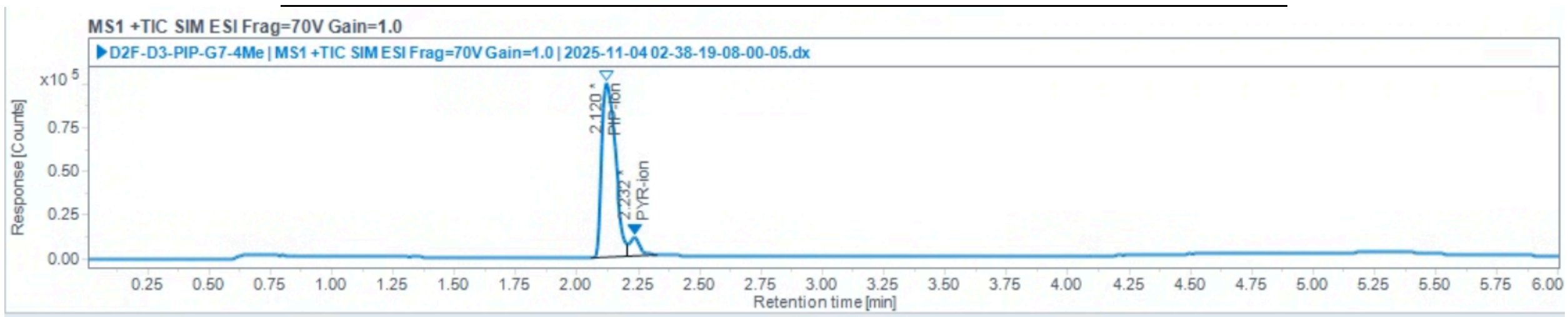

Injection Results

Peaks Summary

Name	Signal description	RT (min)	Area (Counts·s)	Area%	Height (Counts)	Height%	Start time (min)	End time (min)
PIP-ion	MS1 +TIC SIM ESI Frag=70V Gain=1.0	2.120	377340.223	92.606	99584.696	90.24	2.059	2.205
	MS1 +TIC SIM ESI Frag=70V Gain=1.0	2.232	30129.180	7.394	10768.210	9.76	2.205	2.326

lcurve-90A.pmx

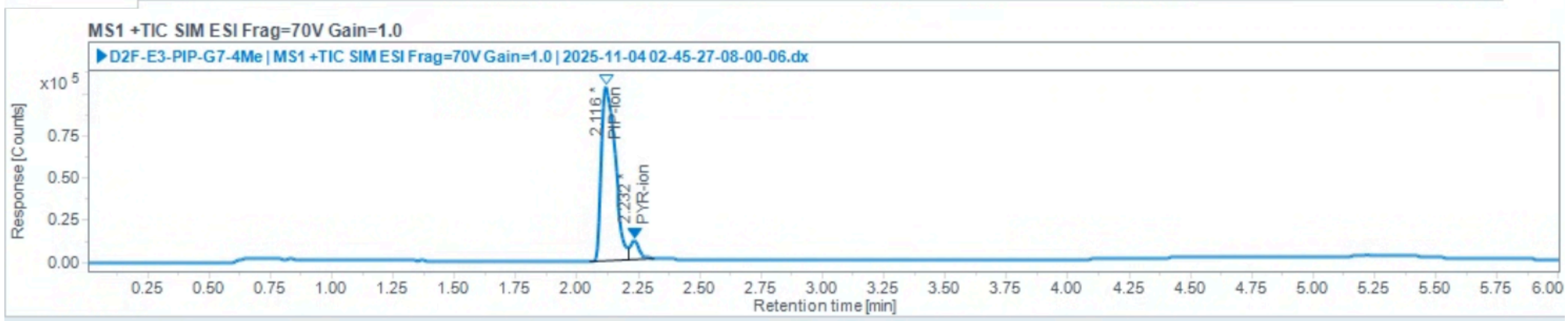

Injection Results

Peaks Summary

Name	Signal description	RT (min)	Area (Counts·s)	Area%	Height (Counts)	Height%	Start time (min)	End time (min)
PIP-ion	MS1 +TIC SIM ESI Frag=70V Gain=1.0	2.116	391193.422	93.401	103046.399	90.28	2.049	2.209
PYR-ion	MS1 +TIC SIM ESI Frag=70V Gain=1.0	2.232	27640.372	6.599	11091.129	9.72	2.209	2.316

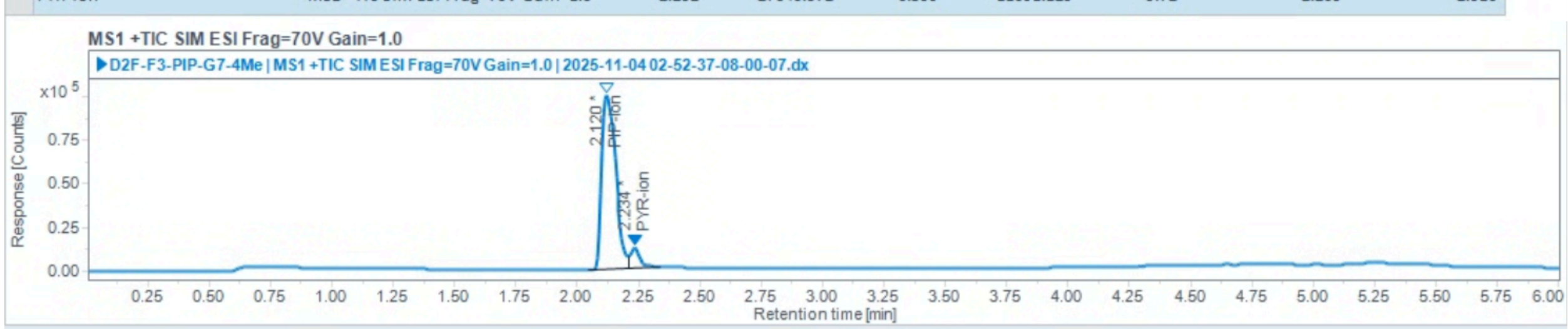

Injection Results

Peaks Summary

Name	Signal description	RT (min)	Area (Counts·s)	Area%	Height (Counts)	Height%	Start time (min)	End time (min)
PIP-ion	MS1 +TIC SIM ESI Frag=70V Gain=1.0	2.120	387767.074	92.790	99736.682	89.84	2.048	2.209
PYR-ion	MS1 +TIC SIM ESI Frag=70V Gain=1.0	2.234	30128.574	7.210	11283.322	10.16	2.209	2.337

Regioselectivity analysis of **2f** using ***Tm*Pgb-PIP-G7-5448** (reverse phase HPLC-MS on C18 column):

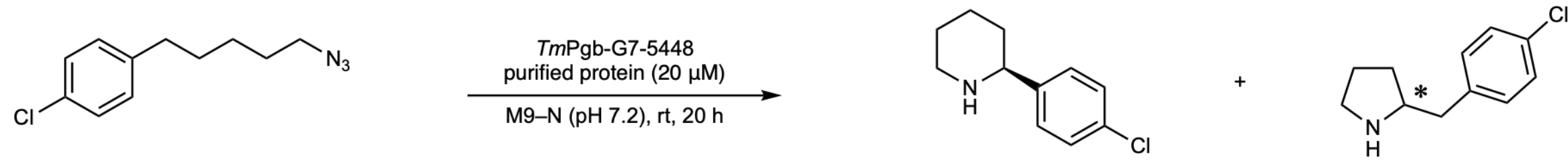

1e **2e** **3e**

	**2e**	**3e**	rr (**2e**:**3e**)	Average rr (**2e**:**3e**)
**1**	170626.9	14325.8	92.3:7.7	
**2**	169557.4	13730.7	92.5:7.5	92:8
**3**	150887.4	12134.5	92.6:7.4	

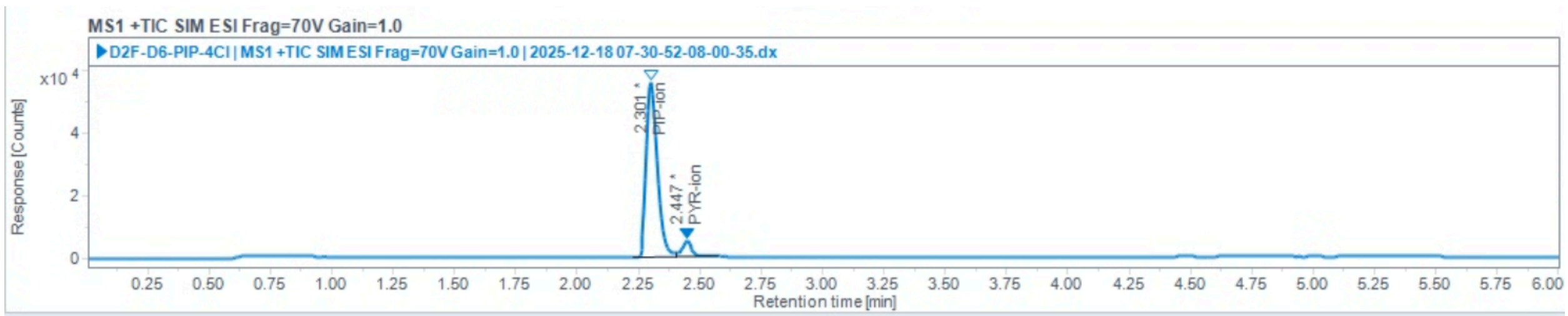

Injection Results

Peaks Summary

Name	Signal description	RT (min)	Area (Counts·s)	Area%	Height (Counts)	Height%	Start time (min)	End time (min)
PIP-ion	MS1 +TIC SIM ESI Frag=70V Gain=1.0	2.301	170626.918	92.254	56006.345	91.55	2.226	2.404
PYR-ion	MS1 +TIC SIM ESI Frag=70V Gain=1.0	2.447	14325.824	7.746	5171.816	8.45	2.404	2.576

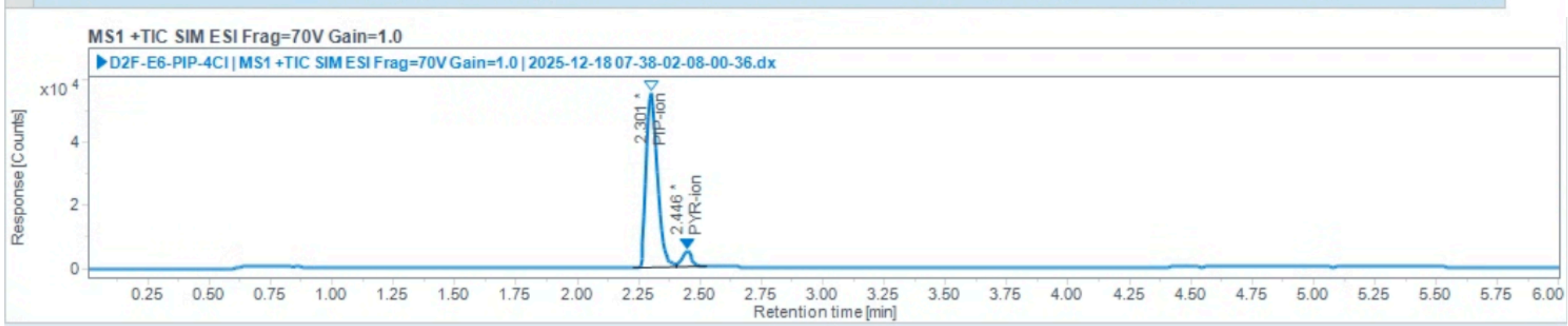

Injection Results

Peaks Summary

Name	Signal description	RT (min)	Area (Counts·s)	Area%	Height (Counts)	Height%	Start time (min)	End time (min)
PIP-ion	MS1 +TIC SIM ESI Frag=70V Gain=1.0	2.301	169557.362	92.509	55239.240	91.86	2.228	2.404
PYR-ion	MS1 +TIC SIM ESI Frag=70V Gain=1.0	2.446	13730.691	7.491	4892.416	8.14	2.404	2.528

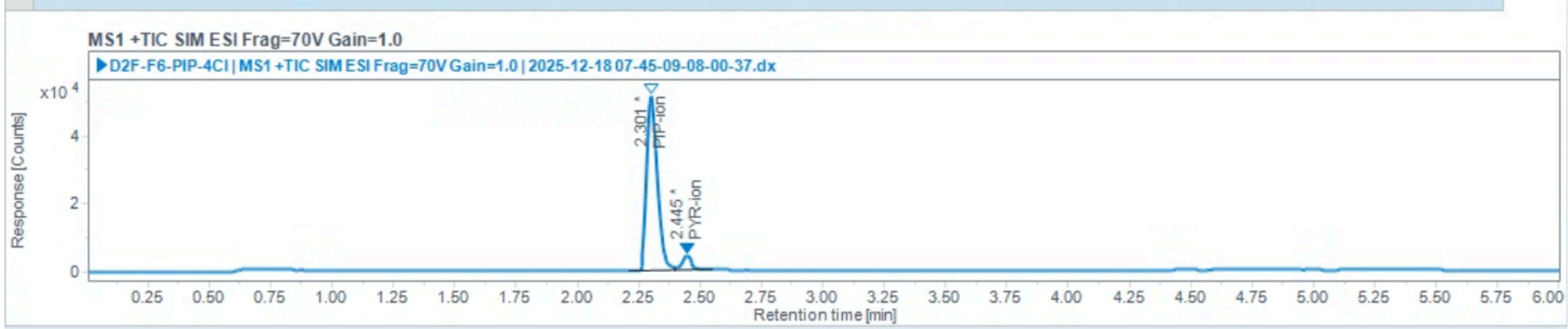

Injection Results

Peaks Summary

Name	Signal description	RT (min)	Area (Counts·s)	Area%	Height (Counts)	Height%	Start time (min)	End time (min)
PIP-ion	MS1 +TIC SIM ESI Frag=70V Gain=1.0	2.301	150887.444	92.557	51420.110	92.35	2.210	2.401
PYR-ion	MS1 +TIC SIM ESI Frag=70V Gain=1.0	2.445	12134.513	7.443	4257.567	7.65	2.401	2.547

Regioselectivity analysis of **2f** using ***Tm*Pgb-PIP-G7-5448** (reverse phase HPLC-MS on C18 column):

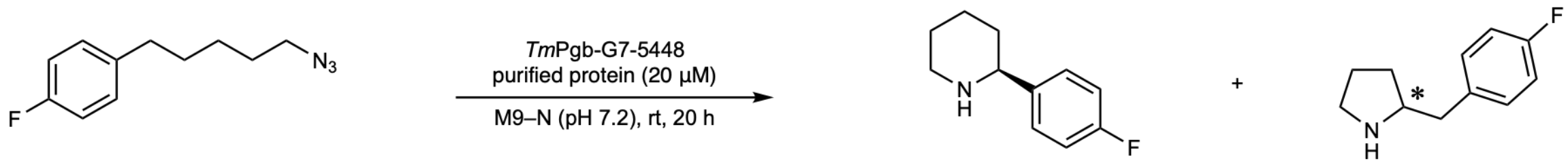

1f **2f** **3f**

	**2f**	**3f**	rr (**2f:3f**)	Average rr (**2f:3f**)
**1**	154974.7	27128.5	85.1:14.9	
**2**	127690.2	24034.0	84.2:15.8	84:16
**3**	161052.5	31395.7	83.7:16.3	

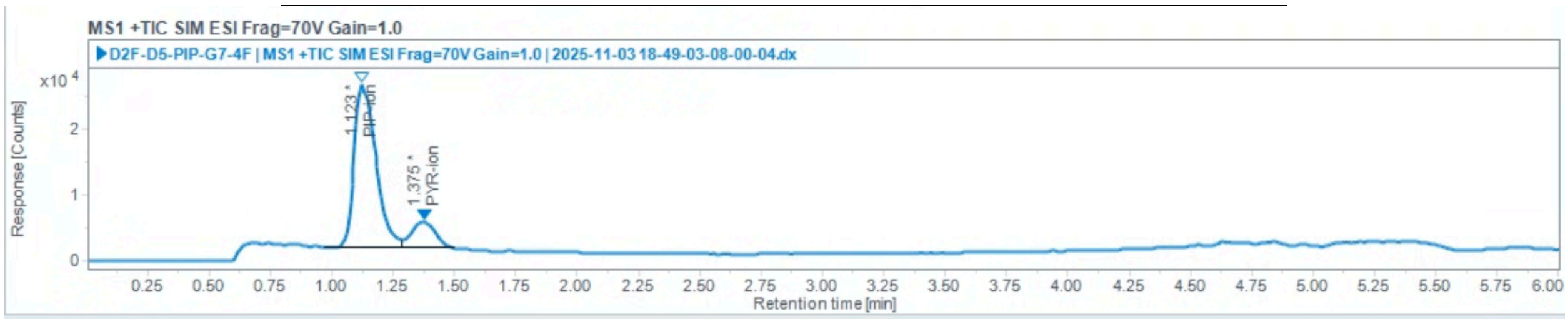

Injection Results

Peaks Summary

Name	Signal description	RT (min)	Area (Counts·s)	Area%	Height (Counts)	Height%	Start time (min)	End time (min)
PIP-ion	MS1 +TIC SIM ESI Frag=70V Gain=1.0	1.123	154974.680	85.103	24939.911	86.01	0.972	1.289
PYR-ion	MS1 +TIC SIM ESI Frag=70V Gain=1.0	1.375	27128.520	14.897	4055.868	13.99	1.289	1.498

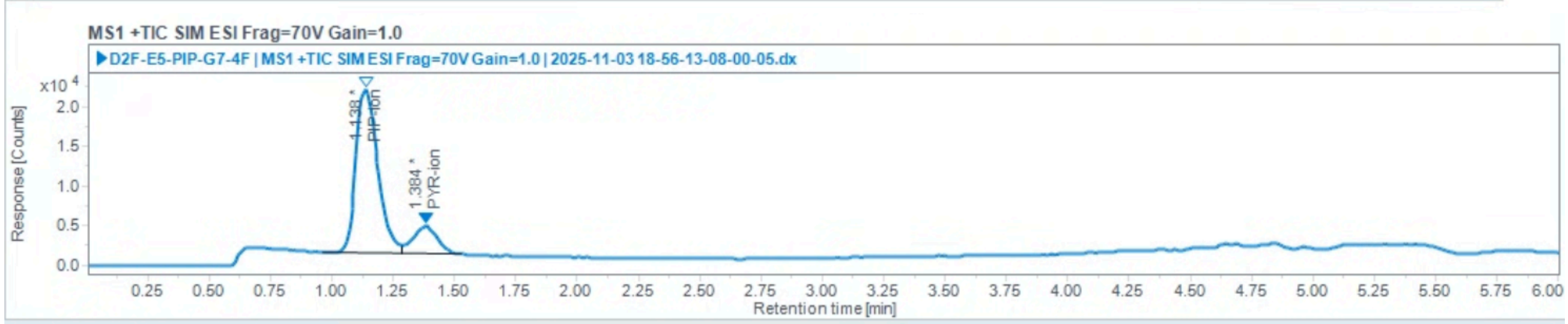

Injection Results

Peaks Summary

Name	Signal description	RT (min)	Area (Counts·s)	Area%	Height (Counts)	Height%	Start time (min)	End time (min)
PIP-ion	MS1 +TIC SIM ESI Frag=70V Gain=1.0	1.138	127690.195	84.159	20574.759	85.15	0.961	1.289
PYR-ion	MS1 +TIC SIM ESI Frag=70V Gain=1.0	1.384	24034.029	15.841	3587.499	14.85	1.289	1.531

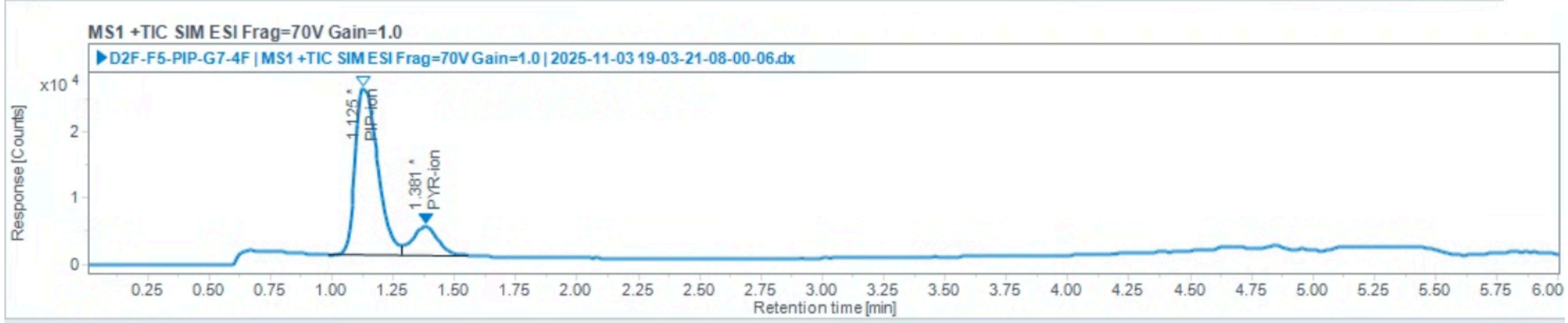

Injection Results

Peaks Summary

Name	Signal description	RT (min)	Area (Counts·s)	Area%	Height (Counts)	Height%	Start time (min)	End time (min)
PIP-ion	MS1 +TIC SIM ESI Frag=70V Gain=1.0	1.125	161052.532	83.686	25143.111	84.87	0.989	1.289
PYR-ion	MS1 +TIC SIM ESI Frag=70V Gain=1.0	1.381	31395.699	16.314	4481.209	15.13	1.289	1.554

Regioselectivity analysis of **2g** using ***Tm*Pgb-PIP-G7-5448** (reverse phase HPLC-MS on C18 column):

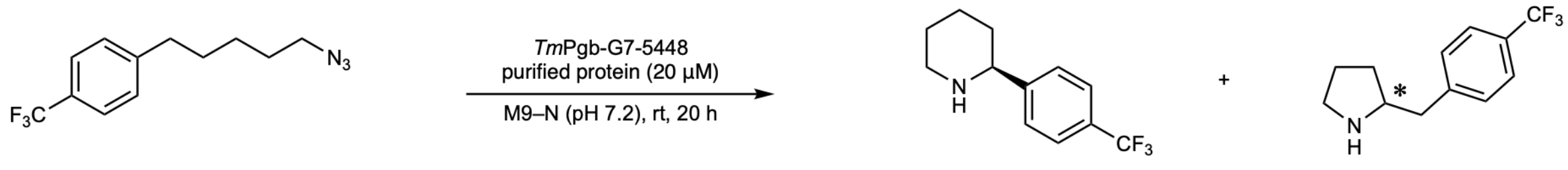

1g **2g** **3g**

	**2g**	**3g**	rr (**2g**:**3g**)	Average rr (**2g**:**3g**)
**1**	33822.5	2900.2	92.1:7.9	
**2**	32734.5	2659.8	92.5:7.5	92:8
**3**	34723.2	2842.1	92.4:92:6	

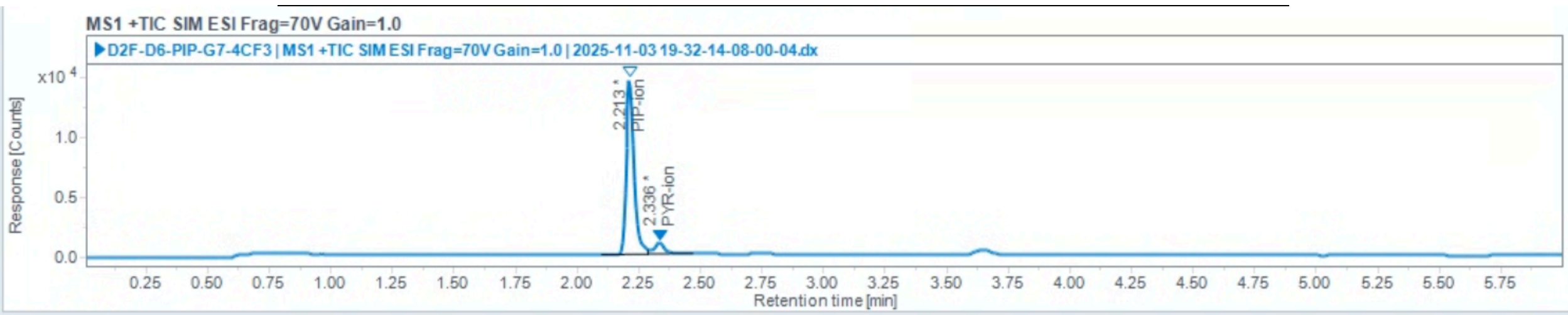

Injection Results

Peaks Summary

Name	Signal description	RT (min)	Area (Counts·s)	Area%	Height (Counts)	Height%	Start time (min)	End time (min)
PIP-ion	MS1 +TIC SIM ESI Frag=70V Gain=1.0	2.213	33822.499	92.102	14647.982	93.77	2.101	2.287
PYR-ion	MS1 +TIC SIM ESI Frag=70V Gain=1.0	2.336	2900.200	7.898	972.908	6.23	2.287	2.468

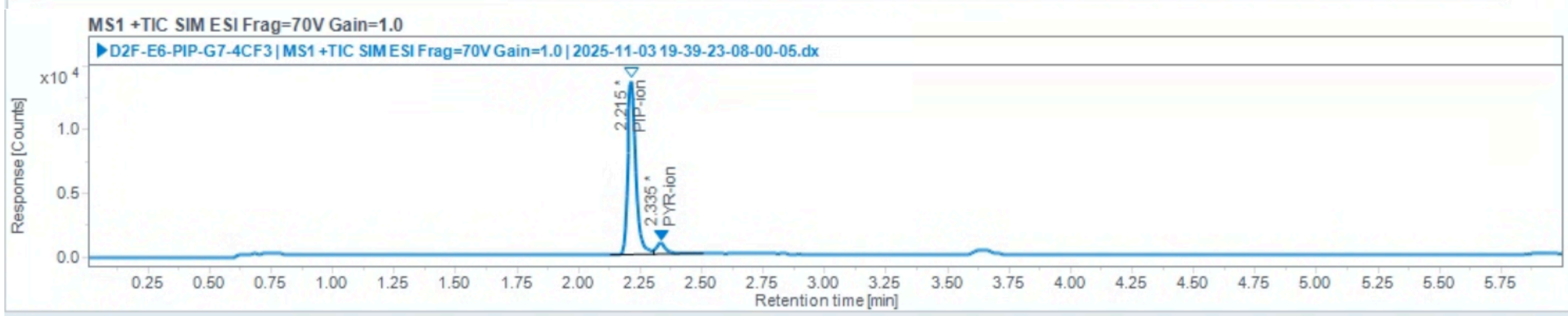

Injection Results

Peaks Summary

Name	Signal description	RT (min)	Area (Counts·s)	Area%	Height (Counts)	Height%	Start time (min)	End time (min)
PIP-ion	MS1 +TIC SIM ESI Frag=70V Gain=1.0	2.215	32734.469	92.485	13677.457	93.62	2.129	2.304
PYR-ion	MS1 +TIC SIM ESI Frag=70V Gain=1.0	2.335	2659.771	7.515	932.196	6.38	2.304	2.507

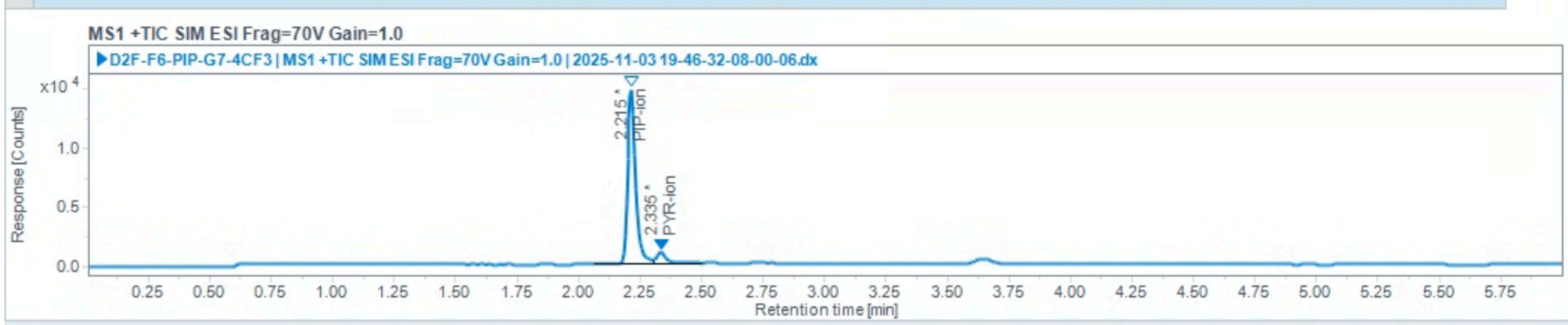

Injection Results

Peaks Summary

Name	Signal description	RT (min)	Area (Counts·s)	Area%	Height (Counts)	Height%	Start time (min)	End time (min)
PIP-ion	MS1 +TIC SIM ESI Frag=70V Gain=1.0	2.215	34723.193	92.434	14859.534	93.80	2.061	2.304
PYR-ion	MS1 +TIC SIM ESI Frag=70V Gain=1.0	2.335	2842.050	7.566	981.927	6.20	2.304	2.502

Regioselectivity analysis of **2h** using ***Tm*Pgb-PIP-G7-5448** (polar ionic HPLC-MS):

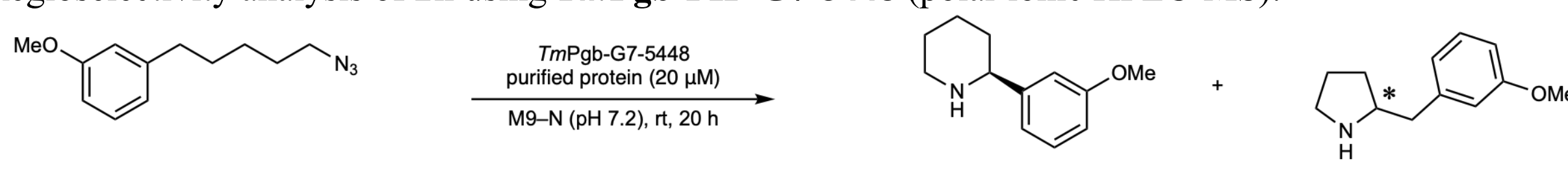

1h 2h 3h

	**2h**-f1 + **2h**-f2	**3h**-f1	**3h**-f2	**2h**	**3h**	rr (**2h:3h**)
**Area (ion counts)**	2802725.8	729527.0	89433.7	2802725.8	818960.7	77:23

Regioselectivity analysis of **2i** using ***Tm*Pgb-PIP-G7-5448** (reverse phase HPLC-MS on C18 column):

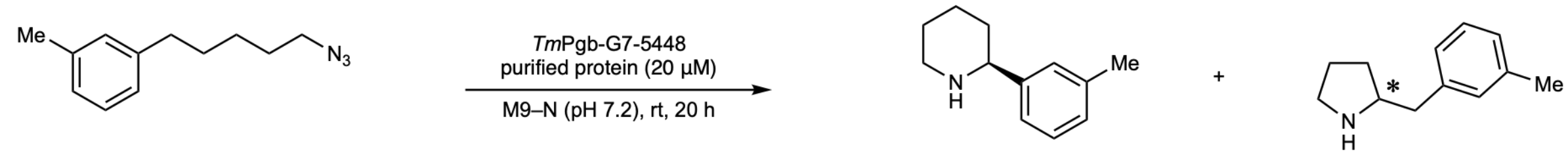

1i **2i** **3i**

	**2i**	**3i**	rr (**2i**:**3i**)	Average rr (**2i**:**3i**)
1	143320.0	64433.3	69.0:31.0	
2	115443.8	49819.4	69.9:30.1	70:30
3	132815.8	53755.6	71.2:28.8	

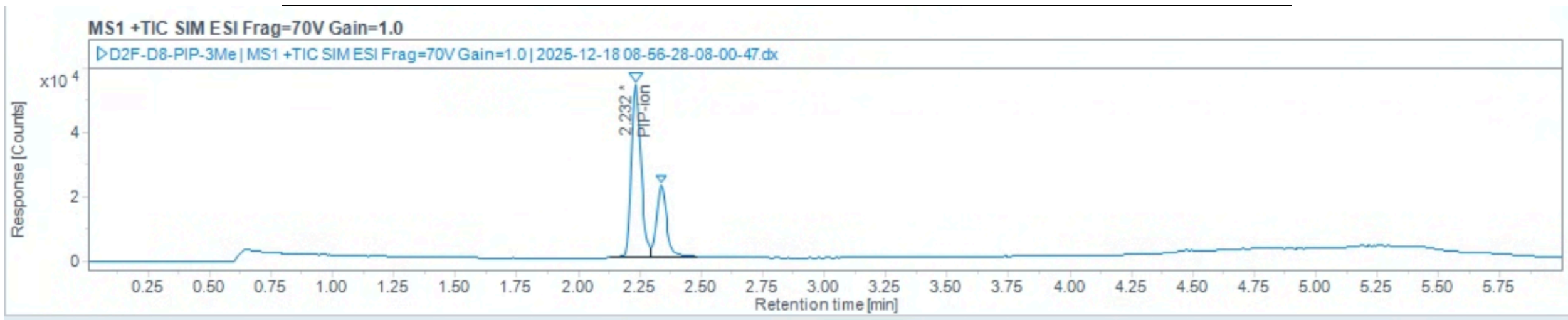

Injection Results

Peaks Summary

Name	Signal description	RT (min)	Area (Counts·s)	Area%	Height (Counts)	Height%	Start time (min)	End time (min)
PIP-ion	MS1 +TIC SIM ESI Frag=70V Gain=1.0	2.232	143319.957	68.986	53576.910	70.43	2.131	2.296
PYR-ion	MS1 +TIC SIM ESI Frag=70V Gain=1.0	2.337	64433.288	31.014	22496.532	29.57	2.296	2.486

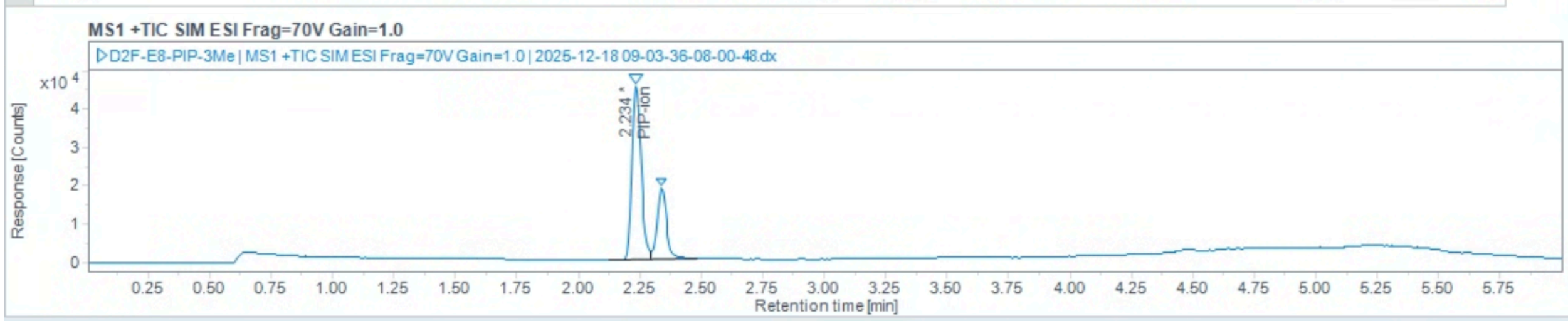

Injection Results

Peaks Summary

Name	Signal description	RT (min)	Area (Counts·s)	Area%	Height (Counts)	Height%	Start time (min)	End time (min)
PIP-ion	MS1 +TIC SIM ESI Frag=70V Gain=1.0	2.234	115443.764	69.854	45279.191	70.95	2.123	2.296
PYR-ion	MS1 +TIC SIM ESI Frag=70V Gain=1.0	2.338	49819.424	30.146	18543.154	29.05	2.296	2.483

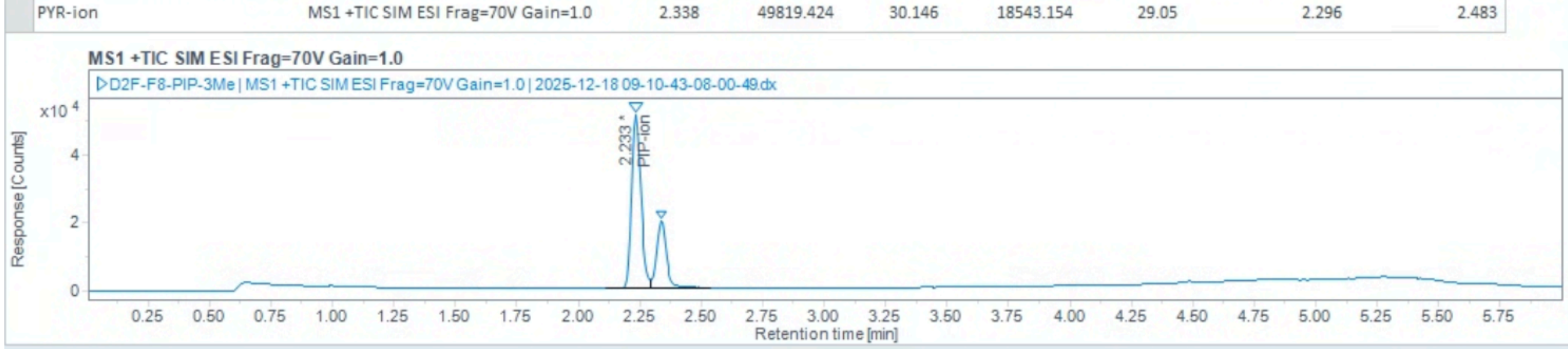

Injection Results

Peaks Summary

Name	Signal description	RT (min)	Area (Counts·s)	Area%	Height (Counts)	Height%	Start time (min)	End time (min)
PIP-ion	MS1 +TIC SIM ESI Frag=70V Gain=1.0	2.233	132815.752	71.188	50984.159	72.07	2.114	2.294
PYR-ion	MS1 +TIC SIM ESI Frag=70V Gain=1.0	2.337	53755.629	28.812	19756.403	27.93	2.294	2.538

Regioselectivity analysis of **2j** using ***Tm*Pgb-PIP-G7-5448** (reverse phase HPLC-MS on C18 column):

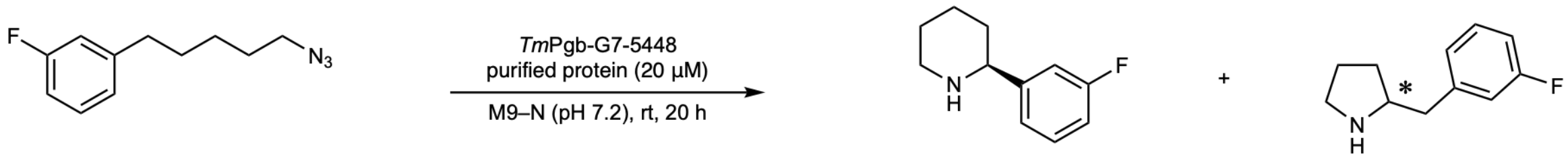

1j 2j 3j

	**2j**	**3j**	rr (**2j**:**3j**)	Average rr (**2j**:**3j**)
1	131907.4	36290.7	78.4:21.6	
2	114366.2	33668.3	77.3:22.7	77:23
3	125007.4	38649.2	76.4:23.6	

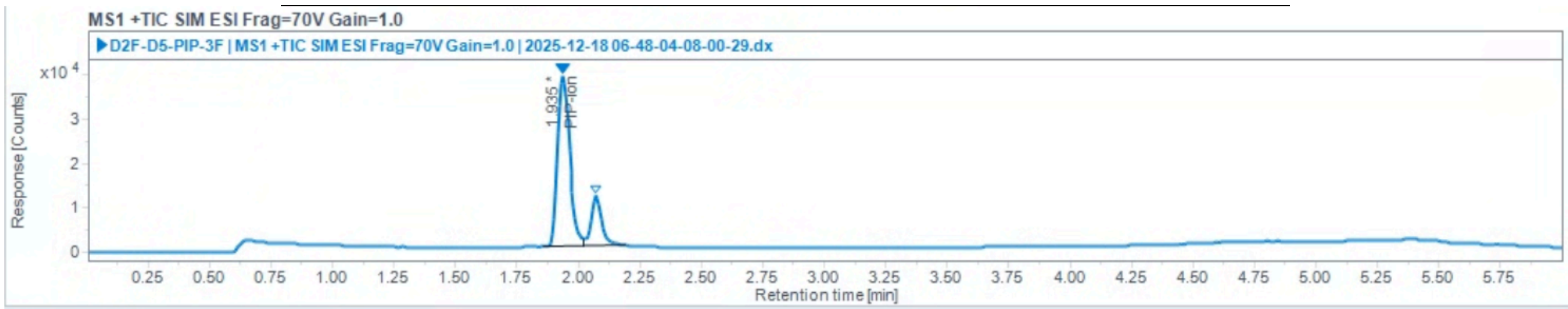

Injection Results

Peaks Summary

Name	Signal description	RT (min)	Area (Counts·s)	Area%	Height (Counts)	Height%	Start time (min)	End time (min)
PIP-ion	MS1 +TIC SIM ESI Frag=70V Gain=1.0	1.935	131907.418	78.424	38562.151	77.34	1.854	2.018
PYR-ion	MS1 +TIC SIM ESI Frag=70V Gain=1.0	2.072	36290.663	21.576	11296.480	22.66	2.018	2.193

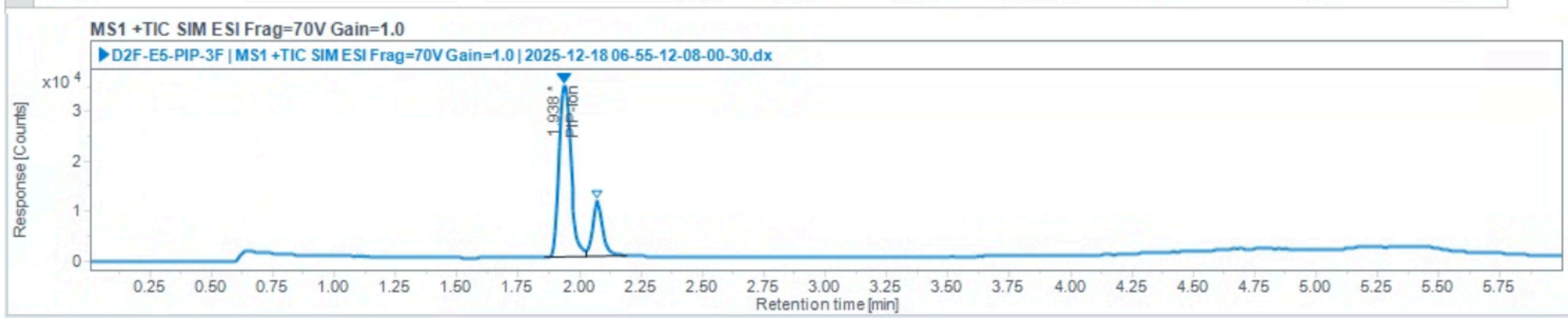

Injection Results

Peaks Summary

Name	Signal description	RT (min)	Area (Counts·s)	Area%	Height (Counts)	Height%	Start time (min)	End time (min)
PIP-ion	MS1 +TIC SIM ESI Frag=70V Gain=1.0	1.938	114366.221	77.256	34470.986	75.50	1.857	2.025
PYR-ion	MS1 +TIC SIM ESI Frag=70V Gain=1.0	2.071	33668.268	22.744	11185.764	24.50	2.025	2.194

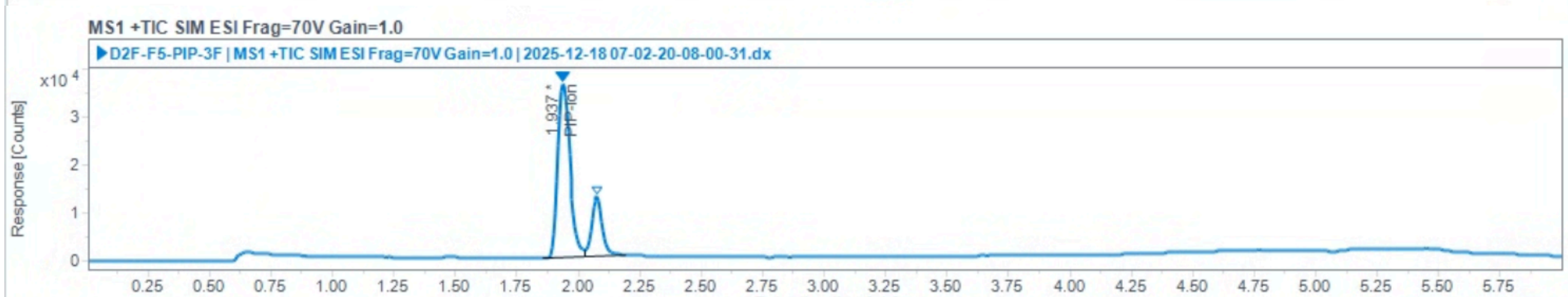

Injection Results

Peaks Summary

Name	Signal description	RT (min)	Area (Counts·s)	Area%	Height (Counts)	Height%	Start time (min)	End time (min)
PIP-ion	MS1 +TIC SIM ESI Frag=70V Gain=1.0	1.937	125007.409	76.384	36238.843	74.33	1.854	2.027
PYR-ion	MS1 +TIC SIM ESI Frag=70V Gain=1.0	2.076	38649.198	23.616	12512.391	25.67	2.027	2.194

Regioselectivity analysis of **2k** using ***Tm*Pgb-PIP-G7-5448** (reverse phase HPLC-MS on C18 column):

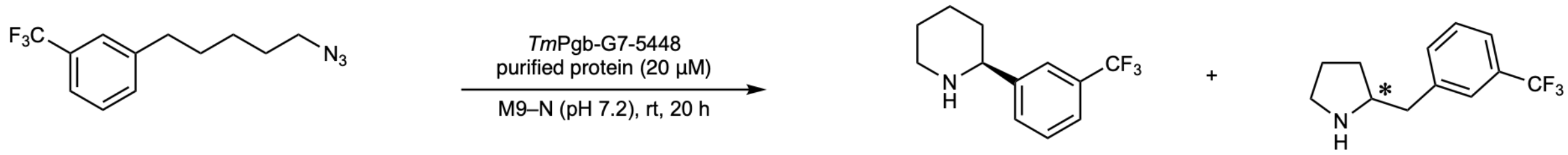

1k **2k** **3k**

	**2k**	**3k**	rr (**2k**:**3k**)	Average rr (**2k**:**3k**)
1	640.8	1528.1	29.5:70.5	
2	566.0	1304.5	30.3:69.7	30:70
3	513.2	1229.4	29.4:70.6	

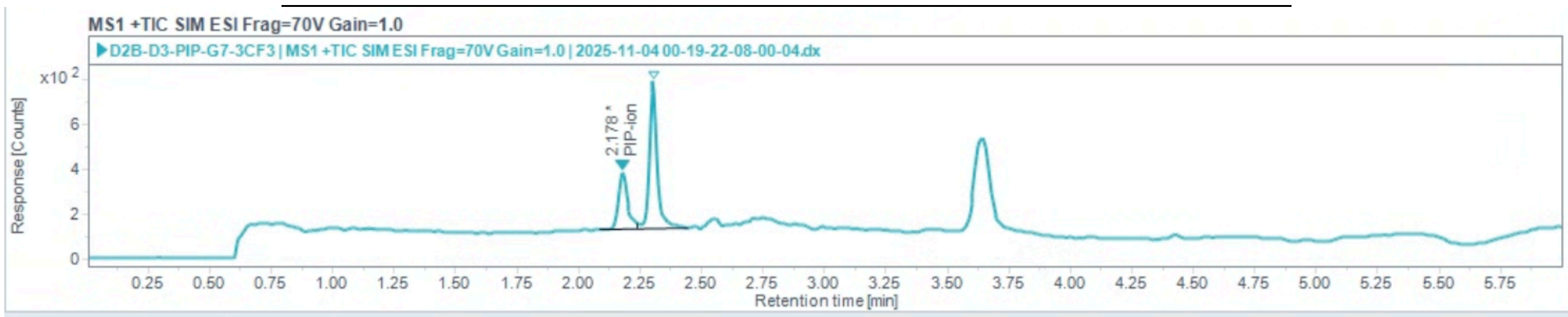

Injection Results

Peaks Summary

Name	Signal description	RT (min)	Area (Counts·s)	Area%	Height (Counts)	Height%	Start time (min)	End time (min)
PIP-ion	MS1 +TIC SIM ESI Frag=70V Gain=1.0	2.178	640.787	29.544	250.550	27.68	2.089	2.241
PYR-ion	MS1 +TIC SIM ESI Frag=70V Gain=1.0	2.303	1528.108	70.456	654.460	72.32	2.241	2.443

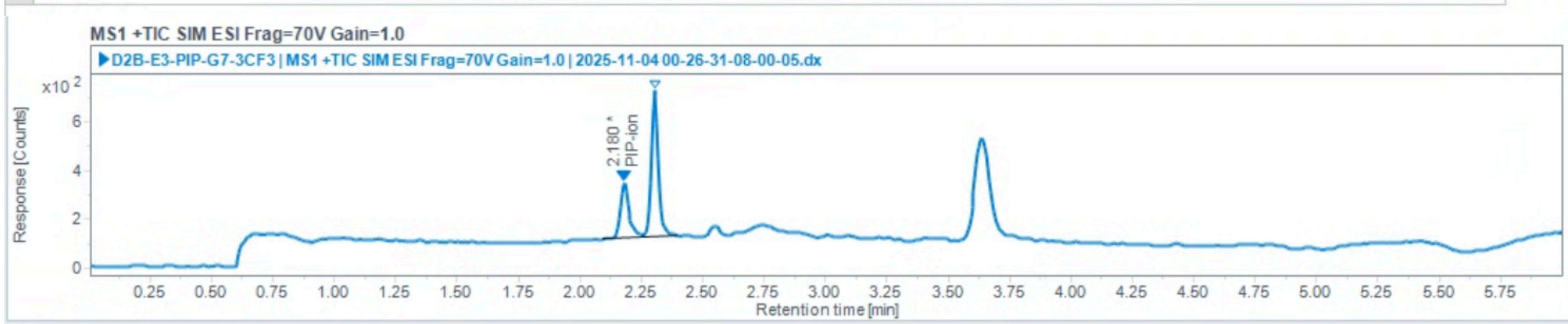

Injection Results

Peaks Summary

Name	Signal description	RT (min)	Area (Counts·s)	Area%	Height (Counts)	Height%	Start time (min)	End time (min)
PIP-ion	MS1 +TIC SIM ESI Frag=70V Gain=1.0	2.180	565.985	30.259	226.489	27.34	2.095	2.260
PYR-ion	MS1 +TIC SIM ESI Frag=70V Gain=1.0	2.304	1304.462	69.741	601.873	72.66	2.260	2.400

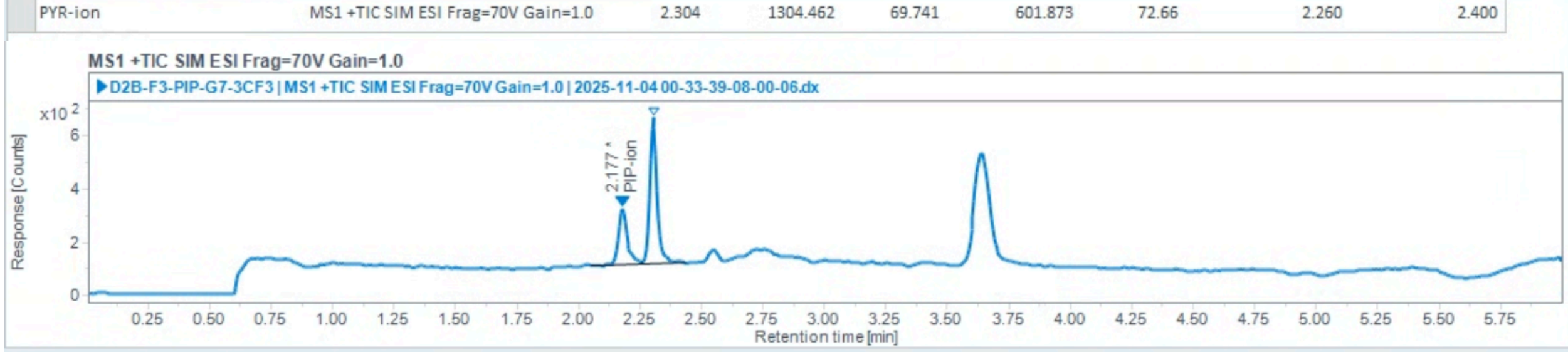

Injection Results

Peaks Summary

Name	Signal description	RT (min)	Area (Counts·s)	Area%	Height (Counts)	Height%	Start time (min)	End time (min)
PIP-ion	MS1 +TIC SIM ESI Frag=70V Gain=1.0	2.177	513.164	29.449	206.349	27.49	2.052	2.260
PYR-ion	MS1 +TIC SIM ESI Frag=70V Gain=1.0	2.303	1229.363	70.551	544.236	72.51	2.260	2.431

Regioselectivity analysis of **2l** using ***Tm*Pgb-PIP-G7-5448** (reverse phase HPLC-MS on C18 column):

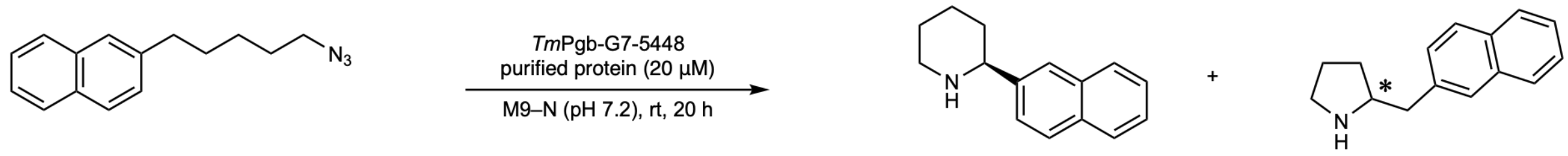

1l **2l** **3l**

	**2l**	**3l**	rr (**2l**:**3l**)	Average rr (**2l**:**3l**)
1	14285.0	7582.3	65.3:34.7	
2	12279.9	5893.3	67.6:32.4	66:34
3	13184.9	7277.3	64.4:35.6	

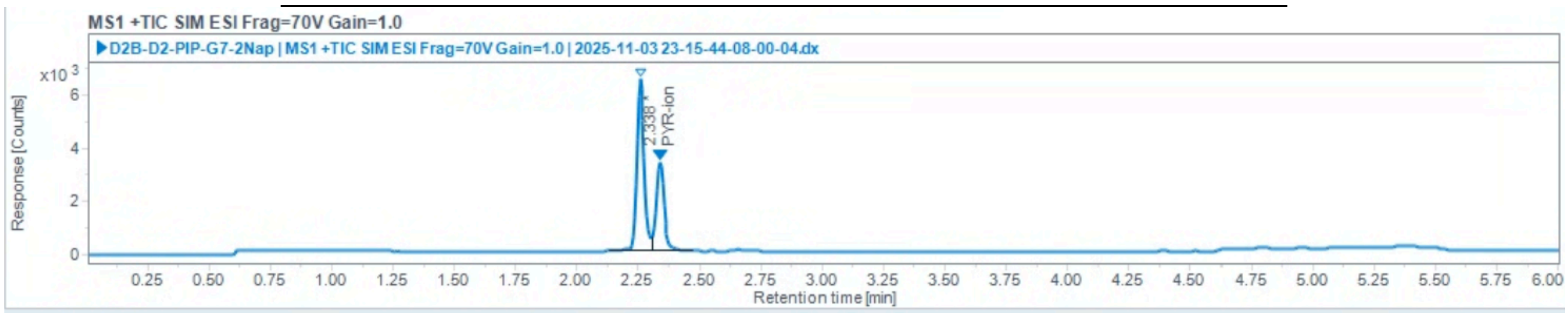

Injection Results

Peaks Summary

Name	Signal description	RT (min)	Area (Counts·s)	Area%	Height (Counts)	Height%	Start time (min)	End time (min)
PIP-ion	MS1 +TIC SIM ESI Frag=70V Gain=1.0	2.257	14285.011	65.326	6486.467	65.84	2.131	2.306
PYR-ion	MS1 +TIC SIM ESI Frag=70V Gain=1.0	2.338	7582.329	34.674	3365.907	34.16	2.306	2.470

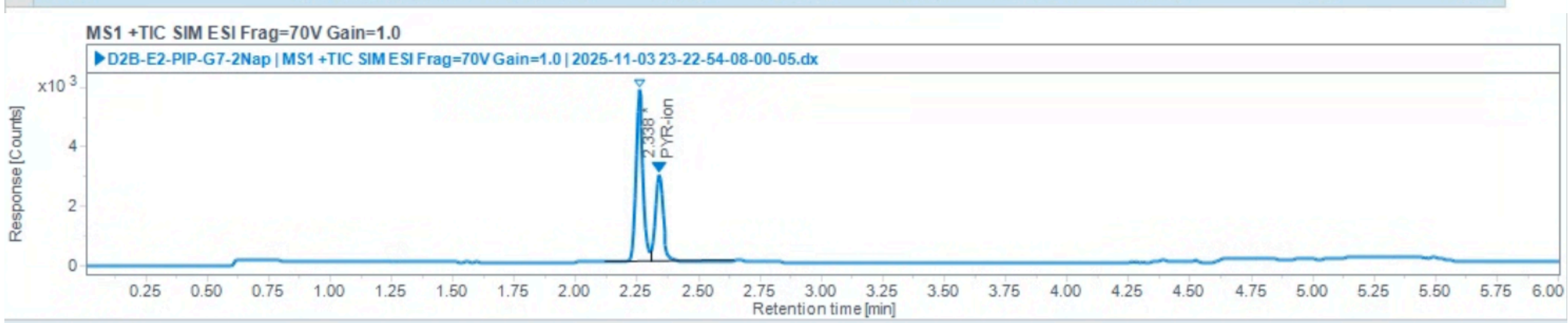

Injection Results

Peaks Summary

Name	Signal description	RT (min)	Area (Counts·s)	Area%	Height (Counts)	Height%	Start time (min)	End time (min)
PIP-ion	MS1 +TIC SIM ESI Frag=70V Gain=1.0	2.258	12279.940	67.572	5812.029	66.53	2.120	2.306
PYR-ion	MS1 +TIC SIM ESI Frag=70V Gain=1.0	2.338	5893.267	32.428	2923.298	33.47	2.306	2.645

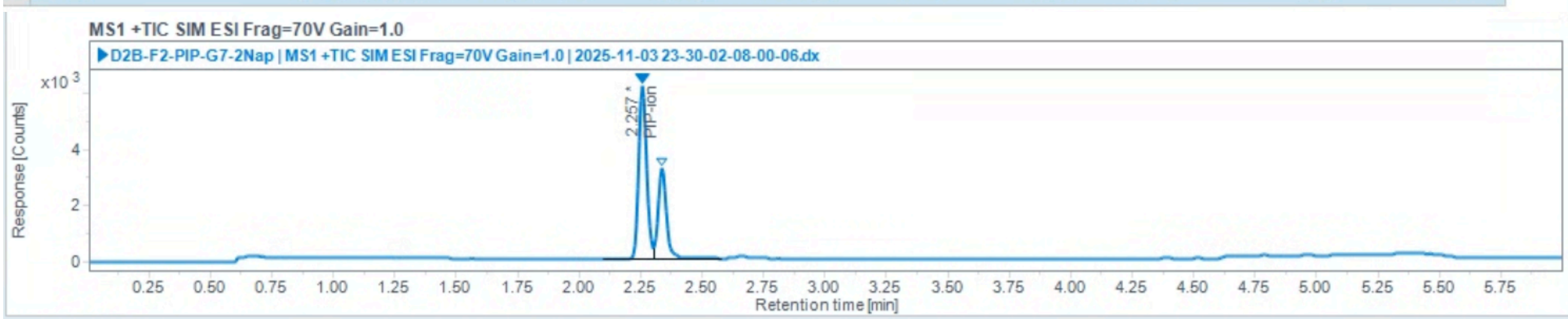

Injection Results

Peaks Summary

Name	Signal description	RT (min)	Area (Counts·s)	Area%	Height (Counts)	Height%	Start time (min)	End time (min)
PIP-ion	MS1 +TIC SIM ESI Frag=70V Gain=1.0	2.257	13184.880	64.435	6147.658	65.56	2.101	2.304
PYR-ion	MS1 +TIC SIM ESI Frag=70V Gain=1.0	2.338	7277.280	35.565	3229.100	34.44	2.304	2.581

Regioselectivity analysis of **2n** using **GDM-NT-401** (reverse phase HPLC-MS on C18 column):

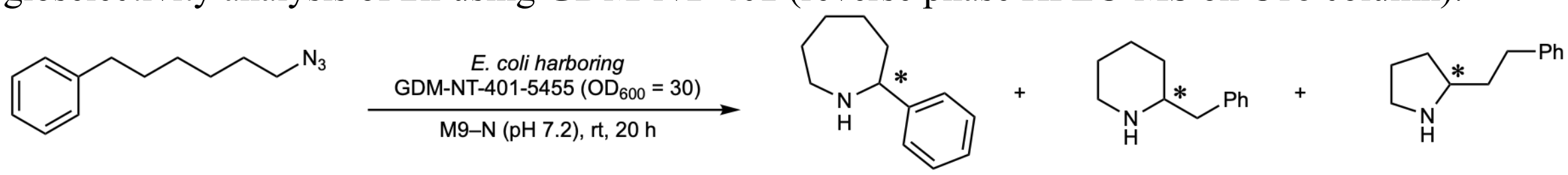

1n **2n** **3n** **4n**

89:8:3 rr (**2n:3n:4n**)

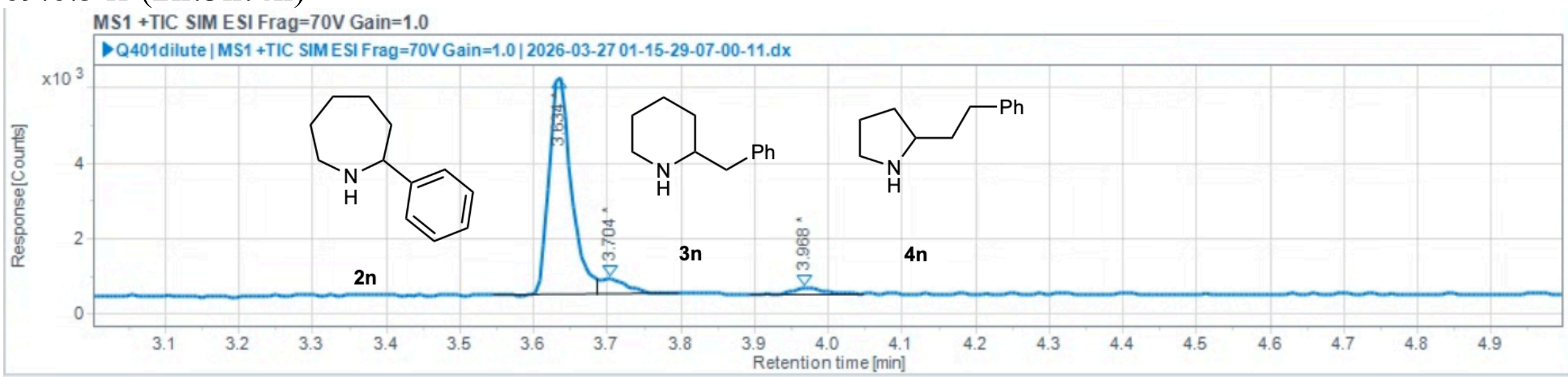

Injection Results

Peaks Summary

#	Name	Signal description	RT (min)	Area (Counts·s)	Area%	Height (Counts)	Height%	Amou	Concentra	Start time (
1		MS1 +TIC SIM ESI Frag=70V Gain	3.634	12083.326	88.828	5834.231	91.13			3.546
2		MS1 +TIC SIM ESI Frag=70V Gain	3.704	1059.616	7.790	399.680	6.24			3.687
3		MS1 +TIC SIM ESI Frag=70V Gain	3.968	460.180	3.383	168.116	2.63			3.895

Overlaied view of reaction mixture (orange, obtained using **GDM-NT-401-5455**), 2-phenylazepane (**2n**, purple, commercially available standard), 2-benzylpiperidine (**3n**, cyan, commercially available standard), 2-phenylethylpyrrolidine (**4n**, blue, commercially available standard),

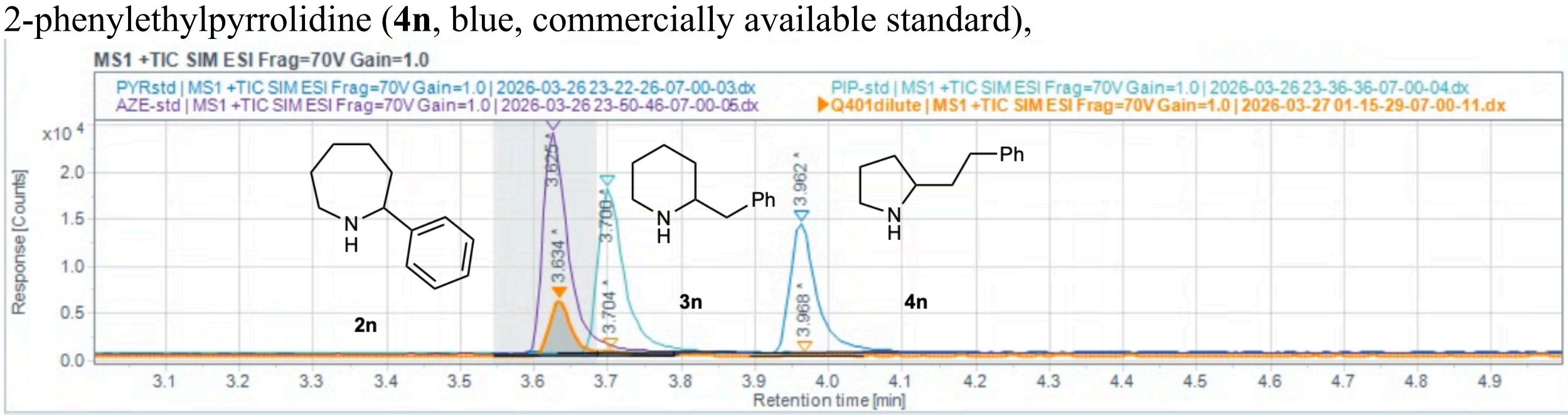

Injection Results

Peaks Summary

#	Name	Signal description	RT (min)	Area (Counts·s)	Area%	Height (Counts)	Height%	Amou	Concentra	Start time (
1		MS1 +TIC SIM ESI Frag=70V Gain	3.634	12083.326	88.828	5834.231	91.13			3.546
2		MS1 +TIC SIM ESI Frag=70V Gain	3.704	1059.616	7.790	399.680	6.24			3.687
3		MS1 +TIC SIM ESI Frag=70V Gain	3.968	460.180	3.383	168.116	2.63			3.895

Computational Details and Additional Computational Results

Computational Details - DFT calculations

All density functional theory (DFT) calculations were carried out using the Gaussian 16 progrm (*15*) . Geometries of intermediates and transition states were optimized in the gas phase using the dispersion-corrected (*16–17*) B3LYP-D3(BJ) functional (*18–19*) with a mixed basis set of SDD (*20*) (*i.e.*, the Stuttgart/Dresden effective core potentials and the associated valence electron basis set) for Fe, and 6-31G(d) for other atoms. Vibrational frequency calculations were performed to verify each optimised structure is a minimum (no imaginary frequencies) or a transition state (exactly one imaginary frequency). Thermal corrections to Gibbs free energies were calculated at 298 K. Single-point energy calculations were carried out using the B3LYP-D3(BJ) functional with the def2-TZVP basis set (*21–22*) for all atoms. Solvation energy corrections were calculated in single point energy calculations using the SMD solvation model (*23*) in diethyl ether to mimic the dielectric constant of enzyme active site (*24*). Wavefunction stability of all structures was confirmed using the "stable=opt" keyword. The bond dissociation enthalpies (BDEs) of δ-C–H and ε-C–H of substrate **1a** were calculated at the M06-2X (*25*)/def2-TZVP level theory in the gas phase, the same level of theory used in the ALFABET BDE database (*26*). CYLview (*27*) was used to visualize 3D structures of DFT-computed complexes and transition states.

DFT-computed reaction energy profiles with a 5-methylimidazole axial ligand

Reaction energy profiles of the δ- and ε-C–H amination were calculated using a model iron porphyrin complex with a 5-methylimidazole (Im) axial ligand and azide **1a** as the model substrate. The reaction starts with an iron–azide complex **CP1**, which undergoes nitrogen elimination through transition state **TS1** to generate the iron–nitrene species **INT1** with an energy barrier of 25.4 kcal/mol. Multiple conformers of the iron–nitrene species **INT1** were located, including reactive conformers **RC2a** and **RC2b**, in which the ε- and δ-C–H bonds point towards the nitrene nitrogen, poised for 1,6- and 1,5-hydrogen atom transfer (HAT), respectively. From reactive conformer **RC2b**, 1,5-HAT occurs through **TS2b** with an energy barrier of 17.3 kcal/mol to generate the homobenzylic radical species **INT2b**. The reactive conformer for 1,6-HAT (**RC2a**), which places the ε-C–H in proximity to the nitrene nitrogen, is 0.2 kcal/mol less stable than **RC2b**. From **RC2a**, 1,6-HAT occurs through **TS2a** to generate the benzylic radical species **INT2a**. From the radical species **INT2a** and **INT2b**, C–N radical rebound through **TS3a** and **TS3b** forms the piperidine complex **P2a** and pyrrolidine complex **P2b**, respectively. The C–N radical rebound steps are both highly exergonic and irreversible. In the 1,6- and 1,5-HAT pathways, the Gibbs free energies of the C–N radical rebound transition states (**TS3a** and **TS3b**) is 6.2 and 3.0 kcal/mol lower than those of the HAT transition states (**TS2a** and **TS2b**). These results indicate that the HAT in both pathways are irreversible and thus regioselectivity-determining. Because of the relatively high barriers to the subsequent HAT, the alkyl chain conformational change of the Fe–nitrene intermediate is expected to occur much faster. Under these Curtin–Hammett conditions, the regioselectivity of the HAT is determined by the energy difference between the competing HAT transition states (**TS2a** and **TS2b**, $\Delta\Delta G^{\ddagger}$ = 2.7 kcal/mol). The overall barriers to the 1,6- and 1,5-HAT were calculated with respect to the lowest-energy conformer (**RC2b**): $\Delta G^{\ddagger}$ = 14.6 and 17.3 kcal/mol for 1,6-HAT (**TS2a**) and 1,5-HAT (**TS2b**), respectively.

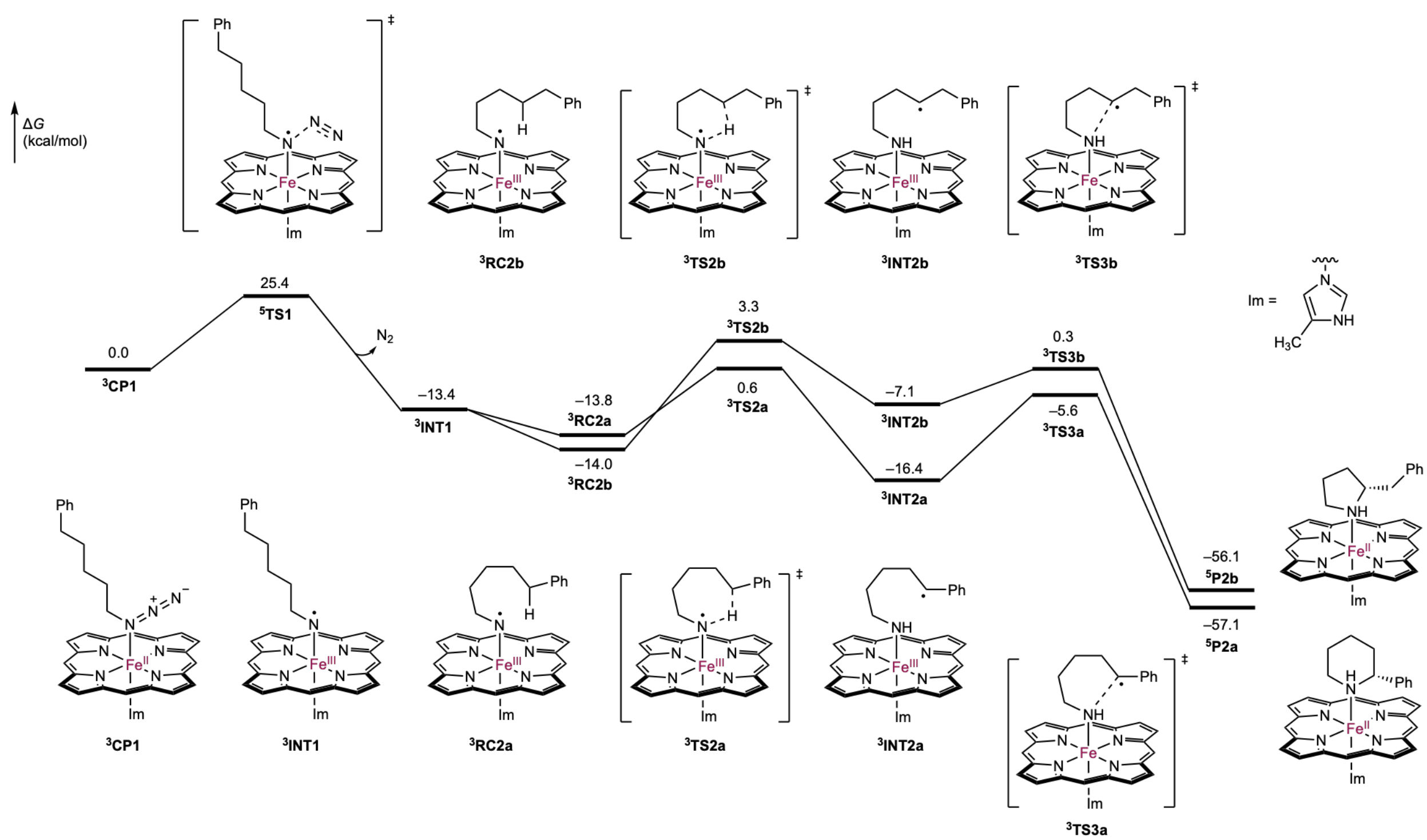

Figure S12. DFT-computed reaction energy profiles with a 5-methylimidazole axial ligand.

Spin state energies of Fe–porphyrin complexes with a 5-methylimidazole axial ligand

Singlet, triplet, quintet, and septet spin states of intermediates **CP1**, **INT1**, **RC2a**, **RC2b**, **INT2a**, **INT2b**, **P2a**, and **P2b** were located. For the singlet state of each species, both closed-shell singlet and open-shell singlet from broken-symmetry DFT calculations were calculated. For **CP1**, **INT1**, **RC2b**, **P2a**, and **P2b**, the closed-shell singlet wavefunction was stable and no open-shell singlet could be obtained, whereas open-shell singlet was obtained for **RC2a** and **INT2a**. Attempts to locate the singlet of the homobenzylic radical **INT2b** were not successful. Triplet was found to be the most stable spin state for **CP1**, **INT1**, **RC2a**, **RC2b**, **INT2a**, **INT2b**, **TS2a**, **TS2b**, **TS3a**, and **TS3b**. Quintet was found to be the most stable spin state for **TS1**, the piperidine product **P2a** and pyrrolidine product **P2b**. Although no spin crossover was observed for HAT in our study, the relatively small energy differences between triplet and quintet states for both 1,6- and 1,5-HAT transition states (**TS2a** and **TS2b**) are consistent with the previous computational study from Mondal that indicated a small triplet-quintet energy gap at the HAT transition states (*28*).

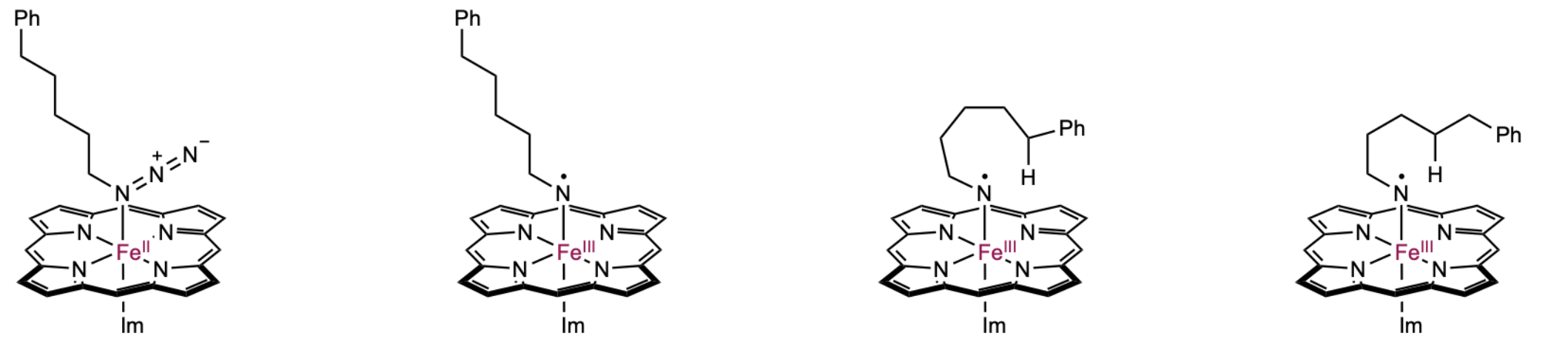

CP1

Spin State	ΔG (kcal/mol)
Closed-shell singlet	6.4
Triplet	0.0
Quintet	0.4
Septet	24.7

INT1

Spin State	ΔG (kcal/mol)
Closed-shell singlet	11.5
Triplet	0.0
Quintet	6.7
Septet	10.8

RC2a

Spin State	ΔG (kcal/mol)
Open-shell singlet	11.3
Triplet	0.0
Quintet	8.7
Septet	13.5

RC2b

Spin State	ΔG (kcal/mol)
Closed-shell singlet	8.0
Triplet	0.0
Quintet	7.2
Septet	13.3

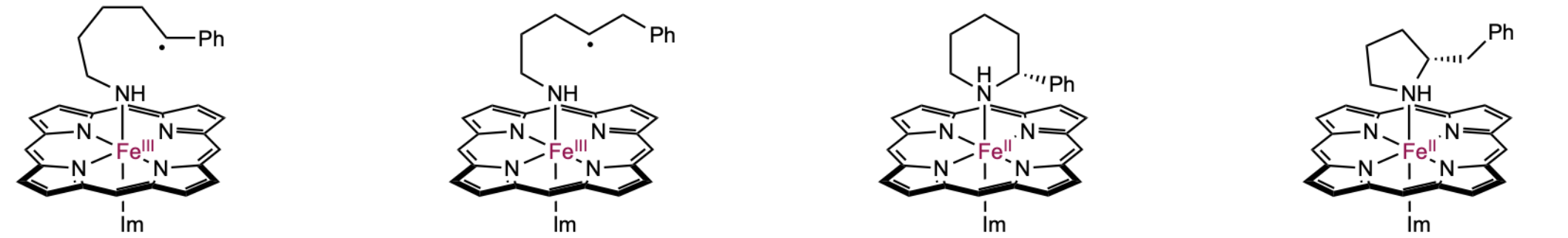

INT2a

Spin State	ΔG (kcal/mol)
Open-shell singlet	23.0
Triplet	0.0
Quintet	12.0
Septet	3.5

INT2b

Spin State	ΔG (kcal/mol)
Singlet	not located
Triplet	0.0
Quintet	20.4
Septet	3.4

P2a

Spin State	ΔG (kcal/mol)
Closed-shell singlet	5.4
Triplet	0.9
Quintet	0.0
Septet	23.0

P2b

Spin State	ΔG (kcal/mol)
Closed-shell singlet	1.4
Triplet	1.0
Quintet	0.0
Septet	21.7

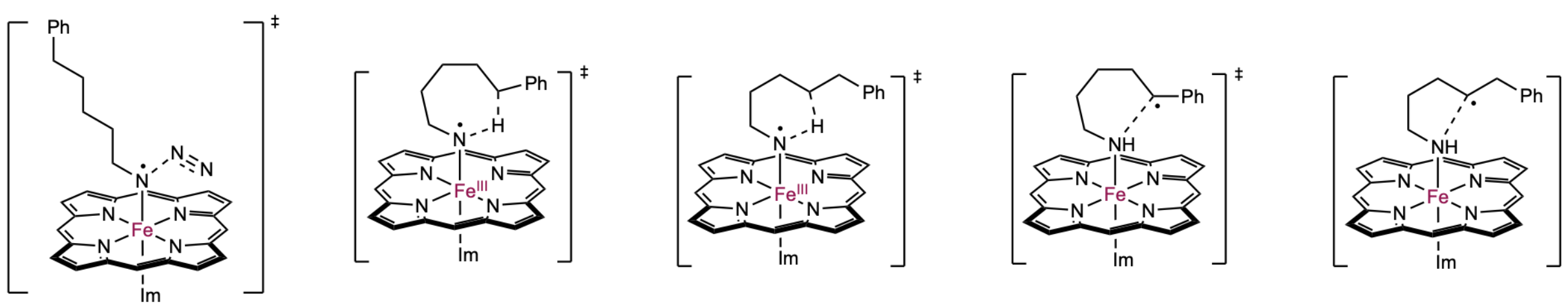

TS1

Spin State	ΔG (kcal/mol)
Closed-shell singlet	5.3
Triplet	0.4
Quintet	0.0
Septet	6.4

TS2a

Spin State	ΔG (kcal/mol)
Closed-shell singlet	8.9
Triplet	0.0
Quintet	0.3
Septet	6.8

TS2b

Spin State	ΔG (kcal/mol)
Closed-shell singlet	8.5
Triplet	0.0
Quintet	0.9
Septet	6.9

TS3a

Spin State	ΔG (kcal/mol)
Singlet	not located
Triplet	0.0
Quintet	2.6
Septet	8.0

TS3b

Spin State	ΔG (kcal/mol)
Singlet	not located
Triplet	0.0
Quintet	10.4
Septet	10.2

Figure S13. Spin state energies of intermediates and transition states in the C–H amination catalyzed by a model Fe–porphyrin complex with a 5-methylimidazole (Im) axial ligand. Gibbs free energies are respect to the most stable spin state of each structure.

DFT-computed reaction energy profiles with a thioether axial ligand

To model the reaction mechanism with the *de novo* designed **GDM-NT-401**, which has a methionine axial ligand, we computed the reaction energy profiles of the δ- and ε-C–H amination using a model iron porphyrin complex with a ethyl methyl thioether axial ligand and azide **1a** as the model substrate.

The reaction starts with an iron–azide complex **CP1'**, which undergoes nitrogen elimination through transition state **TS1'** to generate the iron–nitrene species **INT1'** with an energy barrier of 24.3 kcal/mol. Multiple conformers of the iron–nitrene species **INT1'** were located, including reactive conformers **RC2a'** and **RC2b'**, in which the ε- and δ-C–H bonds point towards the nitrene nitrogen, poised for 1,6- and 1,5-hydrogen atom transfer (HAT), respectively. From reactive conformer **RC2b'**, 1,5-HAT occurs through **TS2b'** with an energy barrier of 16.5 kcal/mol to generate the homobenzylic radical species **INT2b'**. The reactive conformer for 1,6-HAT (**RC2a'**), which places the ε-C–H in proximity to the nitrene nitrogen, is 0.9 kcal/mol less stable than **RC2b'**. From **RC2a'**, 1,6-HAT occurs through **TS2a'** to generate the benzylic radical species **INT2a'**. From the radical species **INT2a'** and **INT2b'**, C–N radical rebound through **TS3a'** and **TS3b'** forms the piperidine complex **P2a'** and pyrrolidine complex **P2b'**, respectively. The C–N radical rebound steps are both highly exergonic and irreversible. In the 1,6- and 1,5-HAT pathways, the Gibbs free energies of the C–N radical rebound transition states (**TS3a'** and **TS3b'**) is 0.3 and 4.2 kcal/mol lower than

those of the HAT transition states (**TS2a'** and **TS2b'**). These results indicate that the HAT in both pathways are irreversible and thus regioselectivity-determining. Because of the relatively high barriers to the subsequent HAT, the alkyl chain conformational change of the Fe–nitrene intermediate is expected to occur much faster. Under these Curtin–Hammett conditions, the regioselectivity of the HAT is determined by the energy difference between the competing HAT transition states (**TS2a'** and **TS2b'**, $\Delta\Delta G^{\ddagger}$ = 3.2 kcal/mol). The overall barriers to the 1,6- and 1,5-HAT were calculated with respect to the lowest-energy conformer (**INT1'**): $\Delta G^{\ddagger}$ = 13.3 and 16.5 kcal/mol for 1,6-HAT (**TS2a'**) and 1,5-HAT (**TS2b'**), respectively.

The DFT calculations indicated that the reaction catalyzed by the Fe–porphyrin catalyst with the thioether axial ligand occurs via the same mechanism as that with the 5-methylimidazole axial ligand, and the computed regioselectivity is not significantly affected by the axial ligand ($\Delta\Delta G^{\ddagger}$ = 3.2 and 2.7 kcal/mol, respectively). These results, along with the classical MD simulations in this study, indicate that the enhanced regioselectivity for the *de novo* designed **GDM-NT-401** compared with the evolved ***Tm*Pgb-PIP-G7** is primarily due to the different active site pocket, rather than the electronic effects of the axial ligand.

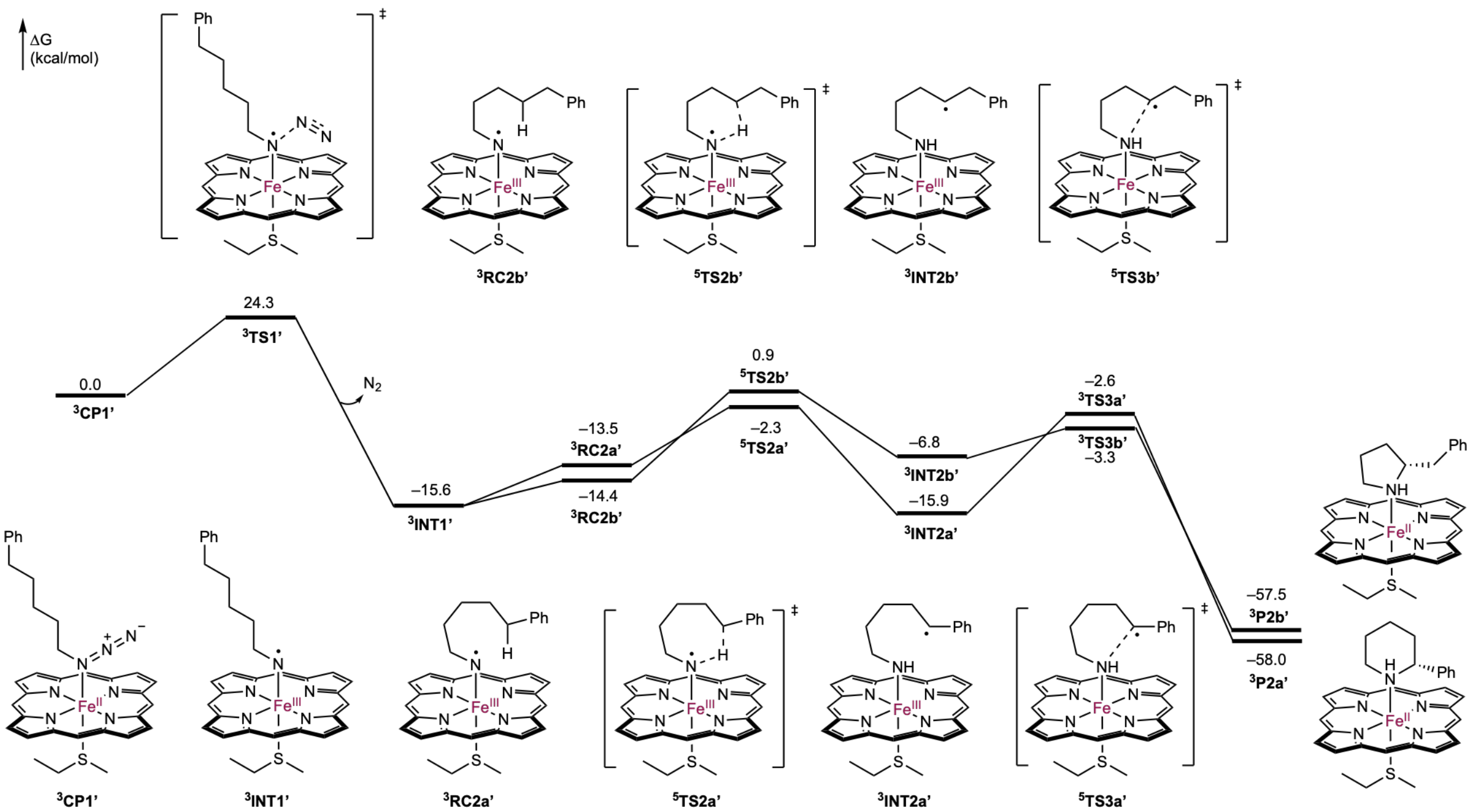

Figure S14. DFT-computed reaction energy profiles with a thioether axial ligand.

Spin state energies of Fe–porphyrin complexes with a thioether axial ligand

Singlet, triplet, quintet, and septet spin states of intermediates **CP1'**, **INT1'**, **RC2a'**, **RC2b'**, **P2a'**, and **P2b'** were located. For the singlet state of each species, both closed-shell singlet and open-shell singlet from broken-symmetry DFT calculations were calculated. For **CP1'**, **INT1'**, **RC2b'**, **RC2a', P2a'**, and **P2b'**, the closed-shell singlet wavefunction was stable and no open-shell singlet could be obtained. Attempts to locate the singlet of the benzylic radical **INT2a'** and the homobenzylic radical **INT2b'** were not successful. Triplet was found to be the most stable spin state for **CP1'**, **INT1'**, **RC2a'**, **RC2b'**, **INT2a'**, **INT2b'**, **P2a'**, and **P2b'**. Quintet was found to be the most stable spin state for the 1,6- and 1,5-HAT transition states (**TS2a'** and **TS2b'**). The spin crossover prior to HAT is consistent with the previous computational study reported by Mondal with a neutral methanethiol axial ligand (*28*).

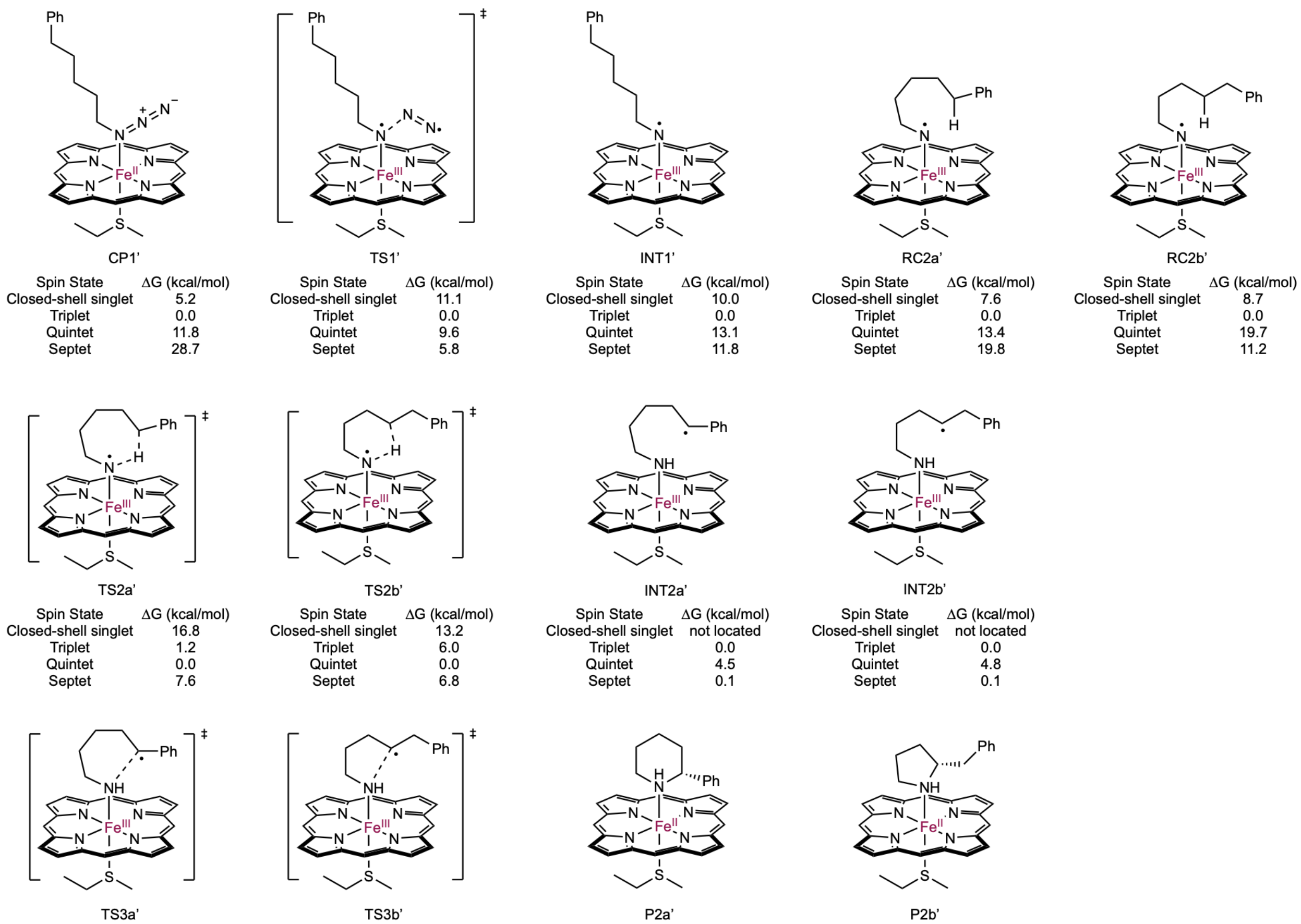

Spin State	CP1' ΔG (kcal/mol)	TS1' ΔG (kcal/mol)	INT1' ΔG (kcal/mol)	RC2a' ΔG (kcal/mol)	RC2b' ΔG (kcal/mol)
Closed-shell singlet	5.2	11.1	10.0	7.6	8.7
Triplet	0.0	0.0	0.0	0.0	0.0
Quintet	11.8	9.6	13.1	13.4	19.7
Septet	28.7	5.8	11.8	19.8	11.2

Spin State	TS2a' ΔG (kcal/mol)	TS2b' ΔG (kcal/mol)	INT2a' ΔG (kcal/mol)	INT2b' ΔG (kcal/mol)
Closed-shell singlet	16.8	13.2	not located	not located
Triplet	1.2	6.0	0.0	0.0
Quintet	0.0	0.0	4.5	4.8
Septet	7.6	6.8	0.1	0.1

Spin State	TS3a' ΔG (kcal/mol)	TS3b' ΔG (kcal/mol)	P2a' ΔG (kcal/mol)	P2b' ΔG (kcal/mol)
Closed-shell singlet	not located	not located	5.1	5.0
Triplet	0.0	0.0	0.0	0.0
Quintet	5.5	13.9	4.3	0.0
Septet	7.0	11.9	25.9	25.1

Figure S15. Spin state energies of intermediates and transition states in the C–H amination catalyzed by a model Fe–porphyrin complex with a thioether axial ligand. Gibbs free energies are respect to the most stable spin state of each structure.

Computational Details – Classical molecular dynamics simulations

Classical molecular dynamics (MD) simulations were performed to investigate active site residue–substrate interactions with the Fe–nitrene intermediate prior to the HAT. The Fe–nitrene derived from azide **1a** was used in the classical MD simulations. The starting structures of the alkyl nitrene intermediate in ***Tm*Pgb-PIP-G7** and **GDM-NT-401** were obtained from co-folding models of each protein with the haem–nitrene ligand, generated using Chai-1 (*29*) with the protein sequence given in one-letter code and the haem–nitrene ligand specified by the following SMILES string: [CH2]=[CH][C]1=[C]2[CH]=[C]3[C]([CH3])=[C]([CH]=[CH2])[C]4=[N]3[Fe+4]35([N]6=[C]([C]([CH2][CH2][C]([OH])=[O])=[C]([CH3])[C]6=[CH][C](=[C]1[CH3])[N]23)[CH]=[C]1[C]([CH2][CH2][C]([OH])=[O])=[C]([CH3])[C](=[CH]4)[N]51)=[N][CH2][CH2][CH2][CH2][CH2][C]1=[CH][CH]=[CH][CH]=[CH]1. Because Chai-1 returns heavy-atom coordinates only, hydrogen atoms were added using the H++ server (*30*), and the resulting complex was used as the starting structure for the MD simulations described below.

Classical MD simulations were carried out using the pmemd module (*31*) of the GPU-accelerated Amber 24 software (*32*). The Amber ff19SB force field (*33*) was used in all classical MD simulations. Force field parameters for Fe and surrounding atoms and bonds in the haem–nitrene complex were generated using the MCPB.py module (*34*). The second-generation general Amber force field (gaff2) (*35*) was used for other atoms in the haem–nitrene intermediate. Using the Merz–Singh–Kollman scheme (*36*–*37*), RESP

charge fitting (*38*) on electrostatic potential generated at the B3LYP/6-31G(d) level of theory was performed to generate partial charges at triplet spin state for the Fe–nitrene. The enzyme was then put into a solvated cuboid box under periodic boundary conditions using the TIP3P water model (*39*). The minimum distance between the enzyme surface and the edge of the water box was set to 10 Å. Water molecules were treated with the SHAKE algorithm (*40*). The system was neutralized by adding Na^+ counterions. Long-range electrostatics were calculated using the particle-mesh-Ewald method (*41*). Lennard-Jones and electrostatic interaction cut-offs were set to 12 Å.

The classical MD simulations were performed in several steps. Three independent replicas of 500 ns MD simulations were performed for the Fe–nitrene intermediate of the ***Tm*Pgb-PIP-G7** and **GDM-NT-401** variants. A 30,000-step energy minimization was first performed with positional restraints for the protein by applying a force constant of 500 $kcal \cdot mol^{-1} \cdot Å^{-2}$. Next, the system was gradually heated without any restraint from 0 K to 300 K in 200 ps, which was followed by an equilibration using the isothermal–isobaric ensemble (NPT) in the next 6 ns. Finally, 500 ns production MD simulations were performed using the same conditions as the equilibration with a time step of 2 fs without any restraint. After the MD simulations, clustering analysis was carried out using the CPPTRAJ module (*42*) to identify the most populated structure from the classical MD simulations of all three replicas. Clustering analysis was performed using the k-means algorithm (*43*) (10 clusters, maximum 500 iterations, sieving every 10th frame), with the RMSD of the protein backbone atoms (N, Cα and C) as the distance metric.

To further probe enzyme–substrate interactions at reactive geometries near the HAT transition state, restrained MD simulations of the Fe–nitrene intermediate were performed to sample the near attack conformations (NACs) (*44*) prior to HAT in ***Tm*Pgb-PIP-G7** and **GDM-NT-401**. The restrained MD simulations were performed following the same four-step protocol described above, with a harmonic distance restraint (2.9–3.5 Å, force constant 100 $kcal \cdot mol^{-1} \cdot Å^{-2}$) applied between the nitrene nitrogen and the centroid of the δ- and ε-carbon atoms. This distance was chosen according to the corresponding N···C distance in the DFT-optimized transition state structures. Three independent replicas of the restrained MD simulations were performed for each enzyme.

Unrestrained classical MD simulations of the Fe–nitrene intermediate in *Tm*Pgb-PIP-G7

To investigate the preferred binding poses of the substrate and the potential interaction modes within the active site, three independent replicas of 500 ns MD simulations were performed for the Fe–nitrene intermediate in ***Tm*Pgb-PIP-G7**. The major binding pose of the alkyl nitrene intermediate in ***Tm*Pgb-PIP-G7** is shown in the main text (Figure 5b, left). The phenyl group of the nitrene alkyl chain is accommodated in a hydrophobic pocket formed by Y60, W59 and F144, engaging in stabilising π···π and C–H···π interactions. These interactions were corroborated by analysing π···π and C–H···π distances along the trajectories. The Cβ of the alkyl chain is 4.7 ± 0.4 Å from the centroid of the aromatic ring of the Y60 side chain, indicating a favorable C–H···π interaction between the nitrene alkyl chain and Y60 (Figure S16). The centroid of the substrate phenyl ring is 5.0 ± 0.7 Å from that of the W59 pyrrole ring (Figure S17) and 4.9 ± 0.8 Å from that of the F144 phenyl ring (Figure S18), indicating stabilising π···π interactions. These persistent interactions hold the nitrene alkyl chain in an extended geometry, with most of the rotatable C(*sp*³)–C(*sp*³) bonds adopting the *trans* conformation (most populated θ_2, θ_3, $\theta_4 \approx 180°$; Figures S19–S22).

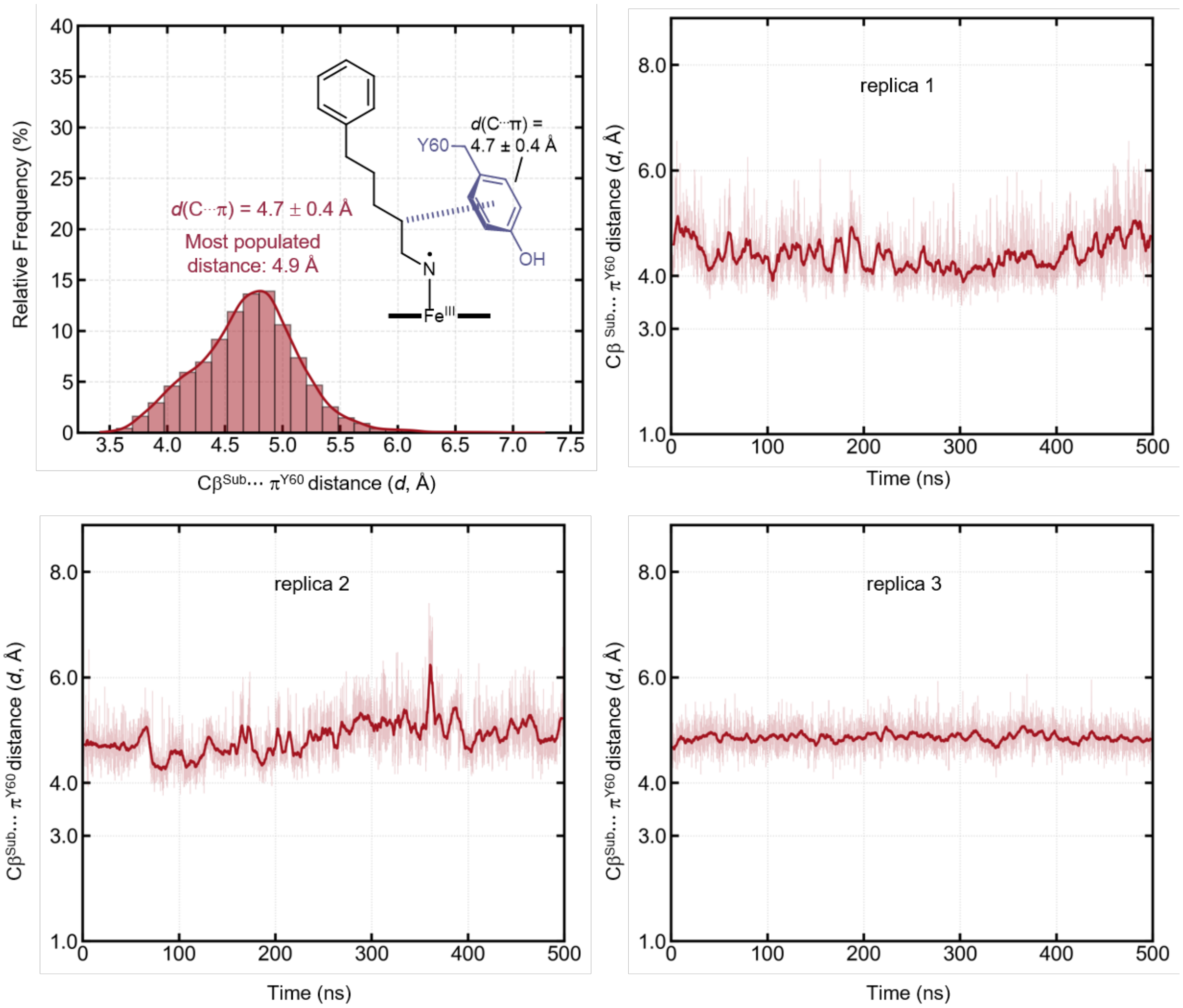

Figure S16. Distance between the centroid of the benzene ring of the Y60 side chain (π^{Y60}) and C_β atom of nitrene observed in unrestrained MD simulations with ***Tm*****Pgb-PIP-G7**.

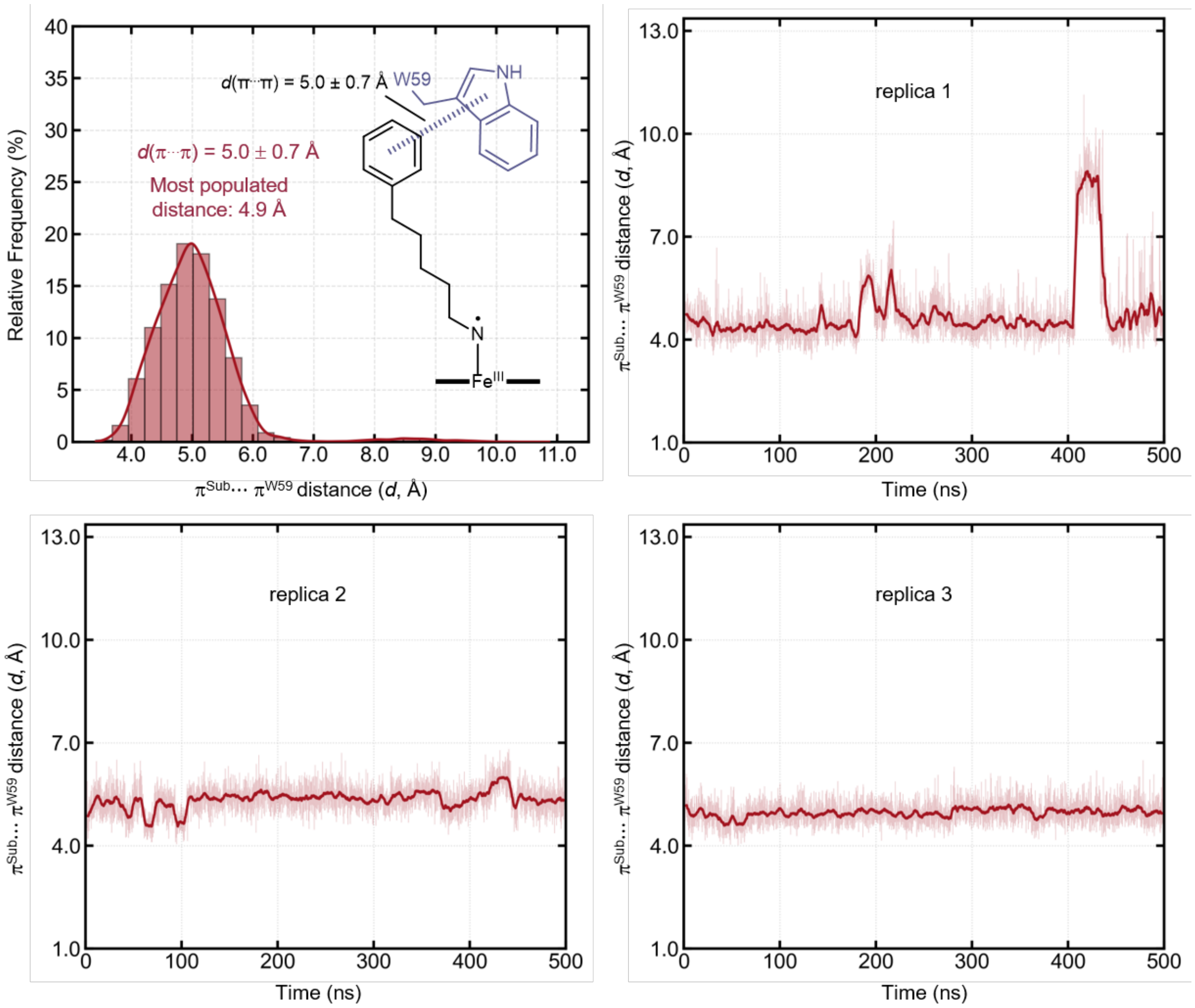

Figure S17. Distance between the centroid of the benzene of the substrate (π^{Sub}) and the centroid of the pyrrole ring of W59 side chain (π^{W59}) observed in unrestrained MD simulations with ***TmPgb*-PIP-G7**.

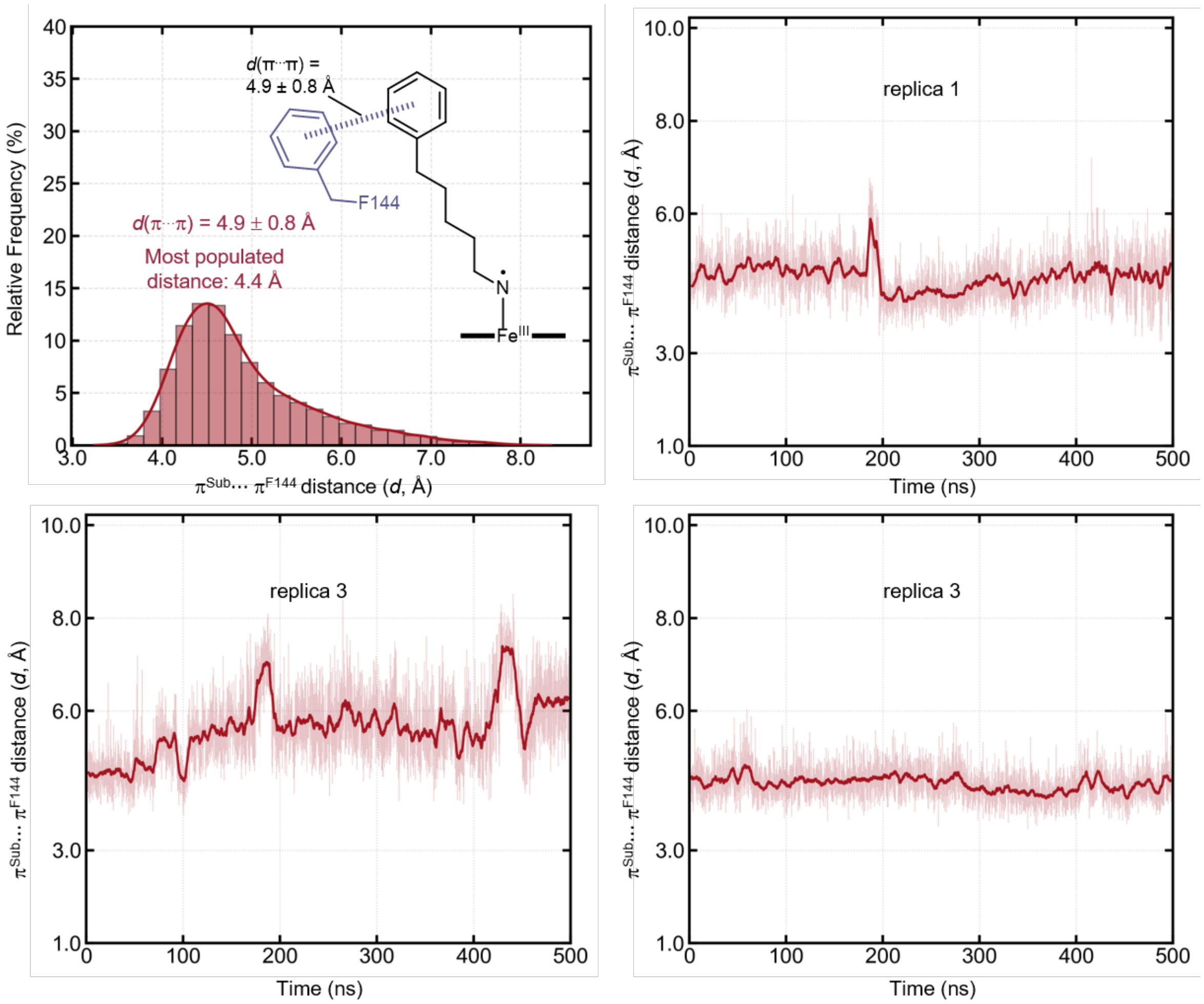

Figure S18. Distance between the centroid of the benzene ring of the substrate (π^{Sub}) and the centroid of the benzene ring of F144 side chain (π^{F144}) observed in unrestrained MD simulations with ***Tm*Pgb-PIP-G7**.

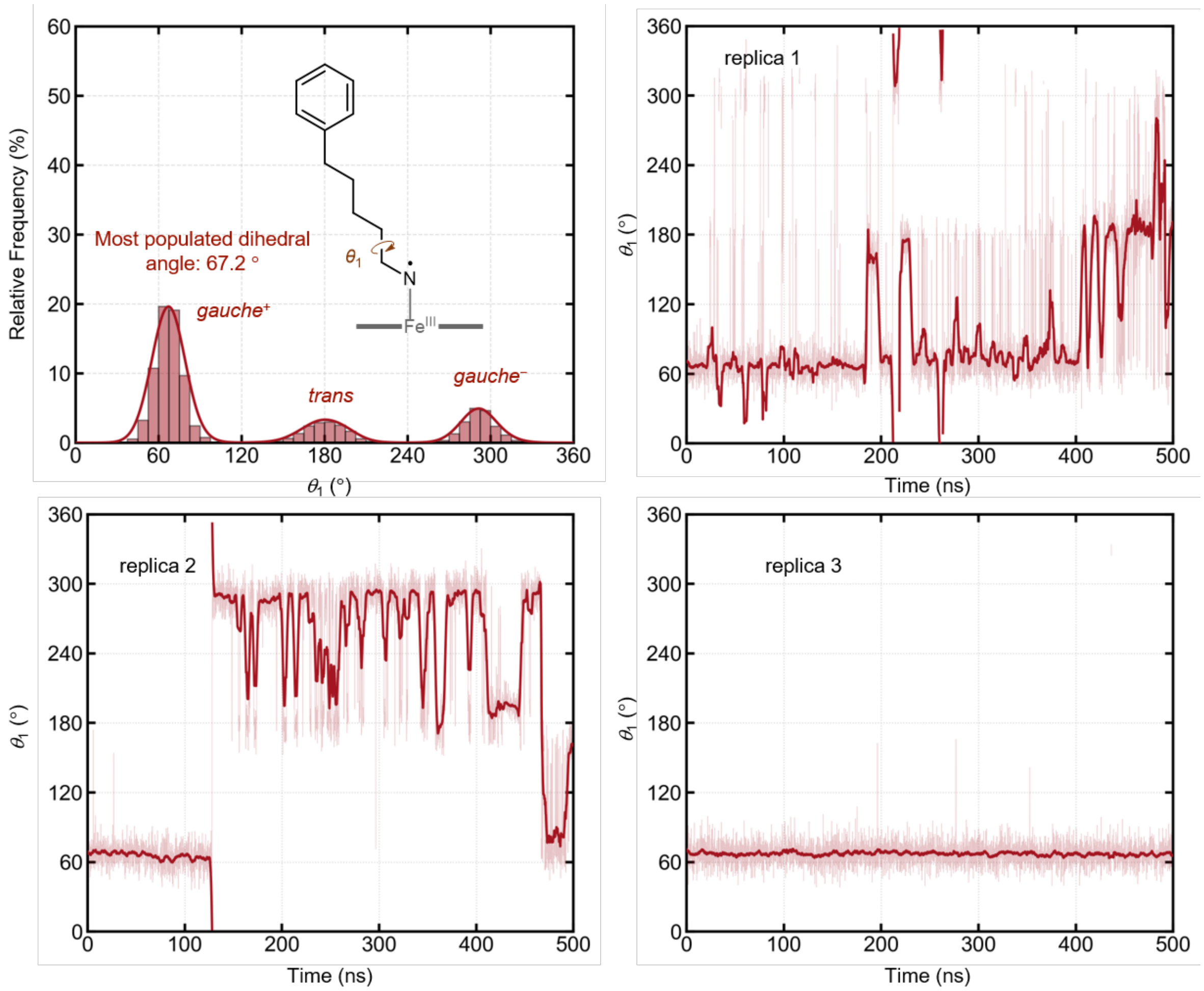

Figure S19. Dihedral angle θ_1 (NCαCβCγ) of the alkyl nitrene intermediate observed in unrestrained MD simulations with ***Tm*Pgb-PIP-G7**.

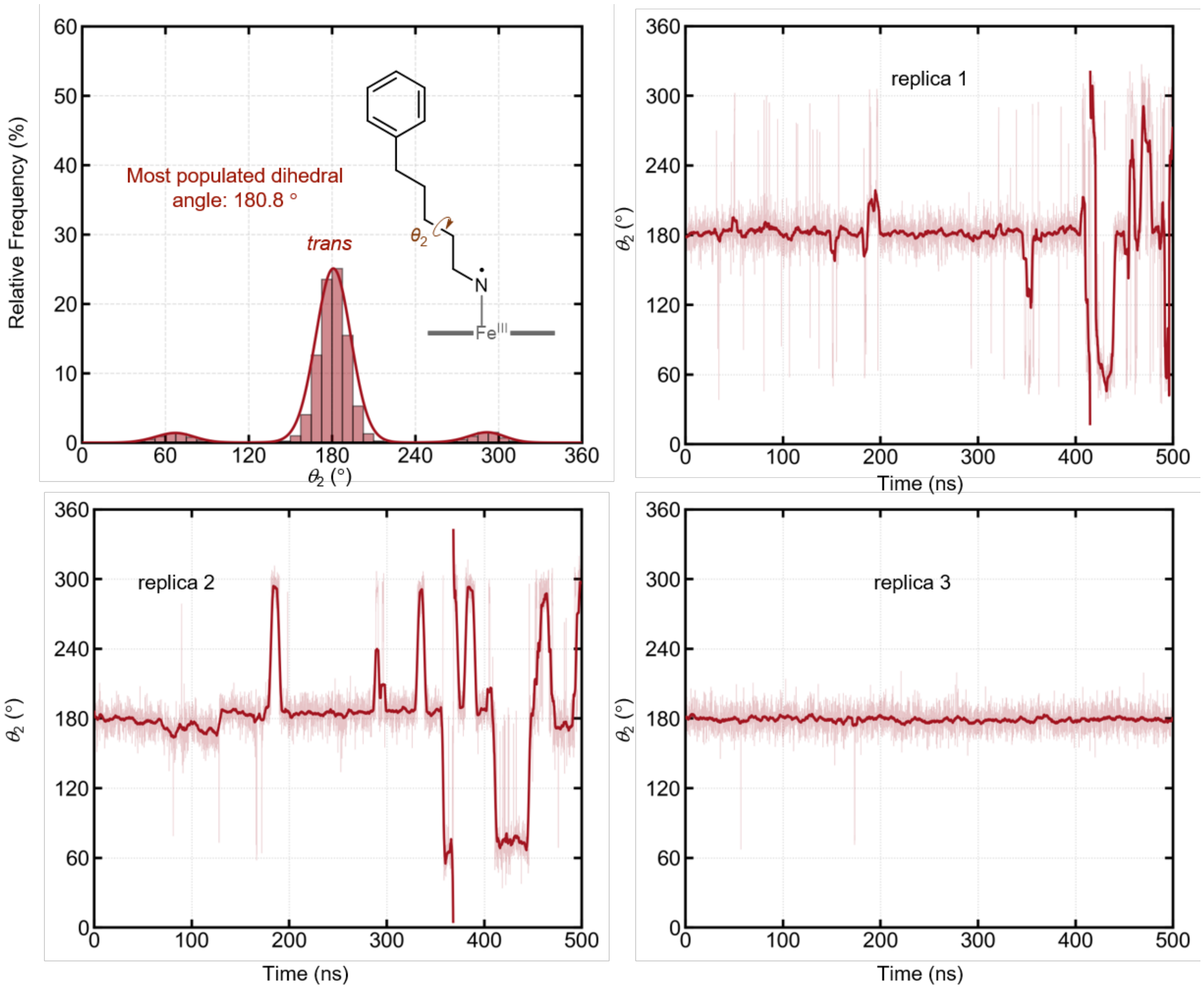

Figure S20. Dihedral angle θ_2 (CαCβCγCδ) of the alkyl nitrene intermediate observed in unrestrained MD simulations with ***Tm*Pgb-PIP-G7**.

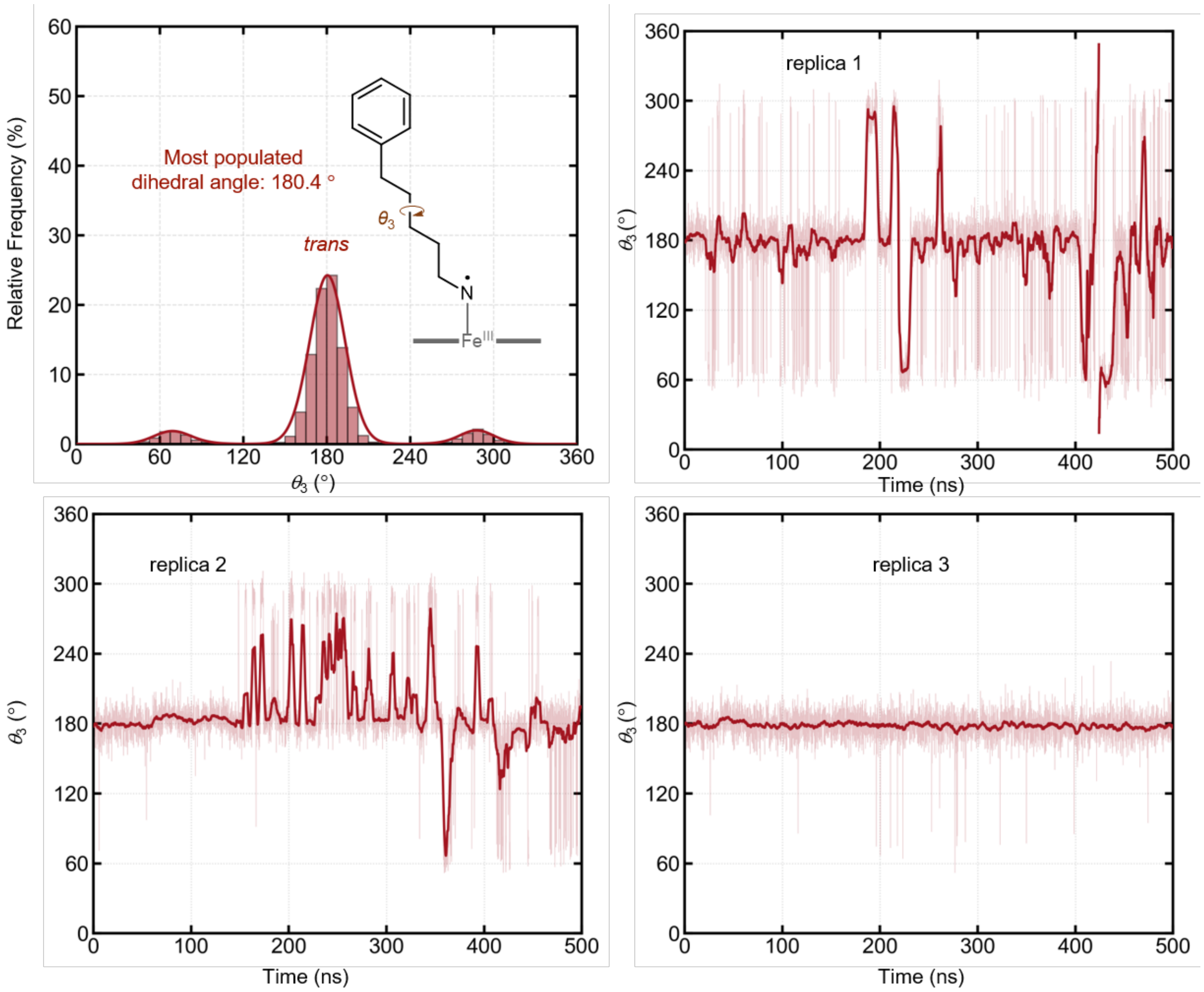

Figure S21. Dihedral angle θ_3 (CβCγCδCε) of the alkyl nitrene intermediate observed in unrestrained MD simulations with ***Tm*****Pgb-PIP-G7**.

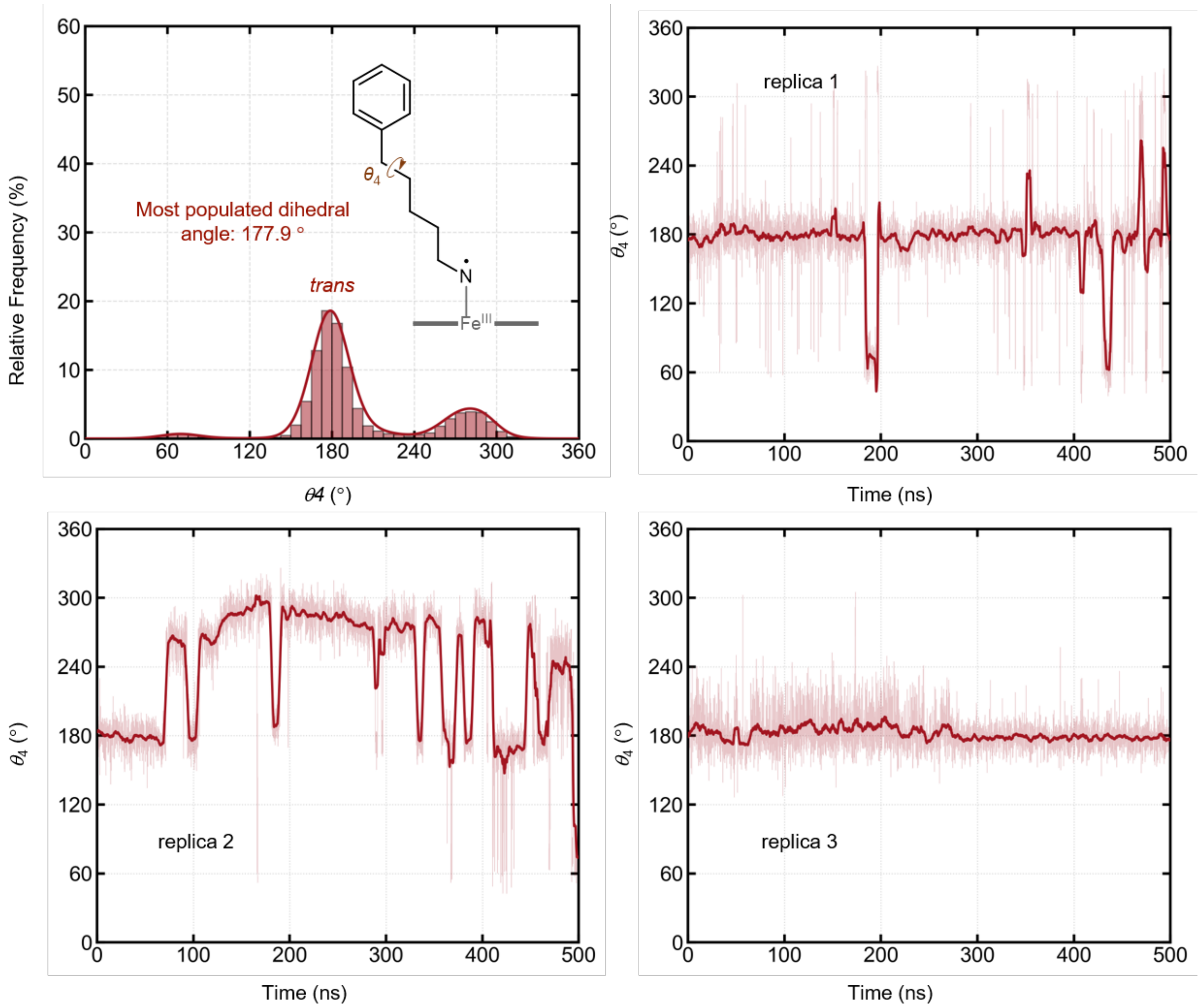

Figure S22. Dihedral angle θ_4 (CγCδCεCphenyl) of the alkyl nitrene intermediate observed in unrestrained MD simulations with ***Tm*Pgb-PIP-G7**.

Unrestrained classical MD simulations of the Fe–nitrene intermediate in GDM-NT-401

To investigate the preferred binding poses of the substrate and the interaction modes within the active site, three independent replicas of 500 ns MD simulations were performed for the Fe–nitrene intermediate in **GDM-NT-401**. The predominant binding pose of the alkyl nitrene intermediate in **GDM-NT-401** is shown in the main text (Figure 5b, right). The Cδ of L20 forms a persistent C–H···π interaction with the substrate phenyl ring, with a mean Cδ···phenyl distance of 4.0 ± 0.4 Å (Figure S23). The side chains of V28, L19, and L16 create a hydrophobic pocket that enforces a bent, reactive conformation of the alkyl chain (Figures S24–S27).

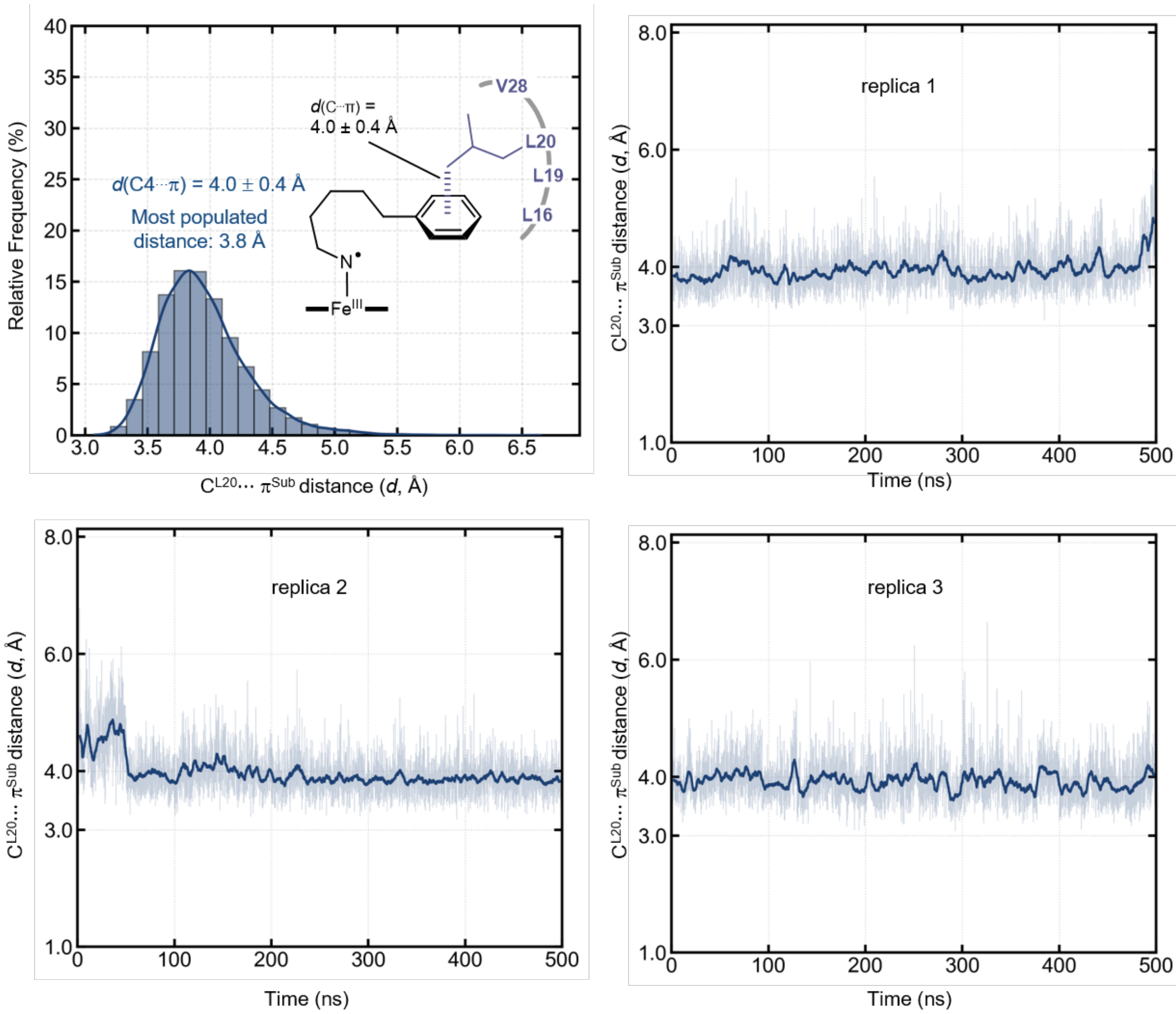

Figure S23. Distance between the centroid of the benzene of the substrate (π^{Sub}) and C_δ atom of L20 observed in unrestrained MD simulations with **GDM-NT-401**.

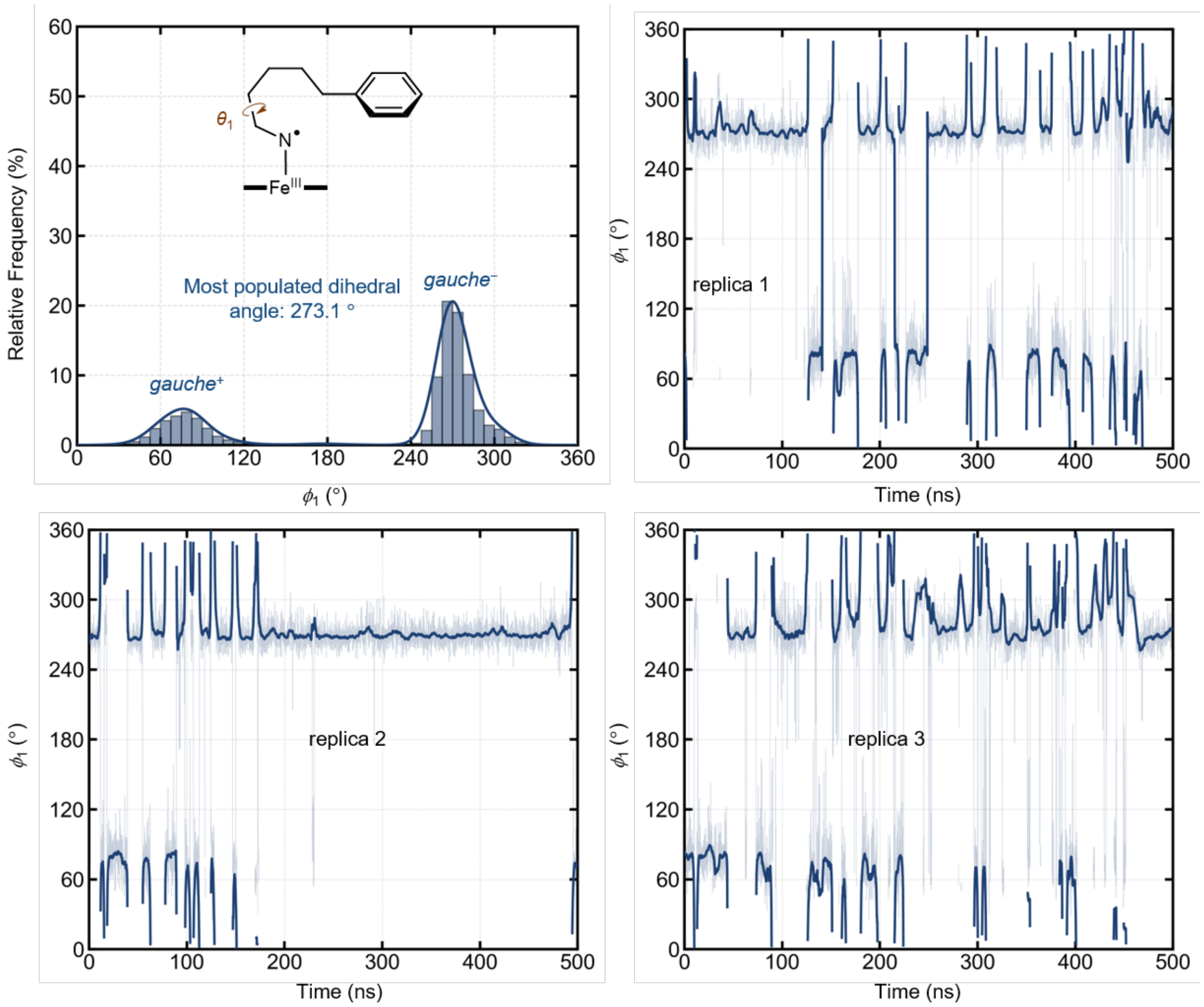

Figure S24. Dihedral angle θ_1 (NCαCβCγ) of the alkyl nitrene intermediate observed in unrestrained MD simulations with **GDM-NT-401**.

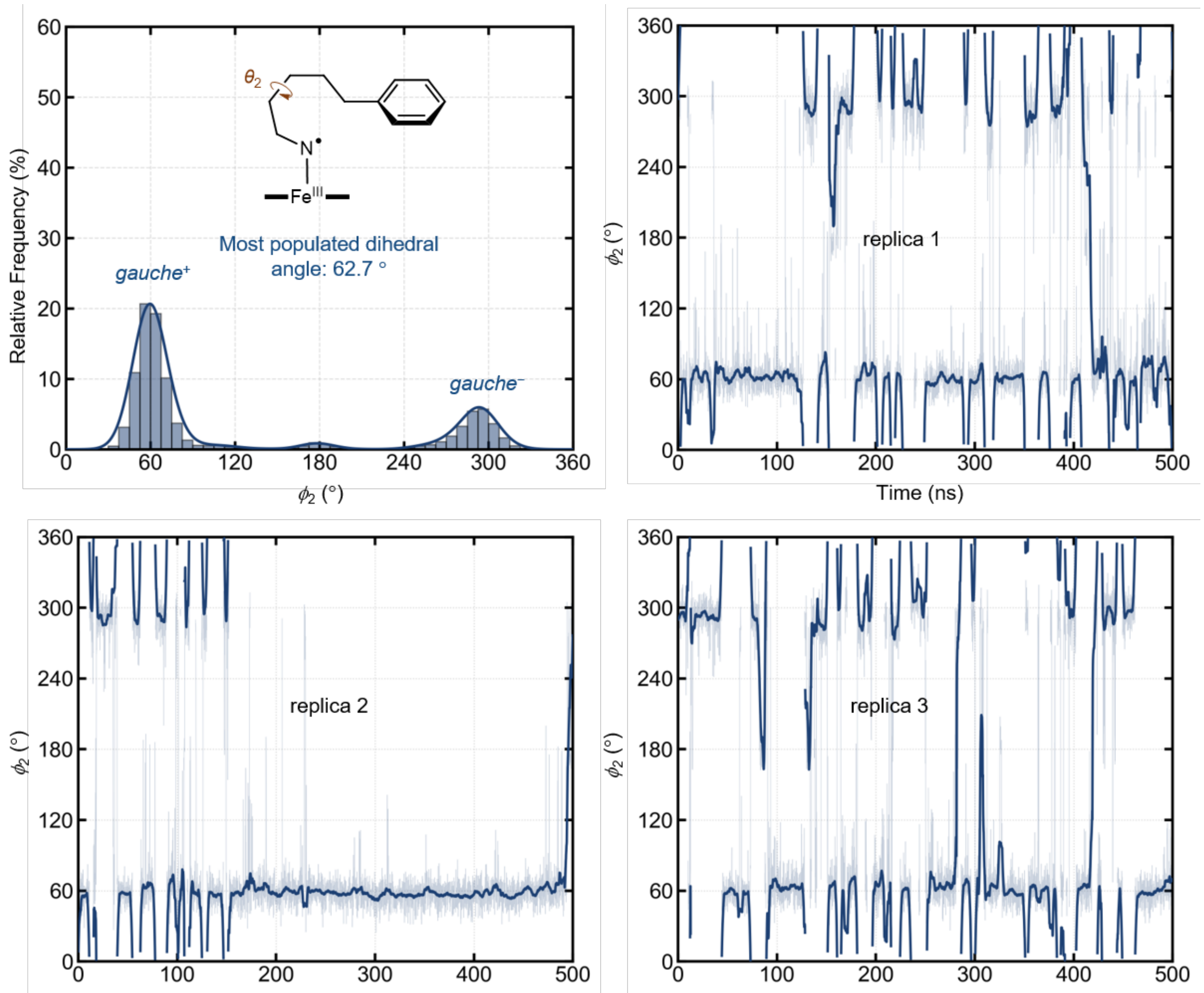

Figure S25. Dihedral angle θ_2 (CαCβCγCδ) of the alkyl nitrene intermediate observed in unrestrained MD simulations with **GDM-NT-401**.

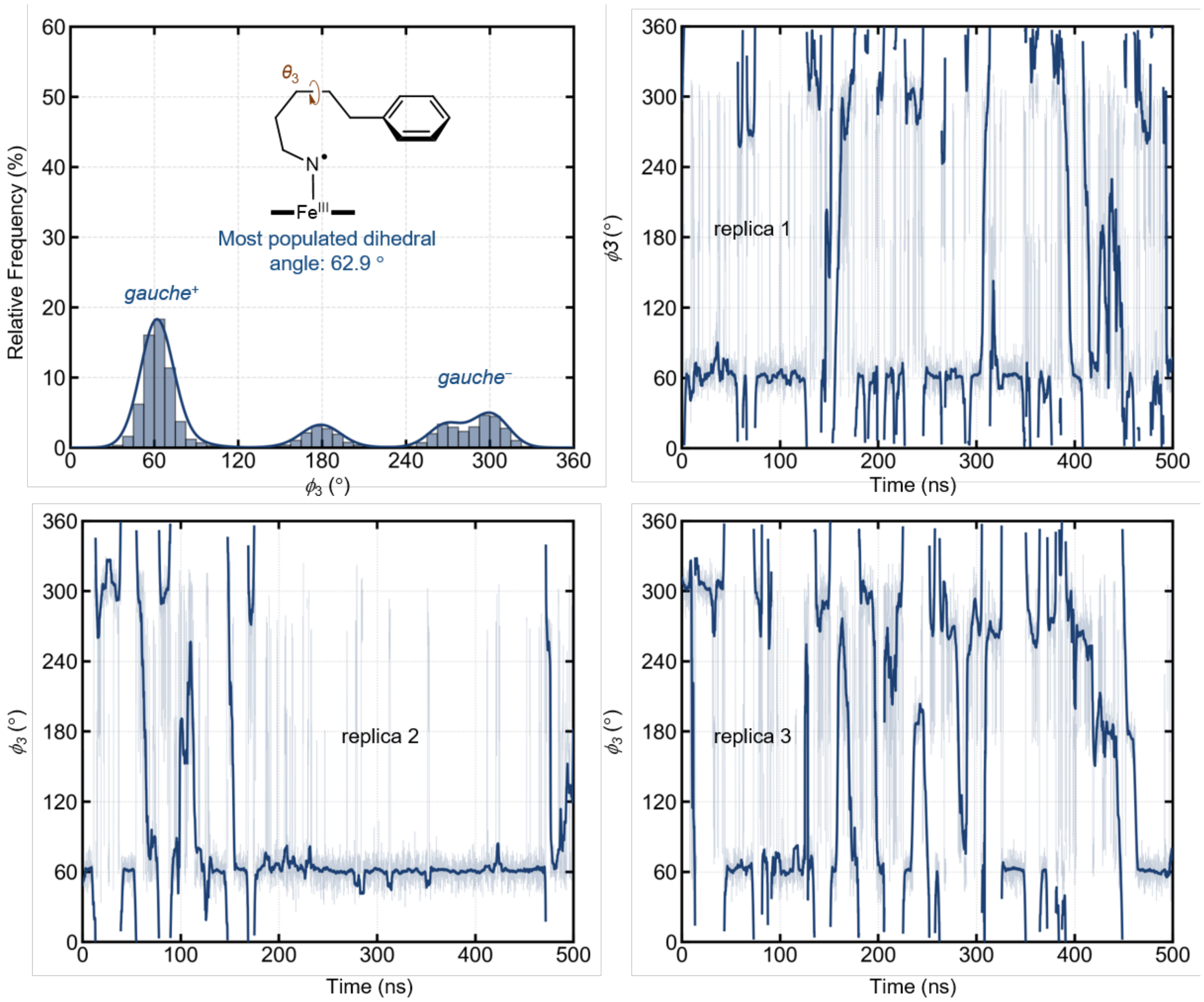

Figure S26. Dihedral angle θ_3 (CβCγCδCε) of the alkyl nitrene intermediate observed in unrestrained MD simulations with **GDM-NT-401**.

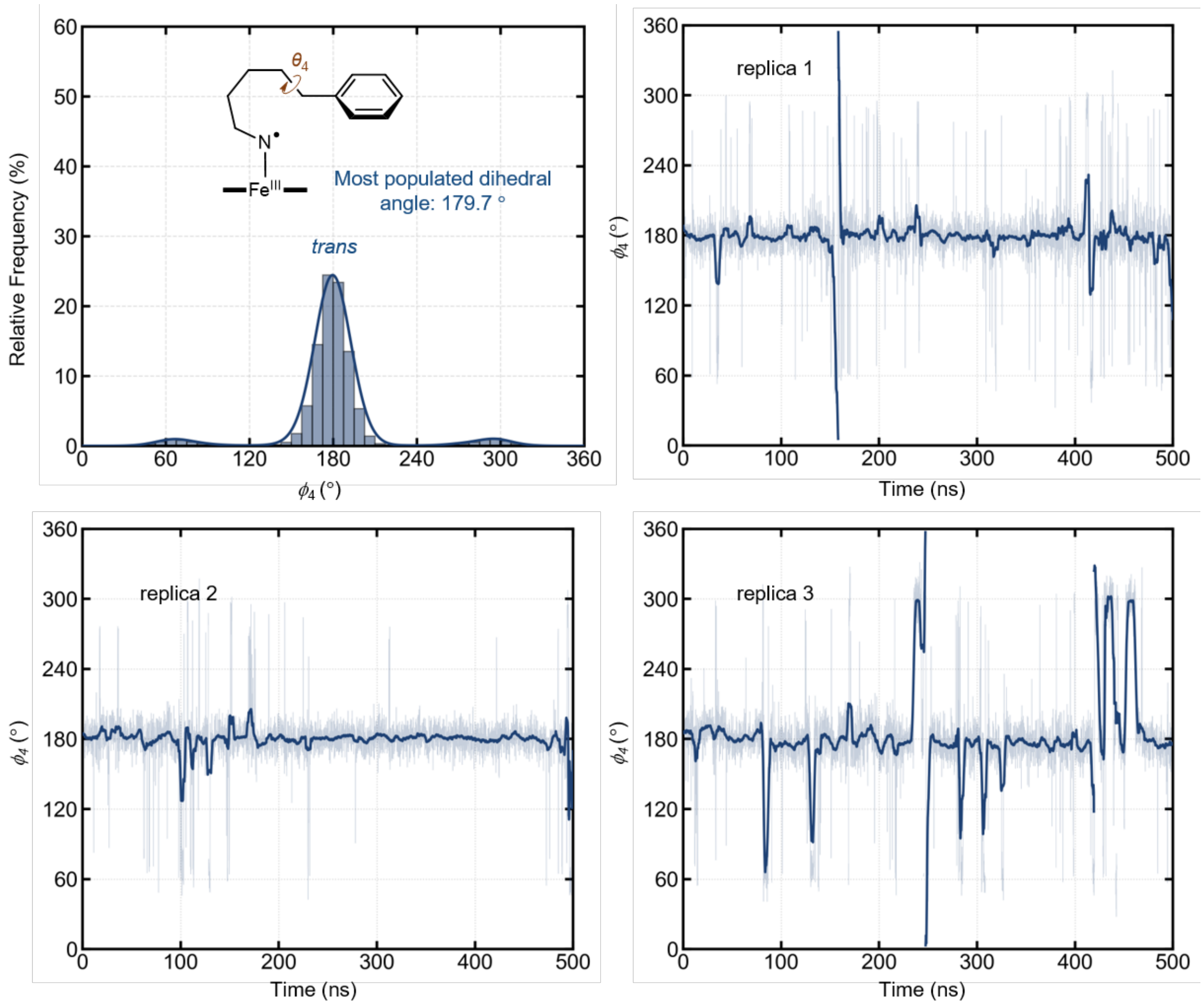

Figure S27. Dihedral angle θ_4 (CγCδCεCphenyl) of the alkyl nitrene intermediate observed in unrestrained MD simulations with **GDM-NT-401**.

Restrained classical MD simulations of the near attack conformations prior to Fe–nitrene HAT in ***Tm*****Pgb-PIP-G7 and GDM-NT-401**

To further investigate the enzyme–substrate interactions at reactive geometries near the HAT transition state, a distance restraint (2.9–3.5 Å, force constant 100 kcal mol^{-1} Å^{-2}) was applied between the nitrene nitrogen and the centroid of the δ- and ε-carbon atoms. In these restrained near-attack conformation (NAC) simulations, ***Tm*****Pgb-PIP-G7** populated 1,6-HAT (ε-C–H) geometries in 32.3% and 1,5-HAT (δ-C–H) geometries in 2.8% of the frames (Figure S28a, left), whereas **GDM-NT-401** reached 42.4% and 2.1%, respectively (Figure S28a, right), consistent with the higher regioselectivity of **GDM-NT-401**. The major near-attack conformations in **GDM-NT-401** and ***Tm*****Pgb-PIP-G7** are shown in Figure S28b and S28c, respectively. For **GDM-NT-401**, the substrate alkyl chain binding pose is essentially unchanged between the unrestrained and restrained simulations (Figure S28b), because the alkyl chain is already preorganised into the reactive bent geometry in the unrestrained MD. The C–H···π interaction between L20 side chain and the phenyl group of the substrate is maintained under the restraint, with essentially the same centroid–centroid distance as in the unrestrained simulation (4.0 ± 0.4 and 4.0 ± 0.3 Å, respectively; Figure S29). Consistent with this, the per-residue RMSF changes little upon introducing the restraint, and is in fact slightly lower in the restrained than in the unrestrained simulation, possibily induced by the reduced substrate flexibility (Figure S28d, left). By contrast, in ***Tm*****Pgb-PIP-G7** the nitrene alkyl chain adopts an

extended geometry in the unrestrained simulations, so reaching the HAT transition state-like bent geometry under the restraint requires substantial reorganisation (Figure S28c). The less complementary shape of the ***Tm*Pgb-PIP-G7** active site with the HAT transition state is reflected in the RMSF, which increases upon applying the restraint relative to the unrestrained simulations (Figure S28d, right). Moreover, the conformational change disrupts the favourable interactions of the Y60 and W59 aromatic side chains with the alkyl chain of the nitrene, and weakens the remaining F144–substrate $\pi\cdots\pi$ interaction, whose centroid–centroid distance increases from 4.6 ± 0.6 Å in the unrestrained simulations to 5.4 ± 0.7 Å under restraint (Figure S30).

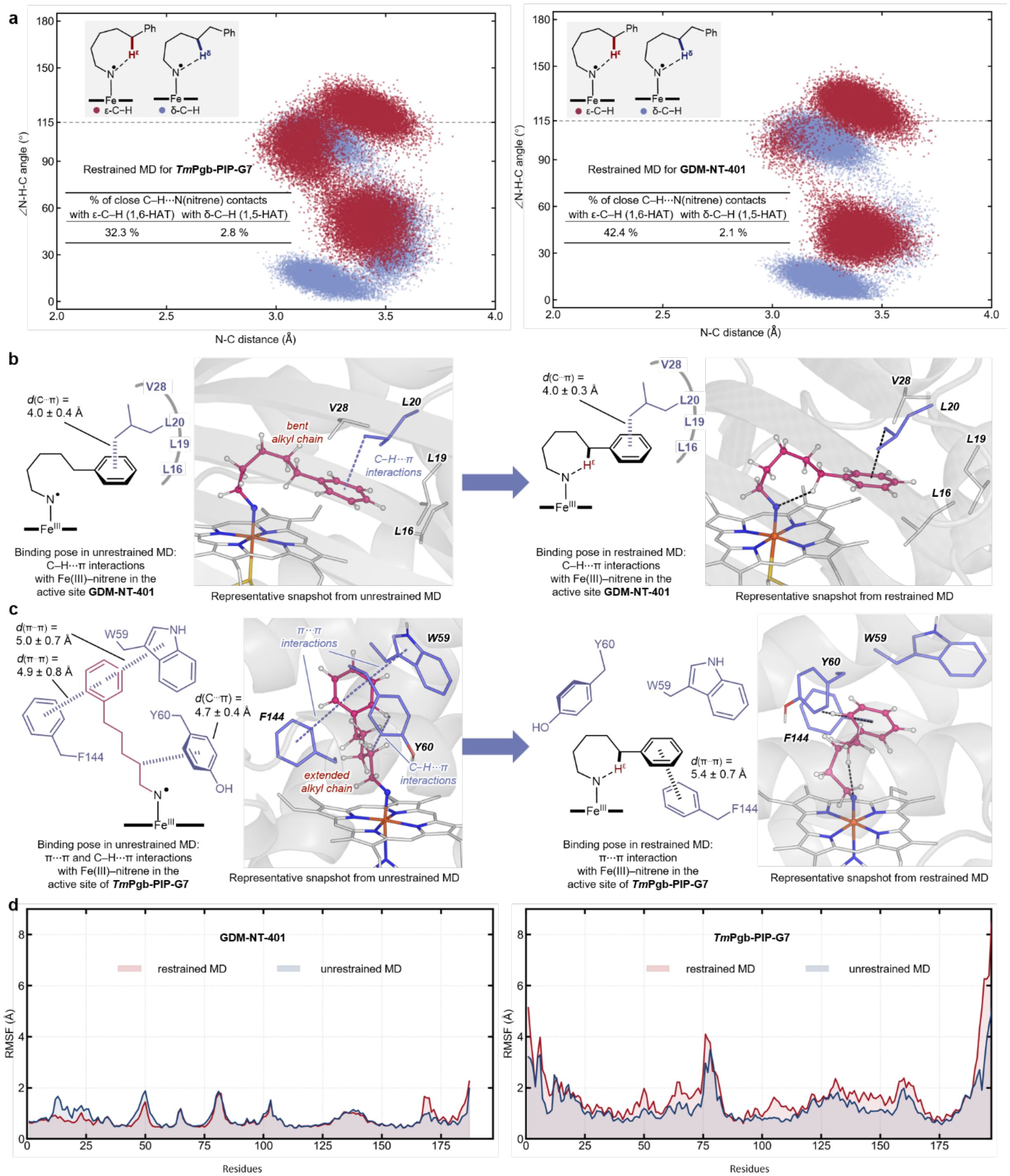

Figure S28. a, Close contacts between the nitrene N atom and the ε- and δ-C–H bonds observed in the restrained MD simulations of ***Tm*Pgb-PIP-G7** (left) and **GDM-NT-401** (right). **b,** Representative binding pose of the alkyl nitrene intermediate in the active site of **GDM-NT-401** (left, unrestrained MD; right, restrained MD). **c,** Representative binding pose of the alkyl nitrene intermediate in the active site of ***Tm*Pgb-PIP-G7** (left, unrestrained MD; right, restrained MD). **d,** RMSF profiles of the backbone C_α atoms for **GDM-NT-401** (restrained vs unrestrained MD) and ***Tm*Pgb-PIP-G7** (restrained vs unrestrained MD).

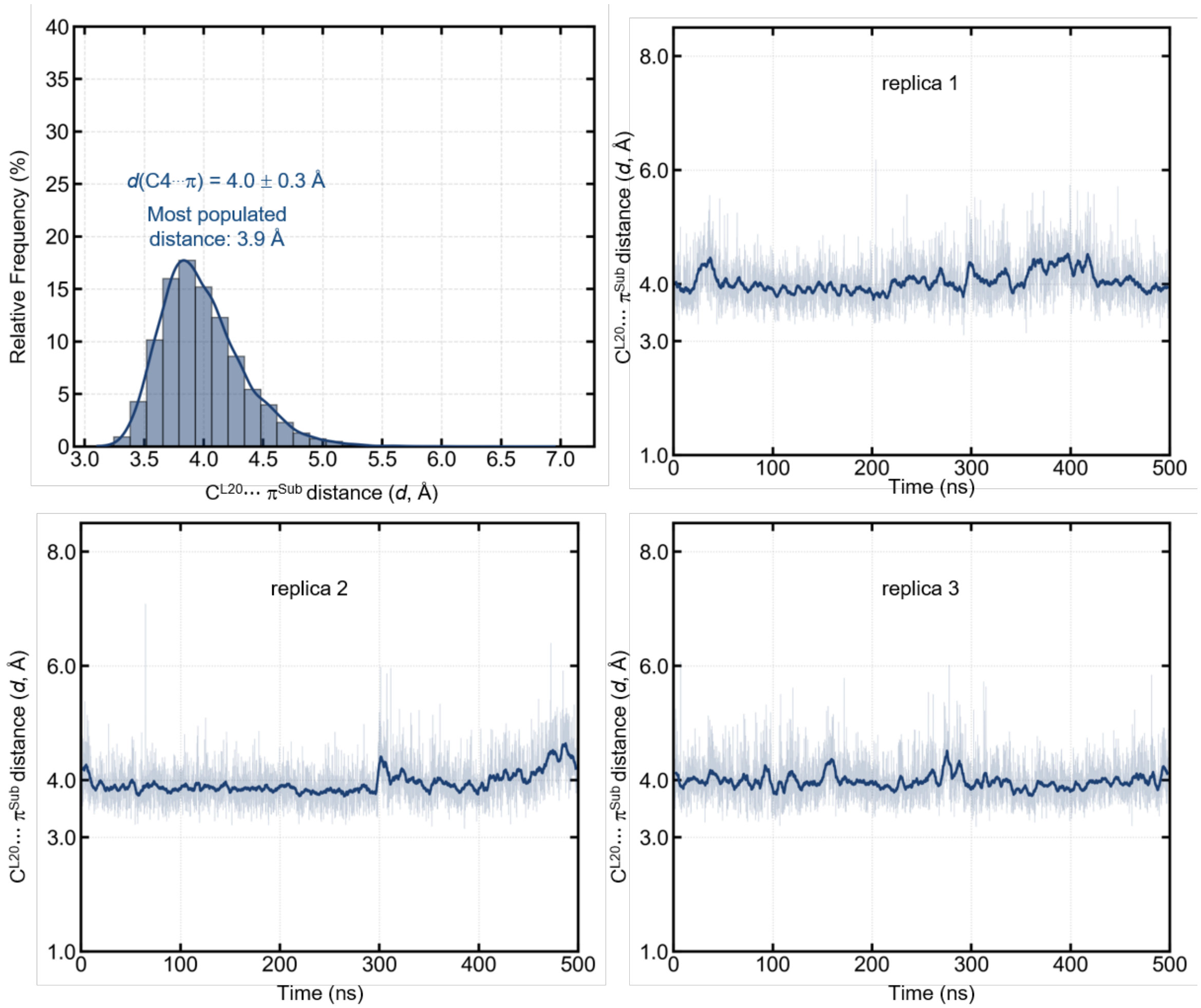

Figure S29. Distance between the centroid of the benzene ring of the substrate (π^{Sub}) and the C_δ atom of L20 in restrained MD simulations with **GDM-NT-401**.

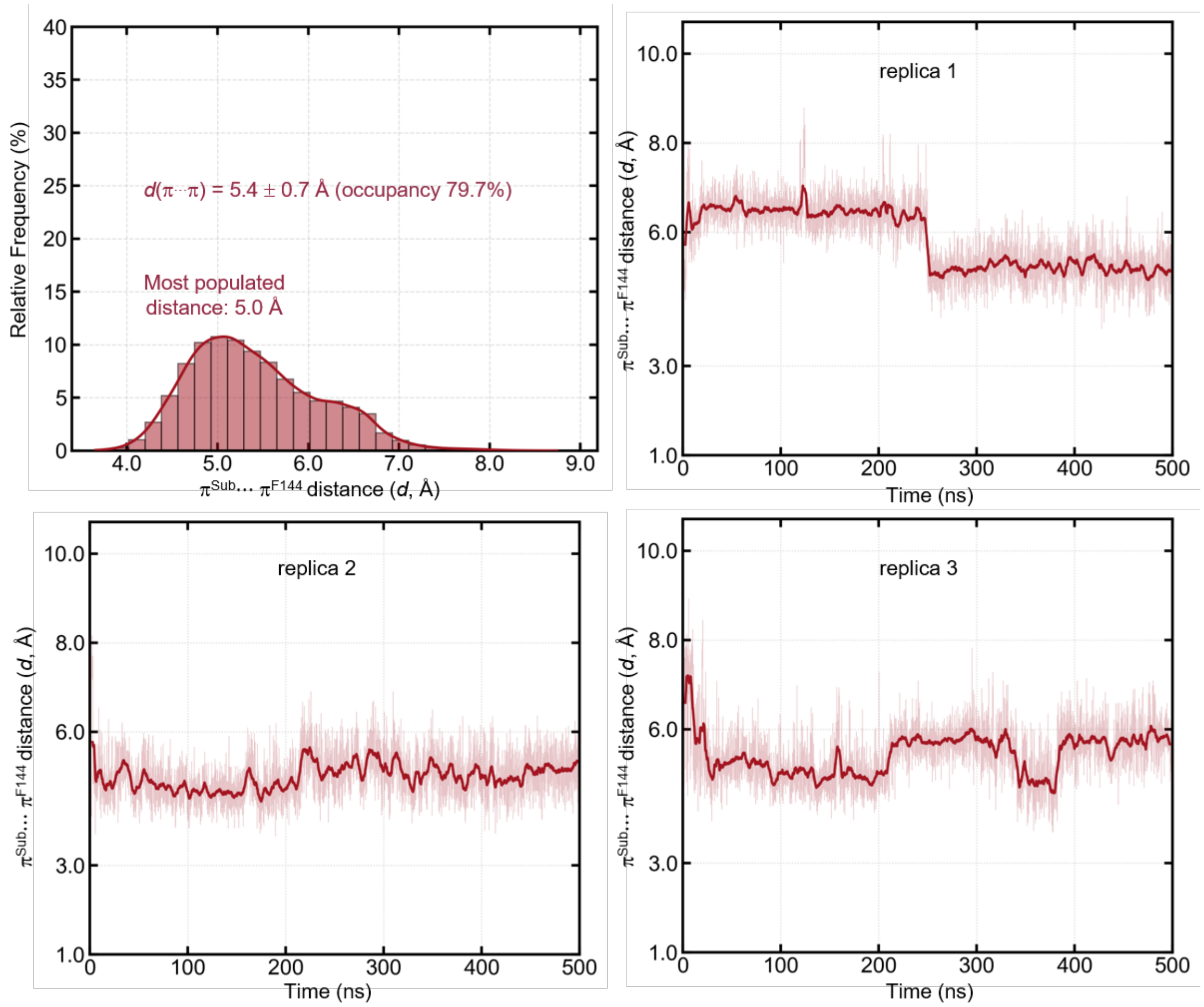

Figure S30. Distance between the centroid of the benzene ring of the substrate (π^{Sub}) and the centroid of the benzene ring of F144 side chain (π^{F144}) in restrained MD simulations with ***TmPgb*-PIP-G7**.

DFT-calculated energies of intermediates and transition states

Cartesian coordinates of all optimized geometries are provided in a separate file in the .xyz format.

Table S26. Calculated total (SCF) energies, enthalpies, and Gibbs free energies of intermediates and transition states.

	Level of theory: B3LYP-D3(BJ)/def2TZVP/SMD(Et_2O)//B3LYP-D3(BJ)/SDD-6-31G(d)		
	SCF energy (a.u.)	Enthalpy (a.u.)	Gibbs Free energy (a.u.)
**CP1-quintet**	-3111.101532	-3110.436652	-3110.558096
**CP1-septet**	-3111.060722	-3110.397338	-3110.519353
**CP1-singlet**	-3111.101361	-3110.434284	-3110.548611
**CP1-triplet**	-3111.100184	-3110.434326	-3110.558762
**INT1-quintet**	-3001.522783	-3000.871176	-3000.987808
**INT1-septet**	-3001.511573	-3000.861297	-3000.981276
**INT1-singlet**	-3001.525419	-3000.871093	-3000.980237
**INT1-triplet**	-3001.537833	-3000.884628	-3000.998551
**INT2a-quintet**	-3001.521609	-3000.871688	-3000.984300
**INT2a-septet**	-3001.534966	-3000.884968	-3000.997768

**INT2a-singlet**	-3001.513399	-3000.860142	-3000.966810
**INT2a-triplet**	-3001.548196	-3000.894246	-3001.003424
**INT2b-quintet**	-3001.492097	-3000.841849	-3000.955961
**INT2b-septet**	-3001.518630	-3000.869280	-3000.983126
**INT2b-triplet**	-3001.531464	-3000.878079	-3000.988548
**P2a-quintet**	-3001.613515	-3000.958087	-3001.068240
**P2a-septet**	-3001.578180	-3000.923476	-3001.031571
**P2a-singlet**	-3001.616195	-3000.958149	-3001.059685
**P2a-triplet**	-3001.613808	-3000.957223	-3001.066860
**P2b-quintet**	-3001.610559	-3000.955452	-3001.066702
**P2b-septet**	-3001.576587	-3000.922237	-3001.032105
**P2b-singlet**	-3001.618598	-3000.959315	-3001.064489
**P2b-triplet**	-3001.609119	-3000.953114	-3001.065095
**RC2a-quintet**	-3001.525372	-3000.873481	-3000.985267
**RC2a-septet**	-3001.514601	-3000.863999	-3000.977697
**RC2a-singlet**	-3001.525233	-3000.871133	-3000.981141
**RC2a-triplet**	-3001.542590	-3000.887835	-3000.999173
**RC2b-quintet**	-3001.527742	-3000.876675	-3000.988051
**RC2b-septet**	-3001.516608	-3000.865845	-3000.978407
**RC2b-singlet**	-3001.534163	-3000.879040	-3000.986792
**RC2b-triplet**	-3001.545701	-3000.890697	-3000.999582
**TS1-quintet**	-3111.058521	-3110.397652	-3110.518332
**TS1-septet**	-3111.047634	-3110.386905	-3110.508072
**TS1-singlet**	-3111.057956	-3110.394846	-3110.509861
**TS1-triplet**	-3111.065968	-3110.402709	-3110.517719
**TS2a-quintet**	-3001.511935	-3000.866788	-3000.975892
**TS2a-septet**	-3001.498886	-3000.854379	-3000.965513
**TS2a-singlet**	-3001.507251	-3000.859428	-3000.962146
**TS2a-triplet**	-3001.519847	-3000.872377	-3000.976291
**TS2b-quintet**	-3001.503886	-3000.859109	-3000.970649
**TS2b-septet**	-3001.493961	-3000.849419	-3000.960991
**TS2b-singlet**	-3001.503454	-3000.855394	-3000.958461
**TS2b-triplet**	-3001.510783	-3000.863689	-3000.972014
**TS3a-triplet**	-3001.532820	-3000.881057	-3000.986225
**TS3a-quintet**	-3001.520082	-3000.871102	-3000.982085
**TS3a-septet**	-3001.515090	-3000.866185	-3000.973514
**TS3b-triplet**	-3001.520445	-3000.868722	-3000.976704
**TS3b-quintet**	-3001.495653	-3000.847229	-3000.960153
**TS3b-septet**	-3001.496822	-3000.848232	-3000.960370
**CP1′-quintet**	-3362.837249	-3362.165557	-3362.285389
**CP1′-septet**	-3362.807778	-3362.137789	-3362.258465
**CP1′-singlet**	-3362.852395	-3362.178136	-3362.295865
**CP1′-triplet**	-3362.854157	-3362.181194	-3362.304147
**TS1′-quintet**	-3362.797100	-3362.129884	-3362.250020
**TS1′-septet**	-3362.802062	-3362.134304	-3362.256079
**TS1′-singlet**	-3362.804743	-3362.134438	-3362.247792
**TS1′-triplet**	-3362.818904	-3362.148438	-3362.265385
**INT1′-quintet**	-3253.267817	-3252.610907	-3252.726582
**INT1′-septet**	-3253.265969	-3252.608691	-3252.728605
**INT1′-singlet**	-3253.282891	-3252.621204	-3252.731586
**INT1′-triplet**	-3253.293474	-3252.632873	-3252.747467

**RC2b′-quintet**	-3253.260217	-3252.603921	-3252.714158
**RC2b′-septet**	-3253.273134	-3252.615236	-3252.727794
**RC2b′-singlet**	-3253.286650	-3252.624986	-3252.731748
**RC2b′-triplet**	-3253.298888	-3252.637812	-3252.745600
**RC2a′-quintet**	-3253.269783	-3252.612587	-3252.722691
**RC2a′-septet**	-3253.255721	-3252.598110	-3252.712488
**RC2a′-singlet**	-3253.284012	-3252.623196	-3252.732068
**RC2a′-triplet**	-3253.295532	-3252.634635	-3252.744115
**TS2b′-quintet**	-3253.262031	-3252.610014	-3252.721254
**TS2b′-septet**	-3253.250508	-3252.598777	-3252.710375
**TS2b′-singlet**	-3253.252537	-3252.597577	-3252.700178
**TS2b′-triplet**	-3253.260502	-3252.606693	-3252.711749
**TS2a′-quintet**	-3253.269074	-3252.616755	-3252.726220
**TS2a′-septet**	-3253.256325	-3252.604354	-3252.714181
**TS2a′-singlet**	-3253.252781	-3252.597702	-3252.699501
**TS2a′-triplet**	-3253.274323	-3252.619358	-3252.724310
**INT2b′-quintet**	-3253.270632	-3252.613172	-3252.725799
**INT2b′-septet**	-3253.275537	-3252.619071	-3252.733311
**INT2b′-triplet**	-3253.284049	-3252.624504	-3252.733405
**INT2a′-quintet**	-3253.286577	-3252.628577	-3252.740790
**INT2a′-septet**	-3253.291645	-3252.634557	-3252.747836
**INT2a′-triplet**	-3253.300711	-3252.640581	-3252.747936
**P2b′-quintet**	-3253.365346	-3252.703186	-3252.814124
**P2b′-septet**	-3253.326245	-3252.665082	-3252.774151
**P2b′-singlet**	-3253.367921	-3252.701681	-3252.806188
**P2b′-triplet**	-3253.366838	-3252.703355	-3252.814184
**P2a′-quintet**	-3253.361646	-3252.699228	-3252.808108
**P2a′-septet**	-3253.327806	-3252.666376	-3252.773689
**P2a′-singlet**	-3253.370231	-3252.703652	-3252.806852
**P2a′-triplet**	-3253.370197	-3252.706291	-3252.815040
**TS3a′-septet**	-3253.262661	-3252.606245	-3252.715700
**TS3a′-triplet**	-3253.278375	-3252.619374	-3252.726820
**TS3a′-quintet**	-3253.265614	-3252.608514	-3252.718009
**TS3b′-septet**	-3253.252530	-3252.596867	-3252.708882
**TS3b′-triplet**	-3253.274806	-3252.616748	-3252.727832
**TS3b′-quintet**	-3253.250854	-3252.595489	-3252.705629

Representative Analytical Data of Starting Materials

All product standards were purchased from commercial sources and used without further purification. Most alkyl azides are not stable under FD/FI/TOF conditions; therefore, we were not able to collect the HRMS data for the following compounds.

For review only: Most analytical data of starting materials are reported in our pyrrolidine synthase paper (*ref. 67 in manuscript*). At the discretion of the reviewers, we can provide a copy for the review process.

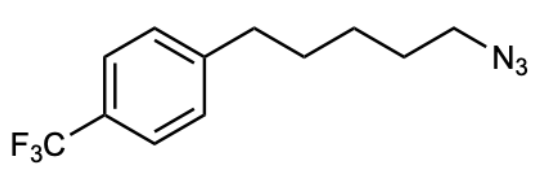

1-(5-azidopentyl)-4-(trifluoromethyl)benzene (1g): The reaction was carried out following General Procedure A. **^{1}H NMR** (400 MHz, $CDCl_3$) δ 7.81–7.52 (m, 2H), 7.34–7.27 (m, 2H), 3.27 (t, J = 6.8 Hz, 2H), 2.68 (t, J = 7.7 Hz, 2H), 1.94–1.60 (m, 4H), 1.53–1.35 (m, 2H). **^{13}C NMR** (101 MHz, $CDCl_3$) ^{13}C NMR (101 MHz, $CDCl_3$) δ 146.49, 128.80, 128.66–128.44 (m), 125.41 (q, $J_{C–F}$ = 3.7 Hz), 123.15, 51.47, 35.75, 30.84, 28.85, 26.45. **^{19}F NMR** (376 MHz, $CDCl_3$) δ –62.30.

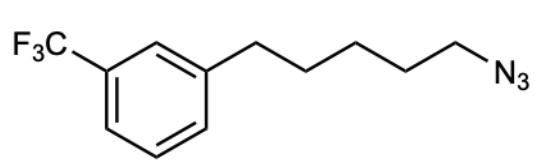

1-(5-azidopentyl)-3-(trifluoromethyl)benzene (1k): The reaction was carried out following General Procedure A. ^{1}H NMR (400 MHz, $CDCl_3$) δ 7.72–7.29 (m, 4H), 3.27 (td, J = 6.9, 4.0 Hz, 2H), 2.80–2.56 (m, 2H), 1.84–1.59 (m, 4H), 1.46–1.36 (m, 2H). **^{13}C NMR** (101 MHz, $CDCl_3$) δ 143.26, 131.91 (d, $J_{C–F}$ = 1.6 Hz), 130.78 (d, $J_{C–F}$ = 31.7 Hz), 128.99, 128.87, 125.16 (q, $J_{C–F}$ = 3.8 Hz), 122.85 (q, $J_{C–F}$ = 3.9 Hz), 51.46, 35.73, 30.94, 28.84, 26.46. **^{19}F NMR** (376 MHz, $CDCl_3$) δ –62.57.

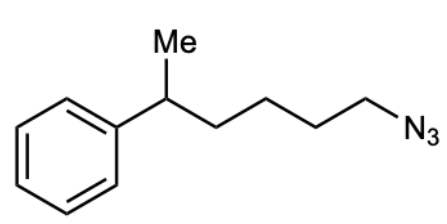

(6-azidohexan-2-yl)benzene (1m): The reaction was carried out following General Procedure B. ^{1}H NMR (400 MHz, $CDCl_3$) δ 7.46–7.26 (m, 2H), 7.23–7.11 (m, 3H), 3.21 (td, J = 7.0, 1.7 Hz, 2H), 2.68 (q, J = 7.1 Hz, 1H), 1.72–1.53 (m, 4H), 1.46–1.14 (m, 5H). **^{13}C NMR** (101 MHz, $CDCl_3$) δ 147.46, 128.51, 127.08, 126.10, 51.51, 40.01, 38.03, 29.05, 25.01, 22.45.

Supplementary References

1. Kille, S. et al. Reducing codon redundancy and screening effort of combinatorial protein libraries created by saturation mutagenesis. *ACS Synth. Biol.* **2**, 83–92 (2013).
2. Zhao, H., Giver, L., Shao, Z., Affholter, J. A. & Arnold, F. H. Molecular evolution by staggered extension process (StEP) in vitro recombination. *Nat. Biotechnol.* **16**, 258–261 (1998).
3. Gibson, D. G. et al. Enzymatic assembly of DNA molecules up to several hundred kilobases. *Nat. Methods* **6**, 343–345 (2009).
4. Barr, I. & Guo, F. Pyridine hemochromagen assay for determining the concentration of heme in purified protein solutions. *Bio Protoc.* **5**, e1594 (2015).
5. Ni, H.-Q., Li, Z.-Q., Tran, V. T. & Engle, K. M. Modular synthesis of non-conjugated *N*-(quinolin-8-yl) alkenyl amides via cross-metathesis. *Tetrahedron* **93**, 132279 (2021).
6. Google DeepMind & Frances Arnold Group. AlphaProtein Novo. Manuscript in preparation.
7. Abramson, J. *et al.* Accurate structure prediction of biomolecular interactions with AlphaFold 3. *Nature* **630**, 493–500 (2024).
8. Zhang, Y. & Skolnick, J. TM-align: a protein structure alignment algorithm based on the TM-score. *Nucleic Acids Res.* **33**, 2302–2309 (2005).
9. Mariani, V., Biasini, M., Barbato, A. & Schwede, T. lDDT: a local superposition-free score for comparing protein structures and models using distance difference tests. *Bioinformatics* **29**, 2722–2728 (2013).
10. Dauparas, J. *et al.* Atomic context-conditioned protein sequence design using LigandMPNN. *Nat. Methods* **22**, 717–723 (2025).
11. Berman, H. M. *et al.* The Protein Data Bank. *Nucleic Acids Res.* **28**, 235–242 (2000).
12. Steinegger, M. & Söding, J. MMseqs2 enables sensitive protein sequence searching for the analysis of massive data sets. *Nat. Biotechnol.* **35**, 1026–1028 (2017).
13. Pedregosa, F. et al. Scikit-learn: machine learning in Python. *J. Mach. Learn. Res.* **12**, 2825–2830 (2011).
14. McInnes, L., Healy, J. & Melville, J. UMAP: uniform manifold approximation and projection for dimension reduction. Preprint at https://arxiv.org/abs/1802.03426 (2018).
15. Frisch, M. J. et al. Gaussian 16 (Gaussian, Inc., Wallingford, CT, 2016).
16. Grimme, S., Antony, J., Ehrlich, S. & Krieg, H. A consistent and accurate ab initio parametrization of density functional dispersion correction (DFT-D) for the 94 elements H–Pu. *J. Chem. Phys.* **132**, 154104 (2010).
17. Grimme, S., Ehrlich, S. & Goerigk, L. Effect of the damping function in dispersion corrected density functional theory. *J. Comput. Chem.* **32**, 1456–1465 (2011).
18. Lee, C., Yang, W. & Parr, R. G. Development of the Colle-Salvetti correlation-energy formula into a functional of the electron density. *Phys. Rev. B* **37**, 785–789 (1988).
19. Becke, A. D. Density-functional thermochemistry. III. The role of exact exchange. *J. Chem. Phys.* **98**, 5648–5652 (1993).
20. Dolg, M., Wedig, U., Stoll, H. & Preuss, H. Energy-adjusted ab initio pseudopotentials for the first row transition elements. *J. Chem. Phys.* **86**, 866–872 (1987).
21. Weigend, F. & Ahlrichs, R. Balanced basis sets of split valence, triple zeta valence and quadruple zeta valence quality for H to Rn: design and assessment of accuracy. *Phys. Chem. Chem. Phys.* **7**, 3297–3305 (2005).
22. Weigend, F. Accurate Coulomb-fitting basis sets for H to Rn. *Phys. Chem. Chem. Phys.* **8**, 1057–1065 (2006).
23. Marenich, A. V., Cramer, C. J. & Truhlar, D. G. Universal solvation model based on solute electron density and on a continuum model of the solvent defined by the bulk dielectric constant and atomic surface tensions. *J. Phys. Chem. B* **113**, 6378–6396 (2009).

24. Warshel, A. & Russell, S. T. Calculations of electrostatic interactions in biological systems and in solutions. *Q. Rev. Biophys.* **17**, 283–422 (1984).
25. Zhao, Y. & Truhlar, D. G. The M06 suite of density functionals for main group thermochemistry, thermochemical kinetics, noncovalent interactions, excited states, and transition elements: two new functionals and systematic testing of four M06-class functionals and 12 other functionals. *Theor. Chem. Acc.* **120**, 215–241 (2008).
26. St. John, P. C., Guan, Y., Kim, Y., Kim, S. & Paton, R. S. Prediction of organic homolytic bond dissociation enthalpies at near chemical accuracy with sub-second computational cost. *Nat. Commun.* **11**, 2328 (2020).
27. Legault, C. Y. CYLview20 (Université de Sherbrooke, 2020); http://www.cylview.org.
28. Mahajan, M. & Mondal, B. How axial coordination regulates the electronic structure and C–H amination reactivity of Fe–porphyrin–nitrene? *JACS Au* **3**, 3494–3505 (2023).
29. Boitreaud, J. et al. Chai-1: decoding the molecular interactions of life. Preprint at https://doi.org/10.1101/2024.10.10.615955 (2024).
30. Anandakrishnan, R., Aguilar, B. & Onufriev, A. V. H++ 3.0: automating pK prediction and the preparation of biomolecular structures for atomistic molecular modeling and simulations. *Nucleic Acids Res.* **40**, W537–W541 (2012).
31. Salomon-Ferrer, R., Götz, A. W., Poole, D., Le Grand, S. & Walker, R. C. Routine microsecond molecular dynamics simulations with AMBER on GPUs. 2. Explicit solvent particle mesh Ewald. *J. Chem. Theory Comput.* **9**, 3878–3888 (2013).
32. Case, D. A. et al. Amber 2024 (University of California, San Francisco, 2024).
33. Tian, C. et al. ff19SB: amino-acid-specific protein backbone parameters trained against quantum mechanics energy surfaces in solution. *J. Chem. Theory Comput.* **16**, 528–552 (2020).
34. Li, P. & Merz, K. M. MCPB.py: a python based metal center parameter builder. *J. Chem. Inf. Model.* **56**, 599–604 (2016).
35. Wang, J., Wolf, R. M., Caldwell, J. W., Kollman, P. A. & Case, D. A. Development and testing of a general amber force field. *J. Comput. Chem.* **25**, 1157–1174 (2004).
36. Singh, U. C. & Kollman, P. A. An approach to computing electrostatic charges for molecules. *J. Comput. Chem.* **5**, 129–145 (1984).
37. Besler, B. H., Merz, K. M. Jr & Kollman, P. A. Atomic charges derived from semiempirical methods. *J. Comput. Chem.* **11**, 431–439 (1990).
38. Bayly, C. I., Cieplak, P., Cornell, W. & Kollman, P. A. A well-behaved electrostatic potential based method using charge restraints for deriving atomic charges: the RESP model. *J. Phys. Chem.* **97**, 10269–10280 (1993).
39. Jorgensen, W. L., Chandrasekhar, J., Madura, J. D., Impey, R. W. & Klein, M. L. Comparison of simple potential functions for simulating liquid water. *J. Chem. Phys.* **79**, 926–935 (1983).
40. Ryckaert, J.-P., Ciccotti, G. & Berendsen, H. J. C. Numerical integration of the cartesian equations of motion of a system with constraints: molecular dynamics of n-alkanes. *J. Comput. Phys.* **23**, 327–341 (1977).
41. Darden, T., York, D. & Pedersen, L. Particle mesh Ewald: an N·log(N) method for Ewald sums in large systems. *J. Chem. Phys.* **98**, 10089–10092 (1993).
42. Roe, D. R. & Cheatham, T. E. III. PTRAJ and CPPTRAJ: software for processing and analysis of molecular dynamics trajectory data. *J. Chem. Theory Comput.* **9**, 3084–3095 (2013).
43. Shao, J., Tanner, S. W., Thompson, N. & Cheatham, T. E. III. Clustering molecular dynamics trajectories: 1. Characterizing the performance of different clustering algorithms. *J. Chem. Theory Comput.* **3**, 2312–2334 (2007).
44. Lightstone, F. C. & Bruice, T. C. Ground state conformations and entropic and enthalpic factors in the efficiency of intramolecular and enzymatic reactions. 1. Cyclic anhydride formation by substituted glutarates, succinate, and 3,6-endoxo-Δ4-tetrahydrophthalate monophenyl esters. *J. Am. Chem. Soc.* **118**, 2595–2605 (1996).

Representative NMR spectrum

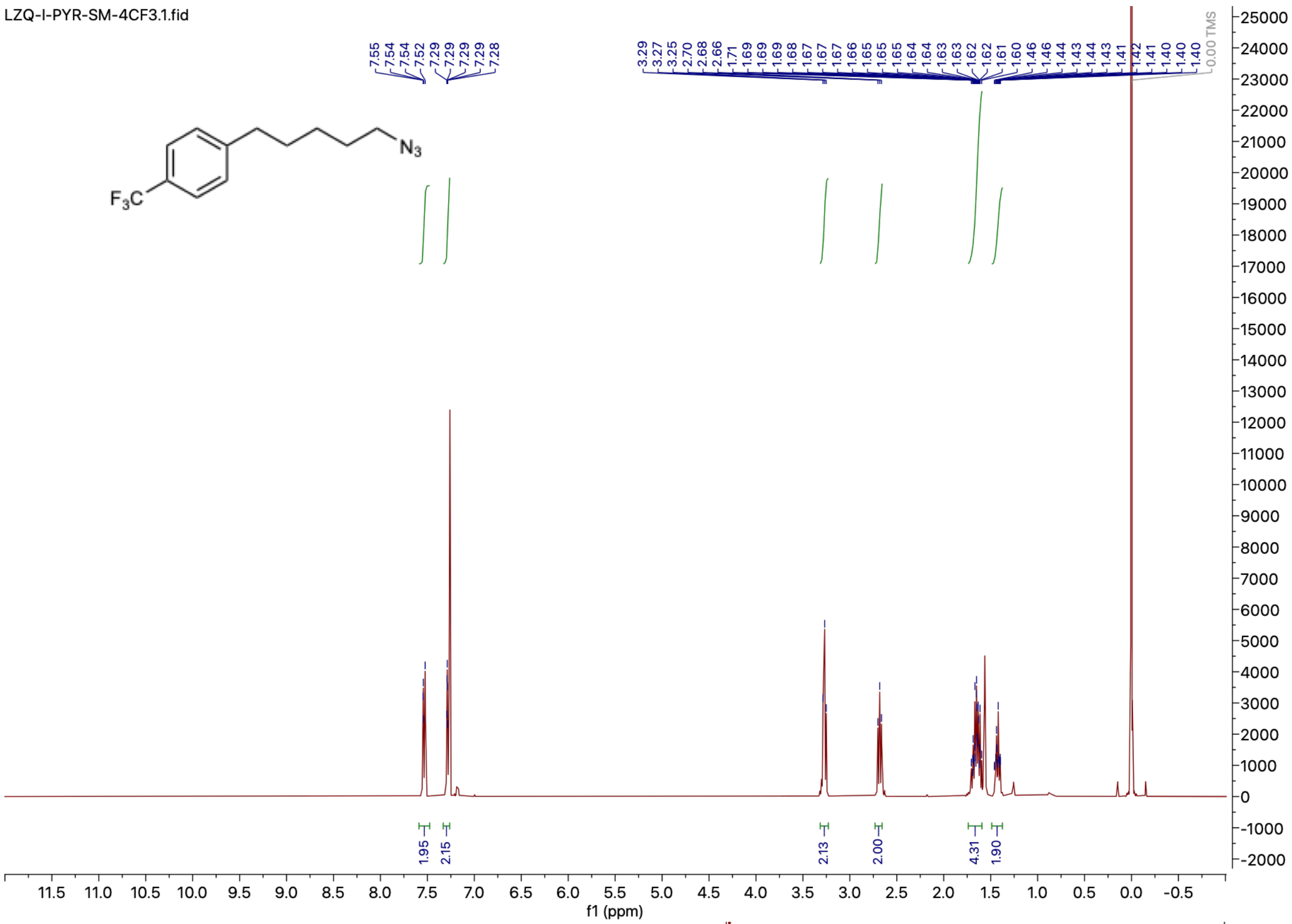

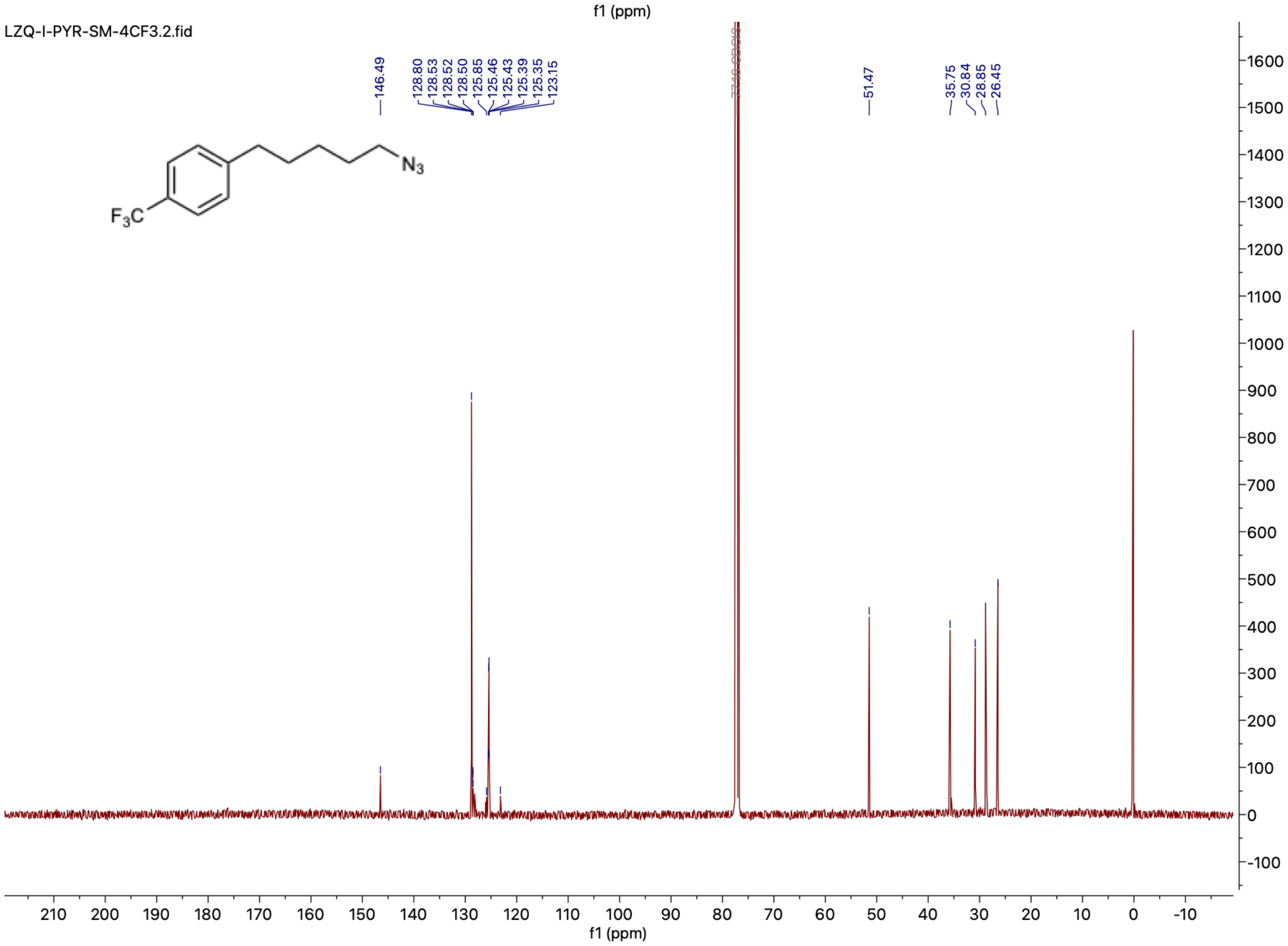

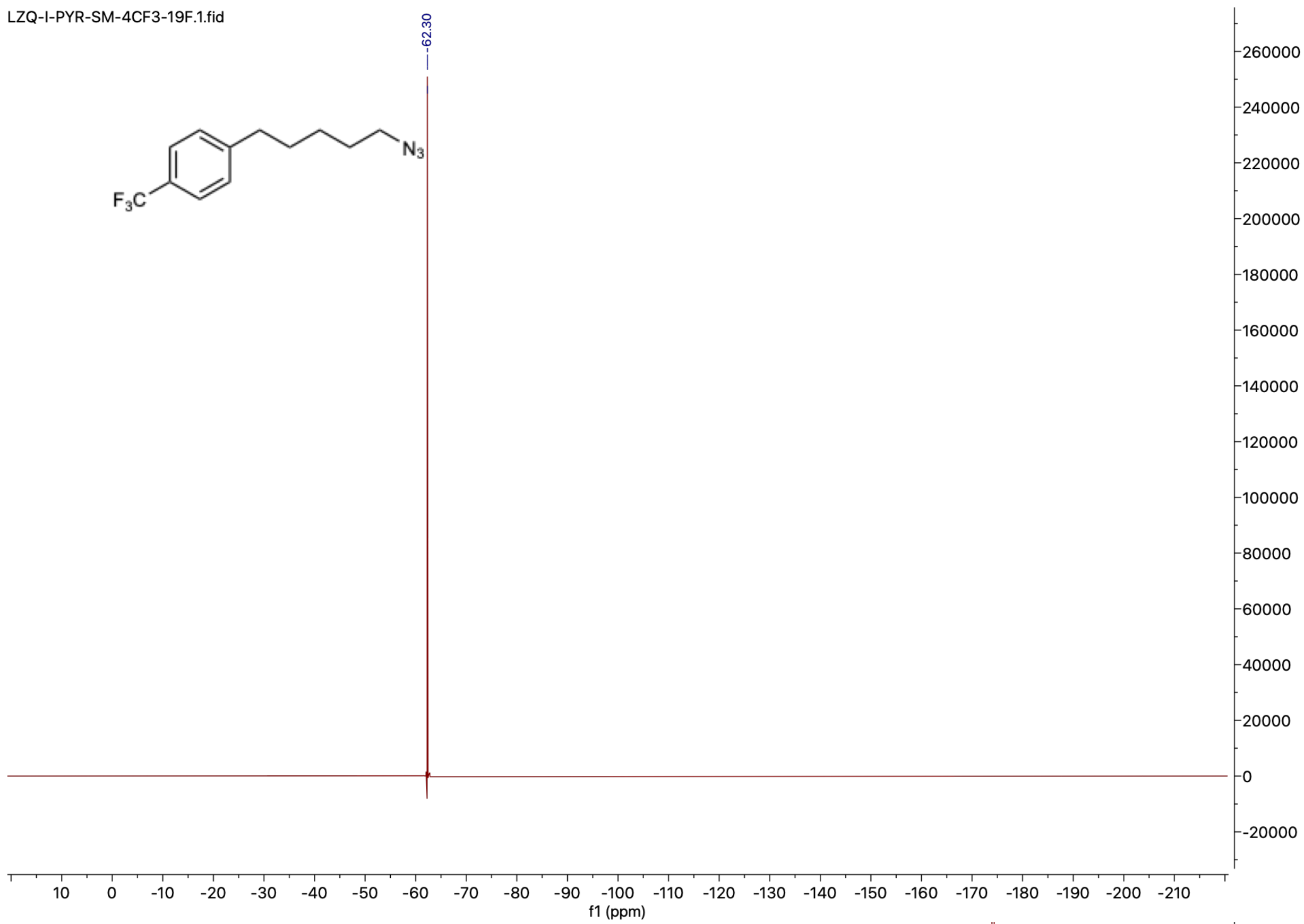
LZQ-I-PYR-SM-4CF3-19F.1.fid
-62.30
f1 (ppm)

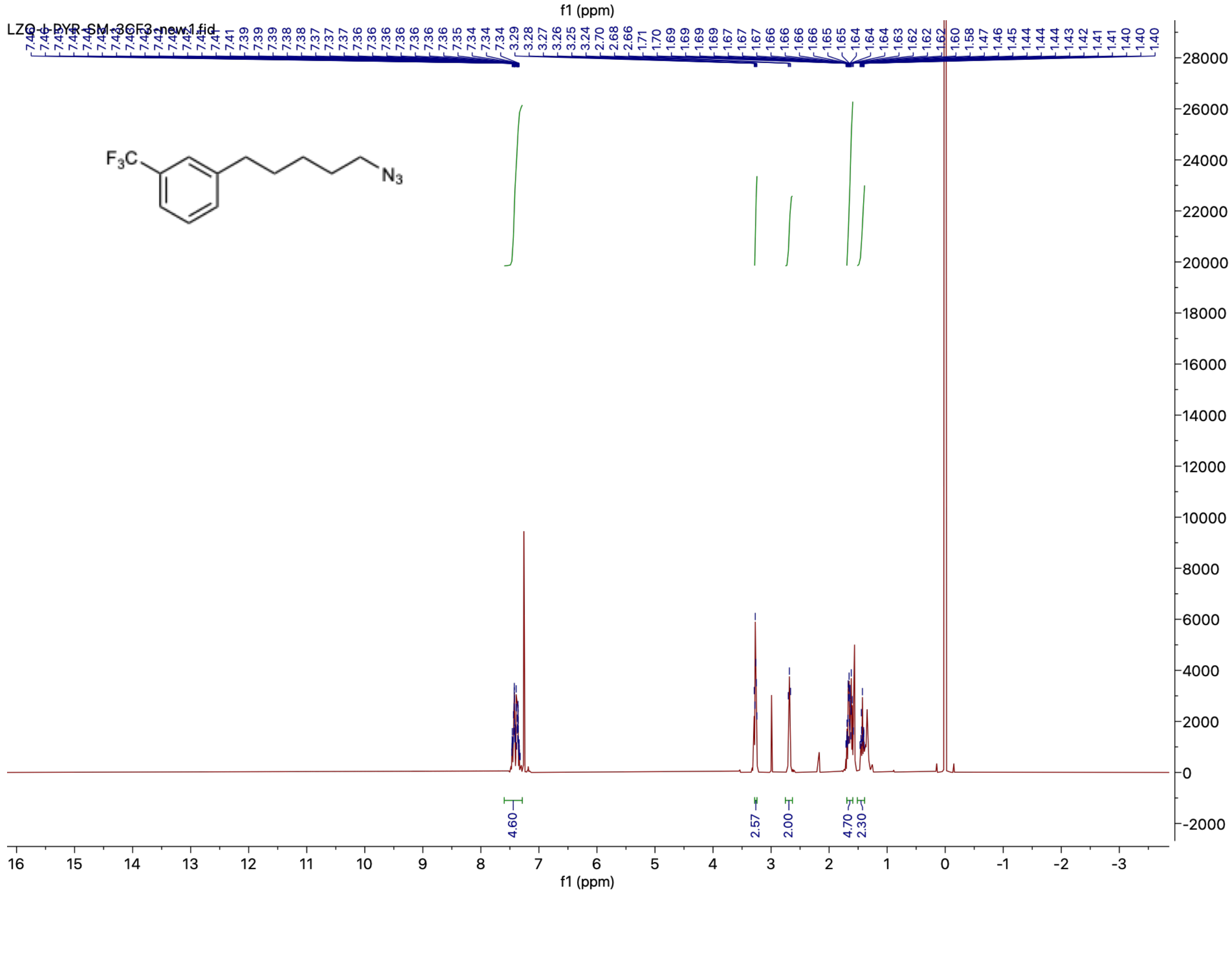
LZQ-I-PYR-SM-3CF3-new.1.fid
4.60
2.57
2.00
4.70
2.30
f1 (ppm)

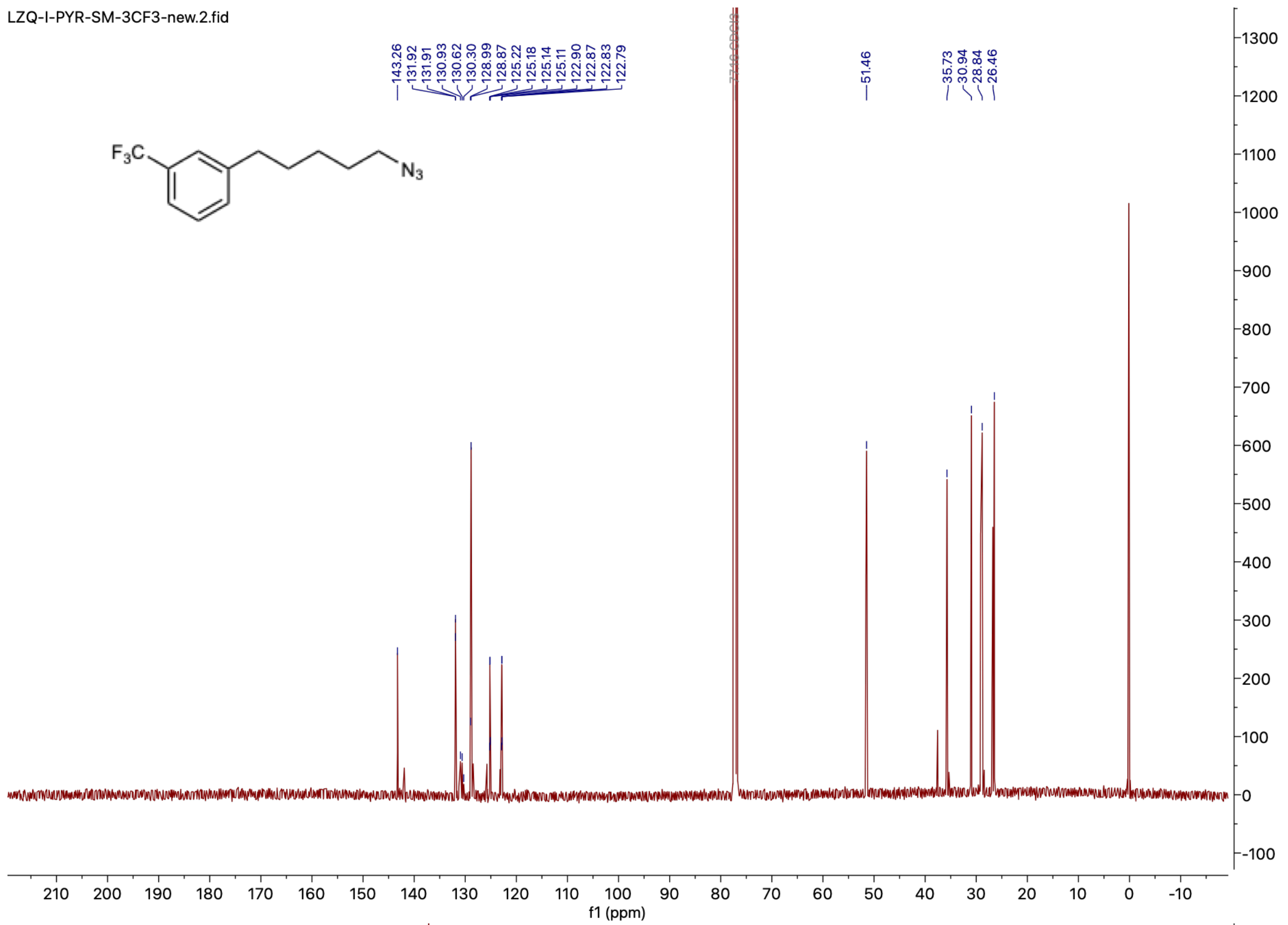

LZQ-I-PYR-SM-3CF3-new.2.fid
143.26
131.92
131.91
130.93
130.62
130.30
128.99
128.87
125.22
125.18
125.14
125.11
122.90
122.87
122.83
122.79
51.46
35.73
30.94
28.84
26.46
F_3C
N_3
f1 (ppm)

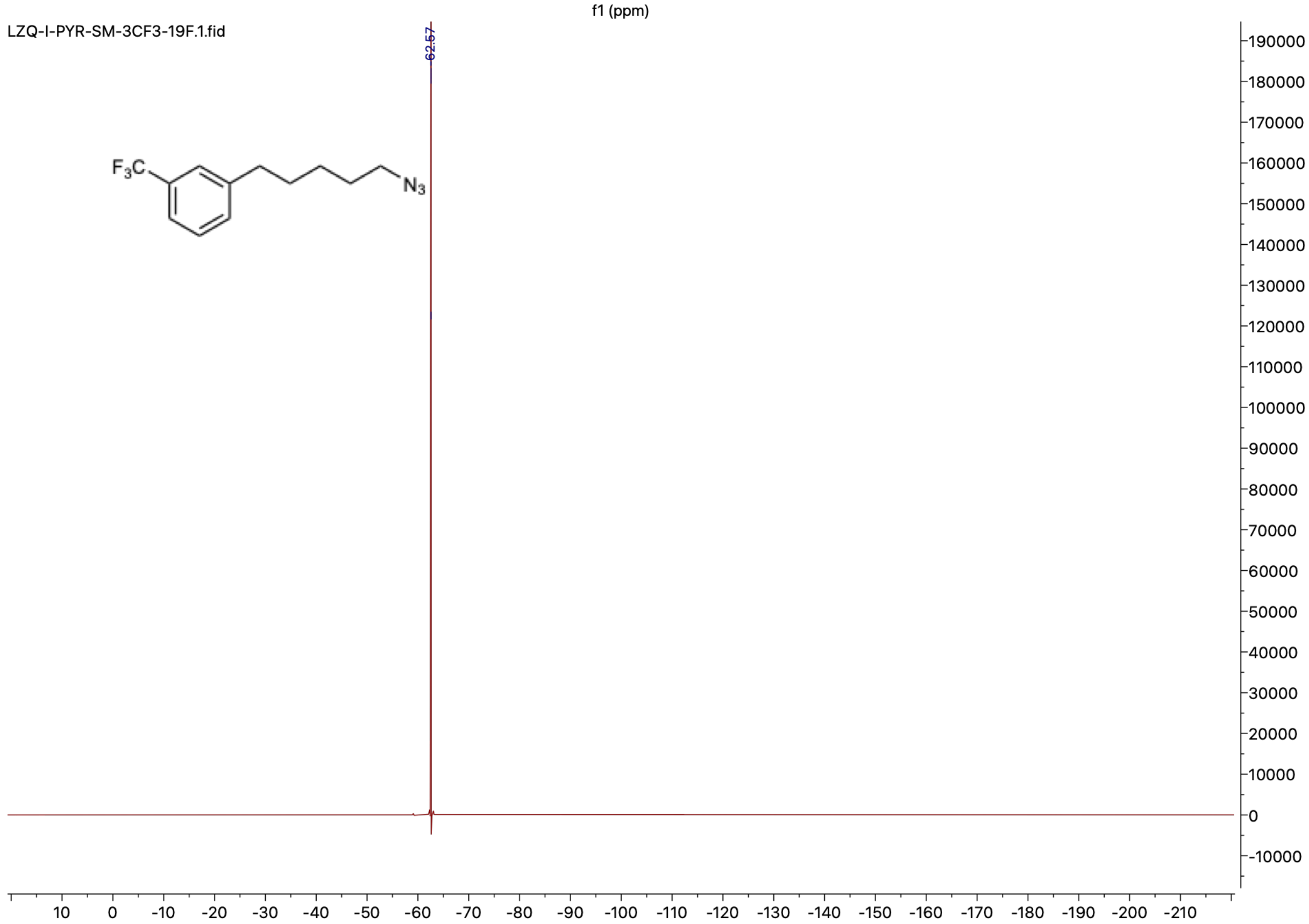

LZQ-I-PYR-SM-3CF3-19F.1.fid
-62.57
F_3C
N_3
f1 (ppm)

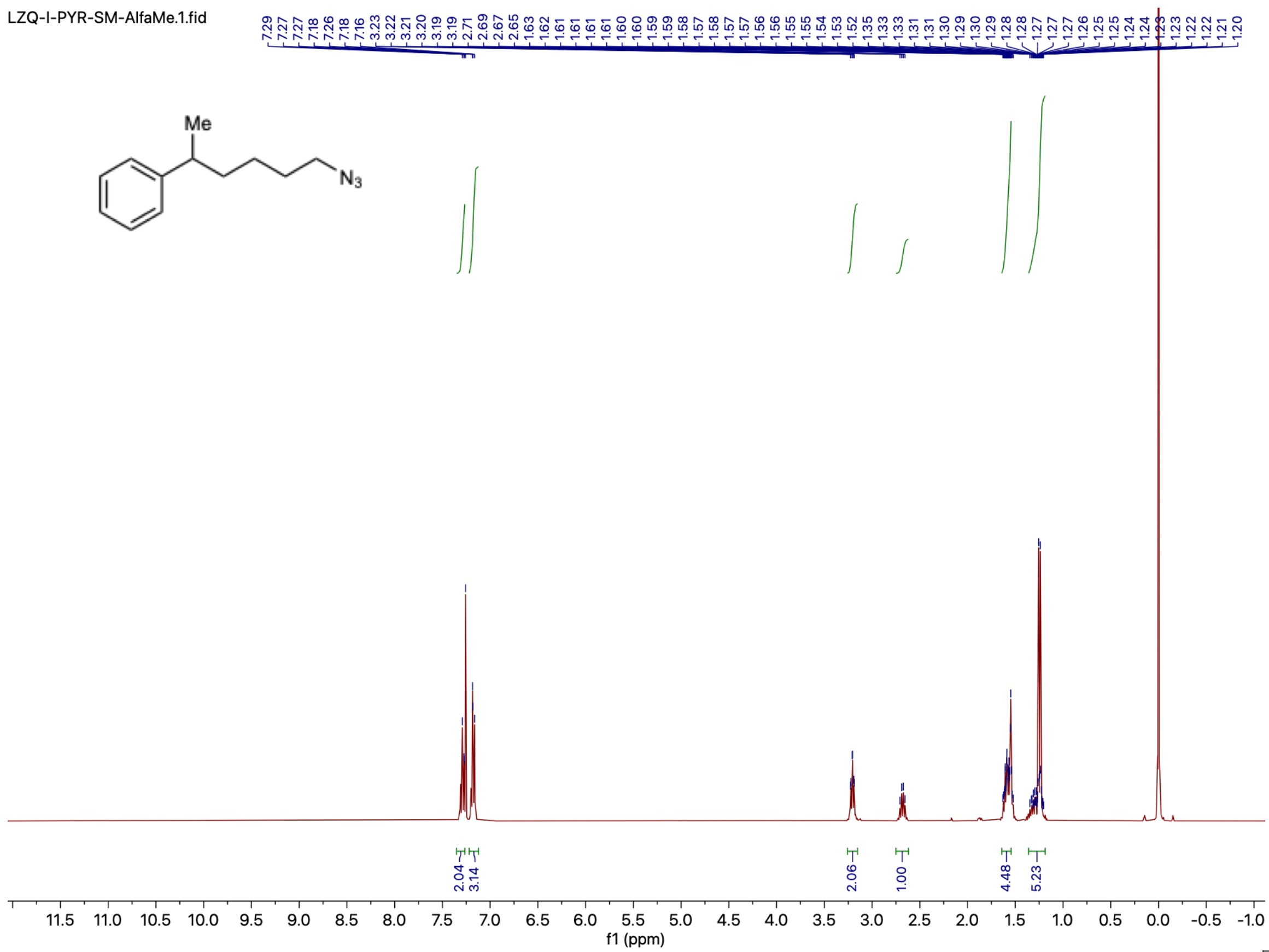
LZQ-I-PYR-SM-AlfaMe.1.fid
Me
N3
7.29 7.27 7.27 7.18 7.18 7.16 3.23 3.22 3.21 3.20 3.19 3.19 2.71 2.69 2.67 2.65 1.63 1.62 1.61 1.61 1.61 1.61 1.60 1.60 1.59 1.59 1.58 1.58 1.57 1.57 1.57 1.56 1.56 1.55 1.55 1.54 1.53 1.52 1.35 1.33 1.33 1.33 1.31 1.31 1.30 1.29 1.30 1.29 1.28 1.28 1.27 1.27 1.27 1.26 1.25 1.25 1.24 1.24 1.23 1.23 1.22 1.22 1.21 1.20
2.04
3.14
2.06
1.00
4.48
5.23
f1 (ppm)
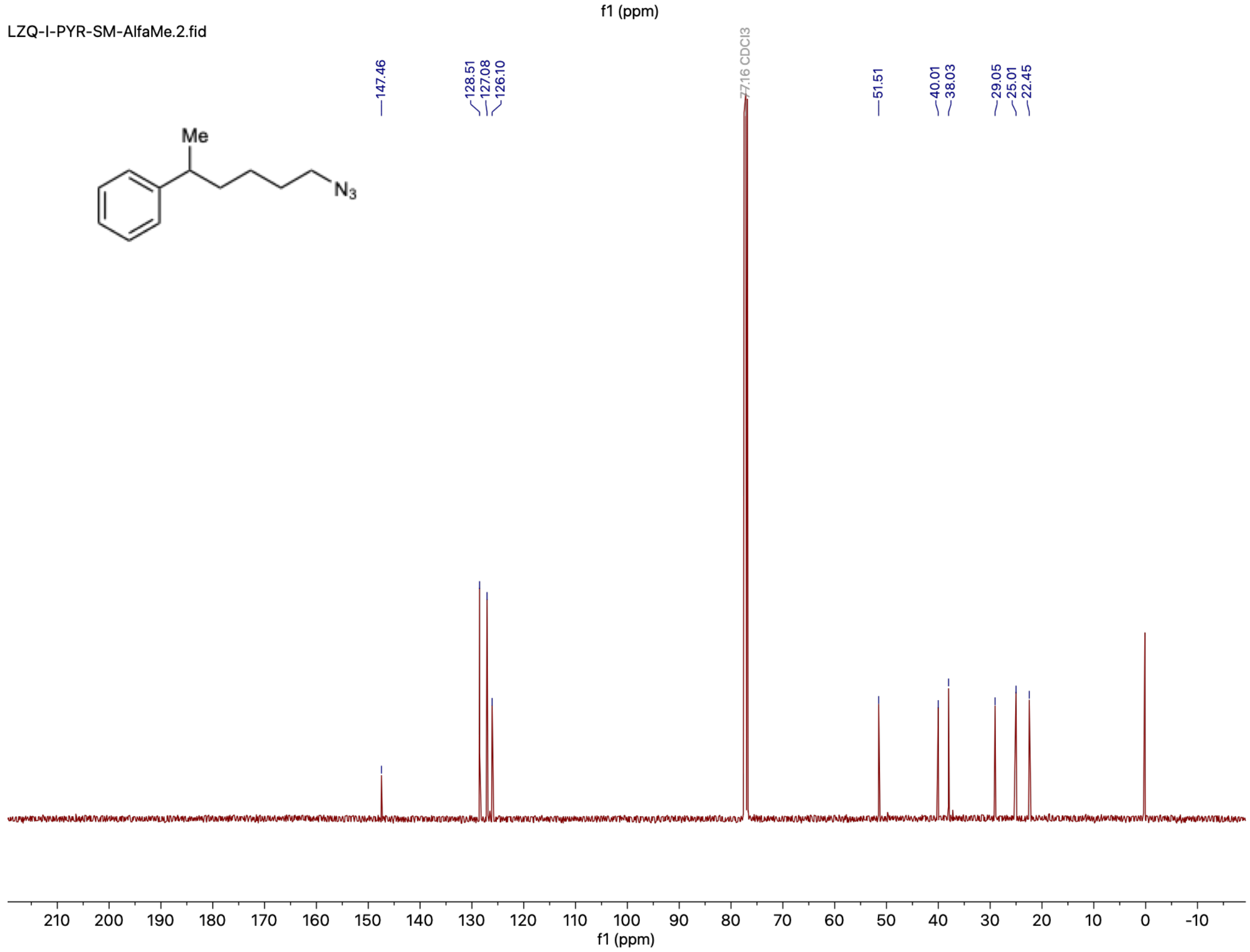
LZQ-I-PYR-SM-AlfaMe.2.fid
Me
N3
147.46
128.51
127.08
126.10
77.16 CDCl3
51.51
40.01
38.03
29.05
25.01
22.45
f1 (ppm)

Representative Chiral HPLC-MS data

Part 1: Regio- and enantioselectivity analysis of **2a** and **3a** on reactions performed with ***Tm*Pgb-PIP** lineage.
Results analyzed on polar ionic chiral HPLC-MS.

Chiral separation method: polar ionic HPLC-MS using *single* Agilent InfinityLab Poroshell 120 Chiral-V, 2.7 µm, 2.1 × 150 mm; eluent: 40% MeOH (0.2 w% NH_4CO_2H), 60% H_2O (20 mM NH_4CO_2H, pH 4.0 adjusted with HCO_2H); flow rate: 0.2 mL/min.
$t_{R\text{-}2a}$ = 15.439 min, $t_{S\text{-}2a}$ = 16.270 min. $t_{R\text{-}3a}$ = 14.466 min, $t_{S\text{-}3a}$ = 17.430 min.

(*rac*)-**2a** (commercially available standard)

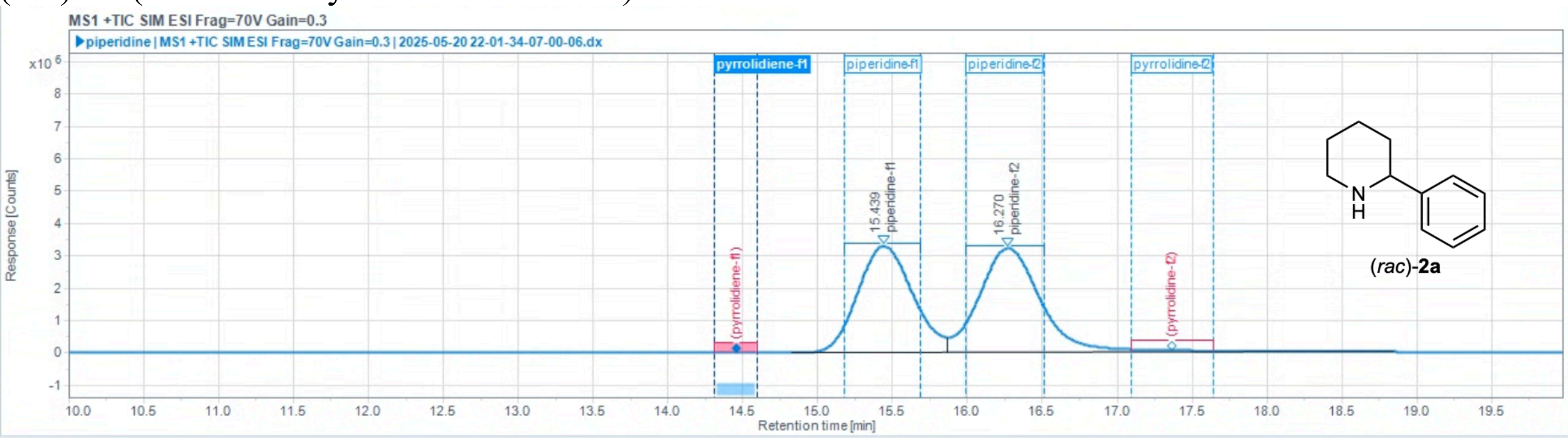

Injection Results

Peaks | Summary

#	Name	Signal description	RT (min)	Area (Counts·s)	Area%	Height (Counts)	Height%	Amount	Concentration	Start time (min)	End time (min)
1	piperidine-f1	MS1 +TIC SIM ESI Frag=70V Gain=0.3	15.439	83873295.146	47.132	3287426.993	50.42			14.825	15.868
2	piperidine-f2	MS1 +TIC SIM ESI Frag=70V Gain=0.3	16.270	94081538.107	52.868	3232983.971	49.58			15.868	18.851

(*rac*)-**3a** (commercially available standard)

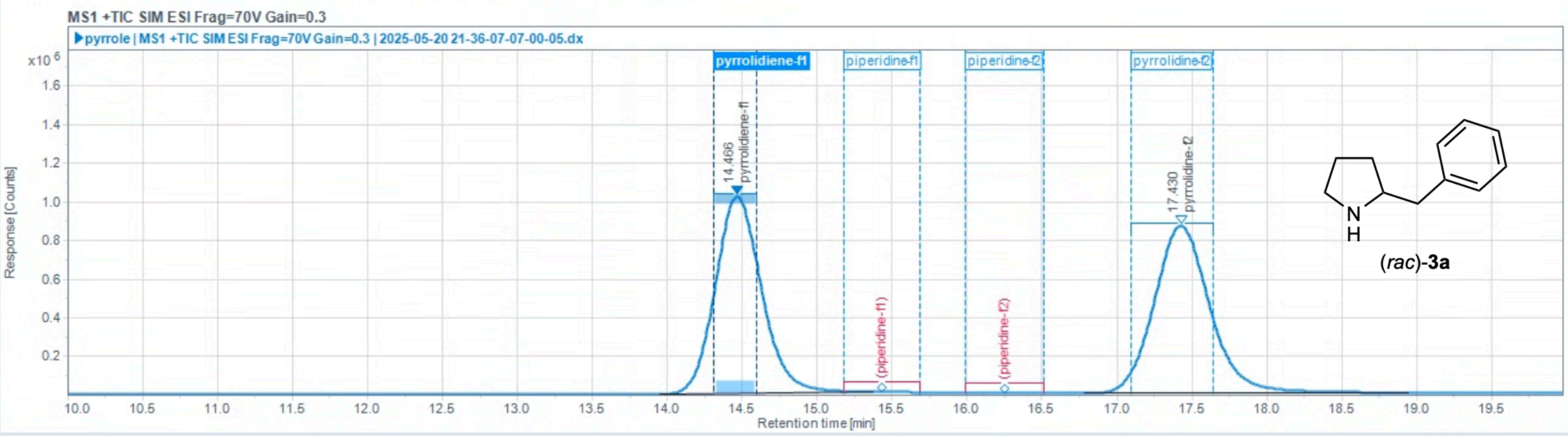

Injection Results

Peaks | Summary

#	Name	Signal description	RT (min)	Area (Counts·s)	Area%	Height (Counts)	Height%	Amount	Concentration	Start time (min)	End time (min)
1	pyrrolidiene-f1	MS1 +TIC SIM ESI Frag=70V Gain=0.3	14.466	21786543.915	49.217	1020171.145	54.01			13.947	15.371
2	pyrrolidine-f2	MS1 +TIC SIM ESI Frag=70V Gain=0.3	17.430	22480066.557	50.783	868519.200	45.99			16.780	18.942

Table S27. Regioselectivity and enantioselectivity of TmPgb-PIP lineage.

	rr (**2a**:**3a**)	**2a** ee (%)
***Tm*Pgb-wt**	3:97	–96
***Tm*Pgb-PIP-G1**	45:55	>99
***Tm*Pgb-PIP-G2**	56:44	>99
***Tm*Pgb-PIP-G3**	71:29	>99
***Tm*Pgb-PIP-G4**	89:11	>99
***Tm*Pgb-PIP-G5**	86:14	>99
***Tm*Pgb-PIP-G6**	85:15	>99
***Tm*Pgb-PIP-G7**	84:16	>99

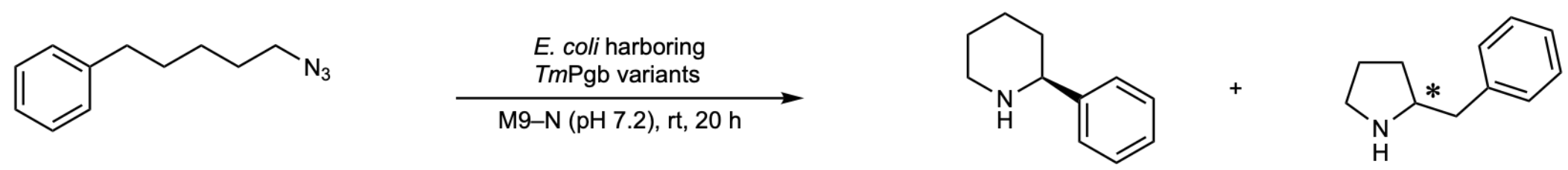

Reaction performed on analytical scale using ***Tm*Pgb-wt**

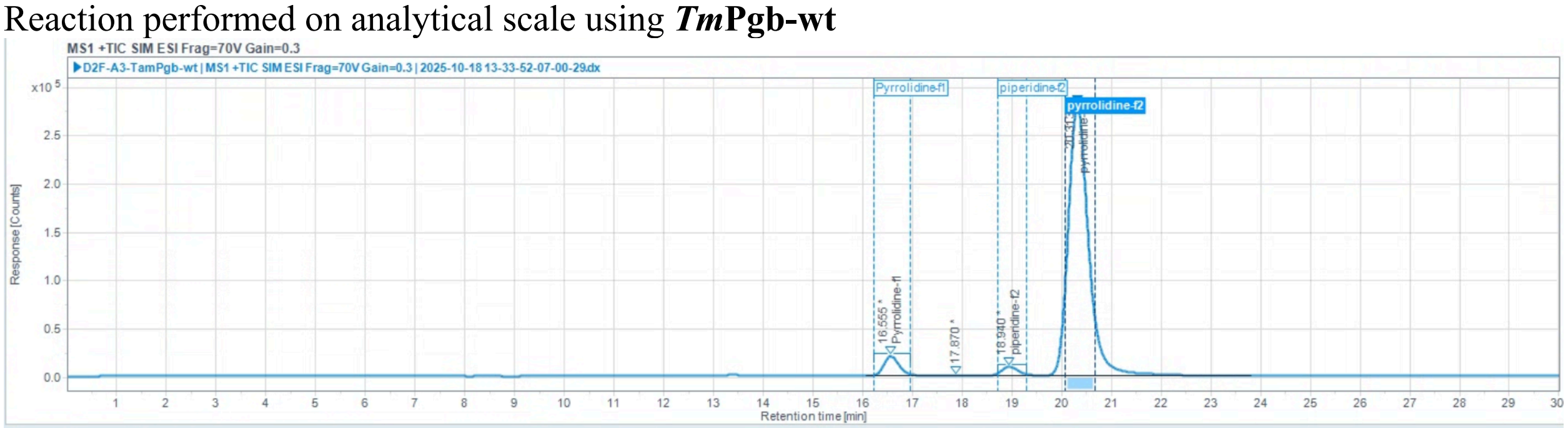

Injection Results

Peaks | Summary

#	Name	Signal description	RT (min)	Area (Counts·s)	Area%	Height (Counts)	Height%	Amount	Concentration	Start time (min)	End time (min)
1	Pyrrolidine-f1	MS1 +TIC SIM ESI Frag=70V Gain=0.3	16.555	455684.495	5.242	20462.948	6.59			16.058	17.526
2		MS1 +TIC SIM ESI Frag=70V Gain=0.3	17.870	4577.763	0.053	199.969	0.06			17.526	18.371
3	piperidine-f2	MS1 +TIC SIM ESI Frag=70V Gain=0.3	18.940	230689.720	2.654	9198.722	2.96			18.371	19.638
4	pyrrolidine-f2	MS1 +TIC SIM ESI Frag=70V Gain=0.3	20.313	8001452.668	92.051	280436.847	90.38			19.638	23.819

Reaction performed on analytical scale using ***Tm*Pgb-PIP-G1-5442**

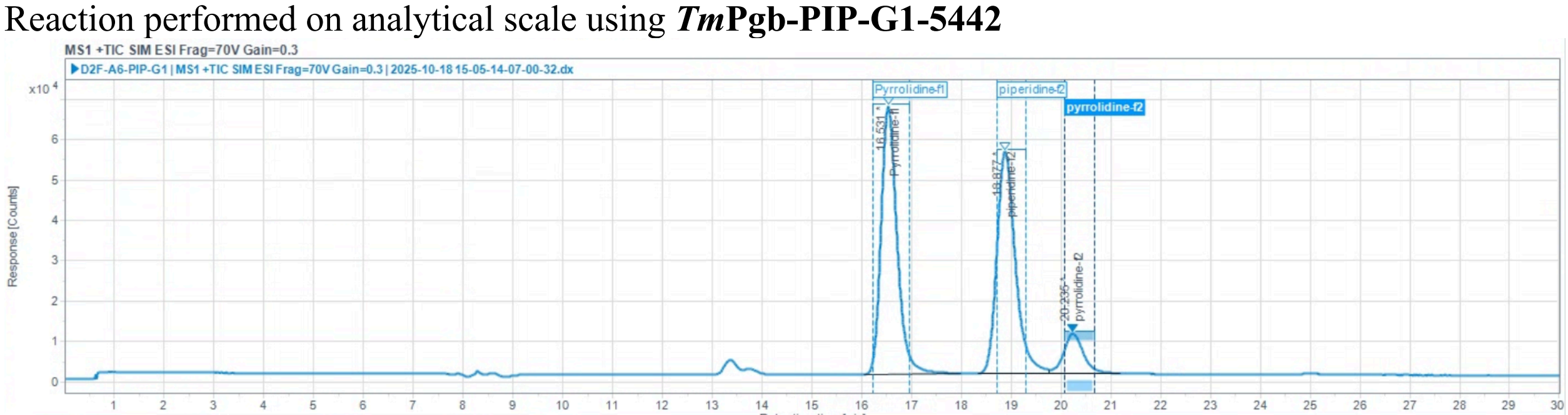

Injection Results

Peaks | Summary

#	Name	Signal description	RT (min)	Area (Counts·s)	Area%	Height (Counts)	Height%	Amount	Concentration	Start time (min)	End time (min)
1	Pyrrolidine-f1	MS1 +TIC SIM ESI Frag=70V Gain=0.3	16.531	1486396.975	46.741	66325.262	50.59			16.038	17.970
2	piperidine-f2	MS1 +TIC SIM ESI Frag=70V Gain=0.3	18.877	1423458.054	44.762	54927.665	41.90			18.330	19.749
3	pyrrolidine-f2	MS1 +TIC SIM ESI Frag=70V Gain=0.3	20.235	270189.383	8.496	9839.649	7.51			19.749	21.173

Reaction performed on analytical scale using ***Tm*Pgb-PIP-G2-5443**

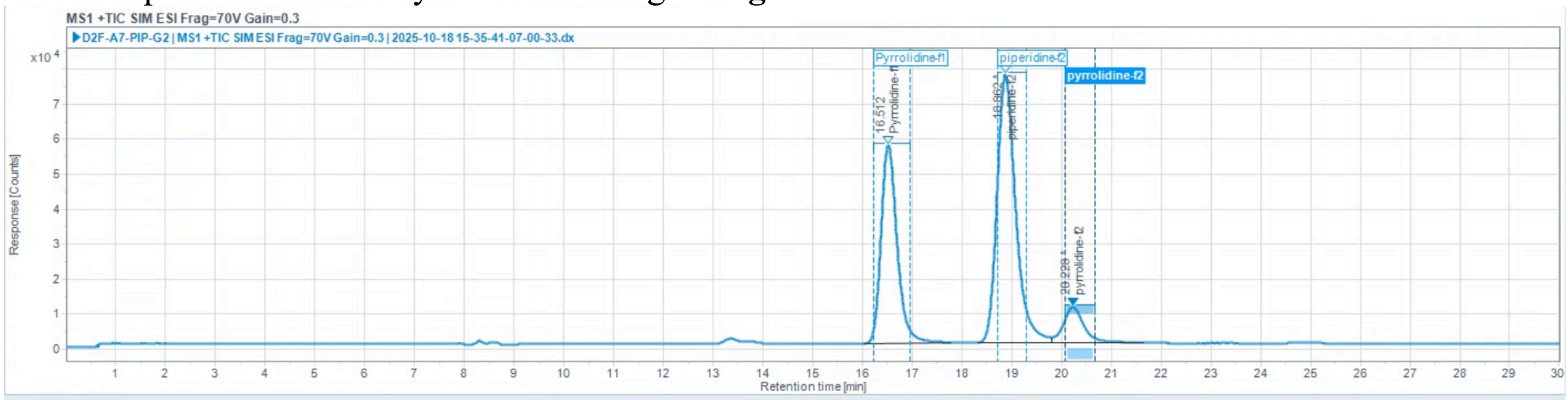

Injection Results

Peaks Summary

#	Name	Signal description	RT (min)	Area (Counts·s)	Area%	Height (Counts)	Height%	Amount	Concentration	Start time (min)	End time (min)
1	Pyrrolidine-f1	MS1 +TIC SIM ESI Frag=70V Gain=0.3	16.512	1270715.320	35.720	56647.764	39.48			16.014	17.782
2	piperidine-f2	MS1 +TIC SIM ESI Frag=70V Gain=0.3	18.862	1996270.480	56.116	76677.708	53.44			18.312	19.794
3	pyrrolidine-f2	MS1 +TIC SIM ESI Frag=70V Gain=0.3	20.228	290421.869	8.164	10165.492	7.08			19.794	21.640

Reaction performed on analytical scale using ***Tm*Pgb-PIP-G3-5444**

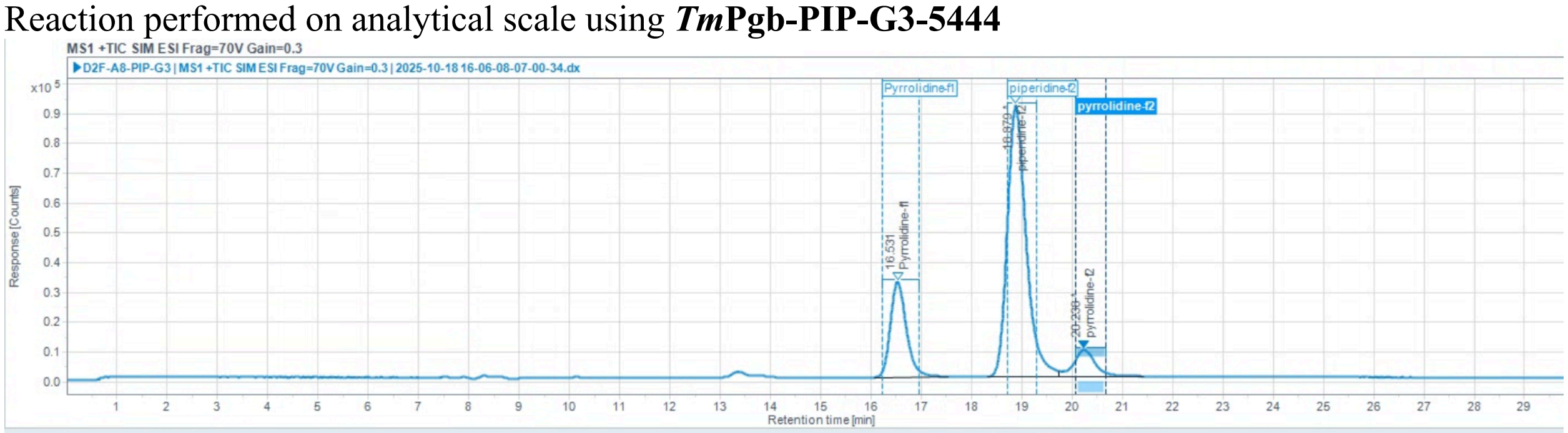

Injection Results

Peaks Summary

#	Name	Signal description	RT (min)	Area (Counts·s)	Area%	Height (Counts)	Height%	Amount	Concentration	Start time (min)	End time (min)
1	Pyrrolidine-f1	MS1 +TIC SIM ESI Frag=70V Gain=0.3	16.531	708546.705	21.126	31871.509	24.13			16.076	17.546
2	piperidine-f2	MS1 +TIC SIM ESI Frag=70V Gain=0.3	18.879	2377421.122	70.884	91218.578	69.05			18.312	19.749
3	pyrrolidine-f2	MS1 +TIC SIM ESI Frag=70V Gain=0.3	20.238	267999.182	7.991	9017.526	6.83			19.749	21.417

Reaction performed on analytical scale using ***Tm*Pgb-PIP-G4-5445**

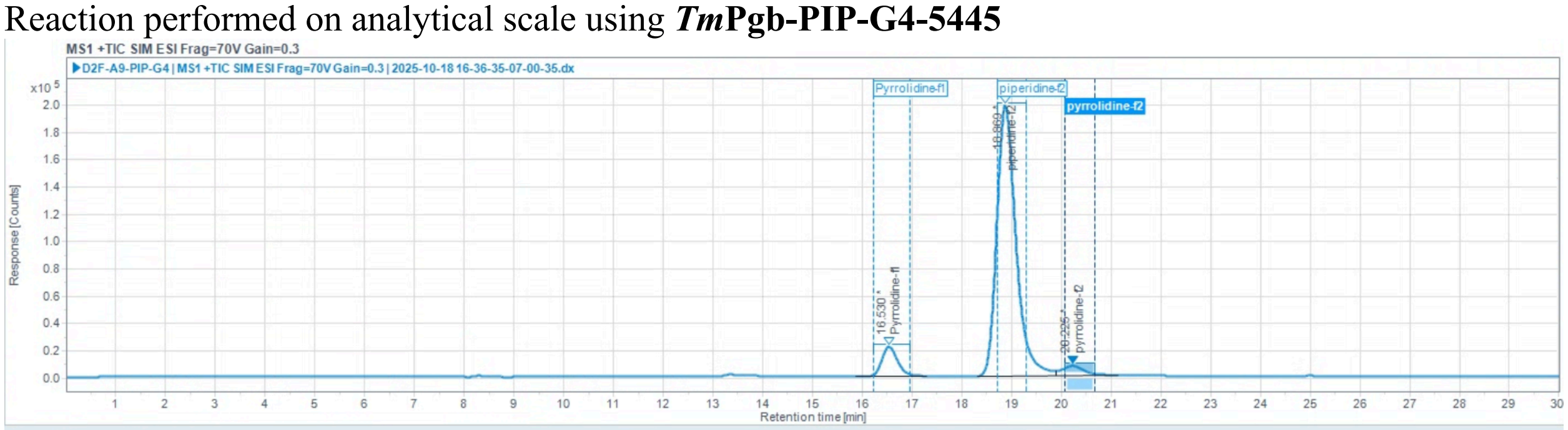

Injection Results

Peaks Summary

#	Name	Signal description	RT (min)	Area (Counts·s)	Area%	Height (Counts)	Height%	Amount	Concentration	Start time (min)	End time (min)
1	Pyrrolidine-f1	MS1 +TIC SIM ESI Frag=70V Gain=0.3	16.530	471100.639	7.840	21447.295	9.45			15.858	17.281
2	piperidine-f2	MS1 +TIC SIM ESI Frag=70V Gain=0.3	18.869	5318512.123	88.514	198256.366	87.37			18.305	19.883
3	pyrrolidine-f2	MS1 +TIC SIM ESI Frag=70V Gain=0.3	20.225	219056.070	3.646	7216.273	3.18			19.883	21.128

Reaction performed on analytical scale using ***Tm*****Pgb-PIP-G5-5446**

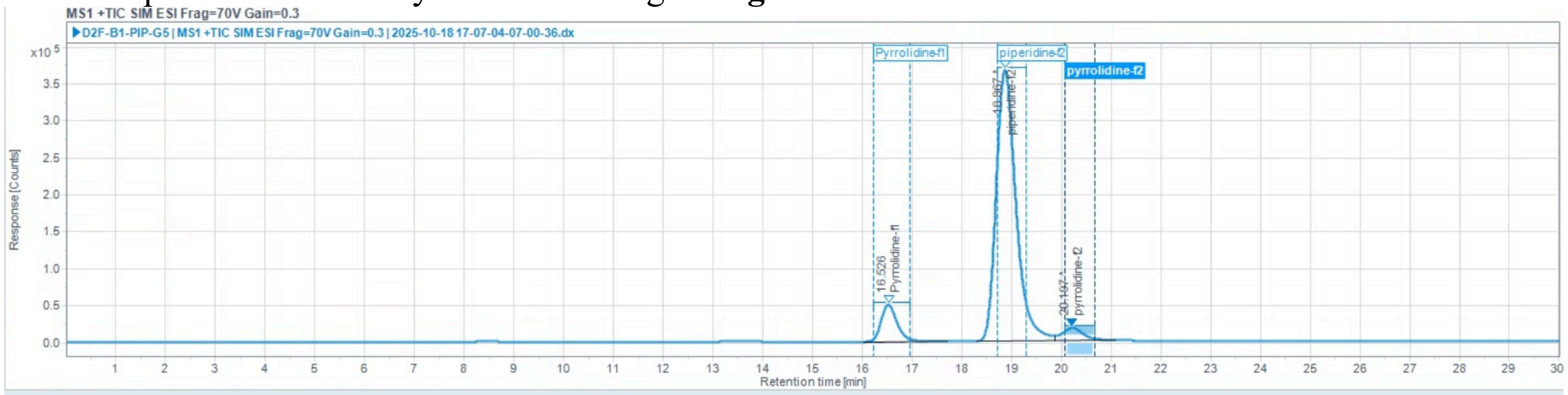

Injection Results

Peaks Summary

#	Name	Signal description	RT (min)	Area (Counts·s)	Area%	Height (Counts)	Height%	Amount	Concentration	Start time (min)	End time (min)
1	Pyrrolidine-f1	MS1 +TIC SIM ESI Frag=70V Gain=0.3	16.526	1133631.338	9.581	50267.241	11.57			16.032	17.708
2	piperidine-f2	MS1 +TIC SIM ESI Frag=70V Gain=0.3	18.867	10183342.844	86.069	367395.020	84.54			18.293	19.861
3	pyrrolidine-f2	MS1 +TIC SIM ESI Frag=70V Gain=0.3	20.197	514638.304	4.350	16941.926	3.90			19.861	21.084

Reaction performed on analytical scale using ***Tm*****Pgb-PIP-G6-5447**

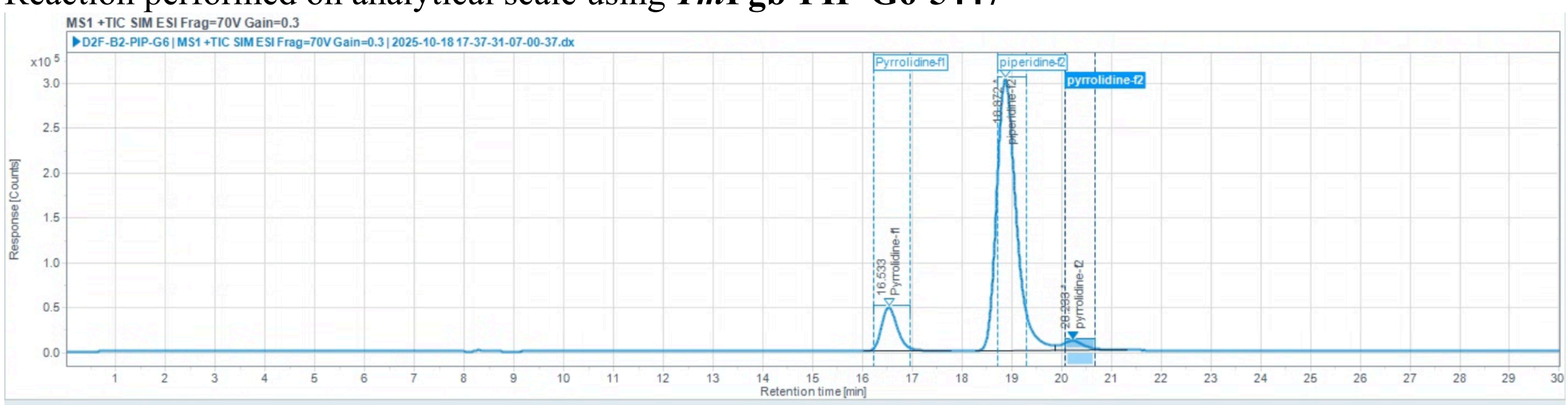

Injection Results

Peaks Summary

#	Name	Signal description	RT (min)	Area (Counts·s)	Area%	Height (Counts)	Height%	Amount	Concentration	Start time (min)	End time (min)
1	Pyrrolidine-f1	MS1 +TIC SIM ESI Frag=70V Gain=0.3	16.533	1115723.894	11.387	49101.793	13.54			16.026	17.782
2	piperidine-f2	MS1 +TIC SIM ESI Frag=70V Gain=0.3	18.872	8326292.544	84.976	302740.448	83.47			18.281	19.861
3	pyrrolidine-f2	MS1 +TIC SIM ESI Frag=70V Gain=0.3	20.233	356352.585	3.637	10841.920	2.99			19.861	21.328

Reaction performed on analytical scale using ***Tm*****Pgb-PIP-G7-5448**

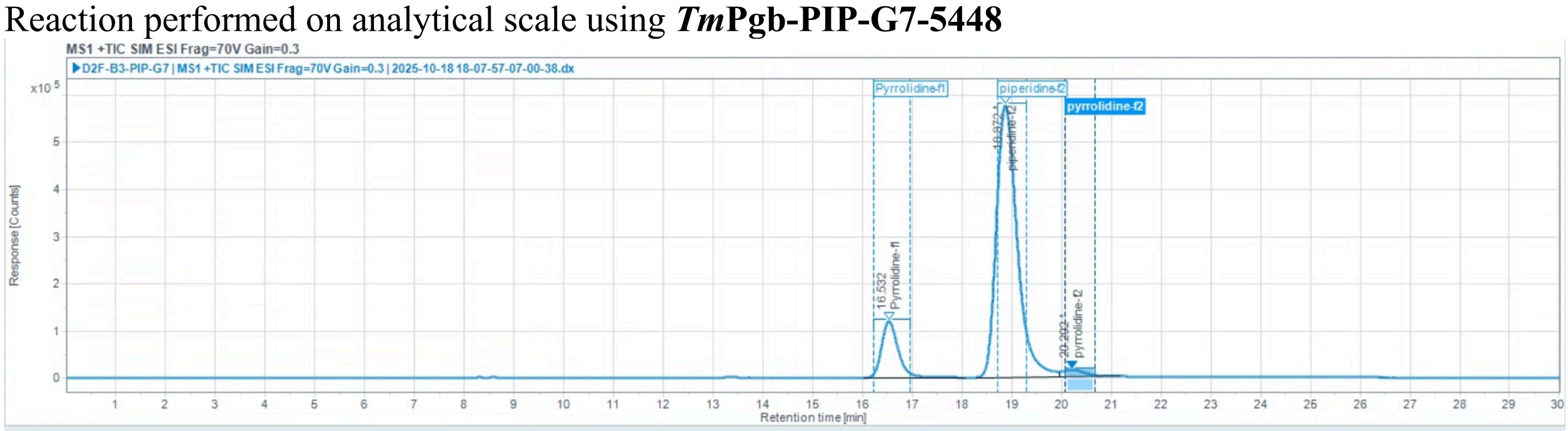

Injection Results

Peaks Summary

#	Name	Signal description	RT (min)	Area (Counts·s)	Area%	Height (Counts)	Height%	Amount	Concentration	Start time (min)	End time (min)
1	Pyrrolidine-f1	MS1 +TIC SIM ESI Frag=70V Gain=0.3	16.532	2771102.938	13.868	119696.705	16.86			16.020	18.075
2	piperidine-f2	MS1 +TIC SIM ESI Frag=70V Gain=0.3	18.872	16744088.324	83.795	575424.514	81.07			18.287	19.950
3	pyrrolidine-f2	MS1 +TIC SIM ESI Frag=70V Gain=0.3	20.202	467014.020	2.337	14659.697	2.07			19.950	21.173

Part 2: Regio- and enantioselectivity analysis of 2a and 3a on reactions performed with GDM-NT variants.

Results analyzed on polar ionic chiral HPLC-MS.

(*rac*)-**2a** (commercially available standard)

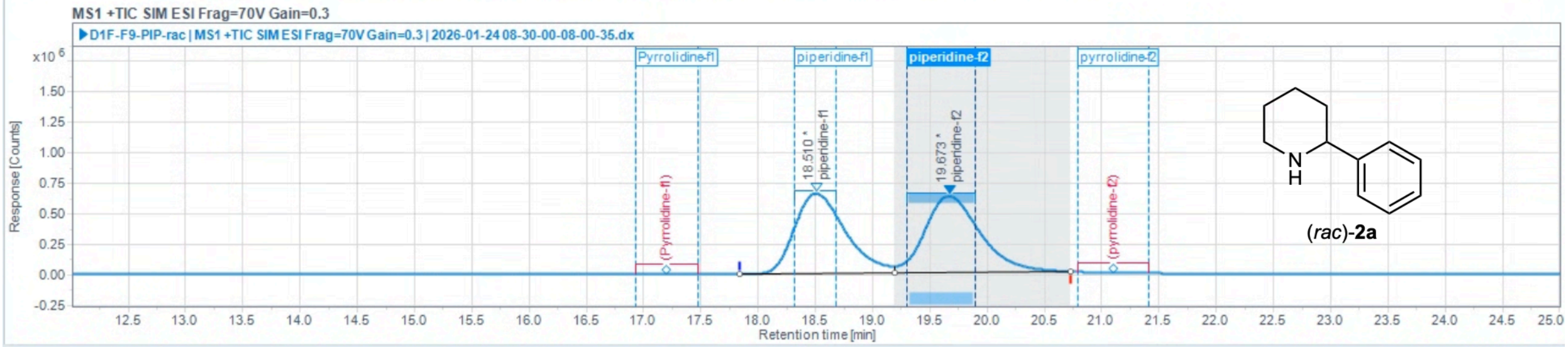

Injection Results

#	Name	Signal description	RT (min)	Area (Counts·s)	Area%	Height (Counts)	Height%	Amount	Concentration	Start time (min)	End time (min)
1	piperidine-f1	MS1 +TIC SIM ESI Frag=70V Gain=0.3	18.510	20366752.140	49.171	656971.736	51.21			17.838	19.188
2	piperidine-f2	MS1 +TIC SIM ESI Frag=70V Gain=0.3	19.673	21053749.561	50.829	625903.993	48.79			19.188	20.723

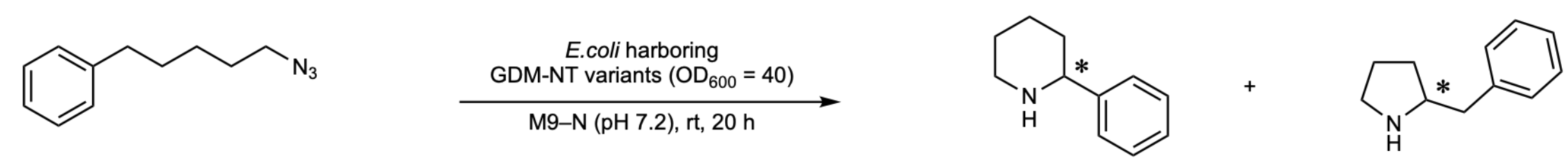

1a **2a** **3a**

Reaction performed on analytical scale using ***Tm*Pgb-wt**: 3:97 rr (**2a**:**3a**), –97% ee favoring (*S*)-**2a**.

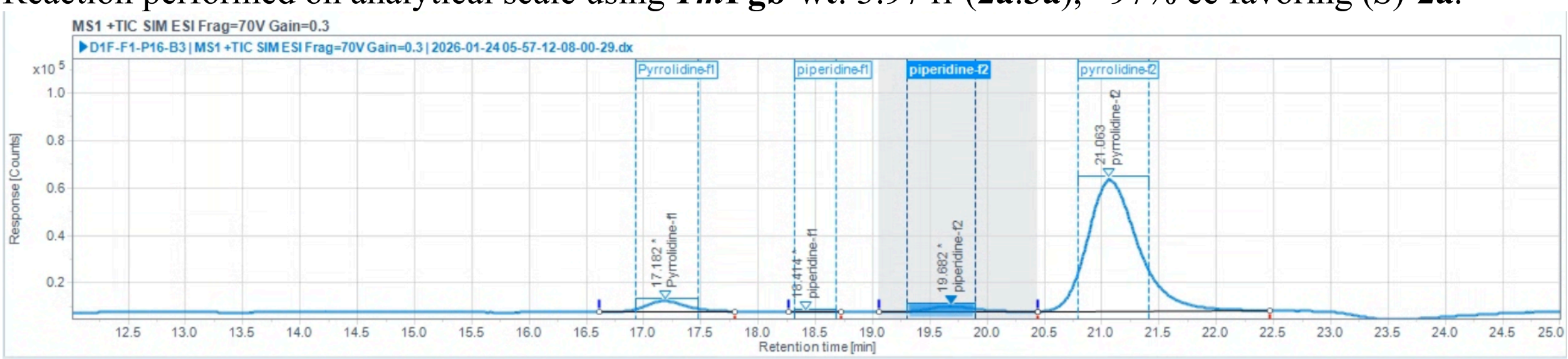

Injection Results

#	Name	Signal description	RT (min)	Area (Counts·s)	Area%	Height (Counts)	Height%	Amount	Concentration	Start time (min)	End time (min)
1	Pyrrolidine-f1	MS1 +TIC SIM ESI Frag=70V Gain=0.3	17.182	114643.960	6.049	4585.096	7.30			16.606	17.796
2	piperidine-f1	MS1 +TIC SIM ESI Frag=70V Gain=0.3	18.414	1009.813	0.053	114.362	0.18			18.259	18.728
3	piperidine-f2	MS1 +TIC SIM ESI Frag=70V Gain=0.3	19.682	64158.247	3.385	2323.900	3.70			19.058	20.442
4	pyrrolidine-f2	MS1 +TIC SIM ESI Frag=70V Gain=0.3	21.063	1715494.866	90.513	55758.236	88.81			20.442	22.465

Table S28. Regioselectivity and enantioselectivity of selected GDM-NT variants using recycled scaffold design.

Variants	rr (**2a**:**3a**)	ee (%)	Variants	rr (**2a**:**3a**)	ee (%)
**GDM-NT-121**	96:4	–40	**GDM-NT-151**	95:5	–69
**GDM-NT-215**	98:2	–24	**GDM-NT-350**	94:6	–45
**GDM-NT-217**	96:4	31	**GDM-NT-356**	99:1	–73
**GDM-NT-226**	>99:1	30	**GDM-NT-359**	98:2	–64
**GDM-NT-227**	94:6	4	**GDM-NT-361**	95:5	–23
**GDM-NT-228**	89:11	50	**GDM-NT-362**	>99:1	89

Reaction performed on analytical scale using **GDM-NT-121**: 96:4 rr (**2a**:**3a**), –40% ee favoring (*S*)-**2a**.

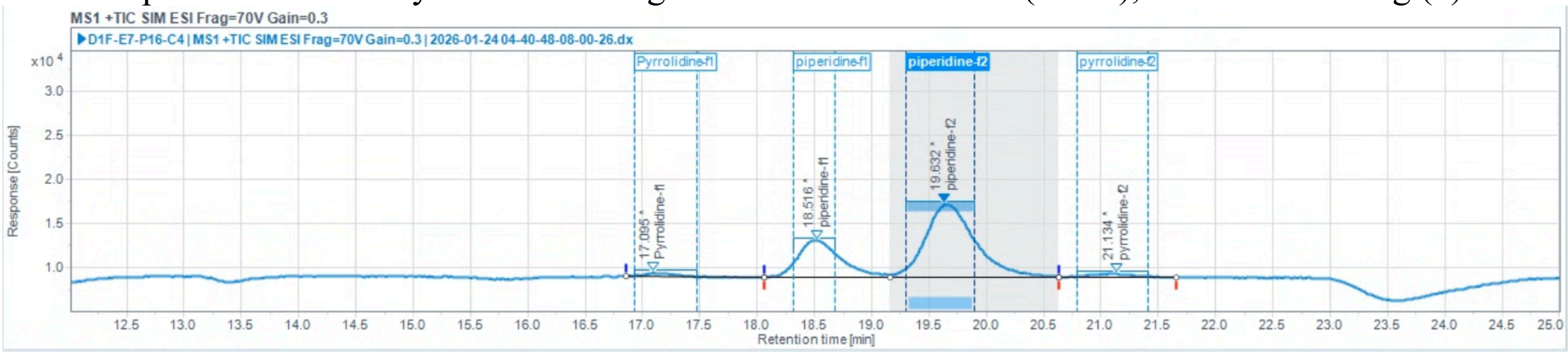

Injection Results

#	Name	Signal description	RT (min)	Area (Counts·s)	Area%	Height (Counts)	Height%	Amount	Concentration	Start time (min)	End time (min)
1	Pyrrolidine-f1	MS1 +TIC SIM ESI Frag=70V Gain=0.3	17.095	5713.548	1.572	413.341	3.16			16.852	18.056
2	piperidine-f1	MS1 +TIC SIM ESI Frag=70V Gain=0.3	18.516	105591.081	29.050	4116.973	31.46			18.056	19.166
3	piperidine-f2	MS1 +TIC SIM ESI Frag=70V Gain=0.3	19.632	244460.026	67.255	8197.223	62.64			19.166	20.627
4	pyrrolidine-f2	MS1 +TIC SIM ESI Frag=70V Gain=0.3	21.134	7718.407	2.123	359.373	2.75			20.627	21.655

Reaction performed on analytical scale using **GDM-NT-215**: 98:2 rr (**2a**:**3a**), –24% ee favoring (*S*)-**2a**.

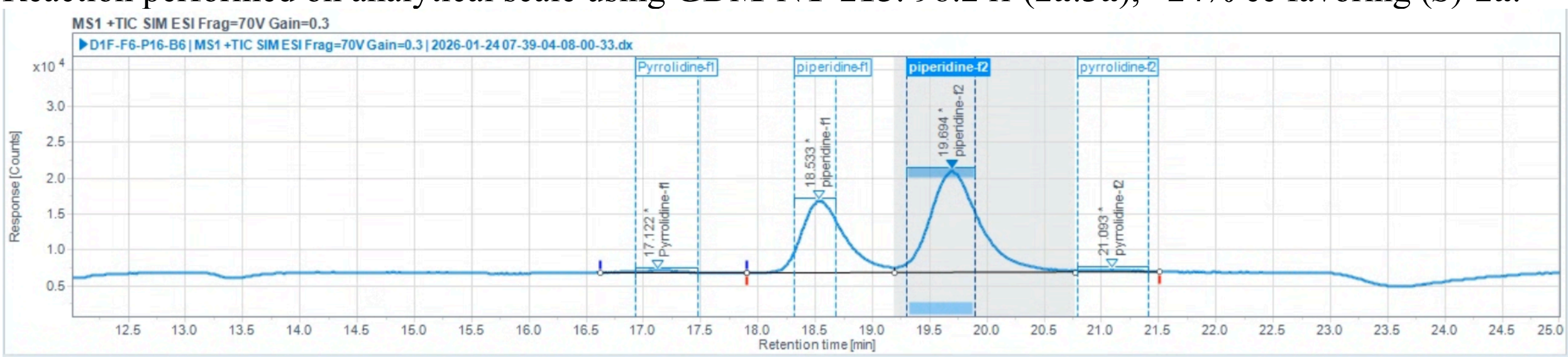

Injection Results

#	Name	Signal description	RT (min)	Area (Counts·s)	Area%	Height (Counts)	Height%	Amount	Concentration	Start time (min)	End time (min)
1	Pyrrolidine-f1	MS1 +TIC SIM ESI Frag=70V Gain=0.3	17.122	8948.585	1.252	351.331	1.41			16.626	17.896
2	piperidine-f1	MS1 +TIC SIM ESI Frag=70V Gain=0.3	18.533	266138.586	37.231	10047.514	40.27			17.896	19.187
3	piperidine-f2	MS1 +TIC SIM ESI Frag=70V Gain=0.3	19.694	430942.757	60.285	14207.662	56.95			19.187	20.772
4	pyrrolidine-f2	MS1 +TIC SIM ESI Frag=70V Gain=0.3	21.093	8809.530	1.232	341.911	1.37			20.772	21.511

Reaction performed on analytical scale using **GDM-NT-217**: 96:4 rr (**2a**:**3a**), 31% ee favoring (*R*)-**2a**.

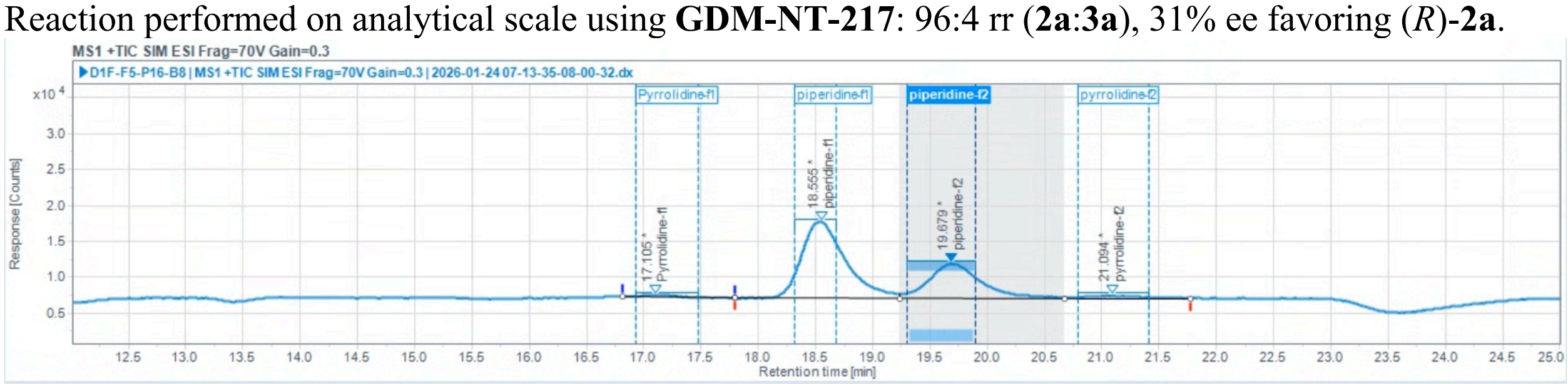

Injection Results

#	Name	Signal description	RT (min)	Area (Counts·s)	Area%	Height (Counts)	Height%	Amount	Concentration	Start time (min)	End time (min)
1	Pyrrolidine-f1	MS1 +TIC SIM ESI Frag=70V Gain=0.3	17.105	4883.638	1.075	270.723	1.68			16.811	17.800
2	piperidine-f1	MS1 +TIC SIM ESI Frag=70V Gain=0.3	18.555	285303.454	62.799	10671.048	66.10			17.800	19.230
3	piperidine-f2	MS1 +TIC SIM ESI Frag=70V Gain=0.3	19.679	151843.229	33.423	4793.964	29.70			19.230	20.670
4	pyrrolidine-f2	MS1 +TIC SIM ESI Frag=70V Gain=0.3	21.094	12283.893	2.704	406.988	2.52			20.670	21.778

Reaction performed on analytical scale using **GDM-NT-226**: >99:1 rr (**2a**:**3a**), 30% ee favoring (*R*)-**2a**.

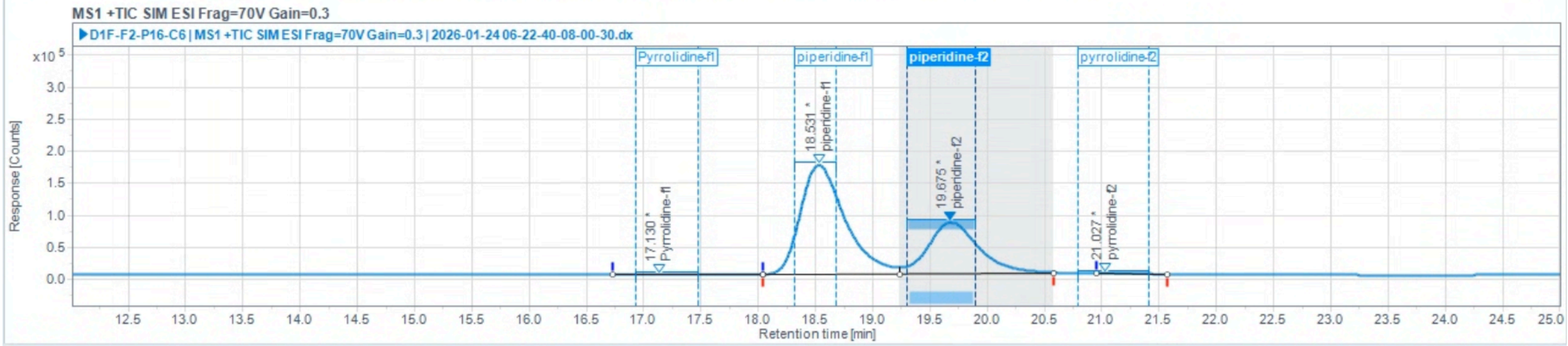

Injection Results

Peaks | Summary

#	Name	Signal description	RT (min)	Area (Counts·s)	Area%	Height (Counts)	Height%	Amount	Concentration	Start time (min)	End time (min)
1	Pyrrolidine-f1	MS1 +TIC SIM ESI Frag=70V Gain=0.3	17.130	1871.163	0.026	565.766	0.22			16.729	18.044
2	piperidine-f1	MS1 +TIC SIM ESI Frag=70V Gain=0.3	18.531	4647606.653	64.790	170386.701	67.68			18.044	19.230
3	piperidine-f2	MS1 +TIC SIM ESI Frag=70V Gain=0.3	19.675	2520856.918	35.142	80549.085	31.99			19.230	20.579
4	pyrrolidine-f2	MS1 +TIC SIM ESI Frag=70V Gain=0.3	21.027	3029.448	0.042	267.841	0.11			20.957	21.573

Reaction performed on analytical scale using **GDM-NT-227**: 94:6 rr (**2a**:**3a**), 4% ee favoring (*R*)-**2a**.

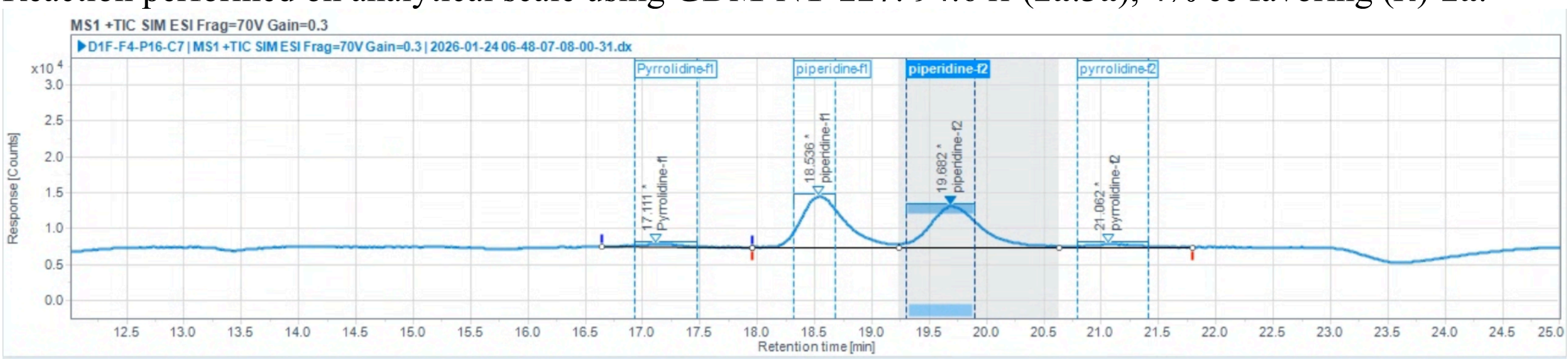

Injection Results

Peaks | Summary

#	Name	Signal description	RT (min)	Area (Counts·s)	Area%	Height (Counts)	Height%	Amount	Concentration	Start time (min)	End time (min)
1	Pyrrolidine-f1	MS1 +TIC SIM ESI Frag=70V Gain=0.3	17.111	11245.983	2.894	464.415	3.37			16.647	17.960
2	piperidine-f1	MS1 +TIC SIM ESI Frag=70V Gain=0.3	18.536	189183.832	48.690	7150.281	51.87			17.960	19.230
3	piperidine-f2	MS1 +TIC SIM ESI Frag=70V Gain=0.3	19.682	175706.389	45.221	5766.752	41.83			19.230	20.629
4	pyrrolidine-f2	MS1 +TIC SIM ESI Frag=70V Gain=0.3	21.062	12413.295	3.195	404.340	2.93			20.629	21.799

Reaction performed on analytical scale using **GDM-NT-228**: 89:11 rr (**2a**:**3a**), 50% ee favoring (*R*)-**2a**.

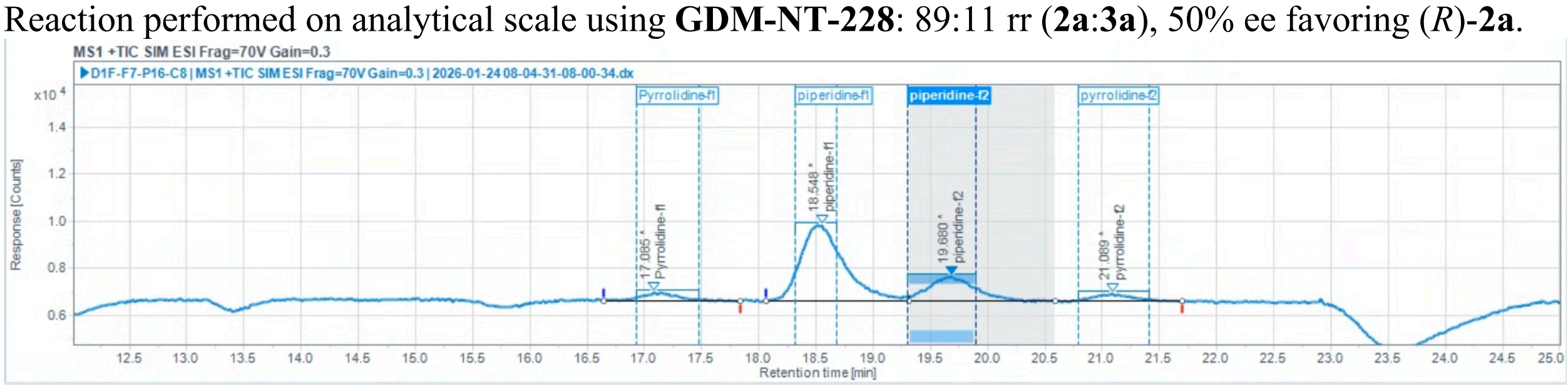

Injection Results

Peaks | Summary

#	Name	Signal description	RT (min)	Area (Counts·s)	Area%	Height (Counts)	Height%	Amount	Concentration	Start time (min)	End time (min)
1	Pyrrolidine-f1	MS1 +TIC SIM ESI Frag=70V Gain=0.3	17.085	7995.271	6.274	362.023	7.35			16.647	17.837
2	piperidine-f1	MS1 +TIC SIM ESI Frag=70V Gain=0.3	18.548	85353.398	66.974	3217.096	65.30			18.056	19.304
3	piperidine-f2	MS1 +TIC SIM ESI Frag=70V Gain=0.3	19.680	28645.553	22.477	1042.446	21.16			19.304	20.588
4	pyrrolidine-f2	MS1 +TIC SIM ESI Frag=70V Gain=0.3	21.089	5448.273	4.275	304.924	6.19			20.588	21.696

Reaction performed on analytical scale using **GDM-NT-151**: 95:5 rr (**2a**:**3a**), –69% ee favoring (*S*)-**2a**.

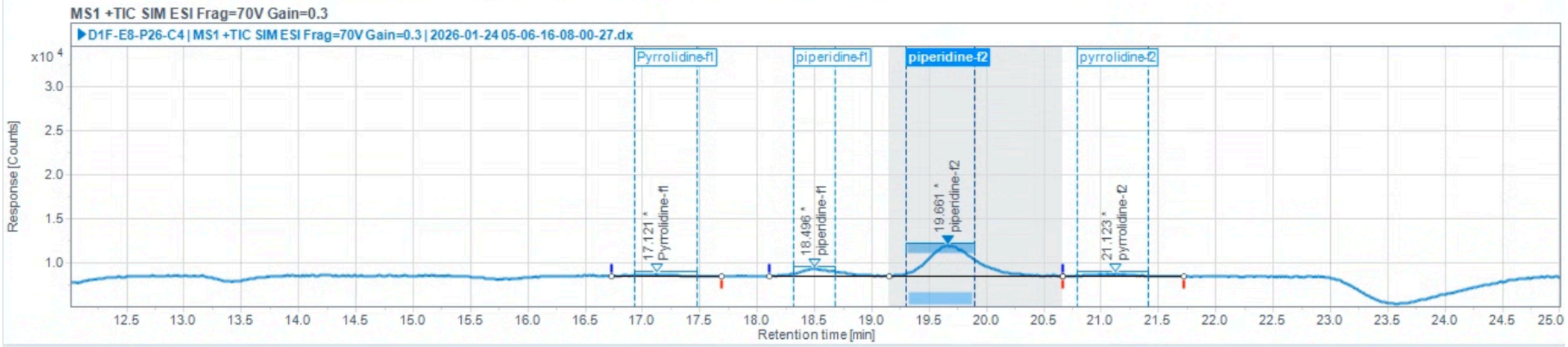

Injection Results

Peaks | Summary

#	Name	Signal description	RT (min)	Area (Counts·s)	Area%	Height (Counts)	Height%	Amount	Concentration	Start time (min)	End time (min)
1	Pyrrolidine-f1	MS1 +TIC SIM ESI Frag=70V Gain=0.3	17.121	2733.013	2.214	180.961	3.88			16.729	17.694
2	piperidine-f1	MS1 +TIC SIM ESI Frag=70V Gain=0.3	18.496	18300.904	14.823	797.049	17.07			18.110	19.155
3	piperidine-f2	MS1 +TIC SIM ESI Frag=70V Gain=0.3	19.661	98460.233	79.748	3461.337	74.14			19.155	20.659
4	pyrrolidine-f2	MS1 +TIC SIM ESI Frag=70V Gain=0.3	21.123	3969.436	3.215	229.479	4.92			20.659	21.716

Reaction performed on analytical scale using **GDM-NT-350**: 94:6 rr (**2a**:**3a**), –45% ee favoring (*S*)-**2a**.

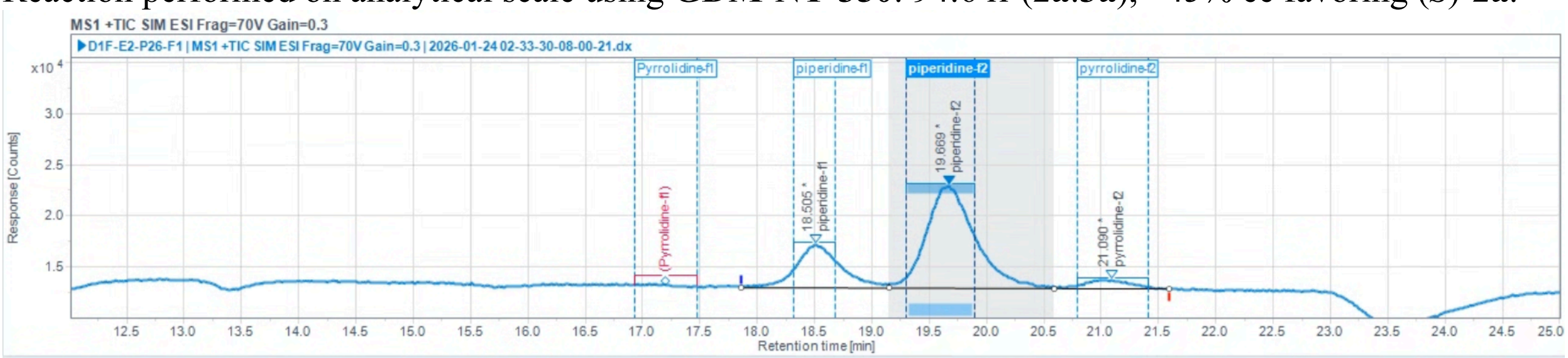

Injection Results

Peaks | Summary

#	Name	Signal description	RT (min)	Area (Counts·s)	Area%	Height (Counts)	Height%	Amount	Concentration	Start time (min)	End time (min)
1	piperidine-f1	MS1 +TIC SIM ESI Frag=70V Gain=0.3	18.505	113819.634	26.055	4175.578	27.63			17.864	19.155
2	piperidine-f2	MS1 +TIC SIM ESI Frag=70V Gain=0.3	19.669	297941.707	68.202	10046.695	66.48			19.155	20.591
3	pyrrolidine-f2	MS1 +TIC SIM ESI Frag=70V Gain=0.3	21.090	25089.895	5.743	890.241	5.89			20.591	21.590

Reaction performed on analytical scale using **GDM-NT-356**: 99:1 rr (**2a**:**3a**), –73% ee favoring (*S*)-**2a**.

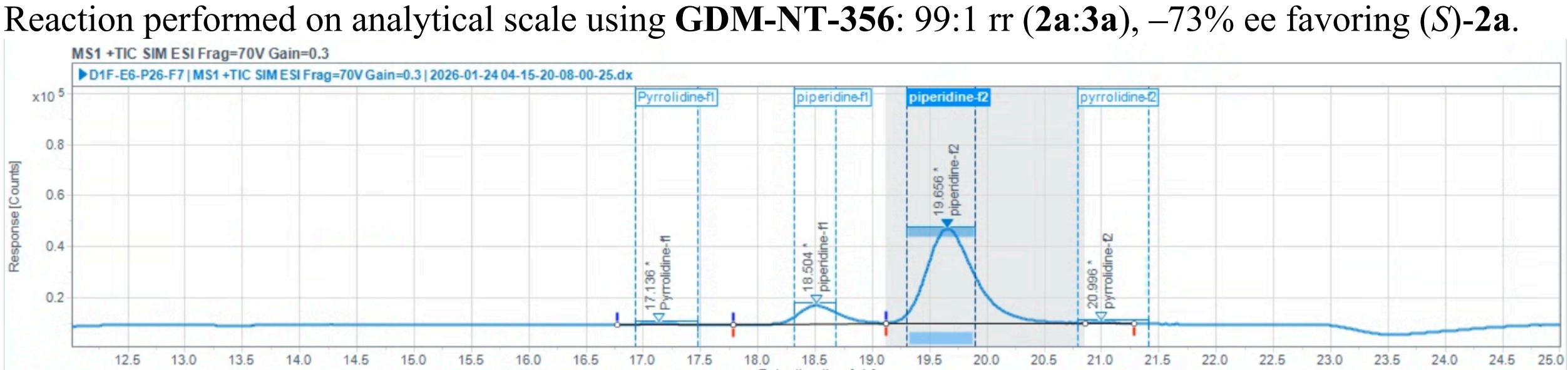

Injection Results

Peaks | Summary

#	Name	Signal description	RT (min)	Area (Counts·s)	Area%	Height (Counts)	Height%	Amount	Concentration	Start time (min)	End time (min)
1	Pyrrolidine-f1	MS1 +TIC SIM ESI Frag=70V Gain=0.3	17.136	6839.246	0.532	396.744	0.88			16.770	17.779
2	piperidine-f1	MS1 +TIC SIM ESI Frag=70V Gain=0.3	18.504	171826.561	13.371	7148.996	15.91			17.779	19.121
3	piperidine-f2	MS1 +TIC SIM ESI Frag=70V Gain=0.3	19.656	1100275.660	85.619	37011.923	82.39			19.121	20.854
4	pyrrolidine-f2	MS1 +TIC SIM ESI Frag=70V Gain=0.3	20.996	6149.236	0.479	367.435	0.82			20.854	21.285

Reaction performed on analytical scale using **GDM-NT-359**: 98:2 rr (**2a**:**3a**), –64% ee favoring (*S*)-**2a**.

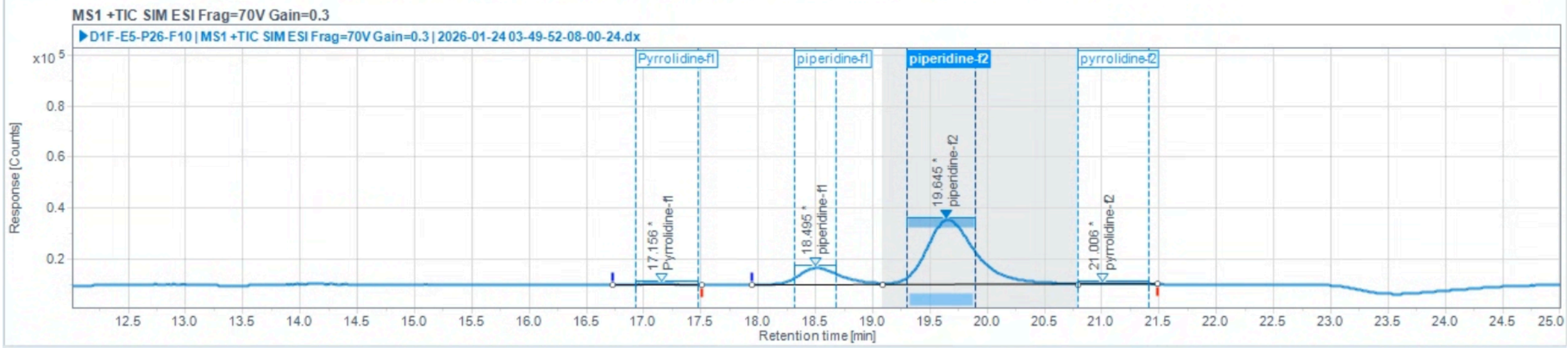

Injection Results

#	Name	Signal description	RT (min)	Area (Counts·s)	Area%	Height (Counts)	Height%	Amount	Concentration	Start time (min)	End time (min)
1	Pyrrolidine-f1	MS1 +TIC SIM ESI Frag=70V Gain=0.3	17.156	3479.897	0.366	288.288	0.88			16.729	17.509
2	piperidine-f1	MS1 +TIC SIM ESI Frag=70V Gain=0.3	18.495	168656.341	17.722	6556.707	20.02			17.950	19.091
3	piperidine-f2	MS1 +TIC SIM ESI Frag=70V Gain=0.3	19.645	767392.089	80.635	25416.737	77.61			19.091	20.793
4	pyrrolidine-f2	MS1 +TIC SIM ESI Frag=70V Gain=0.3	21.006	12155.011	1.277	487.839	1.49			20.793	21.491

Reaction performed on analytical scale using **GDM-NT-361**: 95:5 rr (**2a**:**3a**), –23% ee favoring (*S*)-**2a**.

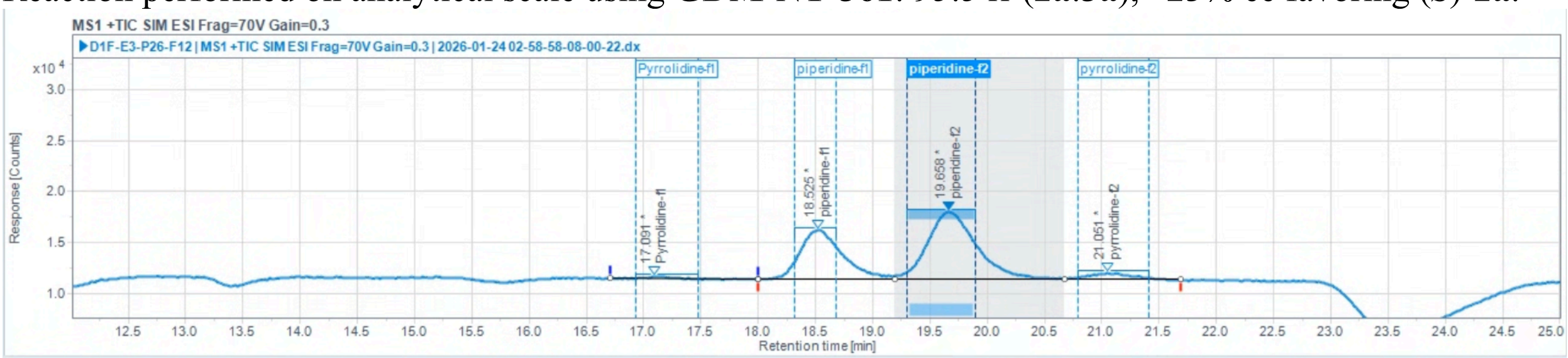

Injection Results

#	Name	Signal description	RT (min)	Area (Counts·s)	Area%	Height (Counts)	Height%	Amount	Concentration	Start time (min)	End time (min)
1	Pyrrolidine-f1	MS1 +TIC SIM ESI Frag=70V Gain=0.3	17.091	2242.808	0.658	225.259	1.82			16.708	17.992
2	piperidine-f1	MS1 +TIC SIM ESI Frag=70V Gain=0.3	18.525	124639.446	36.564	4826.447	38.92			17.992	19.187
3	piperidine-f2	MS1 +TIC SIM ESI Frag=70V Gain=0.3	19.658	197672.871	57.989	6663.560	53.73			19.187	20.670
4	pyrrolidine-f2	MS1 +TIC SIM ESI Frag=70V Gain=0.3	21.051	16326.845	4.790	686.799	5.54			20.670	21.683

Reaction performed on analytical scale using **GDM-NT-362**: >99:1 rr (**2a**:**3a**), 89% ee favoring (*R*)-**2a**.

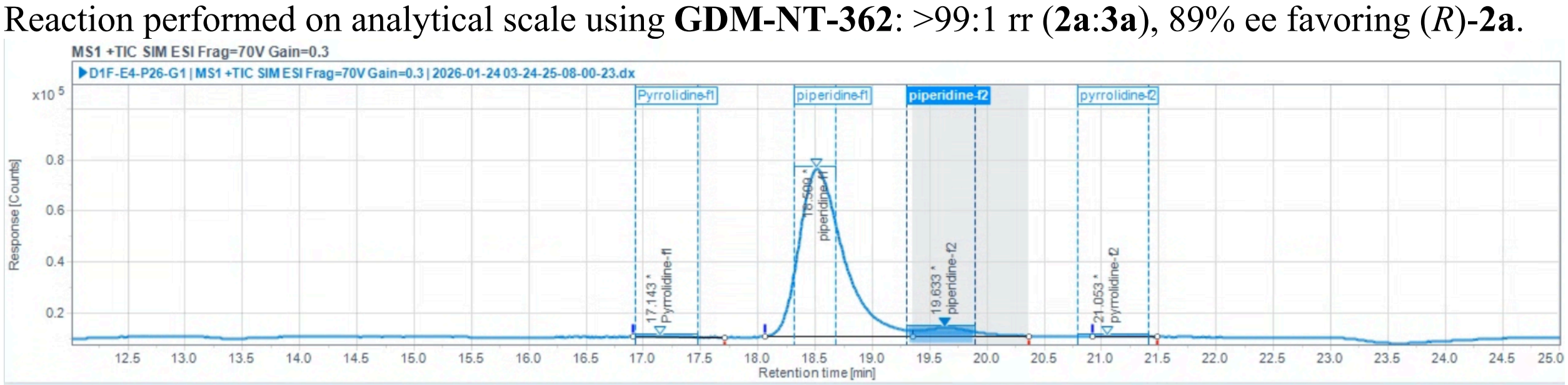

Injection Results

#	Name	Signal description	RT (min)	Area (Counts·s)	Area%	Height (Counts)	Height%	Amount	Concentration	Start time (min)	End time (min)
1	Pyrrolidine-f1	MS1 +TIC SIM ESI Frag=70V Gain=0.3	17.143	2700.514	0.144	228.557	0.33			16.914	17.714
2	piperidine-f1	MS1 +TIC SIM ESI Frag=70V Gain=0.3	18.509	1770314.949	94.320	65740.064	94.69			18.062	19.358
3	piperidine-f2	MS1 +TIC SIM ESI Frag=70V Gain=0.3	19.633	102716.631	5.473	3348.211	4.82			19.358	20.360
4	pyrrolidine-f2	MS1 +TIC SIM ESI Frag=70V Gain=0.3	21.053	1195.317	0.064	110.671	0.16			20.916	21.491

2-phenylpiperidine (**2a** and **2b**)
Chiral separation method: polar ionic HPLC-MS using *dual* Agilent InfinityLab Poroshell 120 Chiral-V, 2.7 µm, 2.1 × 150 mm; eluent: 40% MeOH (0.2 w% NH_4CO_2H), 60% H_2O (20 mM NH_4CO_2H, pH 4.0 adjusted with HCO_2H); flow rate: 0.2 mL/min. t_R = 33.601 min, t_S = 35.347 min.

Reaction performed on analytical scale using **GDM-NT-507**: 98:2 rr (**2a**:**3a**), 52% ee favoring (*R*)-**2a**.

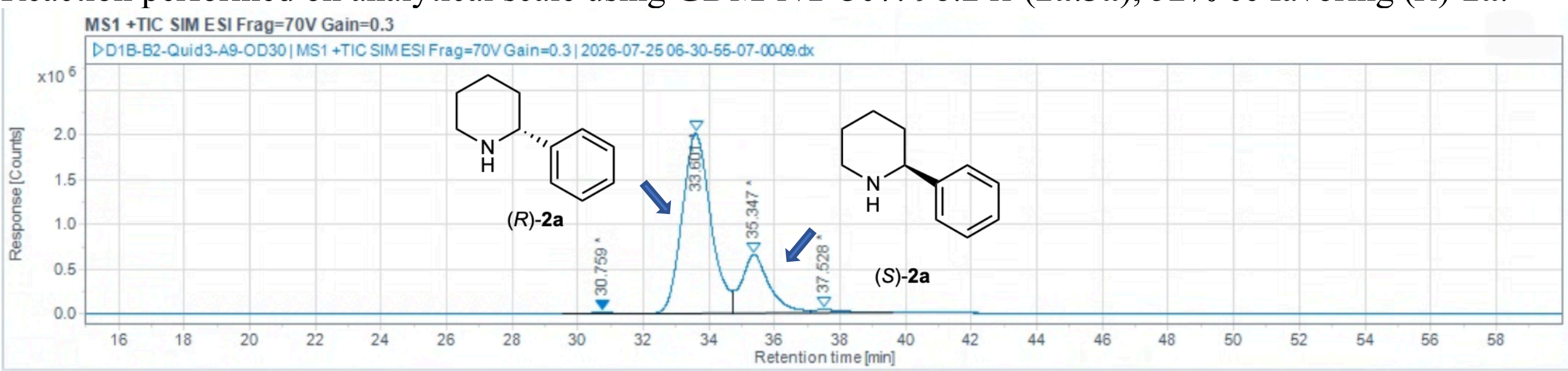

Injection Results

Peaks | Summary

#	Name	Signal description	RT (min)	Area (Counts-s)	Area%	Height (Counts)	Height%	Start time (min)	End time (min)
1		MS1 +TIC SIM ESI Frag=70V Gain=0.3	30.759	529208.228	0.325	10346.358	0.38	29.522	31.848
2		MS1 +TIC SIM ESI Frag=70V Gain=0.3	33.601	121211635.787	74.409	2018226.112	74.29	31.848	34.710
3		MS1 +TIC SIM ESI Frag=70V Gain=0.3	35.347	38645343.611	23.724	651550.876	23.98	34.710	37.096
4		MS1 +TIC SIM ESI Frag=70V Gain=0.3	37.528	2512253.740	1.542	36505.739	1.34	37.096	39.601

Reaction performed on analytical scale using **GDM-NT-534**: >99:1 rr (**2a**:**3a**), 63% ee favoring (*R*)-**2a**.

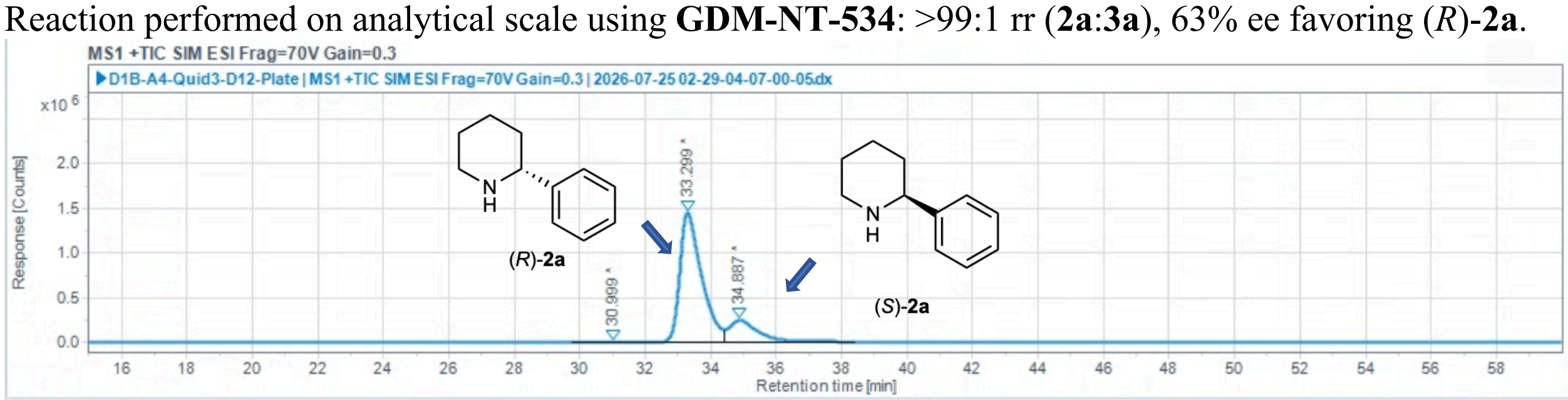

Injection Results

Peaks | Summary

#	Name	Signal description	RT (min)	Area (Counts-s)	Area%	Height (Counts)	Height%	Start time (min)	End time (min)
1		MS1 +TIC SIM ESI Frag=70V Gain=0.3	30.999	135907.235	0.166	2556.737	0.15	29.760	32.265
2		MS1 +TIC SIM ESI Frag=70V Gain=0.3	33.299	66758429.687	81.466	1449242.570	85.51	32.265	34.436
3		MS1 +TIC SIM ESI Frag=70V Gain=0.3	34.887	15051558.847	18.368	242966.759	14.34	34.436	38.408

Reaction performed on analytical scale using **GDM-NT-589**: >99:1 rr (**2a**:**3a**), 94% ee favoring (*R*)-**2a**.

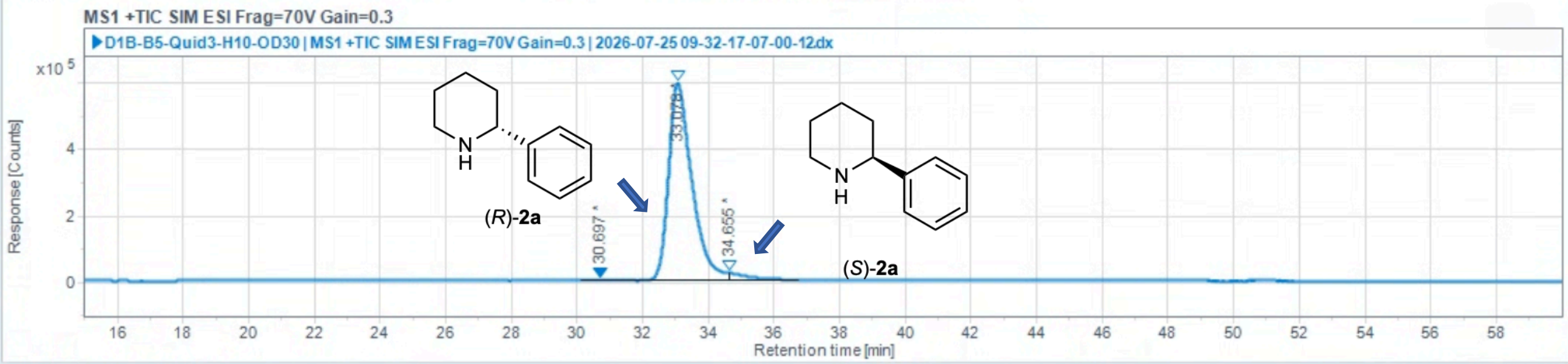

Injection Results

Peaks | Summary

#	Name	Signal description	RT (min)	Area (Counts·s)	Area%	Height (Counts)	Height%	Start time (min)	End time (min)
1		MS1 +TIC SIM ESI Frag=70V Gain=0.3	30.697	31343.261	0.104	826.902	0.13	30.118	32.070
2		MS1 +TIC SIM ESI Frag=70V Gain=0.3	33.078	29271040.482	97.077	594655.374	96.36	32.070	34.651
3		MS1 +TIC SIM ESI Frag=70V Gain=0.3	34.655	849908.043	2.819	21660.289	3.51	34.651	36.738

Part 3: Regio- and enantioselectivity analysis of substrate scope study on reactions performed with ***Tm*Pgb-PIP-G7-5455**

Results analyzed on polar ionic chiral HPLC-MS.

2-phenylpiperidine (**2a** and **2b**)

Chiral separation method: polar ionic HPLC-MS using *dual* Agilent InfinityLab Poroshell 120 Chiral-V, 2.7 µm, 2.1 × 150 mm; eluent: 40% MeOH (0.2 w% NH_4CO_2H), 60% H_2O (20 mM NH_4CO_2H, pH 4.0 adjusted with HCO_2H); flow rate: 0.2 mL/min. t_R = 31.434 min, t_S = 33.346 min.

(*rac*)-**2a** (commercially available standard):

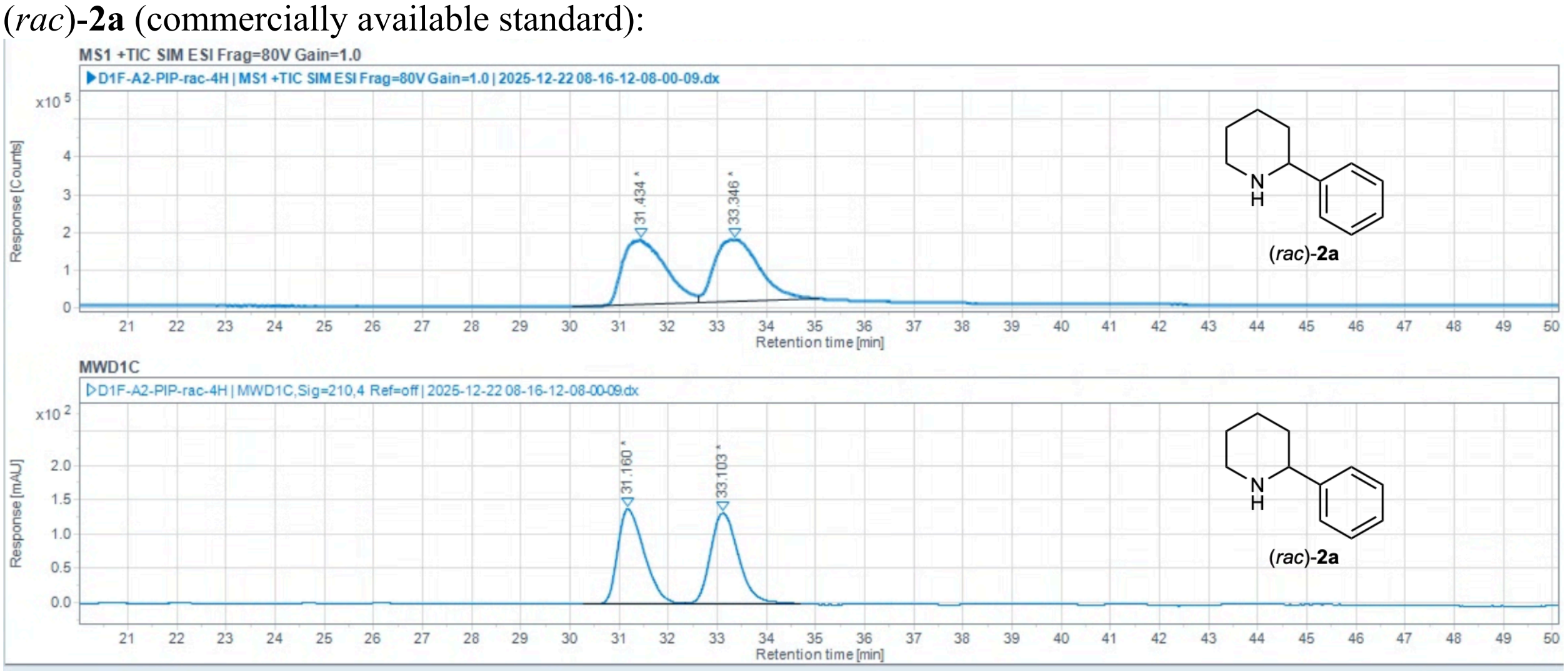

Injection Results

Peaks | Summary

#	Name	Signal description	RT (min)	Area	Area%	Height	Height%	Amou	Concentra	Start time (	End t
2		MS1 +TIC SIM ESI Frag=80V Gain	31.434	10142139.118	49.436	170479.105	50.76			30.059	2.614
4		MS1 +TIC SIM ESI Frag=80V Gain	33.346	10373701.900	50.564	165350.870	49.24			32.614	5.059
1		MWD1C,Sig=210,4 Ref=off	31.160	5211.883	49.692	139.025	51.24			30.277	2.342
3		MWD1C,Sig=210,4 Ref=off	33.103	5276.424	50.308	132.275	48.76			32.342	4.679

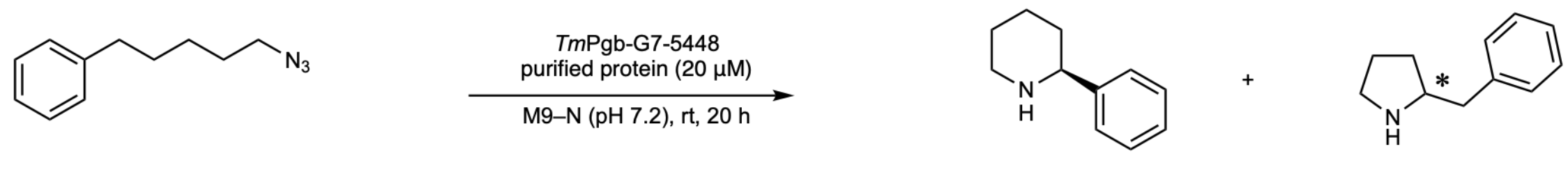

1a **2a** **3a**

Reaction performed on analytical scale using ***Tm*****Pgb-PIP-G7-5448**: 81:19 rr, >99% ee favoring (*S*)-**2a**.

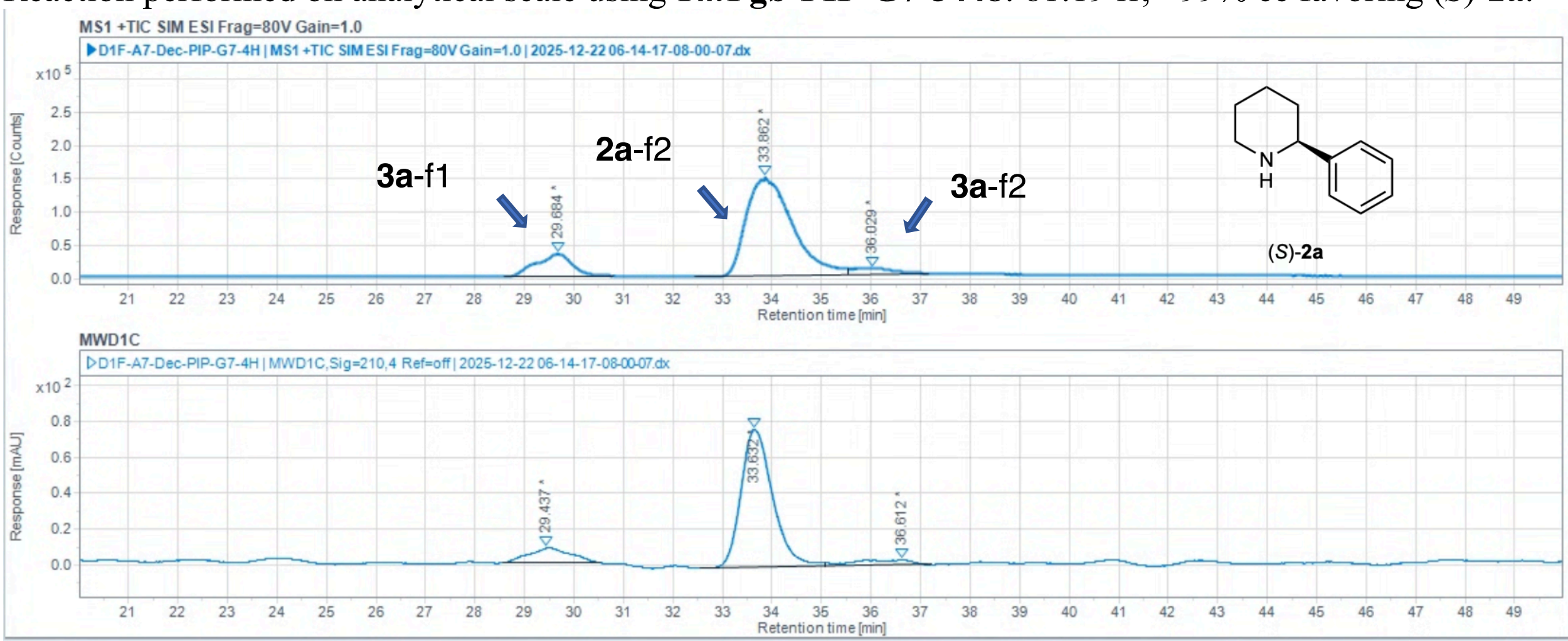

Injection Results

Peaks | Summary

#	Name	Signal description	RT (min)	Area	Area%	Height	Height%	Amou	Concentra	Start time (	End t
2		MS1 +TIC SIM ESI Frag=80V Gain	29.684	1667976.292	14.392	33368.448	17.60			28.590	0.794
4		MS1 +TIC SIM ESI Frag=80V Gain	33.862	9426293.287	81.335	147120.682	77.61			32.439	5.542
5		MS1 +TIC SIM ESI Frag=80V Gain	36.029	495131.124	4.272	9086.690	4.79			35.542	7.174
1		MWD1C,Sig=210,4 Ref=off	29.437	492.277	11.877	8.257	9.39			28.550	0.545
3		MWD1C,Sig=210,4 Ref=off	33.632	3409.544	82.262	76.959	87.50			32.539	5.078
6		MWD1C,Sig=210,4 Ref=off	36.612	242.942	5.861	2.733	3.11			35.078	7.214

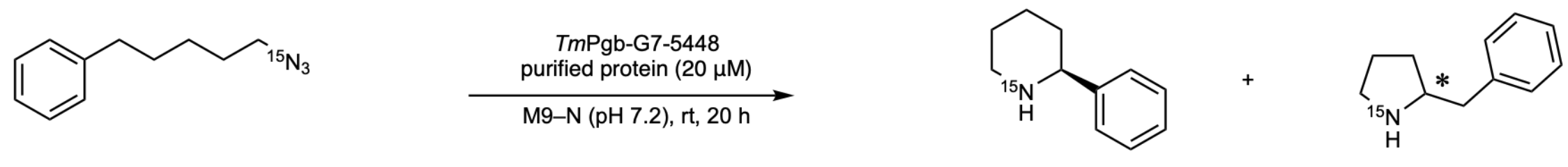

1b **2b** **3b**

Reaction performed on analytical scale using ***Tm*Pgb-PIP-G7-5448**: 78:22 rr, >99% ee favoring (*S*)-**2b**.

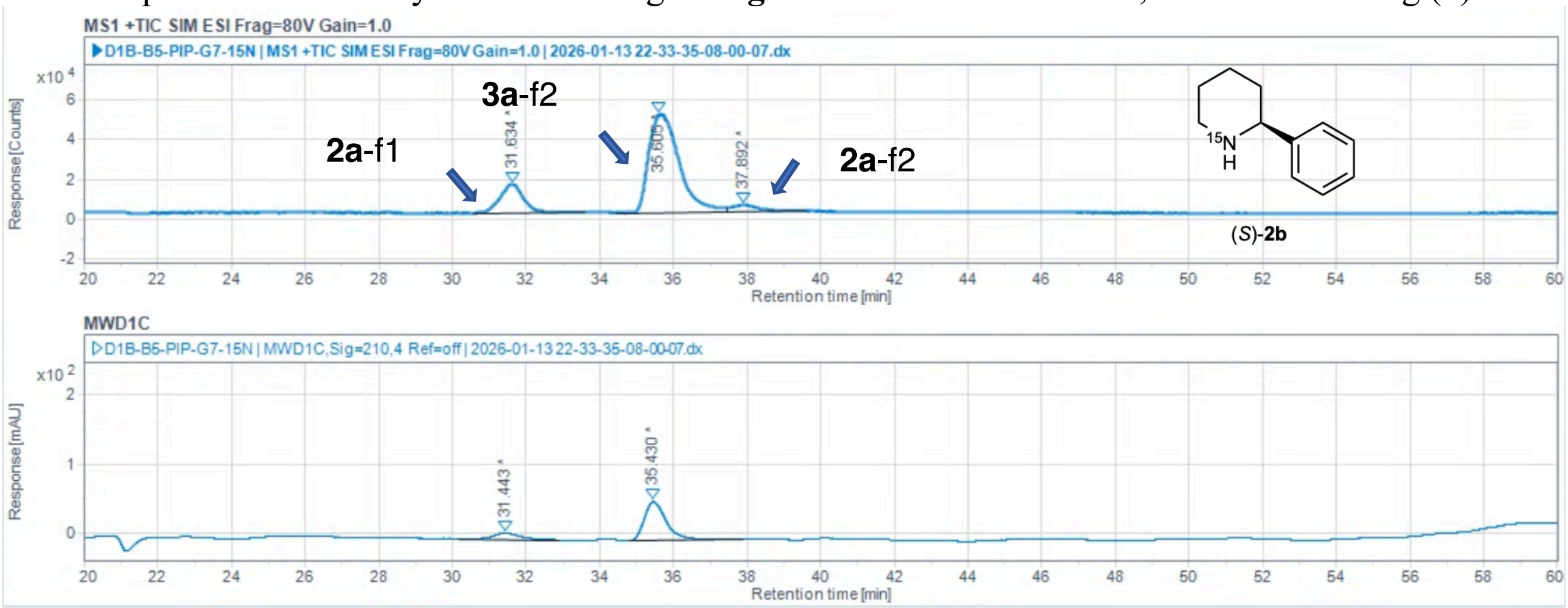

Injection Results

Peaks | Summary

#	Name	Signal description	RT (min)	Area	Area%	Height	Height%	Amou	Concentra	Start time (	End t
2		MS1 +TIC SIM ESI Frag=80V Gain	31.634	634789.751	17.373	14525.135	21.39			30.568	3.600
4		MS1 +TIC SIM ESI Frag=80V Gain	35.605	2834640.698	77.581	49852.346	73.42			34.433	7.434
5		MS1 +TIC SIM ESI Frag=80V Gain	37.892	184360.914	5.046	3521.979	5.19			37.434	9.606
1		MWD1C,Sig=210,4 Ref=off	31.443	425.780	15.435	8.582	13.26			30.152	2.887
3		MWD1C,Sig=210,4 Ref=off	35.430	2332.761	84.565	56.120	86.74			34.789	7.881

2-(4-methoxyphenyl)piperidine (2c)
Chiral separation method: polar ionic HPLC-MS using *dual* Agilent InfinityLab Poroshell 120 Chiral-V, 2.7 µm, 2.1 × 150 mm; eluent: 40% MeOH (0.2 w% NH_4CO_2H), 60% H_2O (20 mM NH_4CO_2H, pH 4.0 adjusted with HCO_2H); flow rate: 0.2 mL/min. t_R = 32.567 min, t_S = 35.979 min.

(*rac*)-**2c** (commercially available standard):

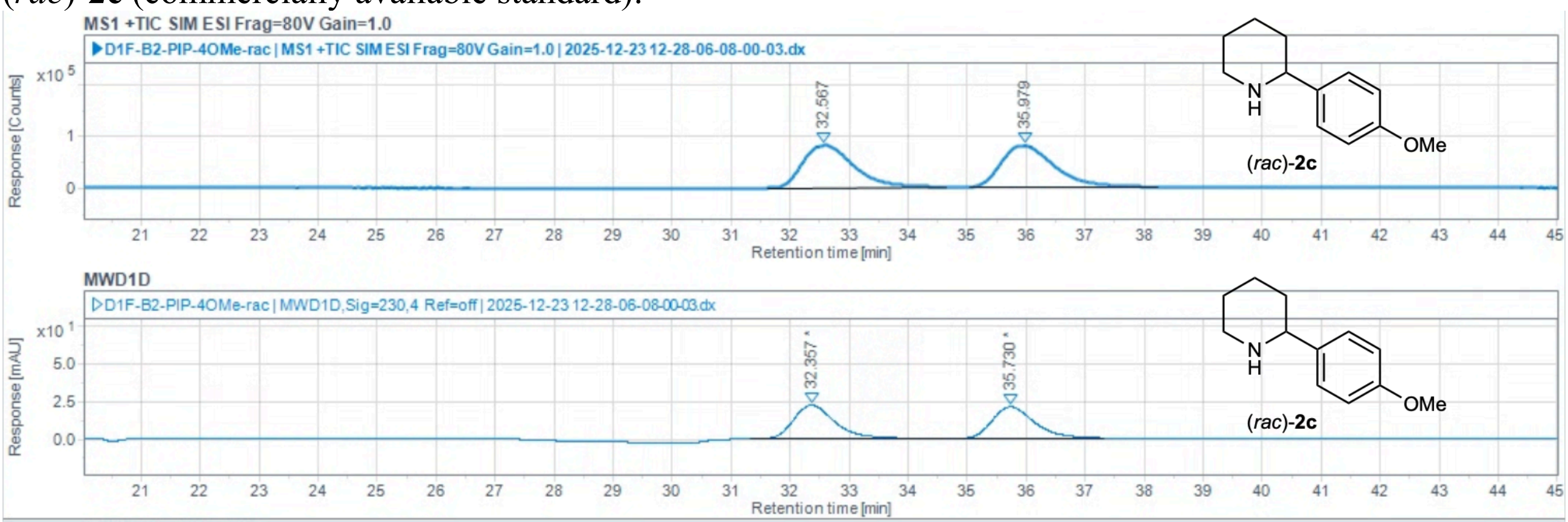

Injection Results

Peaks | Summary

#	Name	Signal description	RT (min)	Area	Area%	Height	Height%	Amou	Concentra	Start time (	End t
2		MS1 +TIC SIM ESI Frag=80V Gain	32.567	4872984.093	49.550	82639.489	50.81			31.612	4.644
4		MS1 +TIC SIM ESI Frag=80V Gain	35.979	4961437.264	50.450	80006.498	49.19			35.045	8.231
1		MWD1D,Sig=230,4 Ref=off	32.357	1073.228	51.465	22.122	51.71			31.314	4.432
3		MWD1D,Sig=230,4 Ref=off	35.730	1012.123	48.535	20.659	48.29			34.432	7.303

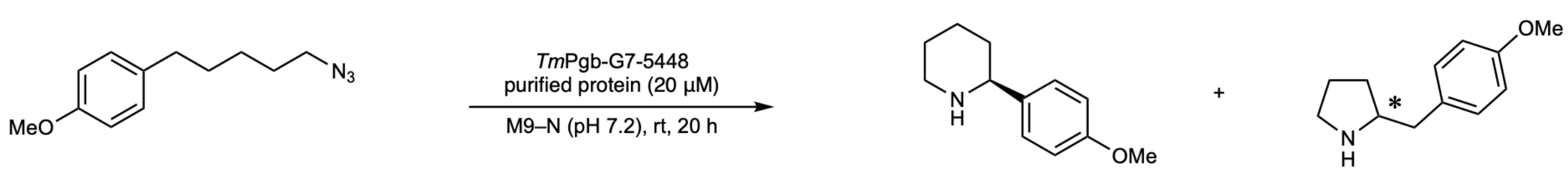

1c **2c** **3c**

Reaction performed on analytical scale using ***Tm*Pgb-PIP-G7-5448**: 84:16 rr, >99% ee favoring (*S*)-**2c**.

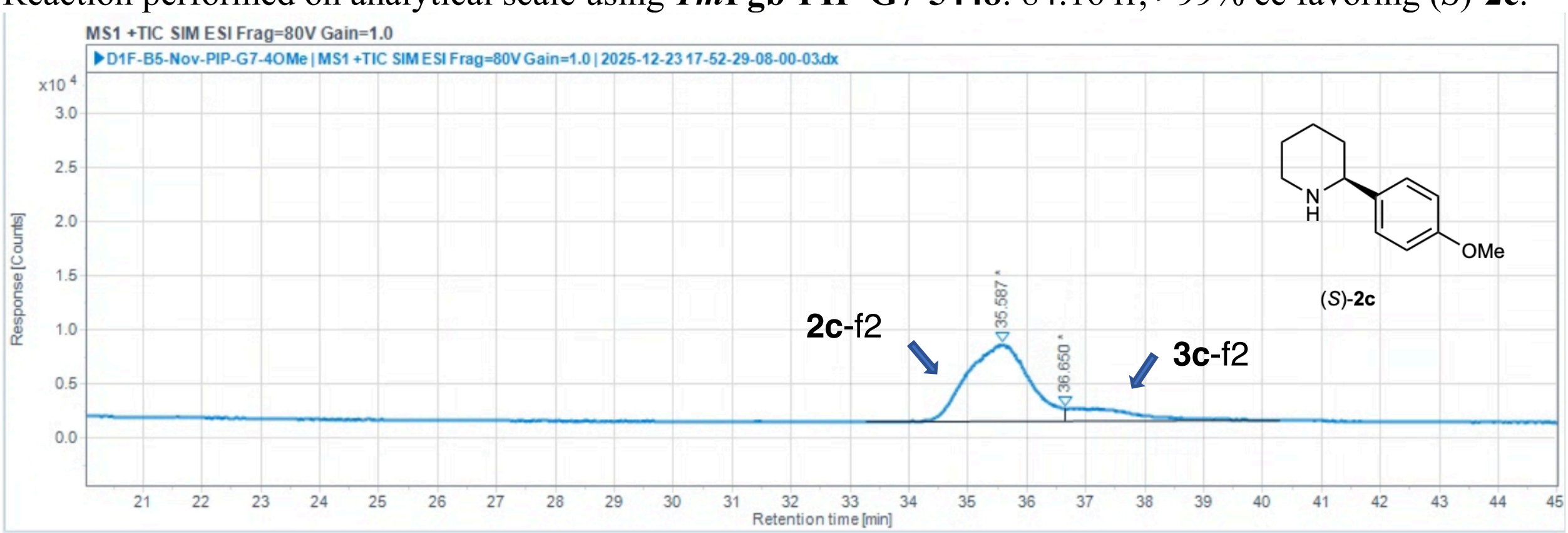

Injection Results

Peaks | Summary

#	Name	Signal description	RT (min)	Area (Counts·s)	Area%	Height (Counts)	Height%	Amou	Concentra	Start time (	End t
1		MS1 +TIC SIM ESI Frag=80V Gain	35.587	512009.489	84.336	7140.513	86.14			33.275	6.634
2		MS1 +TIC SIM ESI Frag=80V Gain	36.650	95097.175	15.664	1149.014	13.86			36.634	0.277

co-injection of (*rac*)-**2c** and (*rac*)-**3c**

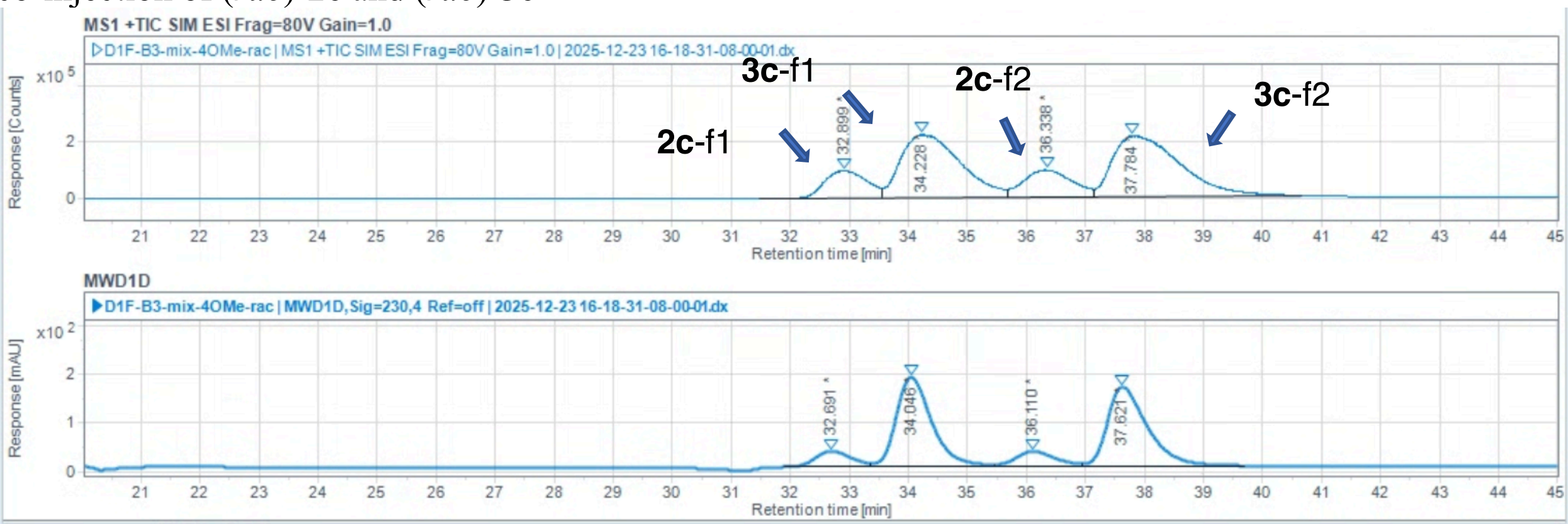

Injection Results

Peaks | Summary

#	Name	Signal description	RT (min)	Area	Area%	Height	Height%	Amou	Concentra	Start time (	End t
2		MS1 +TIC SIM ESI Frag=80V Gain	32.899	4783823.291	11.124	97694.623	15.44			31.461	3.565
4		MS1 +TIC SIM ESI Frag=80V Gain	34.228	15656170.914	36.406	223658.349	35.36			33.565	5.669
6		MS1 +TIC SIM ESI Frag=80V Gain	36.338	5413053.959	12.587	95594.015	15.11			35.669	7.129
8		MS1 +TIC SIM ESI Frag=80V Gain	37.784	17151217.736	39.883	215641.185	34.09			37.129	0.669
1		MWD1D,Sig=230,4 Ref=off	32.691	1356.519	7.489	32.291	7.89			31.882	3.343
3		MWD1D,Sig=230,4 Ref=off	34.046	7714.704	42.593	183.687	44.87			33.343	5.446
5		MWD1D,Sig=230,4 Ref=off	36.110	1431.716	7.905	30.493	7.45			35.446	6.956
7		MWD1D,Sig=230,4 Ref=off	37.621	7609.647	42.013	162.936	39.80			36.956	9.679

2-(p-tolyl)piperidine (2d)

Chiral separation method: polar ionic HPLC-MS using *dual* Agilent InfinityLab Poroshell 120 Chiral-V, 2.7 µm, 2.1 × 150 mm; eluent: 40% MeOH (0.2 w% NH_4CO_2H), 60% H_2O (20 mM NH_4CO_2H, pH 4.0 adjusted with HCO_2H); flow rate: 0.2 mL/min. t_R = 30.346 min, t_S = 36.371 min.

(*rac*)-**2d** (commercially available standard):

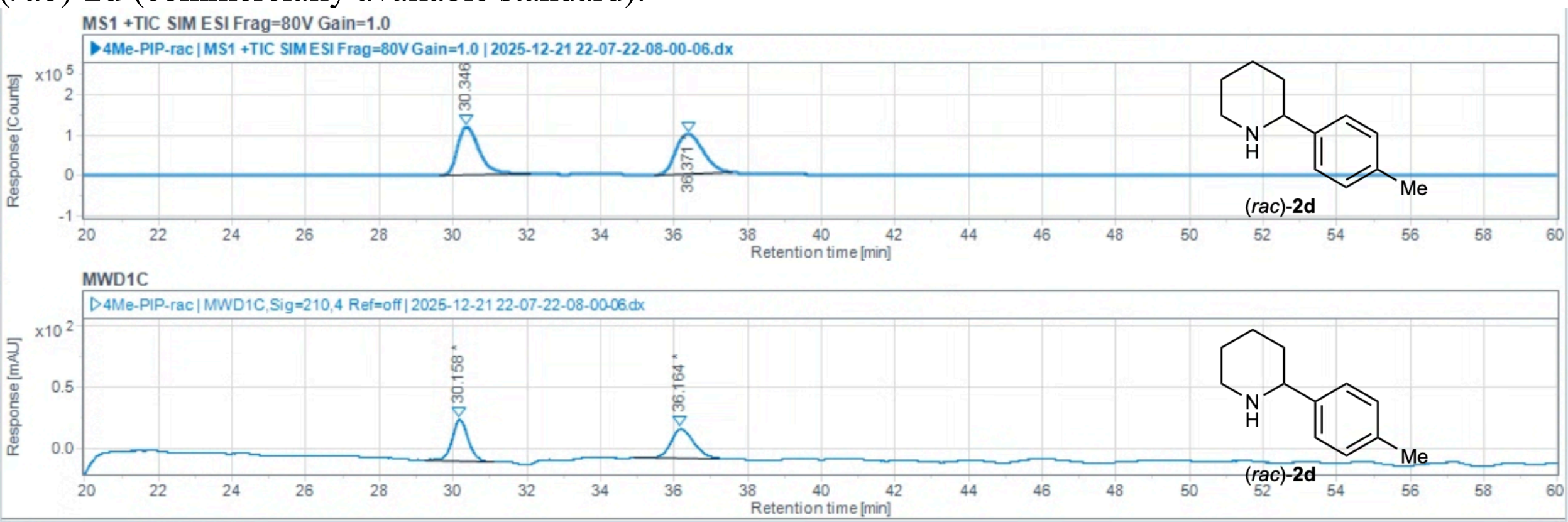

Injection Results

Peaks | Summary

#	Name	Signal description	RT (min)	Area	Area%	Height	Height%	Amou	Concentra	Start time (	End t
2		MS1 +TIC SIM ESI Frag=80V Gain	30.346	4844107.781	48.892	118443.148	54.46			29.653	2.094
4		MS1 +TIC SIM ESI Frag=80V Gain	36.371	5063746.785	51.108	99030.507	45.54			35.493	7.595
1		MWD1C,Sig=210,4 Ref=off	30.158	1000.324	49.030	33.637	58.39			29.227	1.091
3		MWD1C,Sig=210,4 Ref=off	36.164	1039.886	50.970	23.973	41.61			34.938	7.238

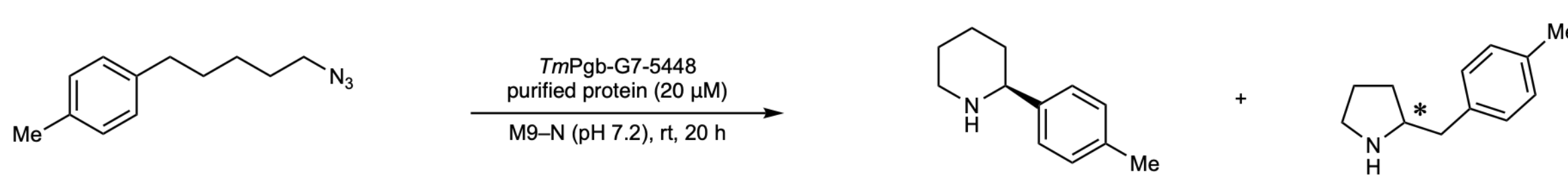

1d **2d** **3d**

Reaction performed on analytical scale using ***Tm*****Pgb-PIP-G7-5448**: >99% ee favoring (*S*)-**2d**.

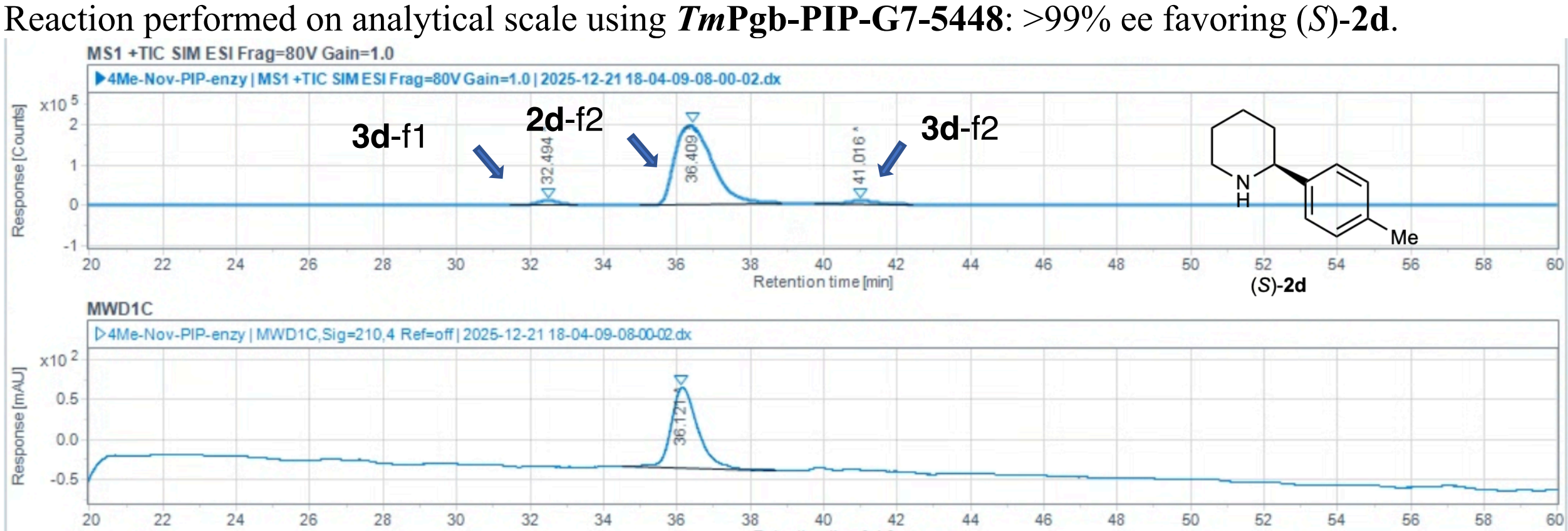

Injection Results

Peaks | Summary

#	Name	Signal description	RT (min)	Area	Area%	Height	Height%	Amou	Concentra	Start time (	End t
1		MS1 +TIC SIM ESI Frag=80V Gain	32.494	445134.209	3.051	10995.479	5.08			31.443	3.270
3		MS1 +TIC SIM ESI Frag=80V Gain	36.409	13736715.937	94.144	196923.774	91.06			35.018	8.831
4		MS1 +TIC SIM ESI Frag=80V Gain	41.016	409372.701	2.806	8336.744	3.86			39.745	2.446
2		MWD1C,Sig=210,4 Ref=off	36.121	5029.515	100.000	100.953	100.00			34.502	8.712

co-injection of (*rac*)-**2d** and (*rac*)-**3d**

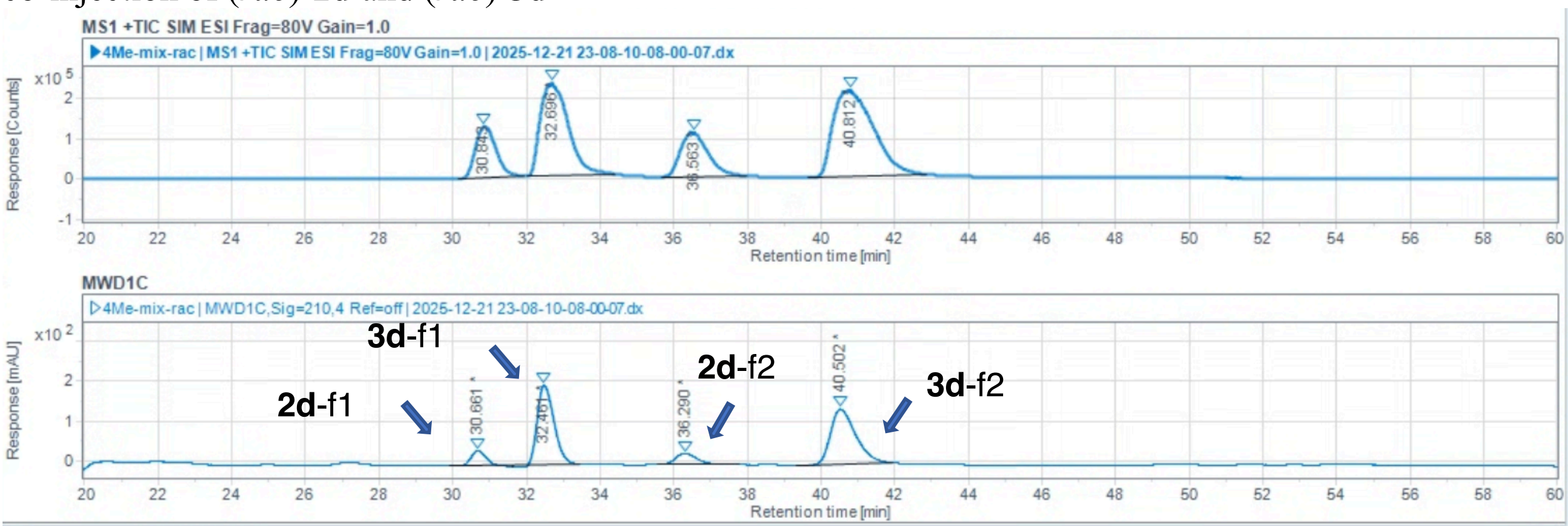

Injection Results

Peaks | Summary

#	Name	Signal description	RT (min)	Area	Area%	Height	Height%	Amou	Concentra	Start time (	End t
2		MS1 +TIC SIM ESI Frag=80V Gain	30.843	4900426.268	12.677	127078.854	18.66			30.168	1.935
4		MS1 +TIC SIM ESI Frag=80V Gain	32.696	12049355.890	31.171	230068.223	33.78			32.014	4.421
6		MS1 +TIC SIM ESI Frag=80V Gain	36.563	5642213.493	14.596	109617.977	16.10			35.691	7.992
8		MS1 +TIC SIM ESI Frag=80V Gain	40.812	16064213.268	41.557	214266.271	31.46			39.658	2.870
1		MWD1C,Sig=210,4 Ref=off	30.661	893.573	6.114	37.203	9.26			29.901	1.606
3		MWD1C,Sig=210,4 Ref=off	32.461	6070.685	41.540	198.902	49.51			31.606	3.431
5		MWD1C,Sig=210,4 Ref=off	36.290	1145.972	7.842	28.398	7.07			35.572	7.794
7		MWD1C,Sig=210,4 Ref=off	40.502	6503.774	44.504	137.270	34.17			39.340	1.958

2-(4-chlorophenyl)piperidine (2e)
Chiral separation method: polar ionic HPLC-MS using *dual* Agilent InfinityLab Poroshell 120 Chiral-V, 2.7 µm, 2.1 × 150 mm; eluent: 40% MeOH (0.2 w% NH_4CO_2H), 60% H_2O (20 mM NH_4CO_2H, pH 4.0 adjusted with HCO_2H); flow rate: 0.2 mL/min. t_R = 38.233 min, t_S = 41.414 min.

(*rac*)-**2e** (commercially available standard):

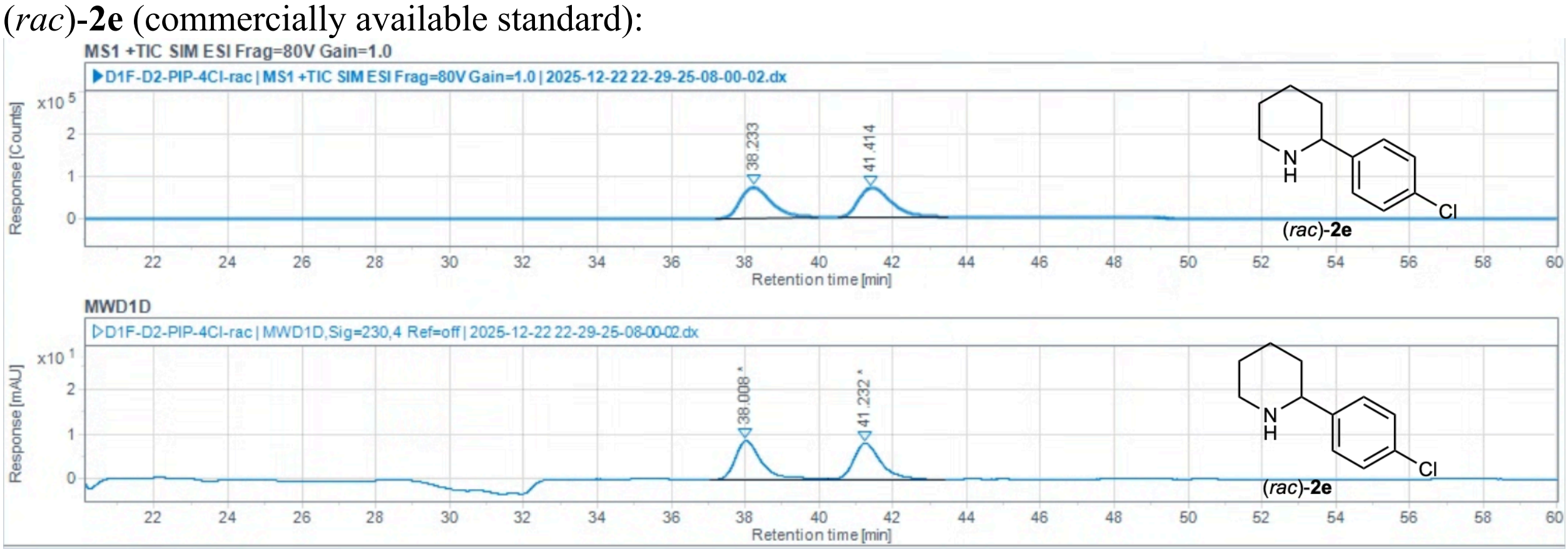

Injection Results

Peaks | Summary

#	Name	Signal description	RT (min)	Area	Area%	Height	Height%	Amou	Concentra	Start time (	End t
2		MS1 +TIC SIM ESI Frag=80V Gain	38.233	4139118.572	49.188	72203.414	50.94			37.225	9.969
4		MS1 +TIC SIM ESI Frag=80V Gain	41.414	4275804.727	50.812	69550.966	49.06			40.520	3.506
1		MWD1D,Sig=230,4 Ref=off	38.008	431.194	51.190	8.651	51.73			37.027	0.230
3		MWD1D,Sig=230,4 Ref=off	41.232	411.153	48.810	8.073	48.27			40.230	3.393

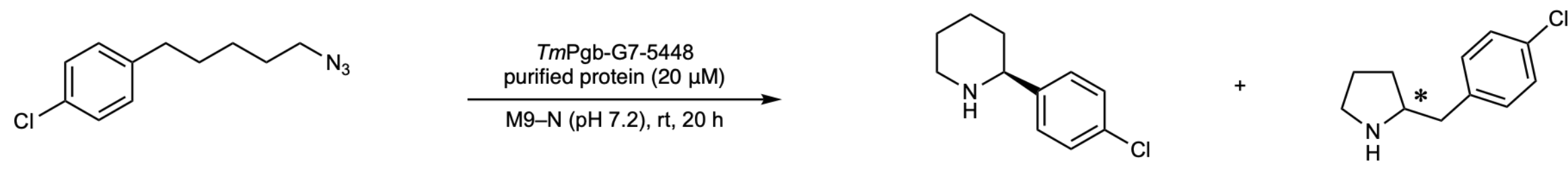

1e **2e** **3e**

Reaction performed on analytical scale using ***Tm*Pgb-PIP-G7-5448**: >99% ee favoring (*S*)-**2e**.

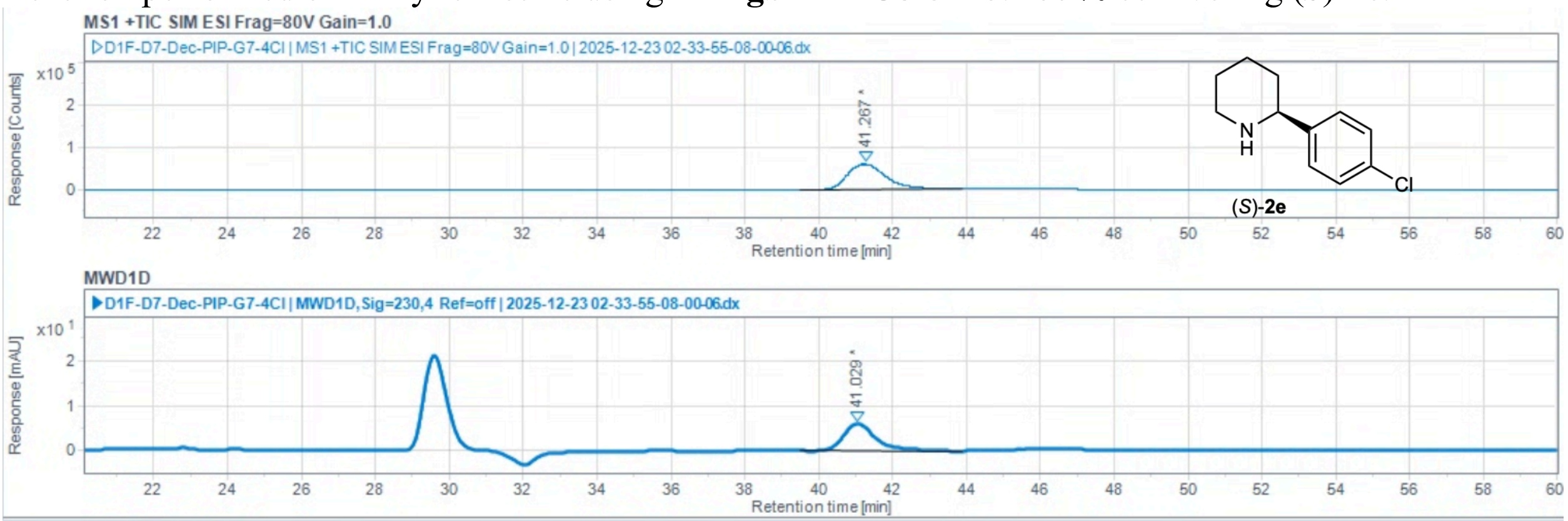

Injection Results

Peaks | Summary

#	Name	Signal description	RT (min)	Area	Area%	Height	Height%	Amou	Concentra	Start time (	End t
2		MS1 +TIC SIM ESI Frag=80V Gain	41.267	4257149.474	100.000	59748.585	100.00			39.479	3.868
1		MWD1D,Sig=230,4 Ref=off	41.029	372.864	100.000	6.018	100.00			39.479	3.868

co-injection of (*rac*)-**2e** and (*rac*)-**3e**

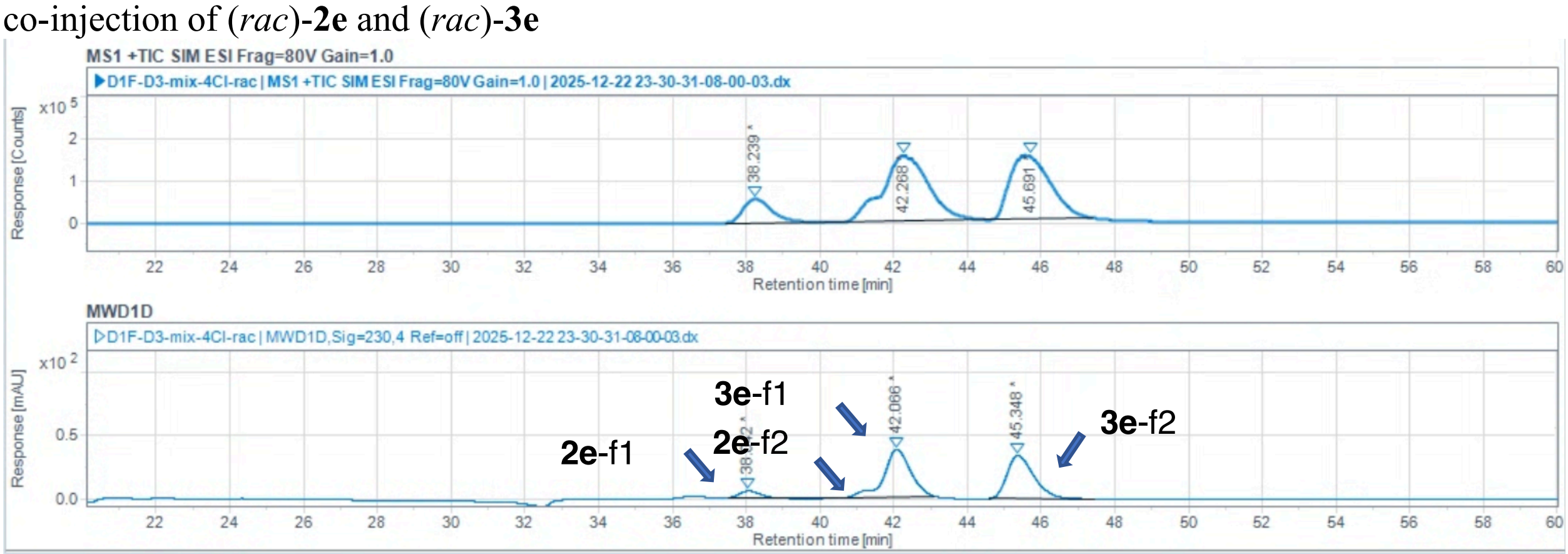

Injection Results

Peaks | Summary

#	Name	Signal description	RT (min)	Area	Area%	Height	Height%	Amou	Concentra	Start time (	End t
2		MS1 +TIC SIM ESI Frag=80V Gain	38.239	3035759.805	10.940	56526.417	15.59			37.423	0.111
4		MS1 +TIC SIM ESI Frag=80V Gain	42.268	13374636.613	48.199	155494.708	42.89			40.111	4.421
6		MS1 +TIC SIM ESI Frag=80V Gain	45.691	11338319.075	40.861	150516.240	41.52			44.421	7.387
1		MWD1D,Sig=230,4 Ref=off	38.042	175.447	4.712	5.433	7.15			37.502	0.507
3		MWD1D,Sig=230,4 Ref=off	42.066	1921.098	51.595	37.354	49.15			40.507	3.117
5		MWD1D,Sig=230,4 Ref=off	45.348	1626.868	43.693	33.210	43.70			44.580	7.427

2-(4-fluorophenyl)piperidine (**2f**)

Chiral separation method: polar ionic HPLC-MS using *dual* Agilent InfinityLab Poroshell 120 Chiral-V, 2.7 µm, 2.1 × 150 mm; eluent: 40% MeOH (0.2 w% NH_4CO_2H), 60% H_2O (20 mM NH_4CO_2H, pH 4.0 adjusted with HCO_2H); flow rate: 0.2 mL/min. t_R = 27.976 min, t_S = 29.024 min.

(*rac*)-**2f** (commercially available standard):

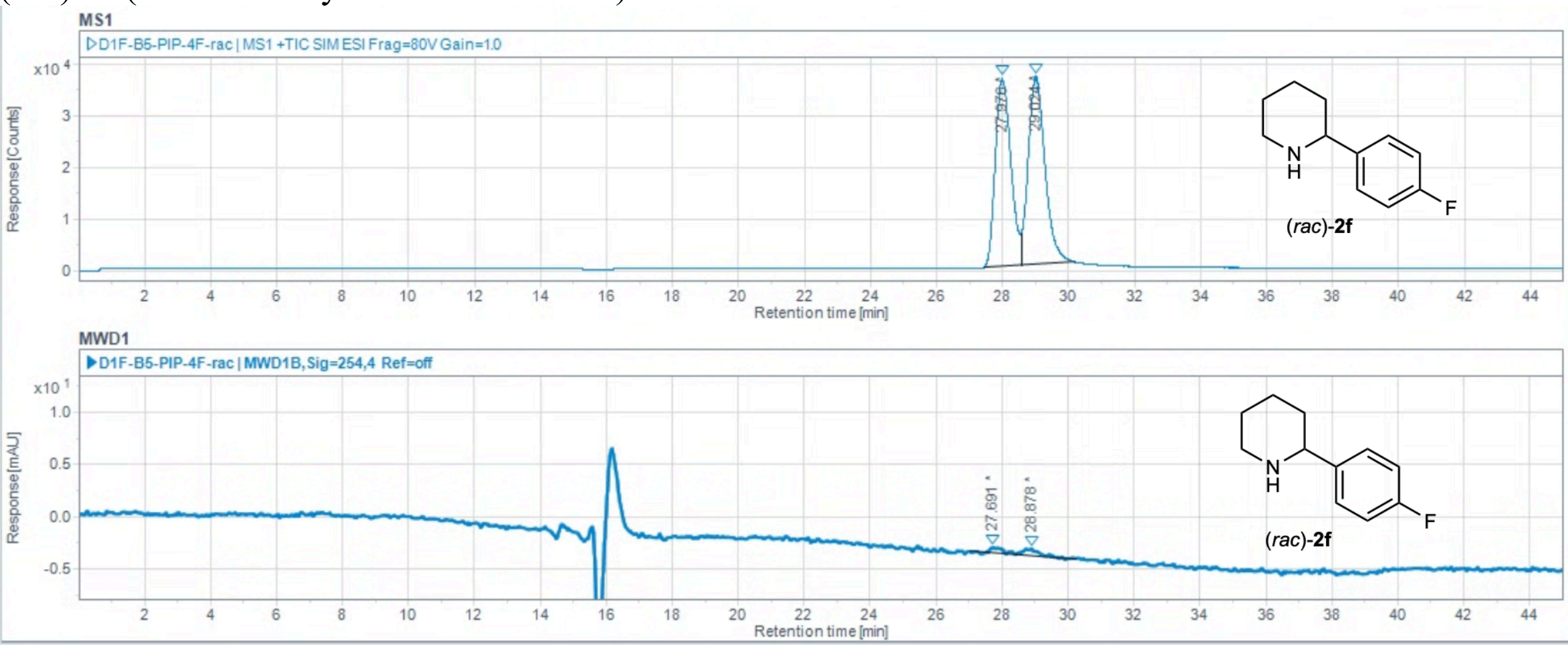

Injection Results

Peaks | Summary

#	Name	Signal description	RT (min)	Area	Area%	Height	Height%	Amou	Concentra	Start time (	End t
2		MS1 +TIC SIM ESI Frag=80V Gain	27.976	1191246.065	47.993	36558.659	50.07			27.446	8.565
4		MS1 +TIC SIM ESI Frag=80V Gain	29.024	1290856.144	52.007	36450.674	49.93			28.565	0.176
1		MWD1B,Sig=254,4 Ref=off	27.691	13.202	41.550	0.592	44.77			27.011	8.410
3		MWD1B,Sig=254,4 Ref=off	28.878	18.572	58.450	0.730	55.23			28.410	0.233

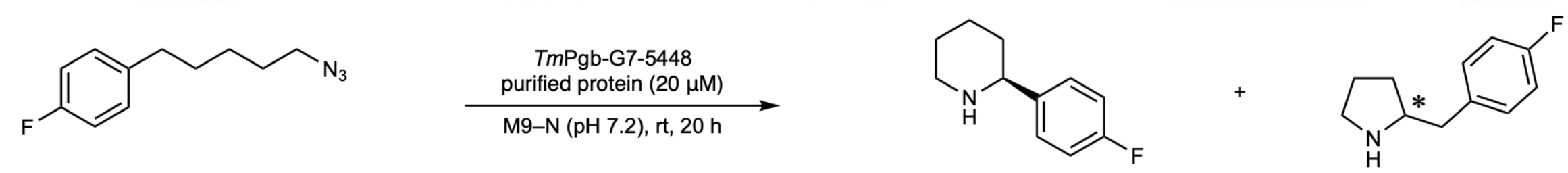

1f **2f** **3f**

Reaction performed on analytical scale using ***Tm*Pgb-PIP-G7-5448**: >99% ee favoring (*S*)-**2f**.

By analyzing the following spectrum together with the calculated average regioselectivity based on reverse-phase HPLC-MS using C18 column, the composition of the following spectrum is: major peak (co-elution of 6% **3f**-f1, 84% **2f**-f2) and minor peak (10% **3f**-f2).

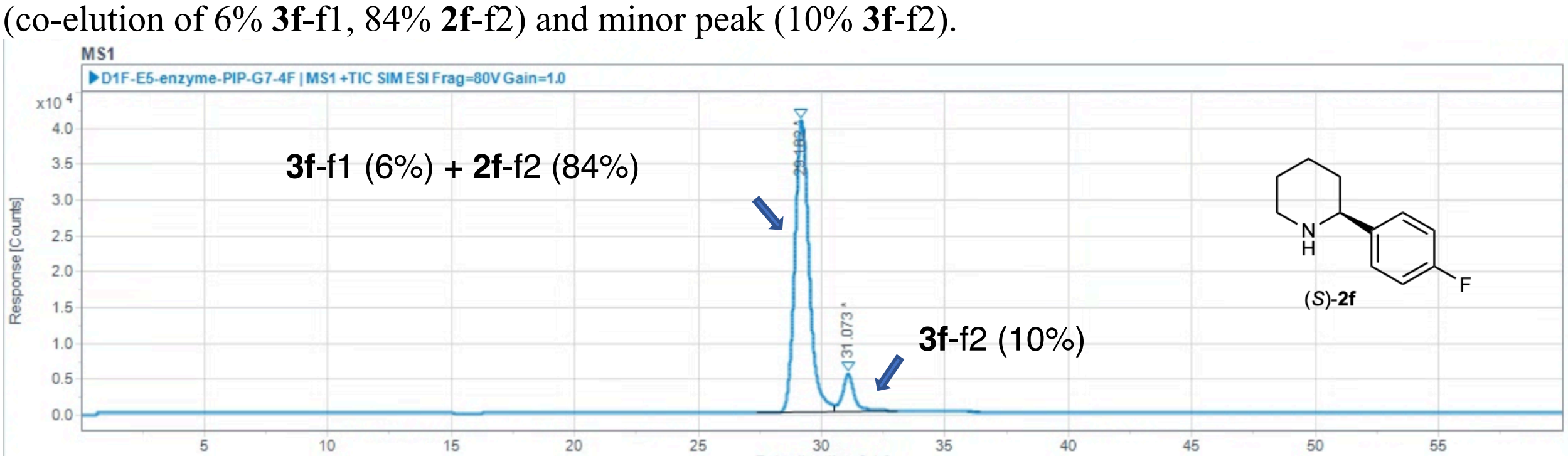

Injection Results

Peaks | Summary

#	Name	Signal description	RT (min)	Area (Counts·s)	Area%	Height (Counts)	Height%	Amou	Concentra	Start time (	End t
1		MS1 +TIC SIM ESI Frag=80V Gain	29.182	1643918.847	90.225	40776.212	88.36			27.430	0.540
2		MS1 +TIC SIM ESI Frag=80V Gain	31.073	178110.789	9.775	5369.829	11.64			30.540	3.084

co-injection of (*rac*)-**2f** and (*rac*)-**3f**

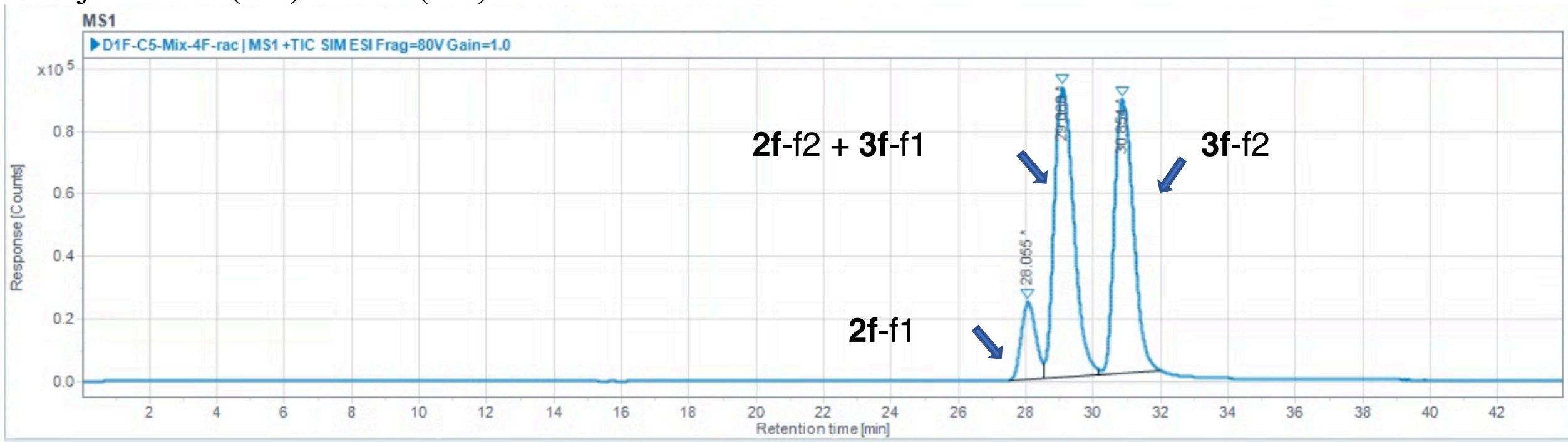

Injection Results

Peaks Summary

#	Name	Signal description	RT (min)	Area (Counts·s)	Area%	Height (Counts)	Height%	Amou	Concentra	Start time (	End t
1		MS1 +TIC SIM ESI Frag=80V Gain	28.055	736548.867	9.378	24882.195	12.11			27.478	8.513
2		MS1 +TIC SIM ESI Frag=80V Gain	29.060	3723211.113	47.407	92884.544	45.19			28.513	0.168
3		MS1 +TIC SIM ESI Frag=80V Gain	30.854	3394038.109	43.215	87776.457	42.70			30.168	1.988

2-(3-fluorophenyl)piperidine (2j)

Chiral separation method: polar ionic HPLC-MS using *dual* Agilent InfinityLab Poroshell 120 Chiral-V, 2.7 µm, 2.1 × 150 mm; eluent: 40% MeOH (0.2 w% NH_4CO_2H), 60% H_2O (20 mM NH_4CO_2H, pH 4.0 adjusted with HCO_2H); flow rate: 0.2 mL/min. t_R = 31.205 min, t_S = 33.295 min.

(*rac*)-**2j** (commercially available standard):

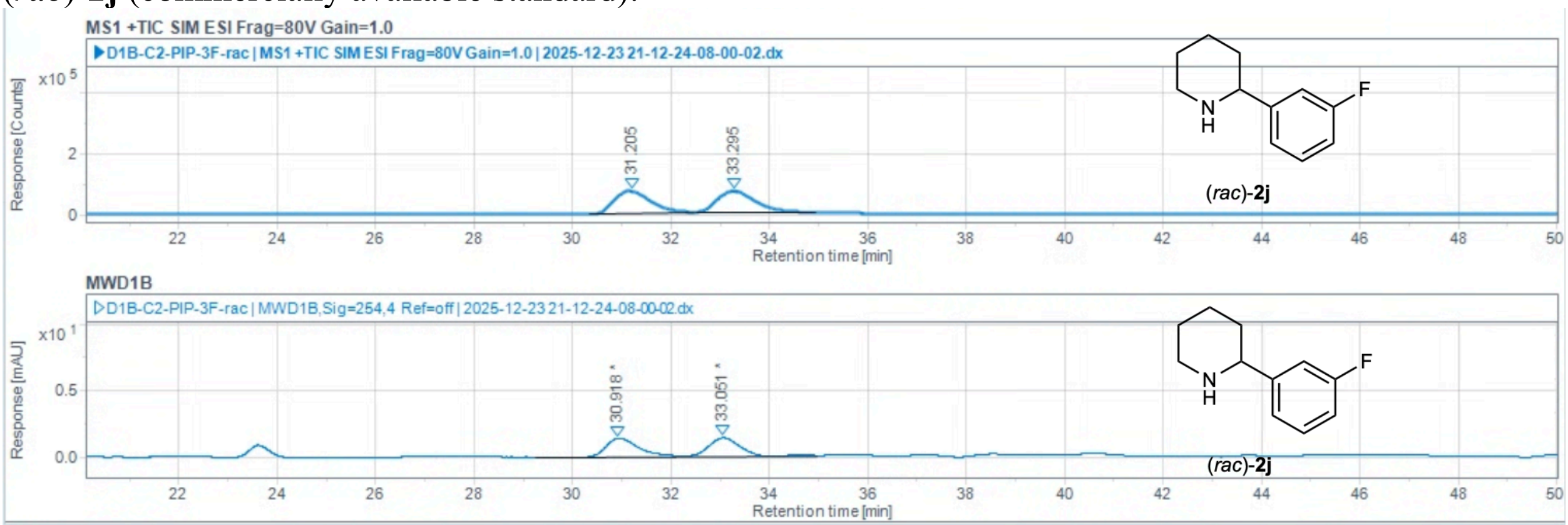

Injection Results

Peaks Summary

#	Name	Signal description	RT (min)	Area	Area%	Height	Height%	Amou	Concentra	Start time (	End t
2		MS1 +TIC SIM ESI Frag=80V Gain	31.205	3730119.493	50.015	73781.663	51.47			30.349	2.492
4		MS1 +TIC SIM ESI Frag=80V Gain	33.295	3727889.147	49.985	69565.262	48.53			32.498	4.934
1		MWD1B,Sig=254,4 Ref=off	30.918	58.558	50.005	1.376	50.07			29.262	2.175
3		MWD1B,Sig=254,4 Ref=off	33.051	58.546	49.995	1.373	49.93			32.175	4.970

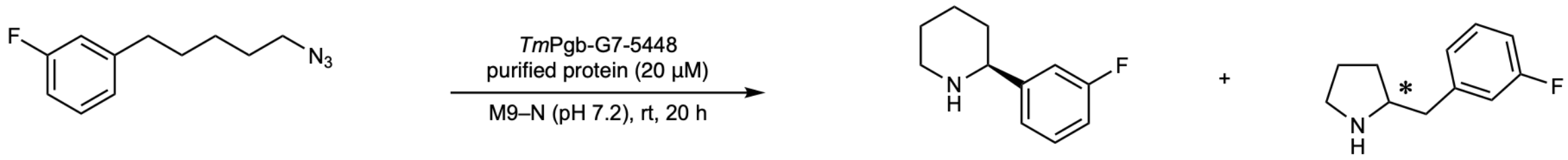

1j **2j** **3j**

Reaction performed on analytical scale using ***Tm*****Pgb-PIP-G7-5448**: >99% ee favoring (*S*)-**2j**.

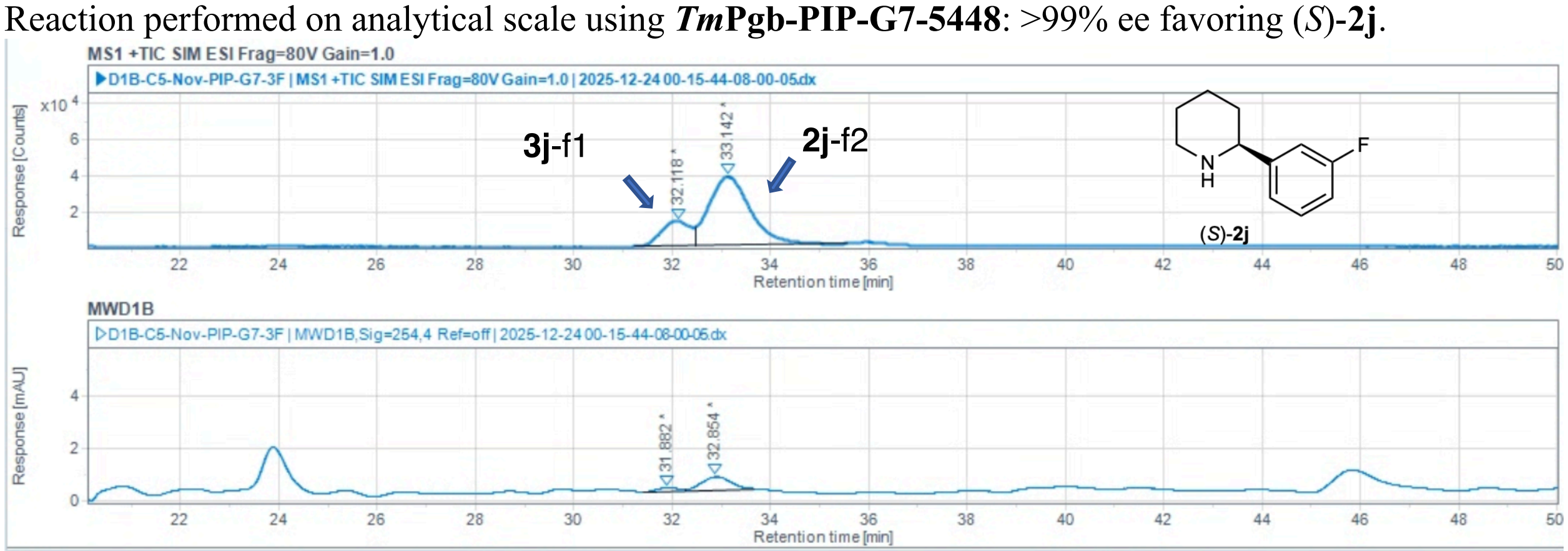

Injection Results

Peaks Summary

#	Name	Signal description	RT (min)	Area	Area%	Height	Height%	Amou	Concentra	Start time (	End t
2		MS1 +TIC SIM ESI Frag=80V Gain	32.118	559072.859	20.184	13689.162	26.63			31.220	2.465
4		MS1 +TIC SIM ESI Frag=80V Gain	33.142	2210741.958	79.816	37707.826	73.37			32.465	5.548
1		MWD1B,Sig=254,4 Ref=off	31.882	4.201	16.747	0.142	22.07			31.405	2.258
3		MWD1B,Sig=254,4 Ref=off	32.854	20.884	83.253	0.501	77.93			32.258	3.663

co-injection of (*rac*)-**2j** and (*rac*)-**3j**

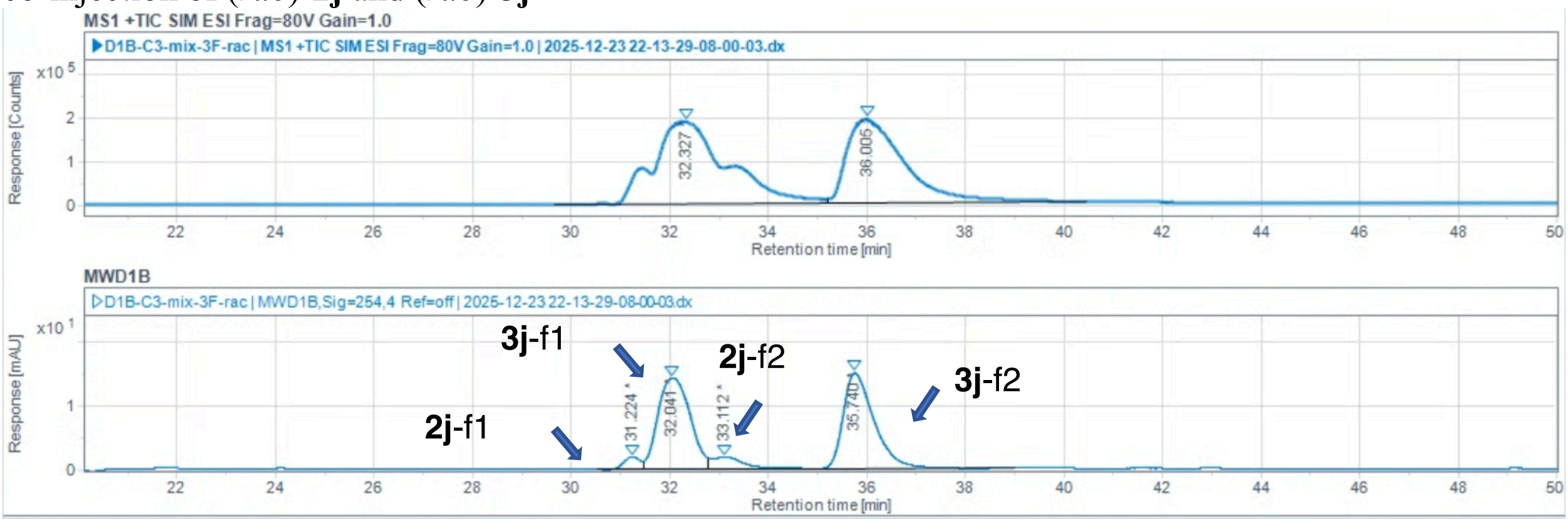

Injection Results

Peaks | Summary

#	Name	Signal description	RT (min)	Area	Area%	Height	Height%	Amou	Concentra	Start time (	End t
3		MS1 +TIC SIM ESI Frag=80V Gain	32.327	19773204.123	55.941	191414.825	49.52			29.675	5.206
6		MS1 +TIC SIM ESI Frag=80V Gain	36.005	15573625.495	44.059	195126.221	50.48			35.206	0.439
1		MWD1B,Sig=254,4 Ref=off	31.224	44.021	3.101	1.943	5.89			30.510	1.461
2		MWD1B,Sig=254,4 Ref=off	32.041	644.785	45.416	14.237	43.17			31.461	2.787
4		MWD1B,Sig=254,4 Ref=off	33.112	86.319	6.080	1.913	5.80			32.787	4.973
5		MWD1B,Sig=254,4 Ref=off	35.740	644.598	45.403	14.890	45.14			34.973	9.013

2-(naphthalen-2-yl)piperidine (2l)

Chiral separation method: polar ionic HPLC-MS using *dual* Agilent InfinityLab Poroshell 120 Chiral-V, 2.7 µm, 2.1 × 150 mm; eluent: 40% MeOH (0.2 w% NH_4CO_2H), 60% H_2O (20 mM NH_4CO_2H, pH 4.0 adjusted with HCO_2H); flow rate: 0.2 mL/min. t_R = 64.074 min, t_S = 75.475 min.

(*rac*)-**2l** (commercially available standard):

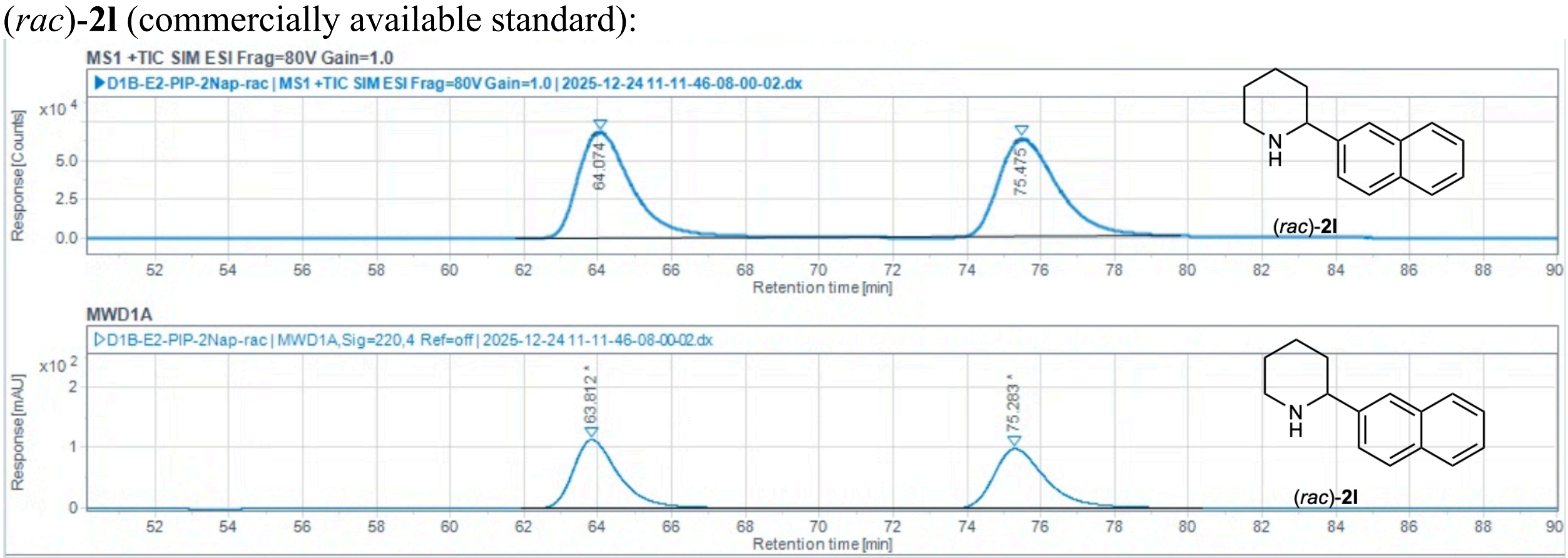

Injection Results

Peaks | Summary

#	Name	Signal description	RT (min)	Area	Area%	Height	Height%	Amou	Concentra	Start time (	End t
2		MS1 +TIC SIM ESI Frag=80V Gain	64.074	6866458.716	50.046	68128.588	51.85			61.766	0.304
4		MS1 +TIC SIM ESI Frag=80V Gain	75.475	6853896.816	49.954	63261.452	48.15			70.304	9.795
1		MWD1A,Sig=220,4 Ref=off	63.812	9781.591	50.474	113.998	53.60			61.925	9.470
3		MWD1A,Sig=220,4 Ref=off	75.283	9598.050	49.526	98.666	46.40			69.470	0.391

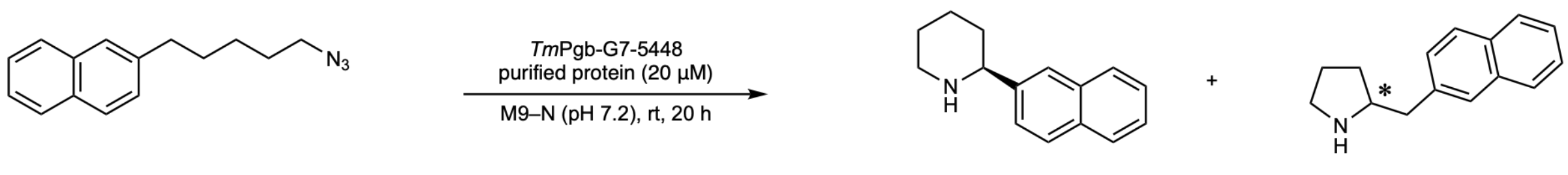

1l **2l** **3l**

Reaction performed on analytical scale using ***Tm*Pgb-PIP-G7-5448**: >99% ee favoring (*S*)-**2l**.

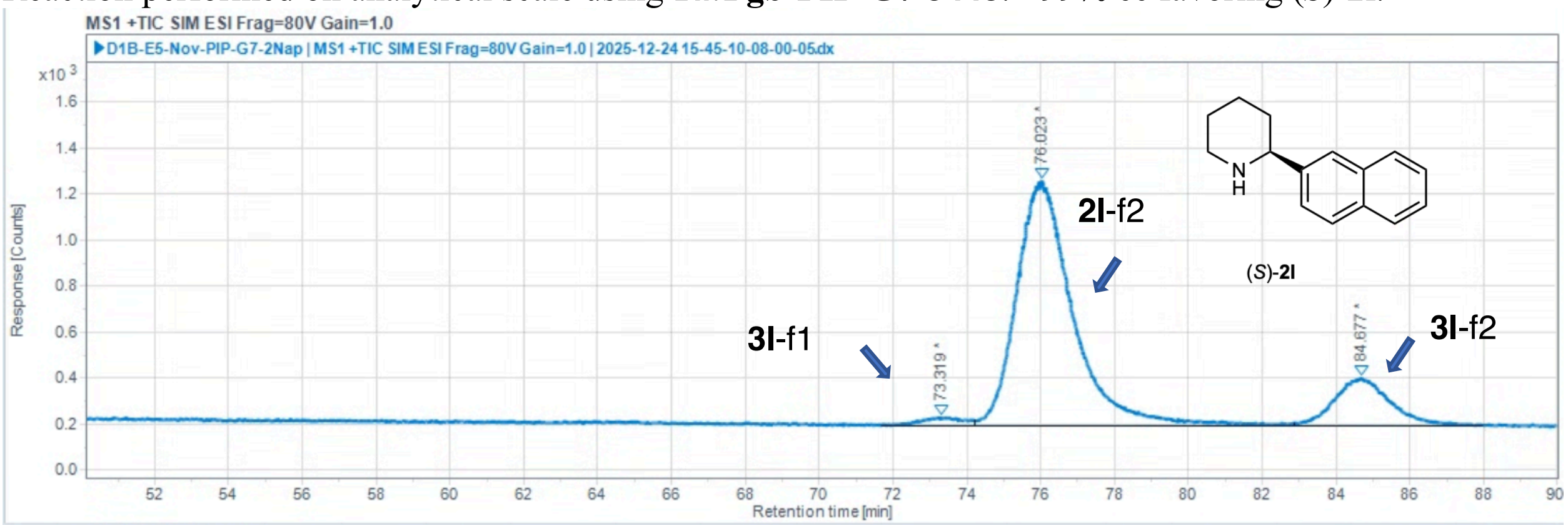

Injection Results

Peaks Summary

#	Name	Signal description	RT (min)	Area (Counts·s)	Area%	Height (Counts)	Height%	Amou	Concentra	Start time (	End t
1		MS1 +TIC SIM ESI Frag=80V Gain	73.319	2721.216	2.015	35.363	2.70			71.666	4.195
2		MS1 +TIC SIM ESI Frag=80V Gain	76.023	111538.137	82.580	1067.800	81.67			74.195	2.849
3		MS1 +TIC SIM ESI Frag=80V Gain	84.677	20807.920	15.406	204.256	15.62			82.849	7.986

co-injection of (*rac*)-**2l** and (*rac*)-**3l**

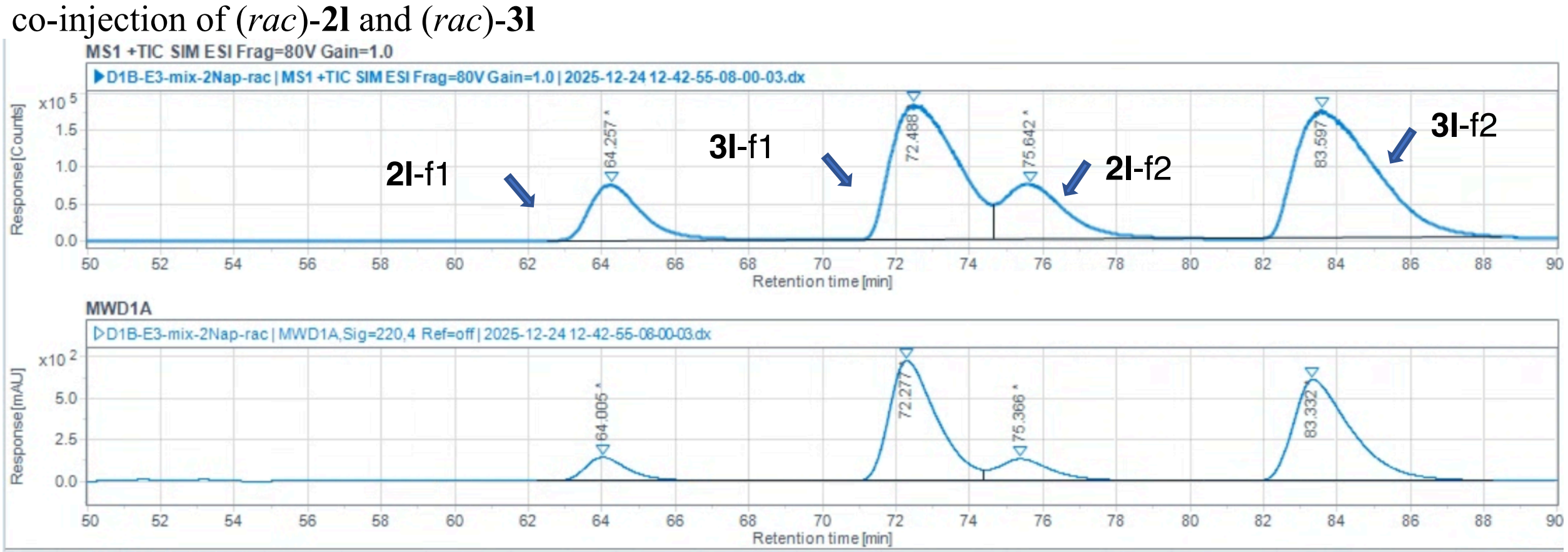

Injection Results

Peaks Summary

#	Name	Signal description	RT (min)	Area	Area%	Height	Height%	Amou	Concentra	Start time (	End t
2		MS1 +TIC SIM ESI Frag=80V Gain	64.257	7429091.587	11.580	76304.937	15.01			62.521	8.795
4		MS1 +TIC SIM ESI Frag=80V Gain	72.488	22467663.451	35.021	184149.241	36.22			68.795	4.641
6		MS1 +TIC SIM ESI Frag=80V Gain	75.642	8744396.594	13.630	75069.078	14.76			74.641	9.994
8		MS1 +TIC SIM ESI Frag=80V Gain	83.597	25513239.464	39.769	172923.615	34.01			79.994	8.492
1		MWD1A,Sig=220,4 Ref=off	64.005	11920.408	7.586	142.924	8.90			62.243	8.716
3		MWD1A,Sig=220,4 Ref=off	72.277	65518.931	41.697	724.926	45.15			68.716	4.394
5		MWD1A,Sig=220,4 Ref=off	75.366	13444.843	8.556	130.755	8.14			74.394	9.954
7		MWD1A,Sig=220,4 Ref=off	83.332	66246.218	42.160	607.128	37.81			79.954	8.254

Part 4: Regio- and enantioselectivity analysis of substrate scope study on reactions performed with **GDM-NT-401**.
Results analyzed on polar ionic chiral HPLC-MS, normal phase HPLC, or chiral GC.

2-phenylpiperidine (**2a** and **2b**)
Chiral separation method: polar ionic HPLC-MS using *dual* Agilent InfinityLab Poroshell 120 Chiral-V, 2.7 µm, 2.1 × 150 mm; eluent: 40% MeOH (0.2 w% NH_4CO_2H), 60% H_2O (20 mM NH_4CO_2H, pH 4.0 adjusted with HCO_2H); flow rate: 0.2 mL/min. t_R = 33.270 min, t_S = 35.262 min.

(*rac*)-**2a** (commercially available standard):

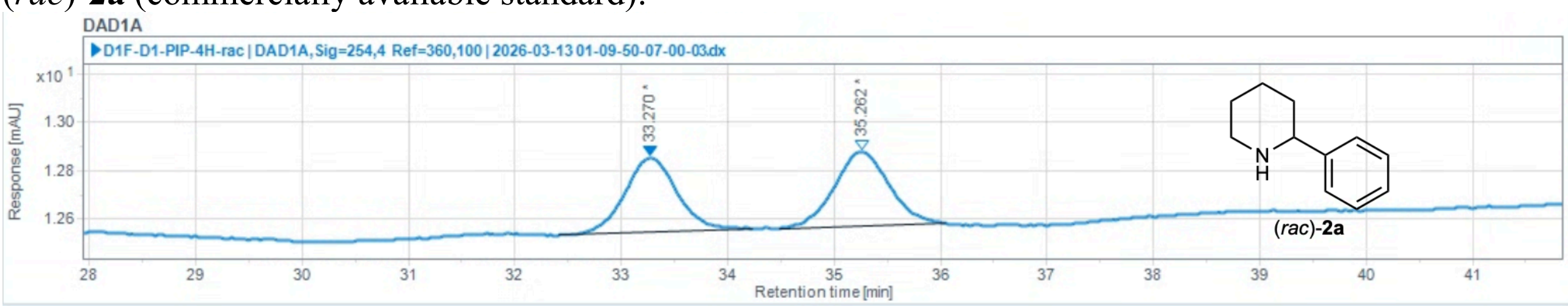

Injection Results

Peaks | Summary

#	Name	Signal description	RT (min)	Area (mAU·s)	Area%	Height (mAU)	Height%	Amount	Concentration	Start tim
1		DAD1A,Sig=254,4 Ref=360,100	33.270	10.460	49.012	0.307	50.25			
2		DAD1A,Sig=254,4 Ref=360,100	35.262	10.881	50.988	0.304	49.75			

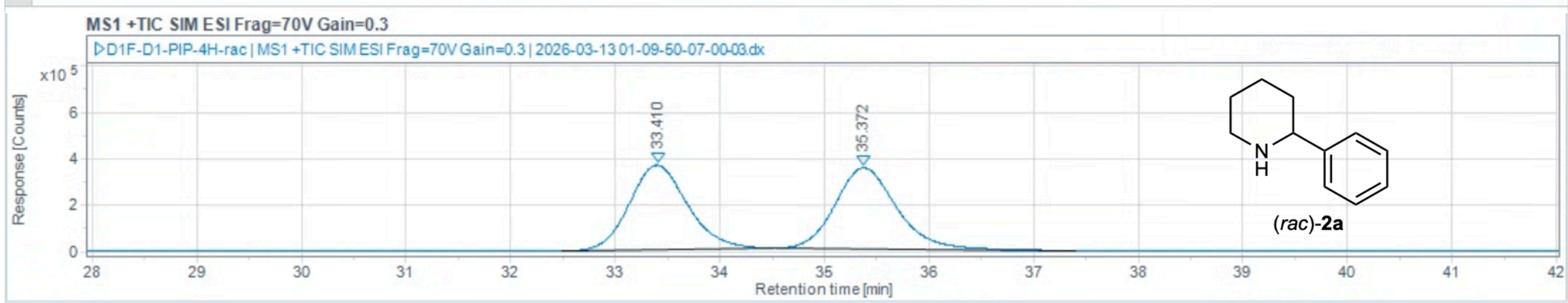

Injection Results

Peaks | Summary

#	Name	Signal description	RT (min)	Area (Counts·s)	Area%	Height (Counts)	Height%	Amount	Concentration	Start tim
1		MS1 +TIC SIM ESI Frag=70V Gain=0.3	33.410	14206364.978	49.736	365616.260	51.07			
2		MS1 +TIC SIM ESI Frag=70V Gain=0.3	35.372	14356974.653	50.264	350287.991	48.93			

(*S*)-**2a** (commercially available standard):

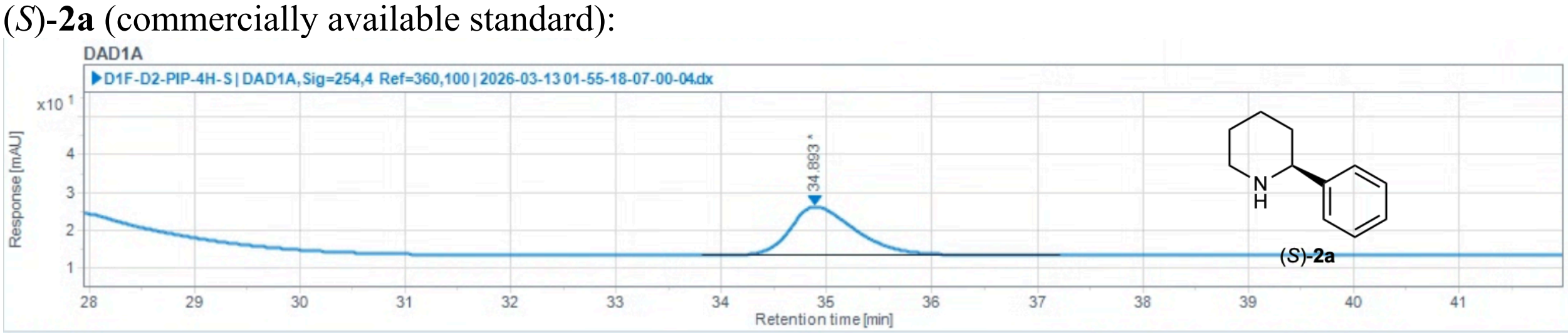

Injection Results

Peaks | Summary

#	Name	Signal description	RT (min)	Area (mAU·s)	Area%	Height (mAU)	Height%	Amount	Concentration	Start tim
1		DAD1A,Sig=254,4 Ref=360,100	34.893	567.528	100.000	12.937	100.00			

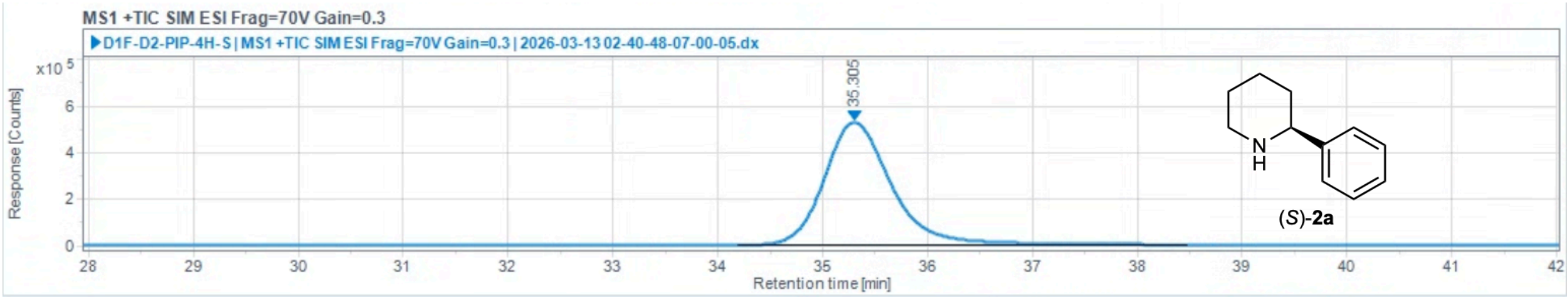

Injection Results

Peaks | Summary

#	Name	Signal description	RT (min)	Area (Counts·s)	Area%	Height (Counts)	Height%	Amount	Concentration	Start tim
1		MS1 +TIC SIM ESI Frag=70V Gain=0.3	35.305	23513403.082	100.000	527373.776	100.00			

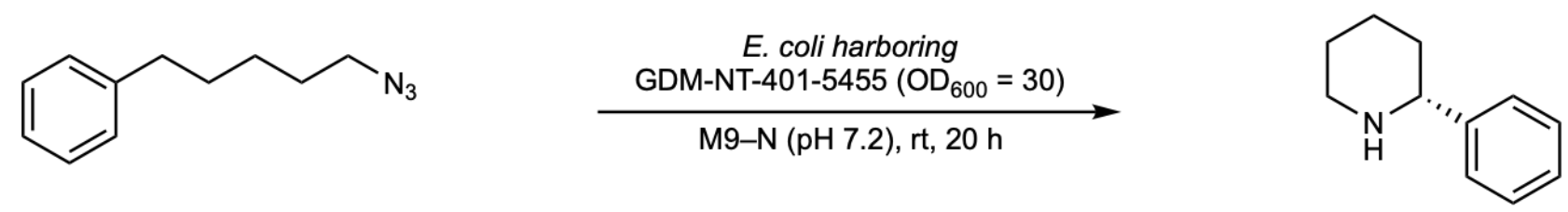

1a **2a**

Reaction performed on analytical scale using **GDM-NT-401-5455**: 95% ee favoring (*R*)-**2a**.

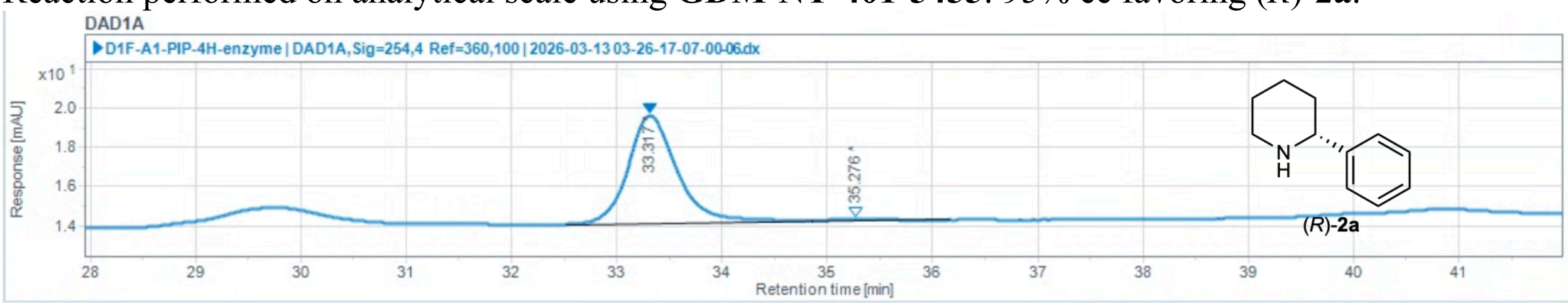

Injection Results

Peaks | Summary

#	Name	Signal description	RT (min)	Area (mAU·s)	Area%	Height (mAU)	Height%	Amount	Concentration	Start tim
1		DAD1A,Sig=254,4 Ref=360,100	33.317	179.212	97.929	5.481	98.32			
2		DAD1A,Sig=254,4 Ref=360,100	35.276	3.790	2.071	0.094	1.68			

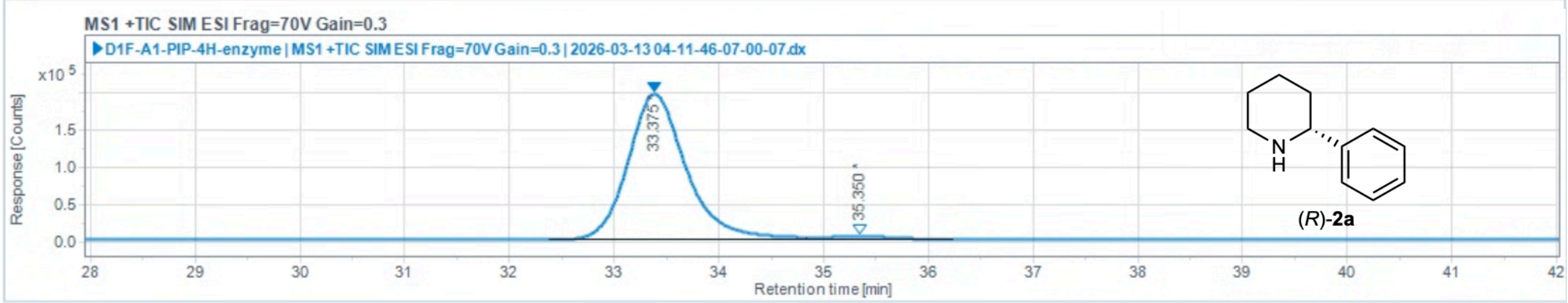

Injection Results

Peaks | Summary

#	Name	Signal description	RT (min)	Area (Counts·s)	Area%	Height (Counts)	Height%	Amount	Concentration	Start tim
1		MS1 +TIC SIM ESI Frag=70V Gain=0.3	33.375	7585259.311	97.548	194831.248	97.90			
2		MS1 +TIC SIM ESI Frag=70V Gain=0.3	35.350	190639.788	2.452	4182.668	2.10			

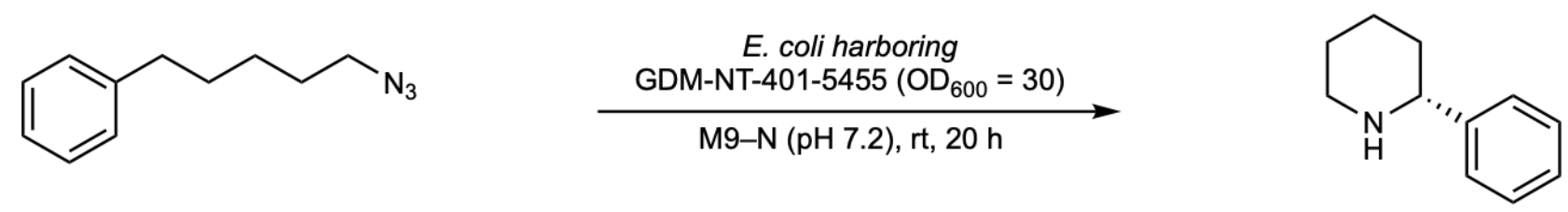

1a **2a**

Reaction performed on 1.0 mmol scale using **GDM-NT-401-5455**: 95% ee favoring (*R*)-**2a**.

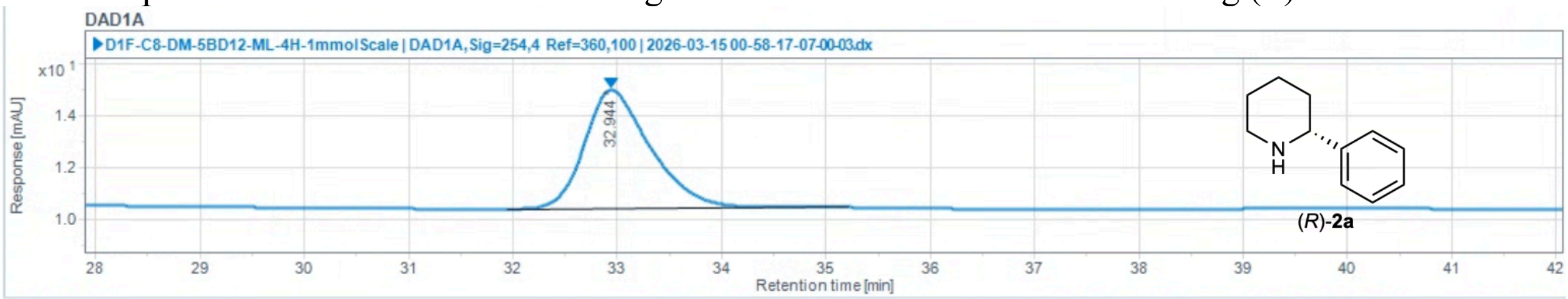

Injection Results

Peaks Summary

#	Name	Signal description	RT (min)	Area (mAU·s)	Area%	Height (mAU)	Height%	Amount	Concentration	Start tim
1		DAD1A,Sig=254,4 Ref=360,100	32.944	201.221	100.000	4.544	100.00			

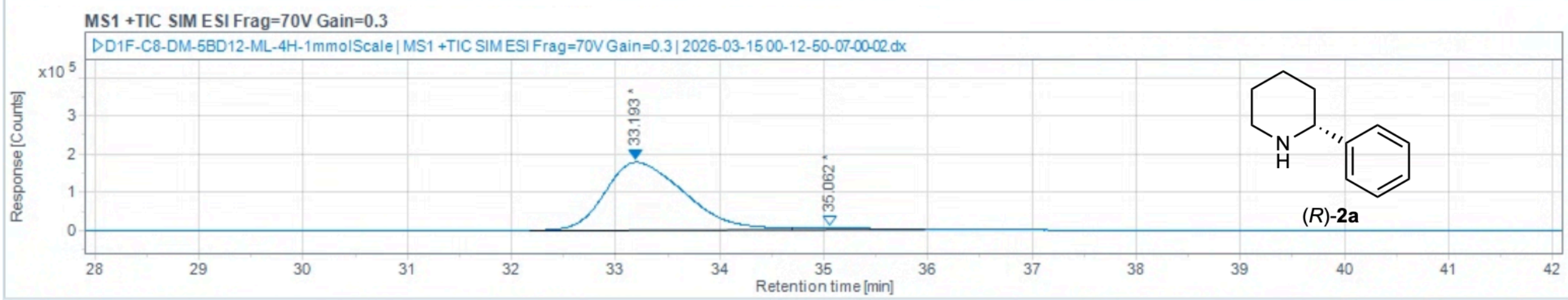

Injection Results

Peaks Summary

#	Name	Signal description	RT (min)	Area (Counts·s)	Area%	Height (Counts)	Height%	Amount	Concentration	Start tim
1		MS1 +TIC SIM ESI Frag=70V Gain=0.3	33.193	9410385.178	97.643	177249.125	97.51			
2		MS1 +TIC SIM ESI Frag=70V Gain=0.3	35.062	227126.251	2.357	4525.898	2.49			

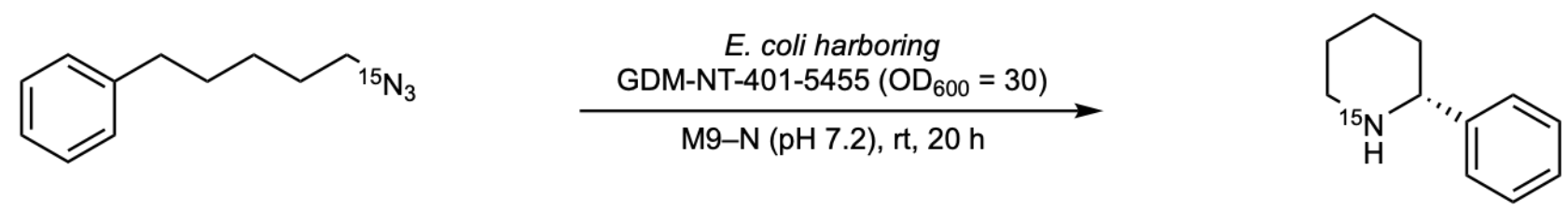

1b **2b**

Reaction performed on analytical scale using **GDM-NT-401-5455**: 95% ee favoring (*R*)-**2b**.

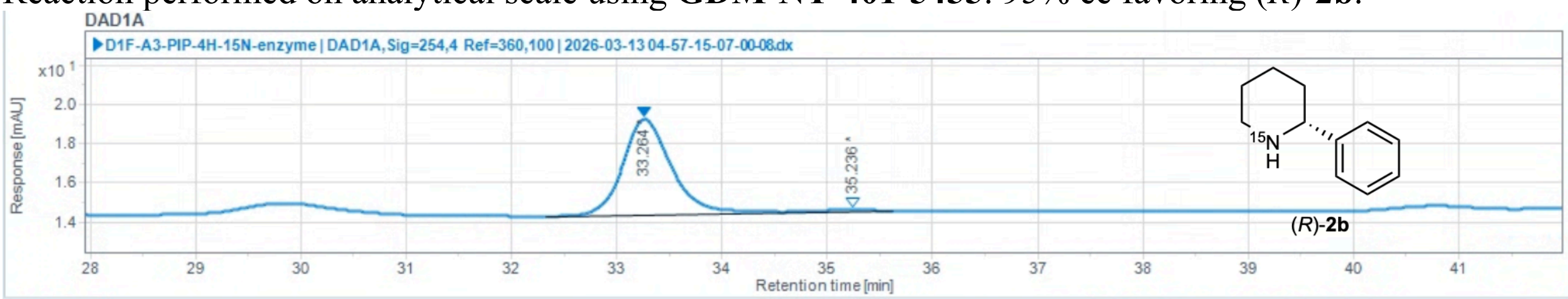

Injection Results

Peaks | Summary

#	Name	Signal description	RT (min)	Area (mAU·s)	Area%	Height (mAU)	Height%	Amount	Concentration	Start tim
1		DAD1A,Sig=254,4 Ref=360,100	33.264	157.519	97.708	4.878	98.17			
2		DAD1A,Sig=254,4 Ref=360,100	35.236	3.695	2.292	0.091	1.83			

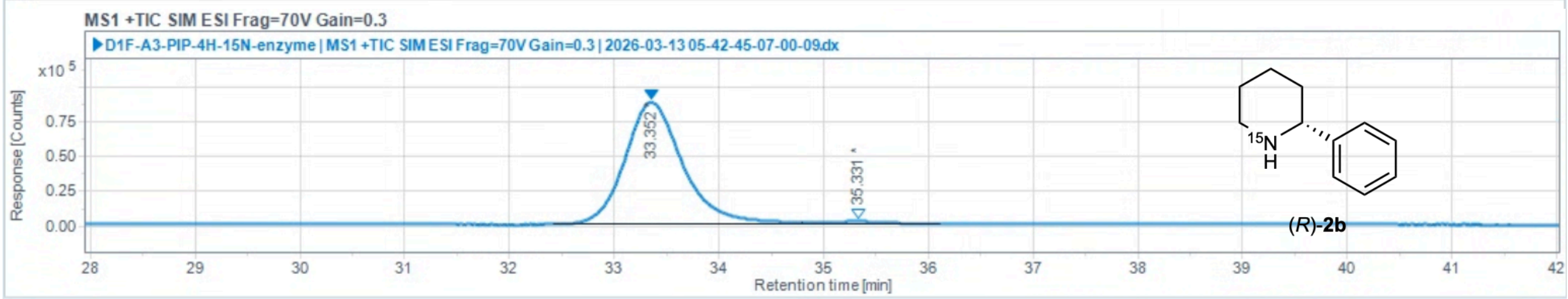

Injection Results

Peaks | Summary

#	Name	Signal description	RT (min)	Area (Counts·s)	Area%	Height (Counts)	Height%	Amount	Concentration	Start tim
1		MS1 +TIC SIM ESI Frag=70V Gain=0.3	33.352	3398754.648	97.660	87738.341	97.96			
2		MS1 +TIC SIM ESI Frag=70V Gain=0.3	35.331	81450.521	2.340	1829.352	2.04			

2-(4-methoxyphenyl)piperidine (2c)
Chiral separation method: polar ionic HPLC-MS using *dual* Agilent InfinityLab Poroshell 120 Chiral-V, 2.7 µm, 2.1 × 150 mm; eluent: 40% MeOH (0.2 w% NH_4CO_2H), 60% H_2O (20 mM NH_4CO_2H, pH 4.0 adjusted with HCO_2H); flow rate: 0.2 mL/min. t_R = 33.460 min, t_S = 36.557 min.

(*rac*)-**2c** (commercially available standard):

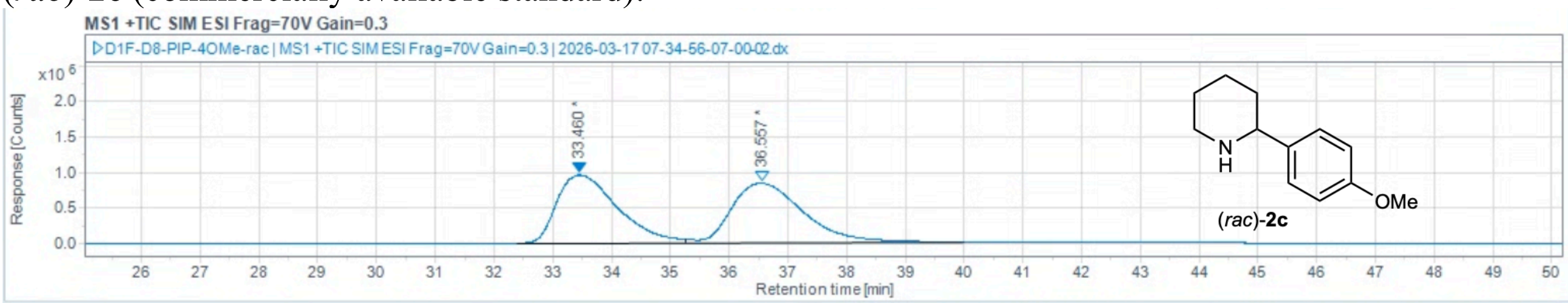

Injection Results

Peaks Summary

#	Name	Signal description	RT (min)	Area (Counts·s)	Area%	Height (Counts)	Height%	Amount	Concentration	Start tim
1		MS1 +TIC SIM ESI Frag=70V Gain=0.3	33.460	68734223.408	49.186	956143.898	53.39			
2		MS1 +TIC SIM ESI Frag=70V Gain=0.3	36.557	71008360.767	50.814	834764.032	46.61			

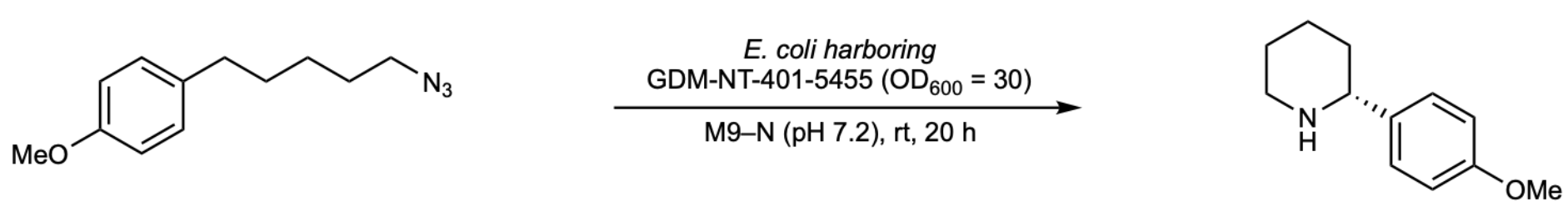

1c **2c**

Reaction performed on analytical scale using **GDM-NT-401-5455**: 90% ee favoring (*R*)-**2c**.

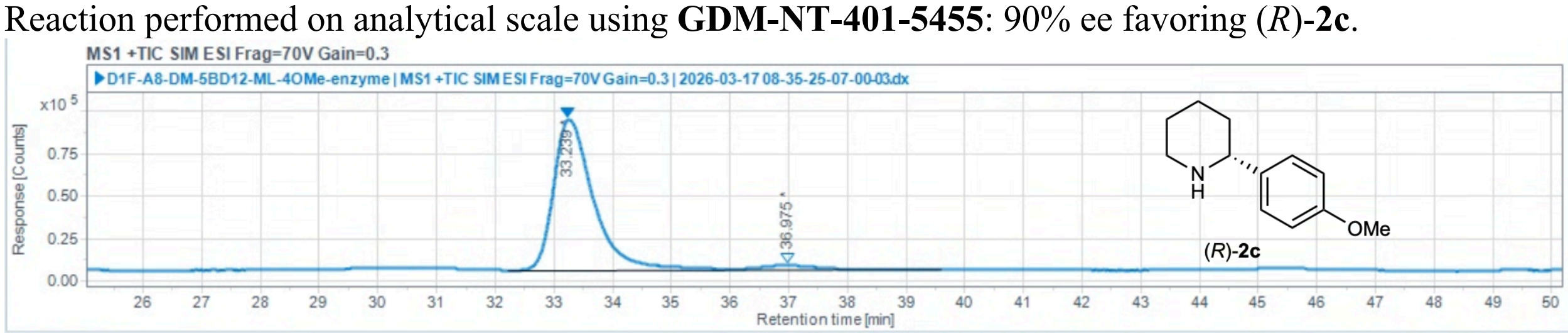

Injection Results

Peaks Summary

#	Name	Signal description	RT (min)	Area (Counts·s)	Area%	Height (Counts)	Height%	Amount	Concentration	Start tim
1		MS1 +TIC SIM ESI Frag=70V Gain=0.3	33.239	4186469.225	94.999	88813.374	96.89			
2		MS1 +TIC SIM ESI Frag=70V Gain=0.3	36.975	220403.147	5.001	2854.616	3.11			

2-(p-tolyl)piperidine (2d)
Chiral separation method: polar ionic HPLC-MS using *dual* Agilent InfinityLab Poroshell 120 Chiral-V, 2.7 µm, 2.1 × 150 mm; eluent: 40% MeOH (0.2 w% NH_4CO_2H), 60% H_2O (20 mM NH_4CO_2H, pH 4.0 adjusted with HCO_2H); flow rate: 0.2 mL/min. t_R = 31.902 min, t_S = 38.191 min.

(*rac*)-**2d** (commercially available standard):

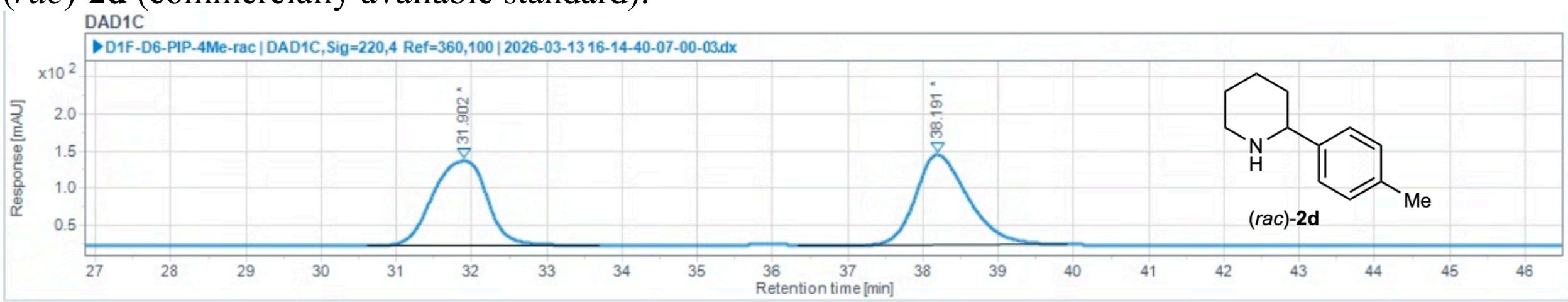

Injection Results

Peaks | Summary

#	Name	Signal description	RT (min)	Area (mAU·s)	Area%	Height (mAU)	Height%	Amount	Concentration	Start tim
1		DAD1C,Sig=220,4 Ref=360,100	31.902	5669.445	49.424	113.074	48.45			
2		DAD1C,Sig=220,4 Ref=360,100	38.191	5801.655	50.576	120.325	51.55			

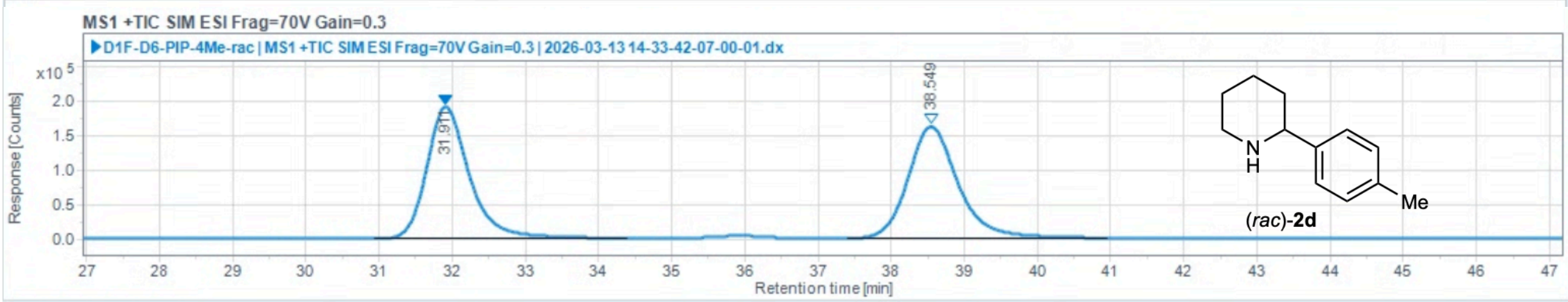

Injection Results

Peaks | Summary

#	Name	Signal description	RT (min)	Area (Counts·s)	Area%	Height (Counts)	Height%	Amount	Concentration	Start tim
1		MS1 +TIC SIM ESI Frag=70V Gain=0.3	31.911	7679986.394	50.051	190124.084	54.17			
2		MS1 +TIC SIM ESI Frag=70V Gain=0.3	38.549	7664386.118	49.949	160841.833	45.83			

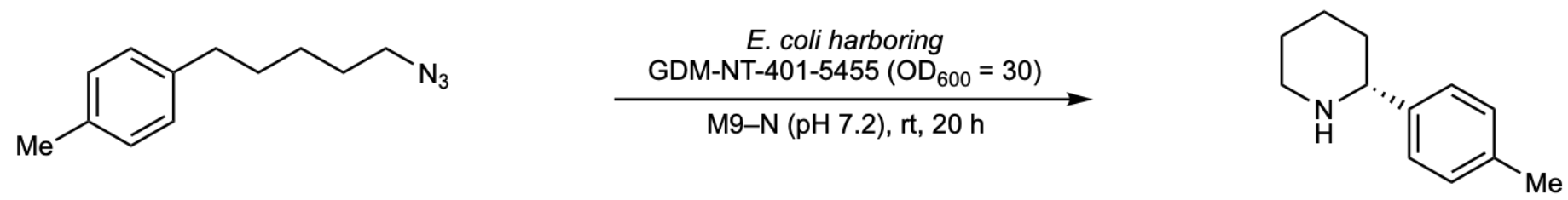

1d **2d**

Reaction performed on analytical scale using **GDM-NT-401-5455**: 95% ee favoring (*R*)-**2d**.

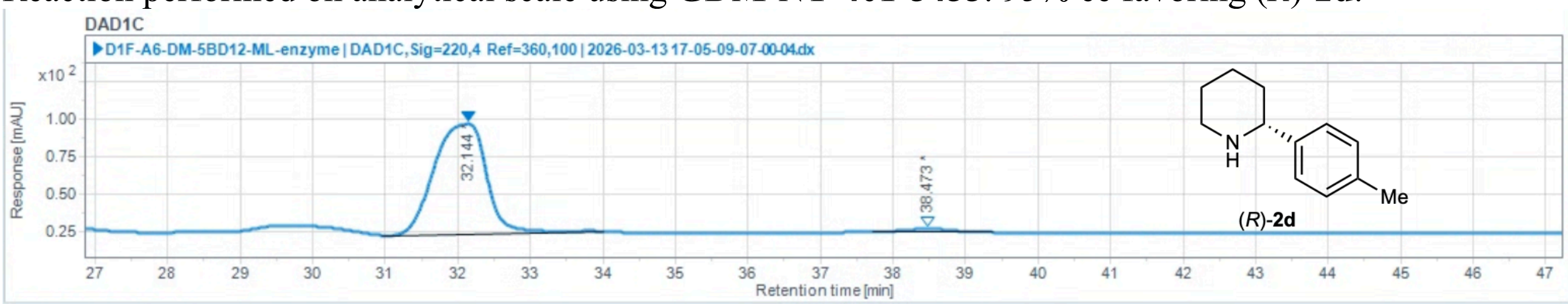

Injection Results

Peaks Summary

#	Name	Signal description	RT (min)	Area (mAU·s)	Area%	Height (mAU)	Height%	Amount	Concentration	Start tim
1		DAD1C,Sig=220,4 Ref=360,100	32.144	3663.540	97.501	73.382	97.22			
2		DAD1C,Sig=220,4 Ref=360,100	38.473	93.883	2.499	2.096	2.78			

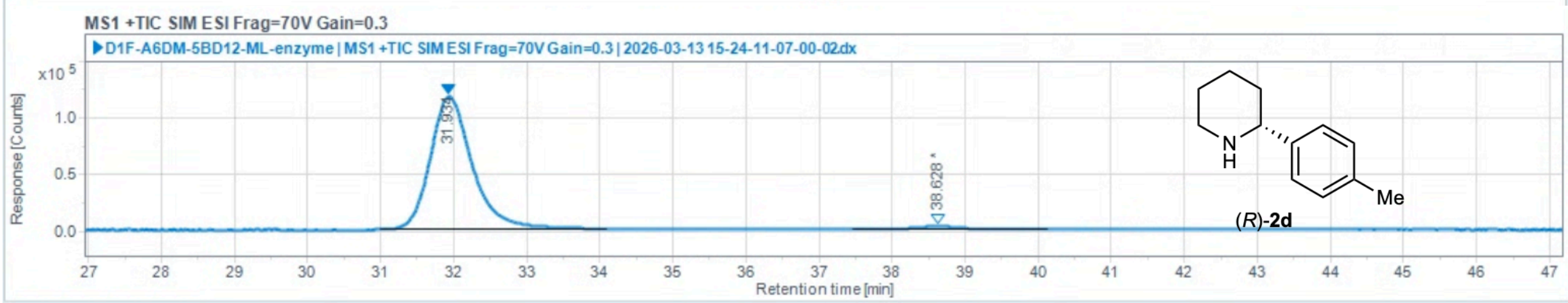

Injection Results

Peaks Summary

#	Name	Signal description	RT (min)	Area (Counts·s)	Area%	Height (Counts)	Height%	Amount	Concentration	Start tim
1		MS1 +TIC SIM ESI Frag=70V Gain=0.3	31.934	4689084.199	97.331	117454.964	97.69			
2		MS1 +TIC SIM ESI Frag=70V Gain=0.3	38.628	128569.878	2.669	2780.380	2.31			

2-(4-chlorophenyl)piperidine (2e)
Chiral separation method: polar ionic HPLC-MS using *dual* Agilent InfinityLab Poroshell 120 Chiral-V, 2.7 µm, 2.1 × 150 mm; eluent: 40% MeOH (0.2 w% NH_4CO_2H), 60% H_2O (20 mM NH_4CO_2H, pH 4.0 adjusted with HCO_2H); flow rate: 0.2 mL/min. t_R = 39.843 min, t_S = 43.259 min.

(*rac*)-**2e** (commercially available standard):

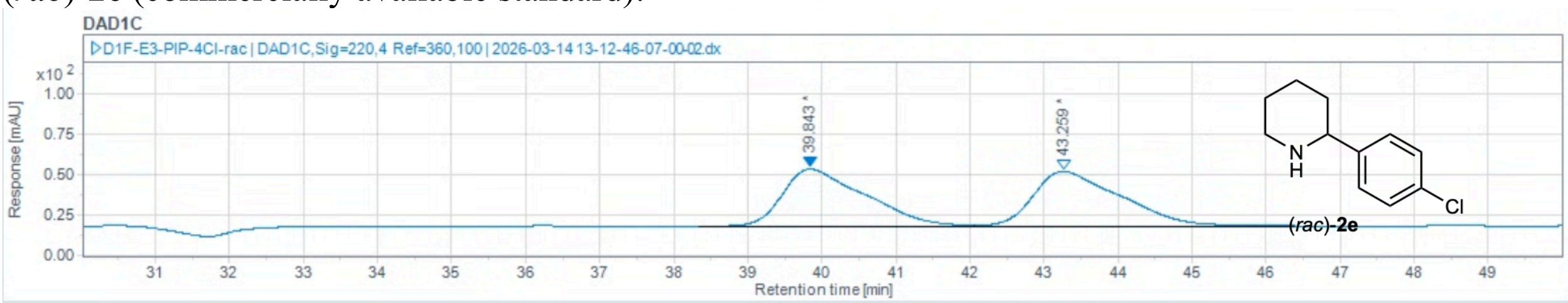

Injection Results

#	Name	Signal description	RT (min)	Area (mAU·s)	Area%	Height (mAU)	Height%	Amount	Concentration	Start tim
1		DAD1C,Sig=220,4 Ref=360,100	39.843	2763.003	49.872	35.547	51.10			
2		DAD1C,Sig=220,4 Ref=360,100	43.259	2777.230	50.128	34.023	48.90			

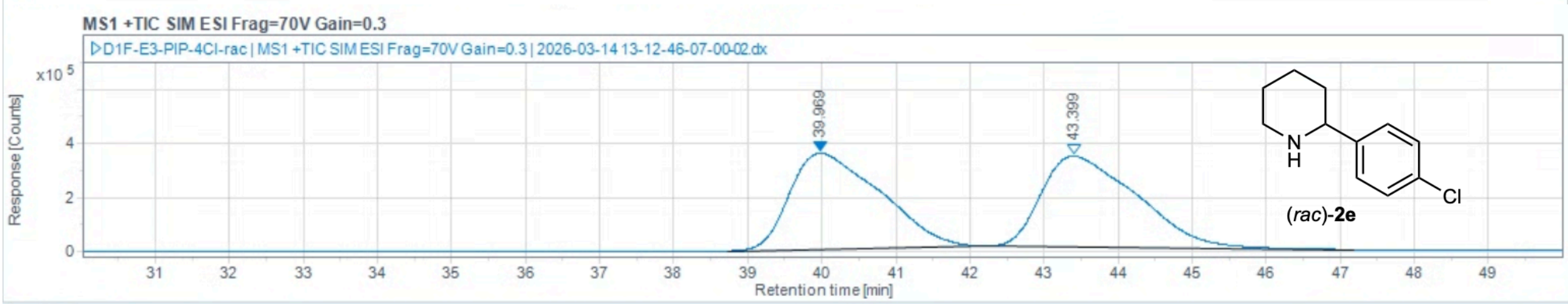

Injection Results

#	Name	Signal description	RT (min)	Area (Counts·s)	Area%	Height (Counts)	Height%	Amount	Concentration	Start tim
1		MS1 +TIC SIM ESI Frag=70V Gain=0.3	39.969	30180876.610	49.930	355181.664	51.36			
2		MS1 +TIC SIM ESI Frag=70V Gain=0.3	43.399	30265091.243	50.070	336422.630	48.64			

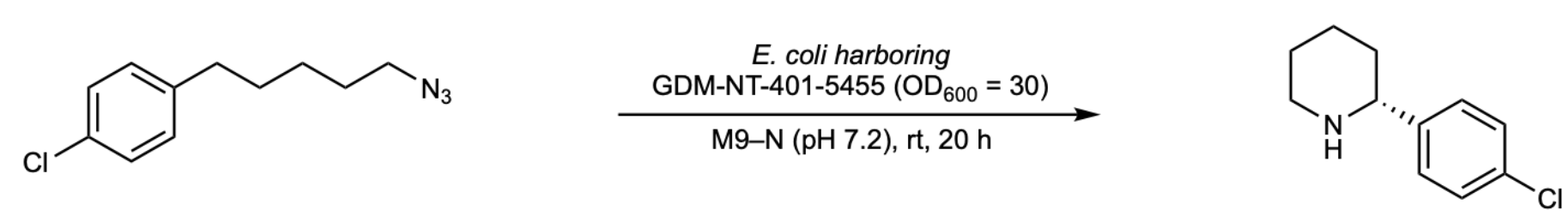

1e **2e**

Reaction performed on analytical scale using **GDM-NT-401-5455**: 98% ee favoring (*R*)-**2d**.

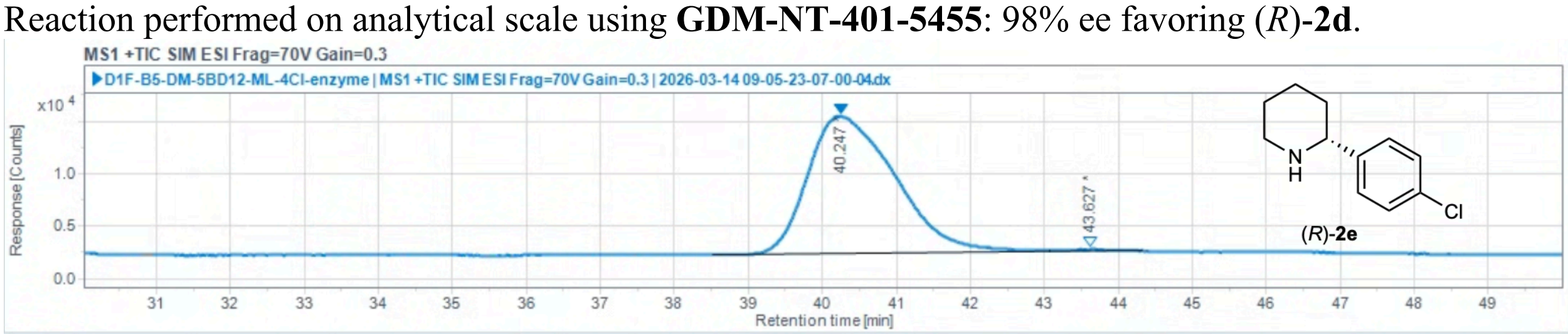

Injection Results

#	Name	Signal description	RT (min)	Area (Counts·s)	Area%	Height (Counts)	Height%	Amount	Concentration	Start tim
1		MS1 +TIC SIM ESI Frag=70V Gain=0.3	40.247	1077781.535	99.164	13085.857	98.56			
2		MS1 +TIC SIM ESI Frag=70V Gain=0.3	43.627	9089.772	0.836	191.822	1.44			

2-(4-fluorophenyl)piperidine (2f)

Chiral separation method: polar ionic HPLC-MS using *dual* Agilent InfinityLab Poroshell 120 Chiral-V, 2.7 µm, 2.1 × 150 mm; eluent: 40% MeOH (0.2 w% NH_4CO_2H), 60% H_2O (20 mM NH_4CO_2H, pH 4.0 adjusted with HCO_2H); flow rate: 0.2 mL/min. t_R = 30.163 min, t_S = 31.511 min.

(*rac*)-**2f** (commercially available standard):

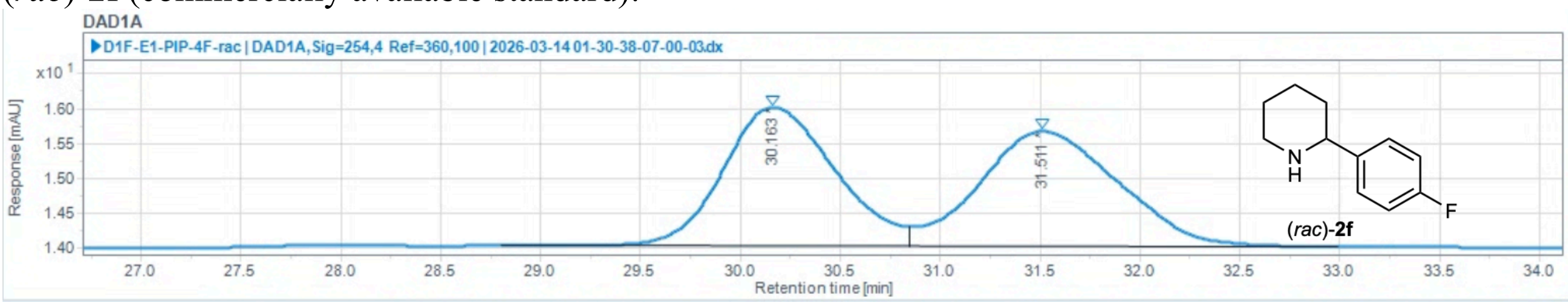

Injection Results

Peaks Summary

#	Name	Signal description	RT (min)	Area (mAU·s)	Area%	Height (mAU)	Height%	Amount	Concentration	Start tim
1		DAD1A,Sig=254,4 Ref=360,100	30.163	77.217	49.283	1.982	54.66			
2		DAD1A,Sig=254,4 Ref=360,100	31.511	79.465	50.717	1.644	45.34			

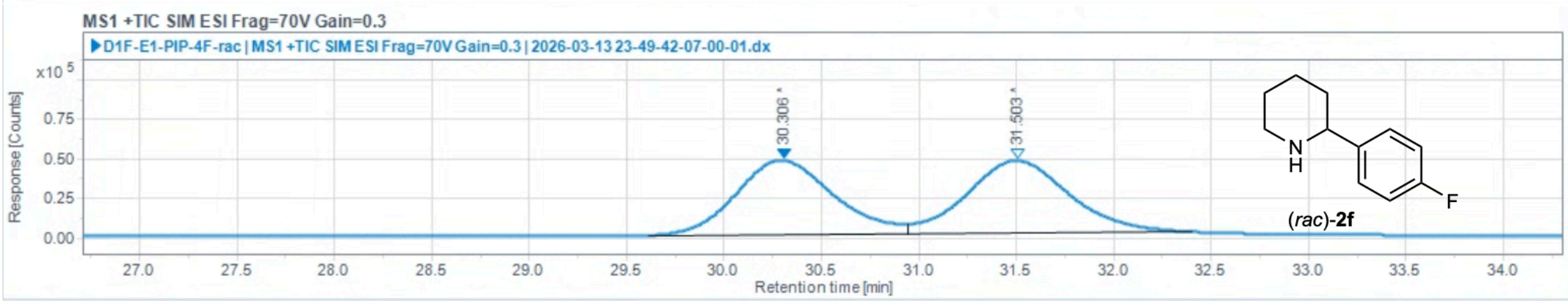

Injection Results

Peaks Summary

#	Name	Signal description	RT (min)	Area (Counts·s)	Area%	Height (Counts)	Height%	Amount	Concentration	Start tim
1		MS1 +TIC SIM ESI Frag=70V Gain=0.3	30.306	1644114.358	49.372	47123.121	50.63			
2		MS1 +TIC SIM ESI Frag=70V Gain=0.3	31.503	1685944.497	50.628	45949.804	49.37			

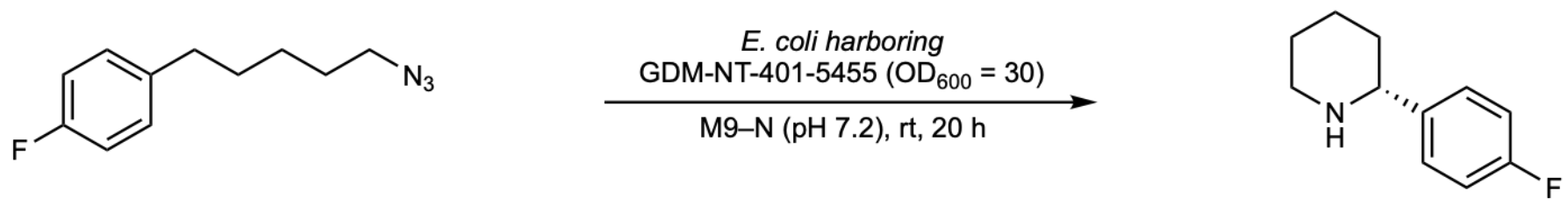

1f **2f**

Reaction performed on analytical scale using **GDM-NT-401-5455**: 89% ee favoring (*R*)-**2f**.

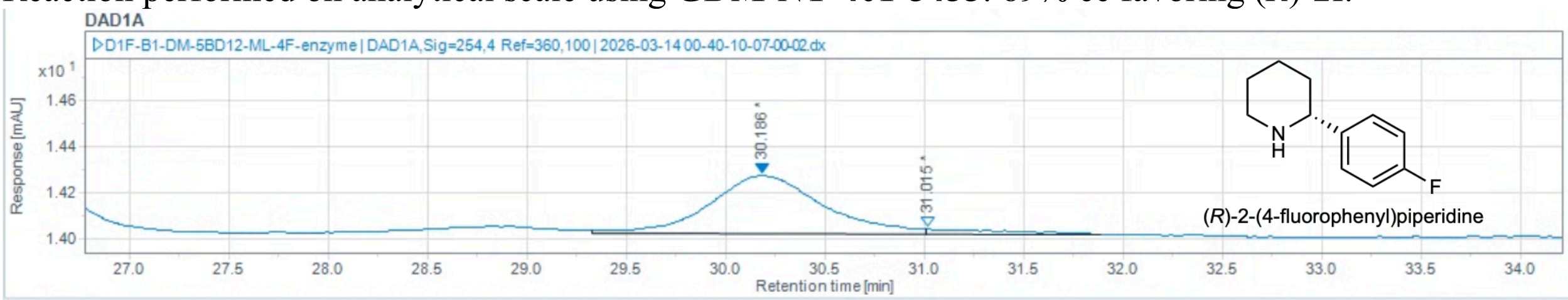

Injection Results

Peaks | Summary

#	Name	Signal description	RT (min)	Area (mAU·s)	Area%	Height (mAU)	Height%	Amount	Concentration	Start tim
1		DAD1A,Sig=254,4 Ref=360,100	30.186	10.046	94.637	0.252	92.17			
2		DAD1A,Sig=254,4 Ref=360,100	31.015	0.569	5.363	0.021	7.83			

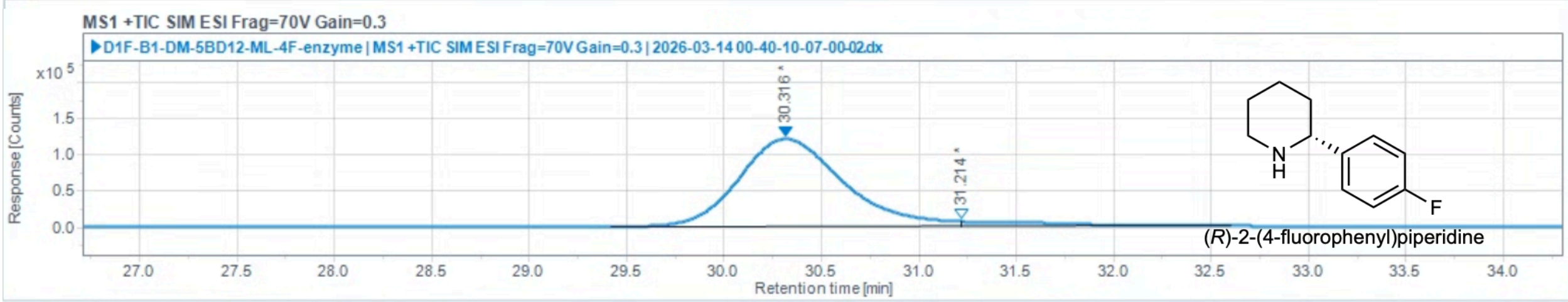

Injection Results

Peaks | Summary

#	Name	Signal description	RT (min)	Area (Counts·s)	Area%	Height (Counts)	Height%	Amount	Concentration	Start tim
1		MS1 +TIC SIM ESI Frag=70V Gain=0.3	30.316	4526151.842	95.489	121277.039	94.89			
2		MS1 +TIC SIM ESI Frag=70V Gain=0.3	31.214	213842.548	4.511	6533.229	5.11			

2-(4-(trifluoromethyl)phenyl)piperidine (2g)

Chiral separation method: chiral GC using chiral column Cyclosil-B (S/N USC696124H). t_S = 23.409 min, t_R = 23.486 min.

(*rac*)-**2g** (commercially available standard):

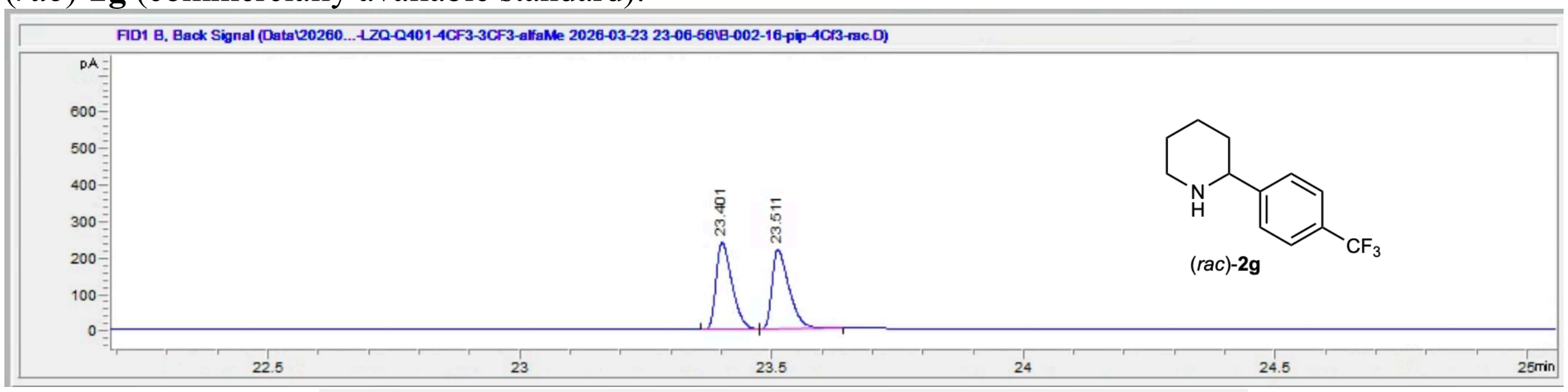

#	Time	Type	Area	Height	Width	Area%	Symmetry
1	23.401	BB	523.1	237.6	0.0329	50.779	0.6
2	23.511	BB	507.1	216.9	0.0361	49.221	0.504

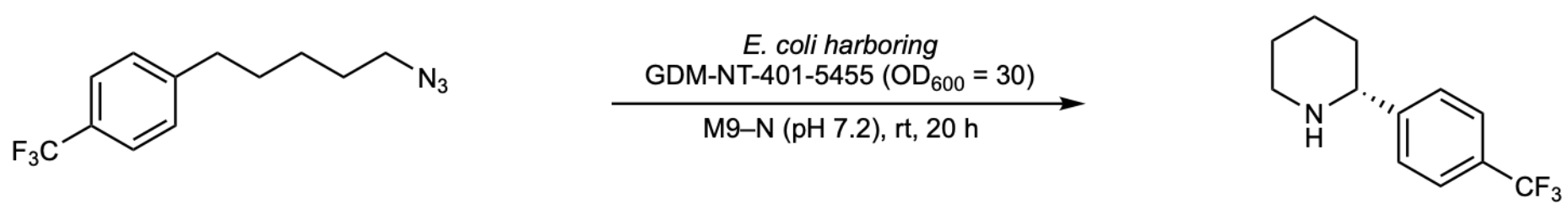

1g **2g**

Reaction performed on analytical scale using **GDM-NT-401-5455**: 87% ee favoring (*R*)-**2g**.

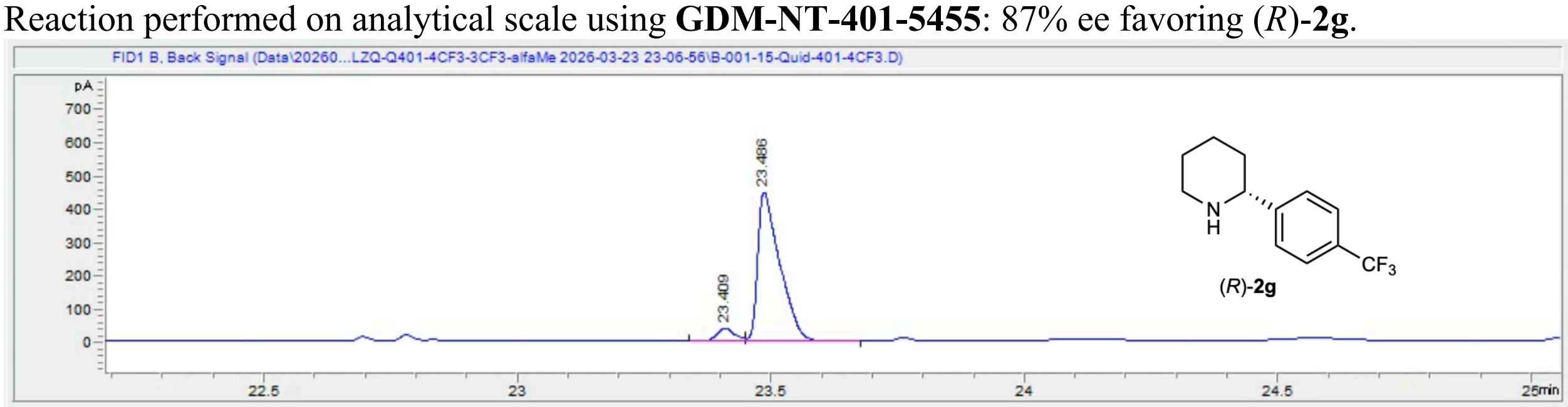

#	Time	Type	Area	Height	Width	Area%	Symmetry
1	23.409	BV	93.4	39	0.0352	6.474	0.854
2	23.486	VB	1349	445.6	0.0425	93.526	0.388

2-(3-methoxyphenyl)piperidine (2h)
Chiral separation method: normal phase HPLC using Daicel chiralpak IC column; eluent: hexanes:*i*-Pr-OH (90:10) with 0.1 v% diethylamine as additive; flowrate: 1.0 mL/min. t_R = 9.144 min, t_S = 10.291 min.

(*rac*)-**2h** (commercially available standard):

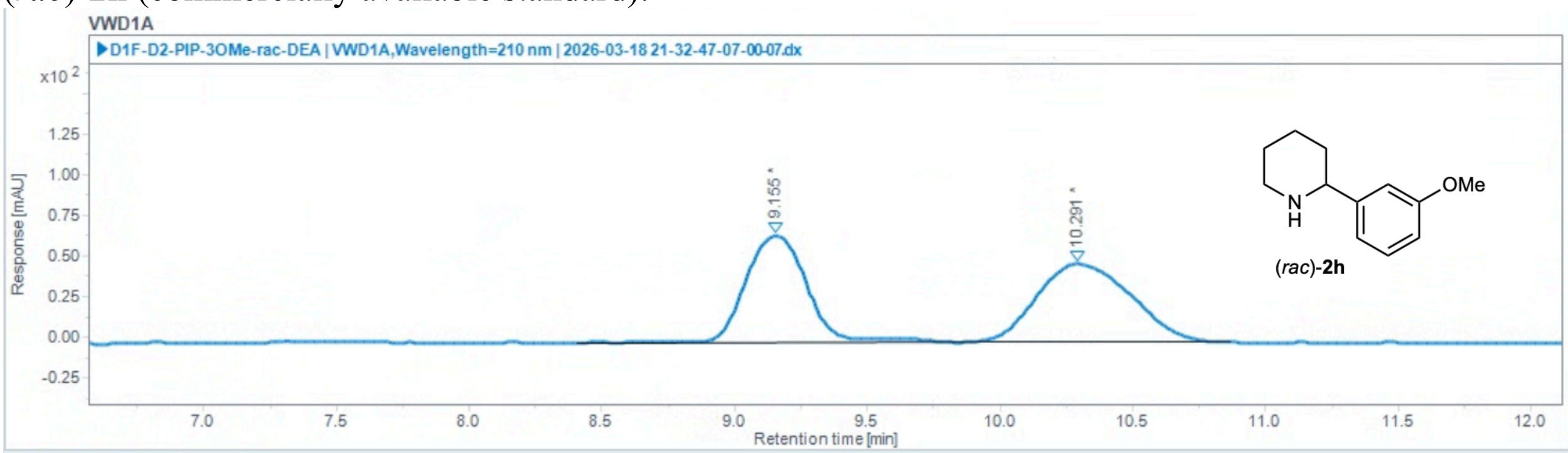

Injection Results

Peaks Summary

Name	Signal description	RT (min)	Area (mAU·s)	Area%	Height (mAU)	Height%	Start time (min)	End time (min)
	VWD1A,Wavelength=210 nm	9.155	1040.382	47.008	65.339	57.87	8.407	9.912
	VWD1A,Wavelength=210 nm	10.291	1172.823	52.992	47.576	42.13	9.912	10.869

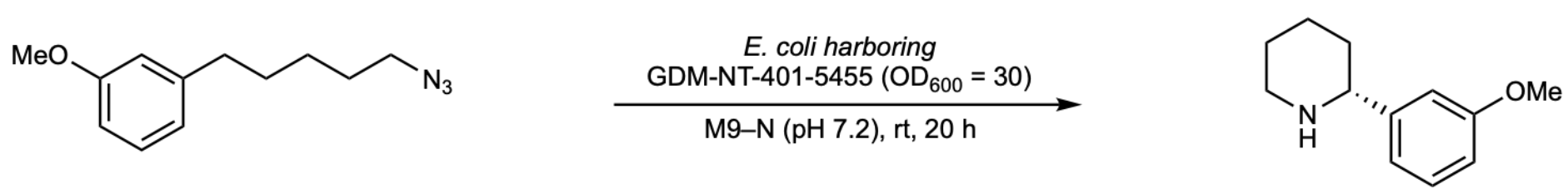

1h **2h**

Reaction performed on analytical scale using **GDM-NT-401-5455**: >99% ee favoring (*R*)-**2h**.

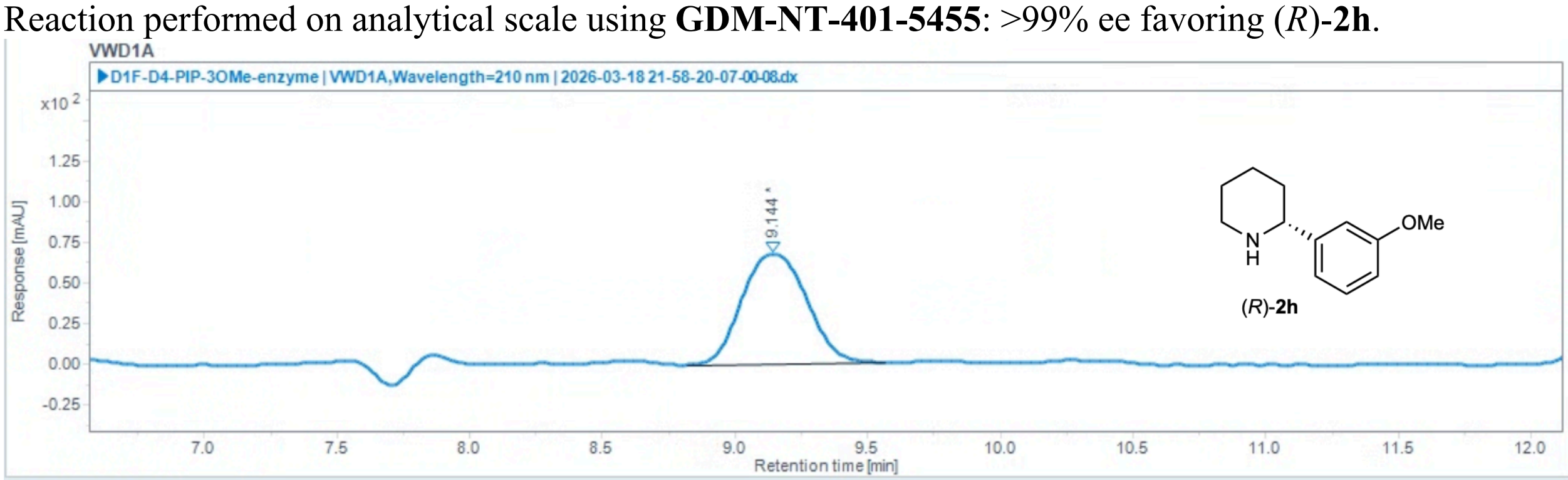

Injection Results

Peaks Summary

Name	Signal description	RT (min)	Area (mAU·s)	Area%	Height (mAU)	Height%	Start time (min)	End time (min)
	VWD1A,Wavelength=210 nm	9.144	1149.552	100.000	67.522	100.00	8.817	9.567

2-(3-methylphenyl)piperidine (2i_Ts)
Chiral separation method: normal phase HPLC using Daicel chiralpak IB column; eluent: hexanes:*i*-Pr-OH (95:5); flowrate: 0.6 mL/min. t_S = 13.353 min, t_R = 14.563 min.

(*rac*)-**2i_Ts** (commercially available standard):

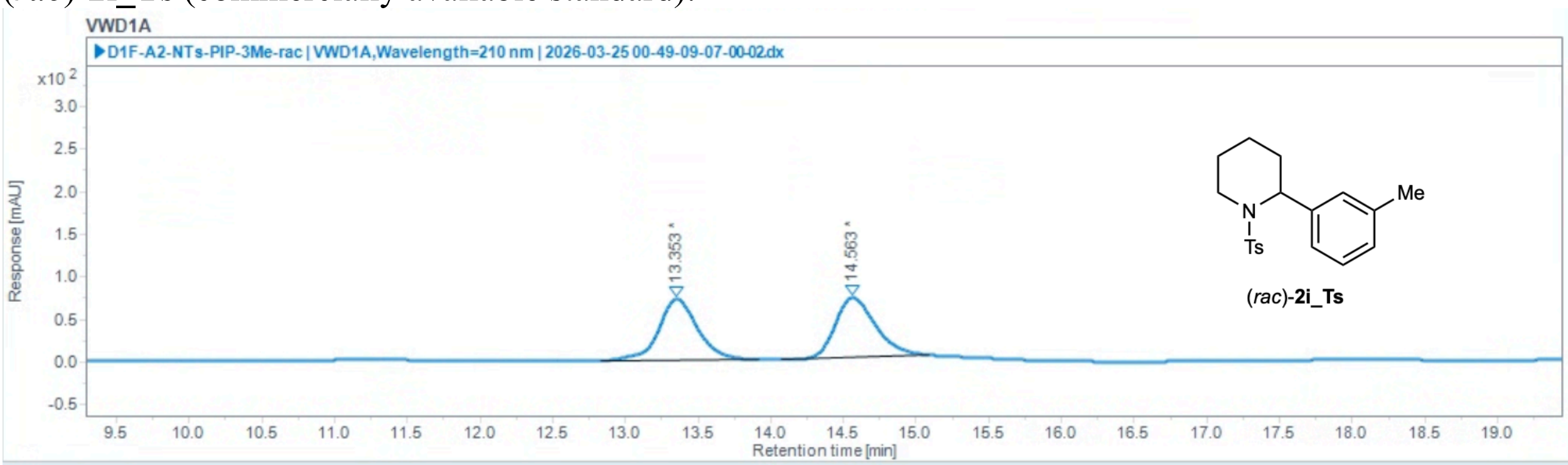

Injection Results

Peaks Summary

Name	Signal description	RT (min)	Area (mAU·s)	Area%	Height (mAU)	Height%	Start time (min)	End time (min)
	VWD1A,Wavelength=210 nm	13.353	1353.378	49.925	72.110	50.61	12.838	13.924
	VWD1A,Wavelength=210 nm	14.563	1357.431	50.075	70.361	49.39	14.079	15.093

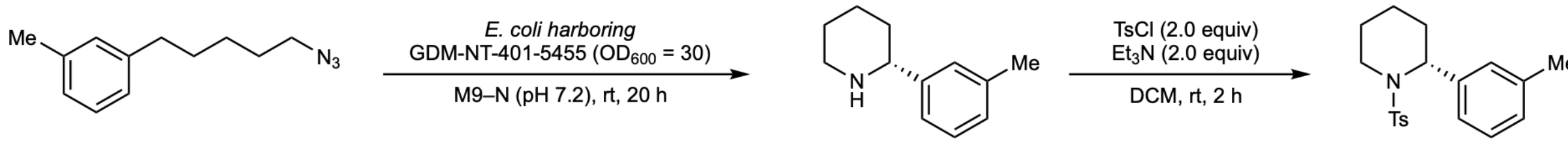

1i **2i** **2i_Ts**

Reaction performed on analytical scale using **GDM-NT-401-5455**: 94% ee favoring (*R*)-**2i**.

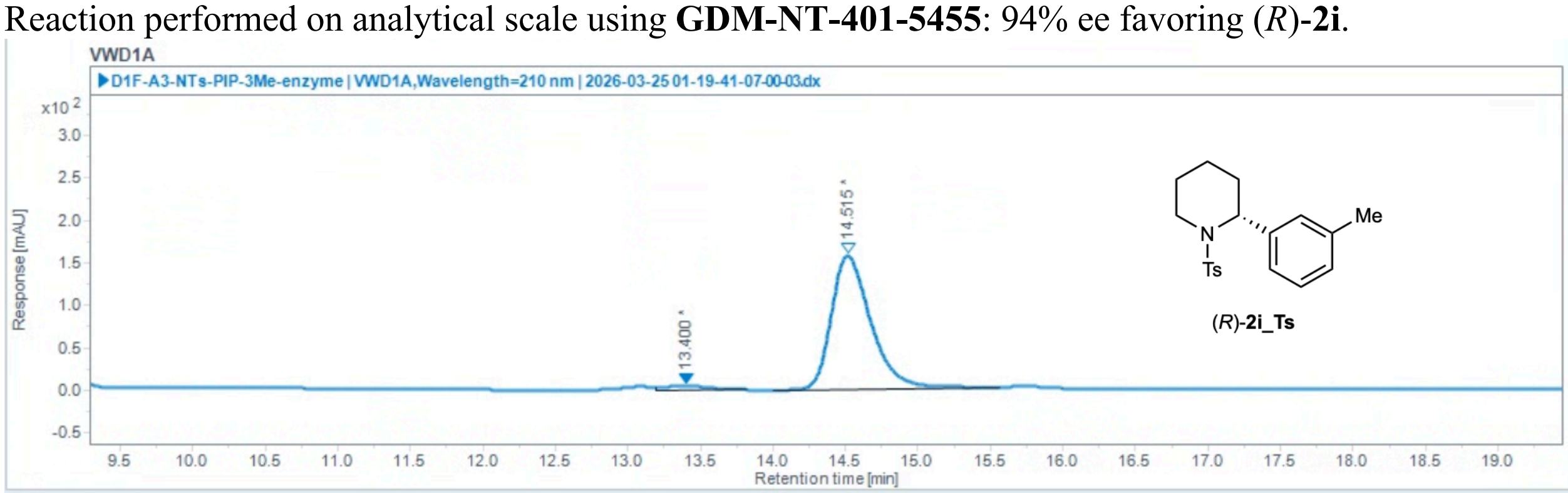

Injection Results

Peaks Summary

Name	Signal description	RT (min)	Area (mAU·s)	Area%	Height (mAU)	Height%	Start time (min)	End time (min)
	VWD1A,Wavelength=210 nm	13.400	94.867	3.104	4.703	2.92	13.200	13.821
	VWD1A,Wavelength=210 nm	14.515	2961.189	96.896	156.314	97.08	14.007	15.569

2-(3-fluorophenyl)piperidine (2j)
Chiral separation method: polar ionic HPLC-MS using *dual* Agilent InfinityLab Poroshell 120 Chiral-V, 2.7 µm, 2.1 × 150 mm; eluent: 40% MeOH (0.2 w% NH_4CO_2H), 60% H_2O (20 mM NH_4CO_2H, pH 4.0 adjusted with HCO_2H); flow rate: 0.2 mL/min. t_R = 29.974 min, t_S = 31.838 min.

(*rac*)-**2j** (commercially available standard):

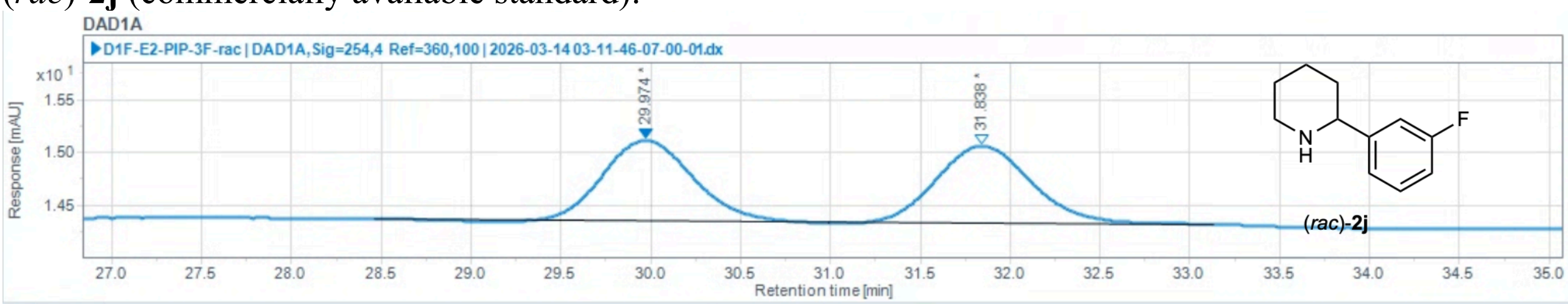

Injection Results

Peaks | Summary

#	Name	Signal description	RT (min)	Area (mAU·s)	Area%	Height (mAU)	Height%	Amount	Concentration	Start tim
1		DAD1A,Sig=254,4 Ref=360,100	29.974	26.382	49.431	0.766	51.17			
2		DAD1A,Sig=254,4 Ref=360,100	31.838	26.990	50.569	0.731	48.83			

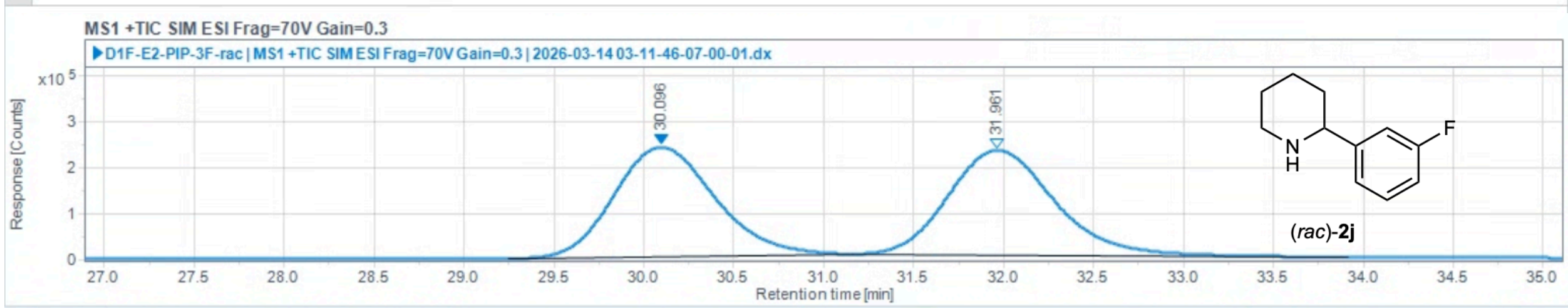

Injection Results

Peaks | Summary

#	Name	Signal description	RT (min)	Area (Counts·s)	Area%	Height (Counts)	Height%	Amount	Concentration	Start tim
1		MS1 +TIC SIM ESI Frag=70V Gain=0.3	30.096	9312011.716	49.689	238722.520	51.08			
2		MS1 +TIC SIM ESI Frag=70V Gain=0.3	31.961	9428684.231	50.311	228584.034	48.92			

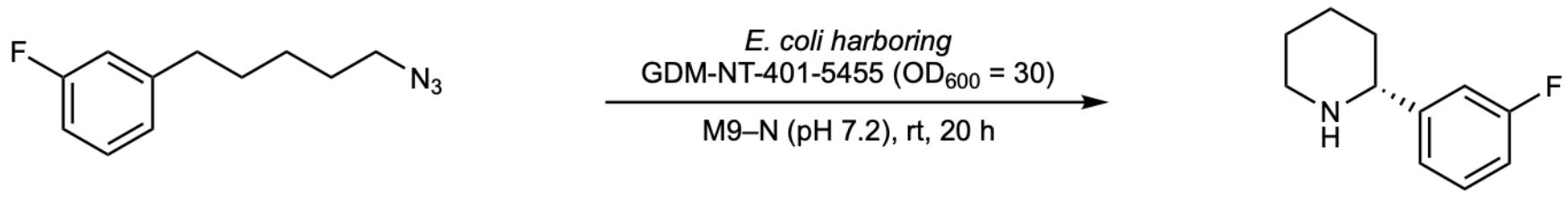

1j **2j**

Reaction performed on analytical scale using **GDM-NT-401-5455**: >99% ee favoring (*R*)-**2j**.

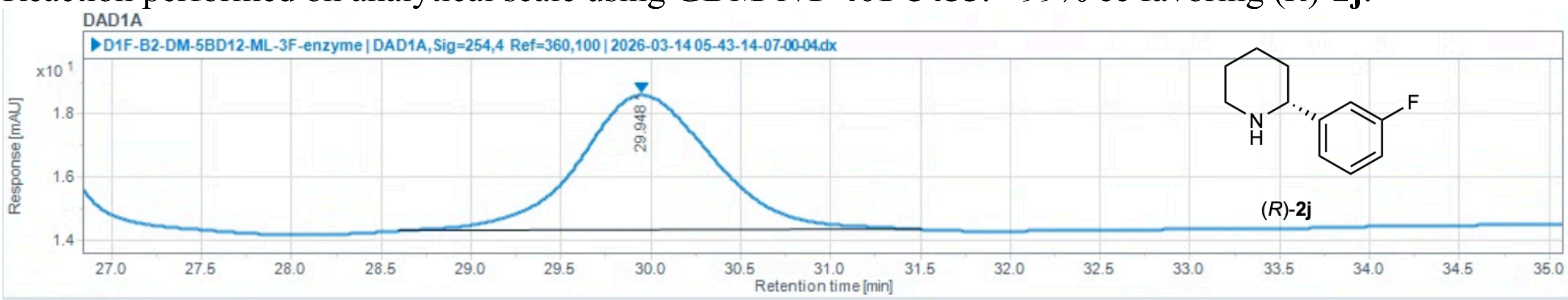

Injection Results

Peaks Summary

#	Name	Signal description	RT (min)	Area (mAU·s)	Area%	Height (mAU)	Height%	Amount	Concentration	Start tim
1		DAD1A,Sig=254,4 Ref=360,100	29.948	224.422	100.000	4.294	100.00			

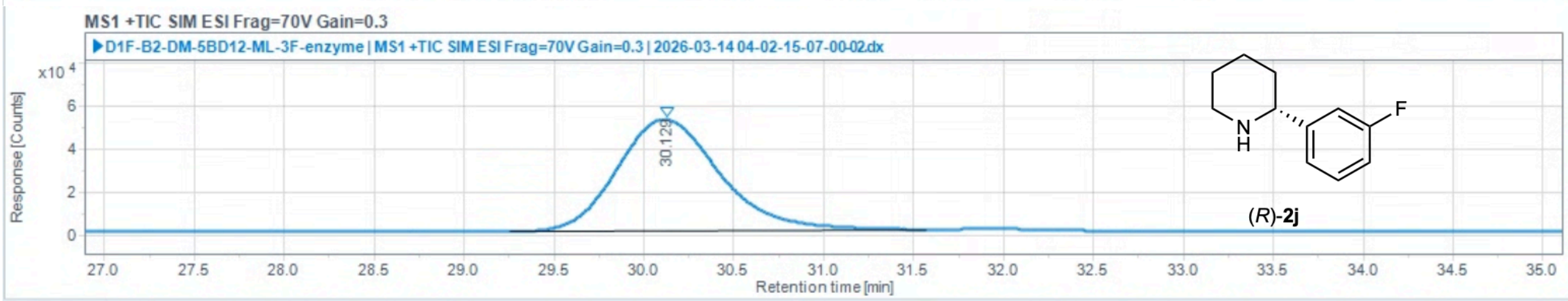

Injection Results

Peaks Summary

#	Name	Signal description	RT (min)	Area (Counts·s)	Area%	Height (Counts)	Height%	Amount	Concentration	Start tim
1		MS1 +TIC SIM ESI Frag=70V Gain=0.3	30.129	2061064.255	100.000	51659.638	100.00			

2-(3-(trifluoromethyl)phenyl)piperidine (2k)

Chiral separation method: chiral GC using chiral column Cyclosil-B (S/N USC696124H). t_S = 22.681 min, t_R = 22.751 min.

(*rac*)-**2k** (commercially available standard):

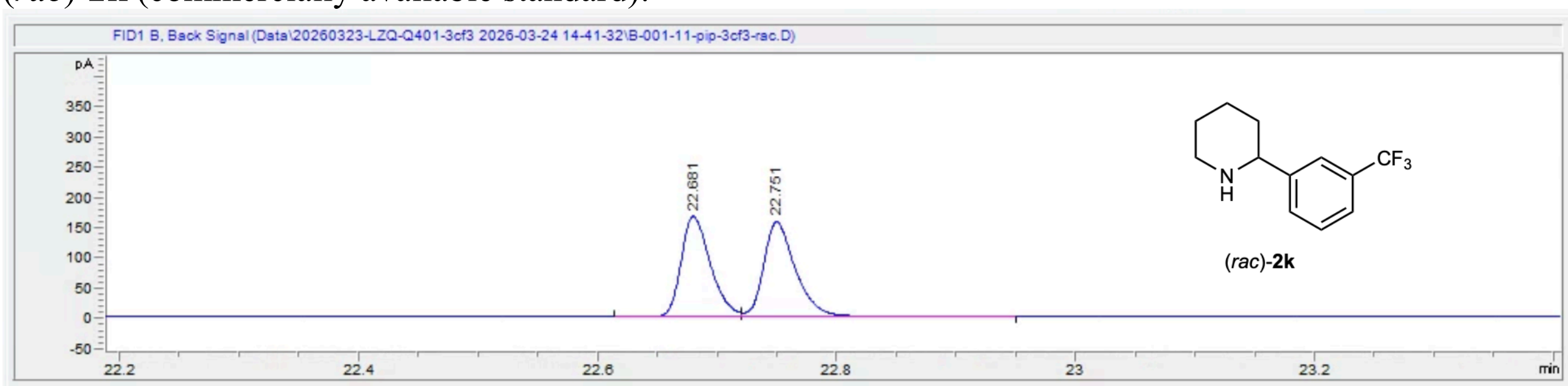

#	Time	Type	Area	Height	Width	Area%	Symmetry
1	22.681	BV	279.8	164.5	0.027	49.337	0.735
2	22.751	VB	287.4	156.2	0.0287	50.663	0.687

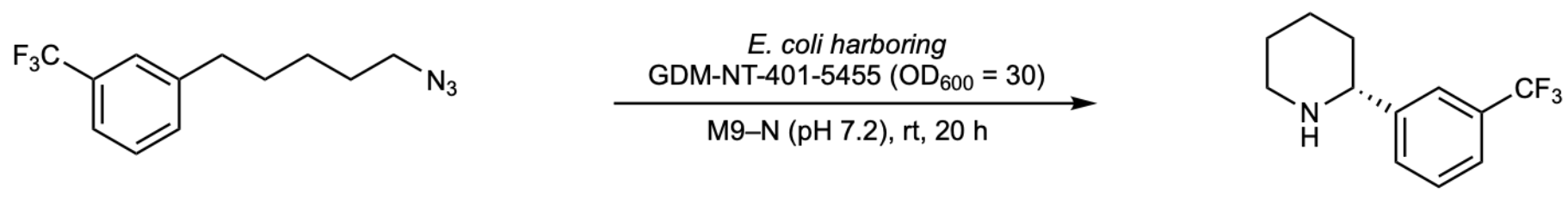

1k **2k**

Reaction performed on analytical scale using **GDM-NT-401-5455**: 95% ee favoring (*R*)-**2k**.

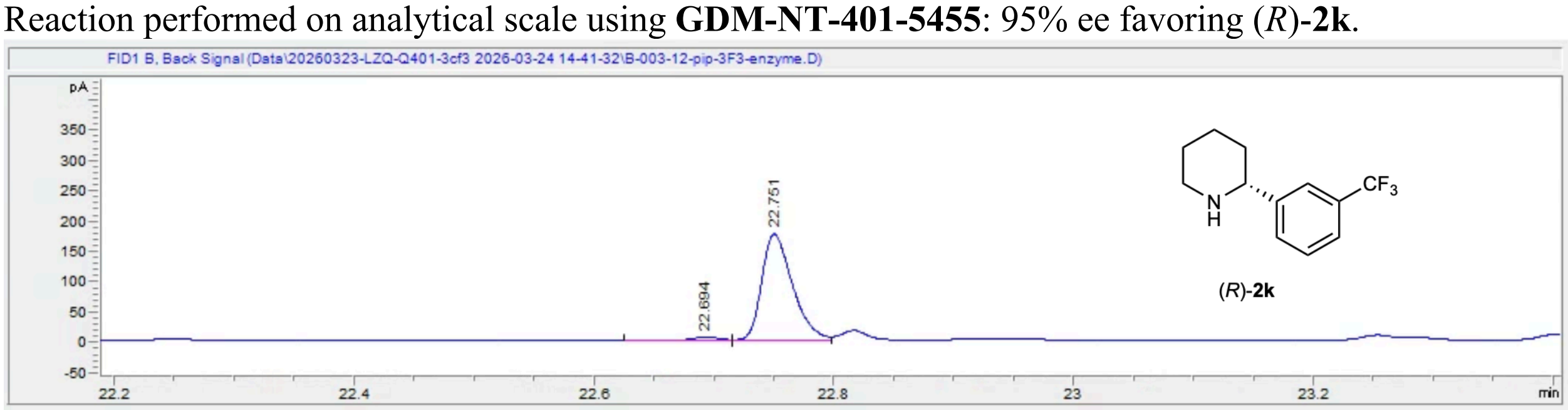

#	Time	Type	Area	Height	Width	Area%	Symmetry
1	22.694	BV	8.2	5.5	0.0238	2.552	0.999
2	22.751	VV	313.2	174.9	0.0281	97.448	0.694

2-(naphthalen-2-yl)piperidine (2l)

Chiral separation method: polar ionic HPLC-MS using *dual* Agilent InfinityLab Poroshell 120 Chiral-V, 2.7 µm, 2.1 × 150 mm; eluent: 40% MeOH (0.2 w% NH_4CO_2H), 60% H_2O (20 mM NH_4CO_2H, pH 4.0 adjusted with HCO_2H); flow rate: 0.2 mL/min. t_R = 66.624 min, t_S = 78.949 min.

(*rac*)-**2l** (commercially available standard):

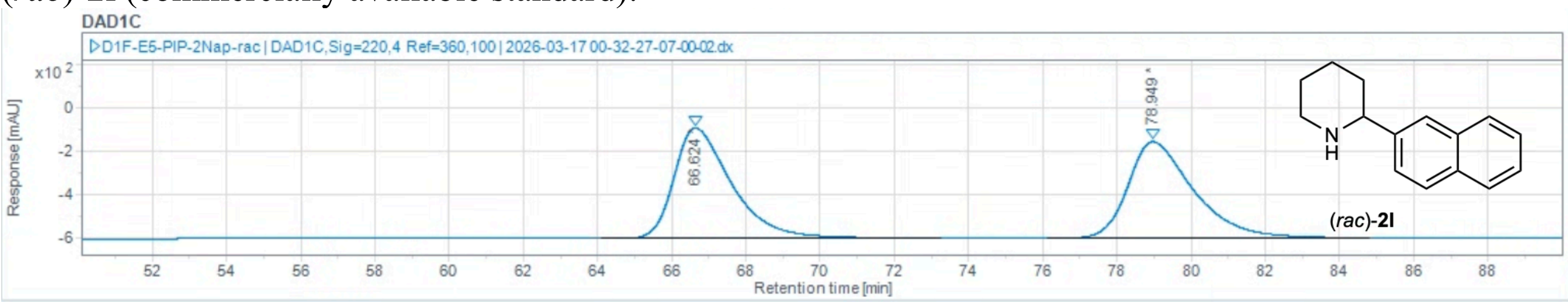

Injection Results

Peaks | Summary

#	Name	Signal description	RT (min)	Area (mAU·s)	Area%	Height (mAU)	Height%	Amount	Concentration	Start tim
1		DAD1C,Sig=220,4 Ref=360,100	66.624	54113.691	50.018	504.433	53.37			
2		DAD1C,Sig=220,4 Ref=360,100	78.949	54073.986	49.982	440.685	46.63			

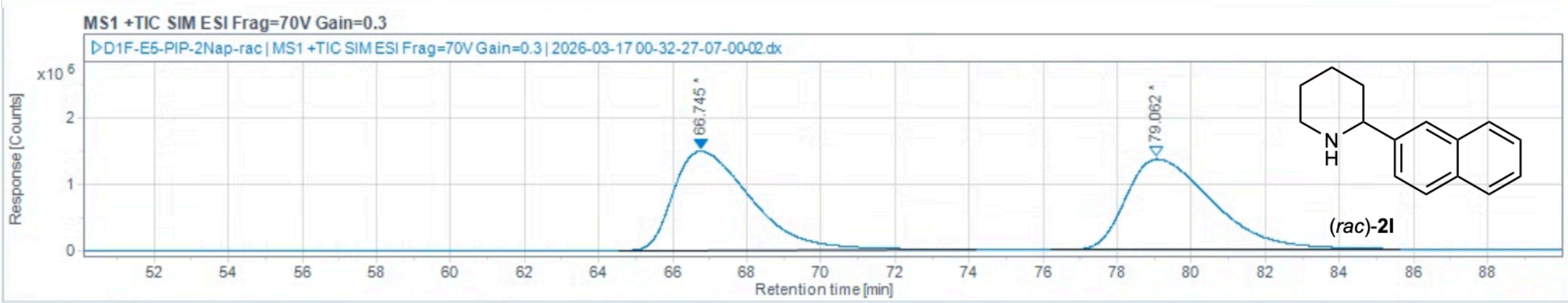

Injection Results

Peaks | Summary

#	Name	Signal description	RT (min)	Area (Counts·s)	Area%	Height (Counts)	Height%	Amount	Concentration	Start tim
1		MS1 +TIC SIM ESI Frag=70V Gain=0.3	66.745	218866336.508	49.596	1497041.905	52.19			
2		MS1 +TIC SIM ESI Frag=70V Gain=0.3	79.062	222436009.551	50.404	1371175.621	47.81			

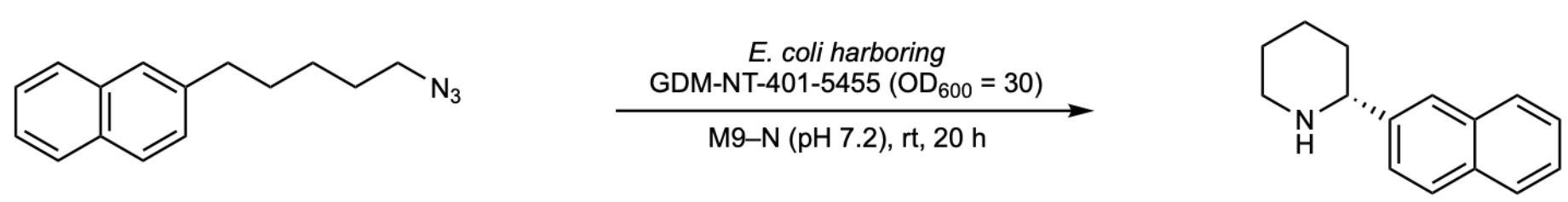

1l **2l**

Reaction performed on analytical scale using **GDM-NT-401-5455**: 89% ee favoring (*R*)-**2l**.

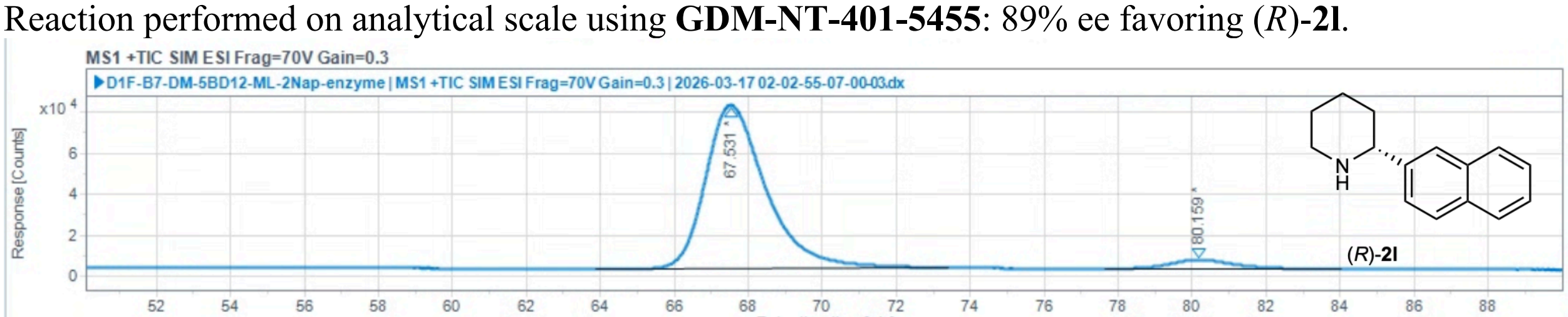

Injection Results

Peaks | Summary

#	Name	Signal description	RT (min)	Area (Counts·s)	Area%	Height (Counts)	Height%	Amount	Concentration	Start tim
1		MS1 +TIC SIM ESI Frag=70V Gain=0.3	67.531	8982932.124	94.481	79526.386	94.72			
2		MS1 +TIC SIM ESI Frag=70V Gain=0.3	80.159	524691.414	5.519	4429.400	5.28			

2-phenylazepane (2m)

Chiral separation method: polar ionic HPLC-MS using *dual* Agilent InfinityLab Poroshell 120 Chiral-V, 2.7 µm, 2.1 × 150 mm; eluent: 40% MeOH (0.2 w% NH_4CO_2H), 60% H_2O (20 mM NH_4CO_2H, pH 4.0 adjusted with HCO_2H); flow rate: 0.2 mL/min. t_1 = 31.105 min, t_2 = 43.372 min.

(*rac*)-**2m** (commercially available standard):

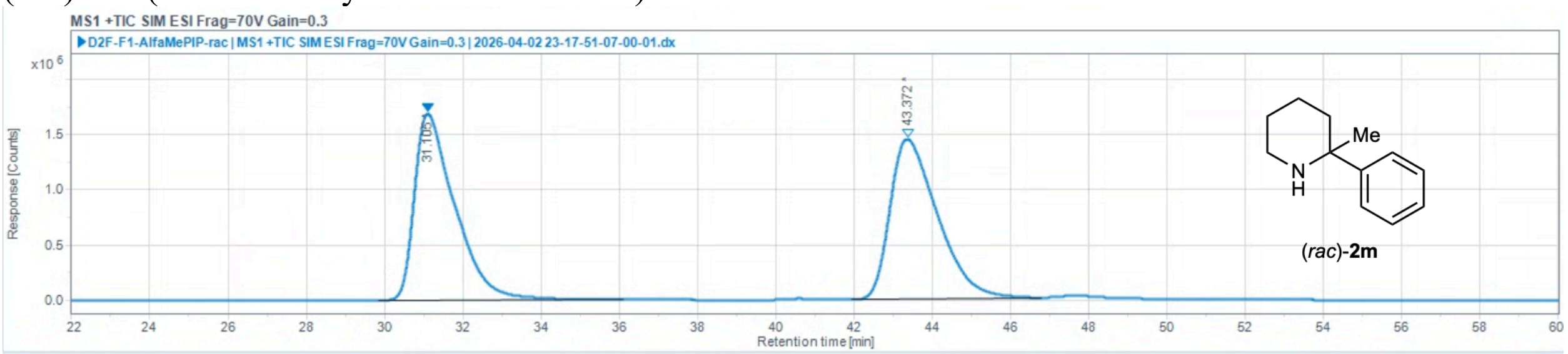

Injection Results

Peaks | Summary

#	Name	Signal description	RT (min)	Area (Counts·s)	Area%	Height (Counts)	Height%	Amount	Concentration	Start time (min)	End time (min)
1		MS1 +TIC SIM ESI Frag=70V Gain=0.3	31.105	120652233.554	49.777	1689689.656	53.87			29.846	36.086
2		MS1 +TIC SIM ESI Frag=70V Gain=0.3	43.372	121734017.317	50.223	1447104.868	46.13			41.952	46.763

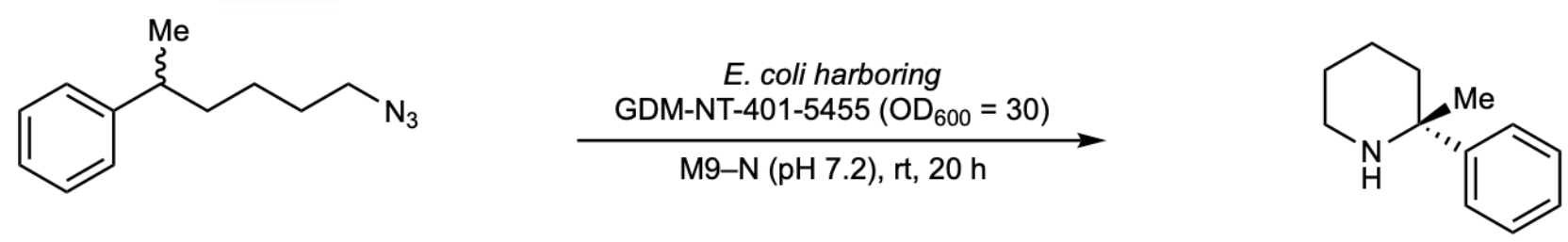

1m **2m**

Reaction performed on analytical scale using **GDM-NT-401-5455**: 36% ee favoring (*R*)-**2m**.

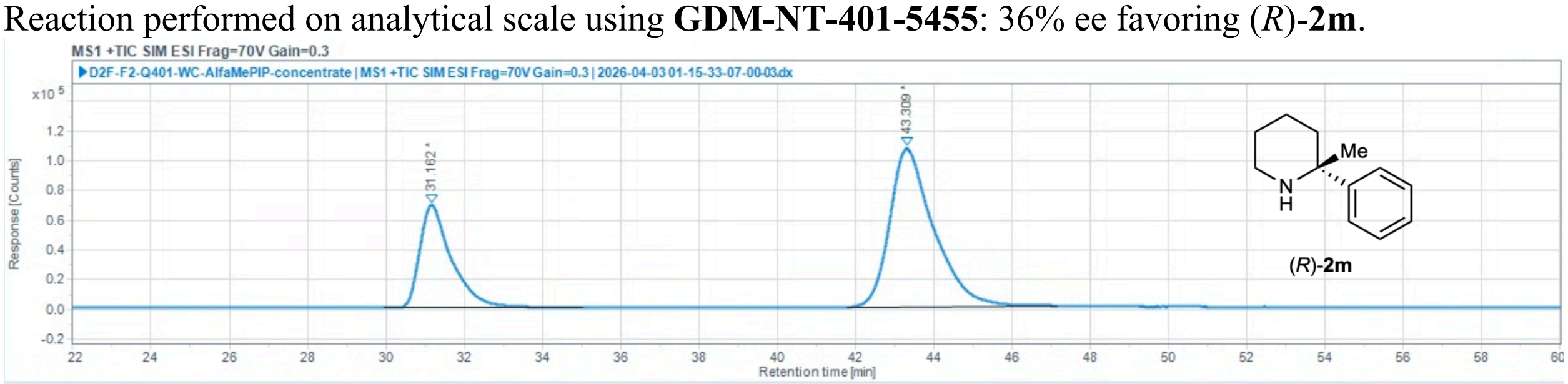

Injection Results

Peaks | Summary

#	Name	Signal description	RT (min)	Area (Counts·s)	Area%	Height (Counts)	Height%	Amount	Concentration	Start time (min)	End time (min)
1		MS1 +TIC SIM ESI Frag=70V Gain=0.3	31.162	3895077.488	31.888	69437.582	39.39			29.952	35.026
2		MS1 +TIC SIM ESI Frag=70V Gain=0.3	43.309	8319617.524	68.112	106846.025	60.61			41.797	47.144

2-phenylazepane (2n)

Chiral separation method: polar ionic HPLC-MS using *dual* Agilent InfinityLab Poroshell 120 Chiral-V, 2.7 µm, 2.1 × 150 mm; eluent: 40% MeOH (0.2 w% NH_4CO_2H), 60% H_2O (20 mM NH_4CO_2H, pH 4.0 adjusted with HCO_2H); flow rate: 0.2 mL/min. t_S = 34.593 min, t_S = 36.125 min.

(*rac*)-**2n** (commercially available standard):

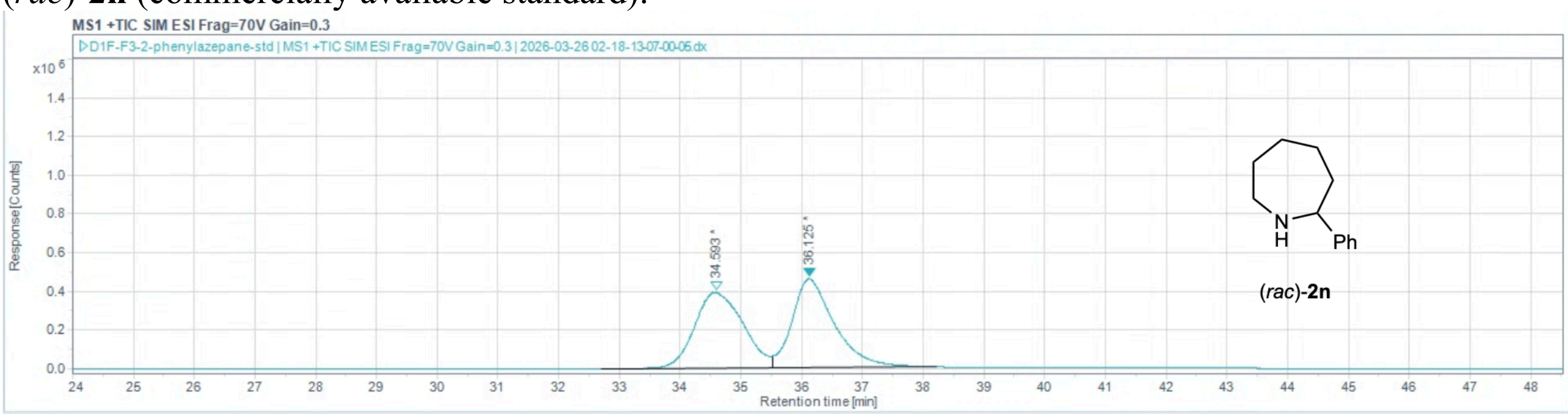

Injection Results

Peaks | Summary

#	Name	Signal description	RT (min)	Area (Counts·s)	Area%	Height (Counts)	Height%	Amount	Concentration	Start time (min)	End time (min)
1		MS1 +TIC SIM ESI Frag=70V Gain=0.3	34.593	21532441.948	49.263	391394.568	45.92			32.711	35.519
2		MS1 +TIC SIM ESI Frag=70V Gain=0.3	36.125	22176390.220	50.737	460855.590	54.08			35.519	38.228

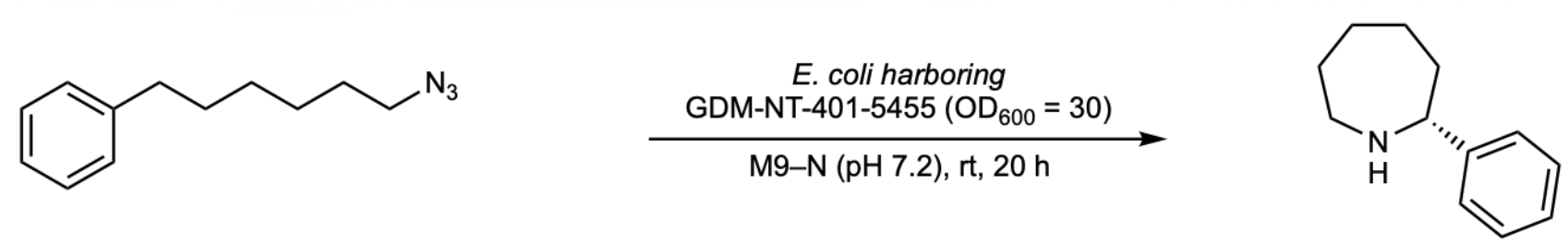

1n **2n**

Reaction performed on analytical scale using **GDM-NT-401-5455**: 98% ee

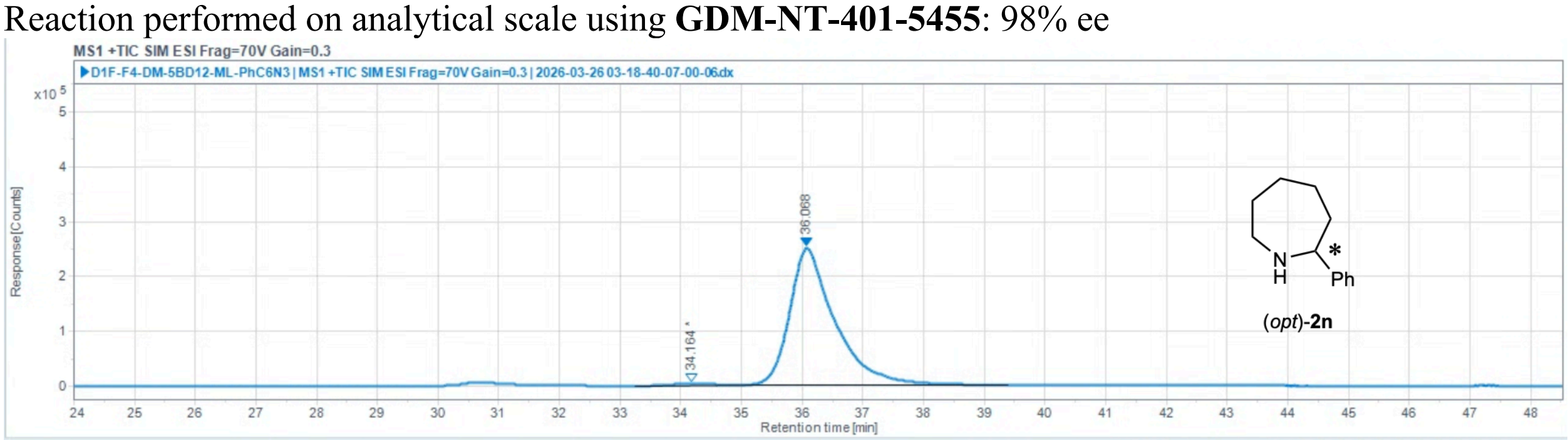

Injection Results

Peaks | Summary

#	Name	Signal description	RT (min)	Area (Counts·s)	Area%	Height (Counts)	Height%	Amount	Concentration	Start time (min)	End time (min)
1		MS1 +TIC SIM ESI Frag=70V Gain=0.3	34.164	136310.449	1.046	3405.375	1.35			33.236	35.065
2		MS1 +TIC SIM ESI Frag=70V Gain=0.3	36.068	12893372.628	98.954	249166.032	98.65			35.065	39.387

2-phenylpyrrolidine (2o)
Chiral separation method: polar ionic HPLC-MS using *dual* Agilent InfinityLab Poroshell 120 Chiral-V, 2.7 µm, 2.1 × 150 mm; eluent: 40% MeOH (0.2 w% NH_4CO_2H), 60% H_2O (20 mM NH_4CO_2H, pH 4.0 adjusted with HCO_2H); flow rate: 0.2 mL/min. t_1 = 32.092 min, t_2 = 33.260 min.

(*rac*)-**2o** (commercially available standard):

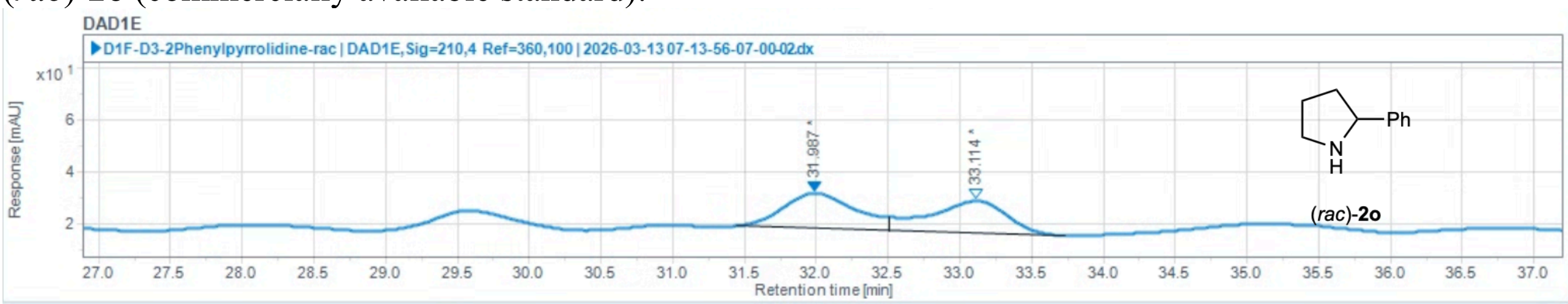

Injection Results

Peaks | Summary

#	Name	Signal description	RT (min)	Area (mAU·s)	Area%	Height (mAU)	Height%	Amount	Concentration	Start tim
1		DAD1E,Sig=210,4 Ref=360,100	31.987	479.985	50.853	13.589	52.20			
2		DAD1E,Sig=210,4 Ref=360,100	33.114	463.884	49.147	12.442	47.80			

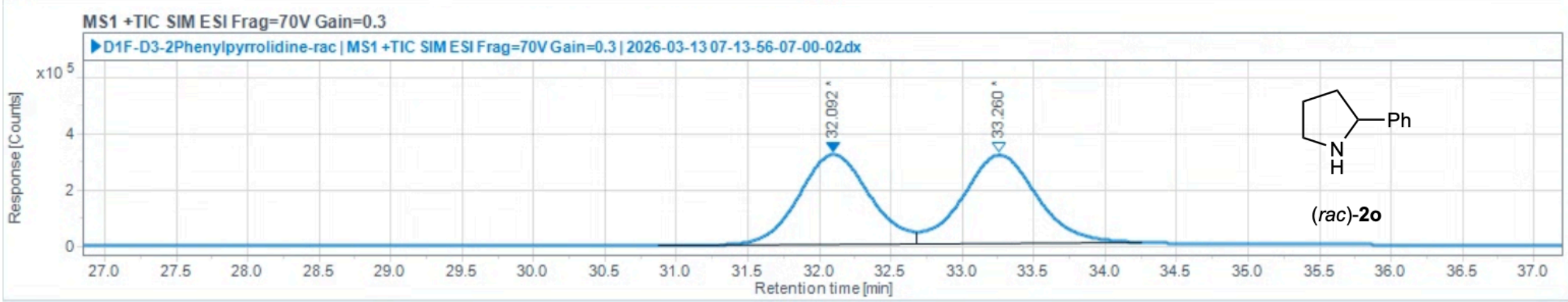

Injection Results

Peaks | Summary

#	Name	Signal description	RT (min)	Area (Counts·s)	Area%	Height (Counts)	Height%	Amount	Concentration	Start tim
1		MS1 +TIC SIM ESI Frag=70V Gain=0.3	32.092	10958013.430	49.032	319228.485	50.38			
2		MS1 +TIC SIM ESI Frag=70V Gain=0.3	33.260	11390788.639	50.968	314351.664	49.62			

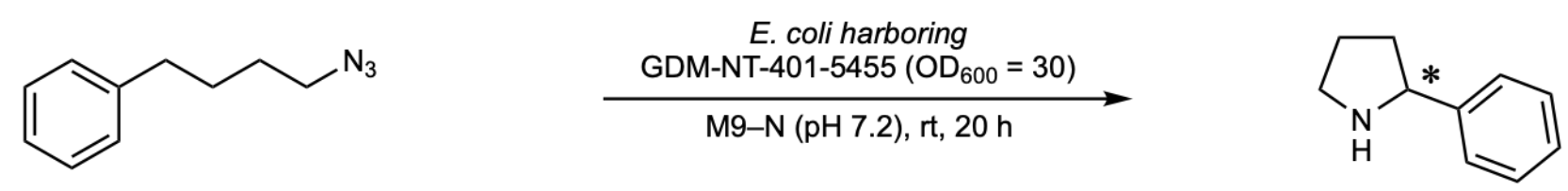

1o **2o**

Reaction performed on analytical scale using **GDM-NT-401-5455**: 15% ee

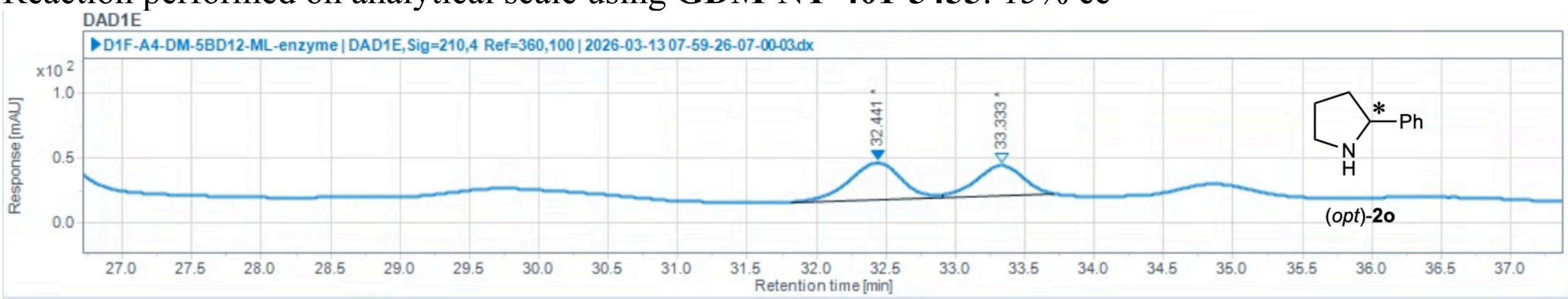

Injection Results

Peaks Summary

#	Name	Signal description	RT (min)	Area (mAU·s)	Area%	Height (mAU)	Height%	Amount	Concentration	Start tim
1		DAD1E,Sig=210,4 Ref=360,100	32.441	726.046	57.676	28.015	55.00			
2		DAD1E,Sig=210,4 Ref=360,100	33.333	532.782	42.324	22.920	45.00			

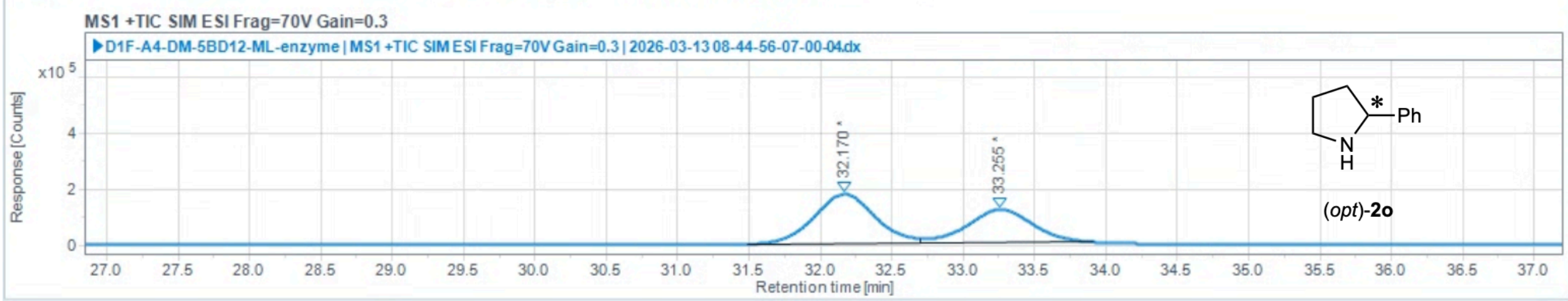

Injection Results

Peaks Summary

#	Name	Signal description	RT (min)	Area (Counts·s)	Area%	Height (Counts)	Height%	Amount	Concentration	Start tim
1		MS1 +TIC SIM ESI Frag=70V Gain=0.3	32.170	5292778.016	58.222	176851.000	59.96			
2		MS1 +TIC SIM ESI Frag=70V Gain=0.3	33.255	3797956.252	41.778	118108.134	40.04			
